# Supplementary material for: Increased expression of peptides from non-coding genes in cancer proteomics datasets suggests potential tumor neoantigens
Source: Commun Biol. 2021 Apr 22;4:496. doi: 10.1038/s42003-021-02007-2 (PMC8062694; doi:10.1038/s42003-021-02007-2)
Supplement: Supplementary file 2 — Supplementary Information [file 42003_2021_2007_MOESM2_ESM.pdf]

Figure S1

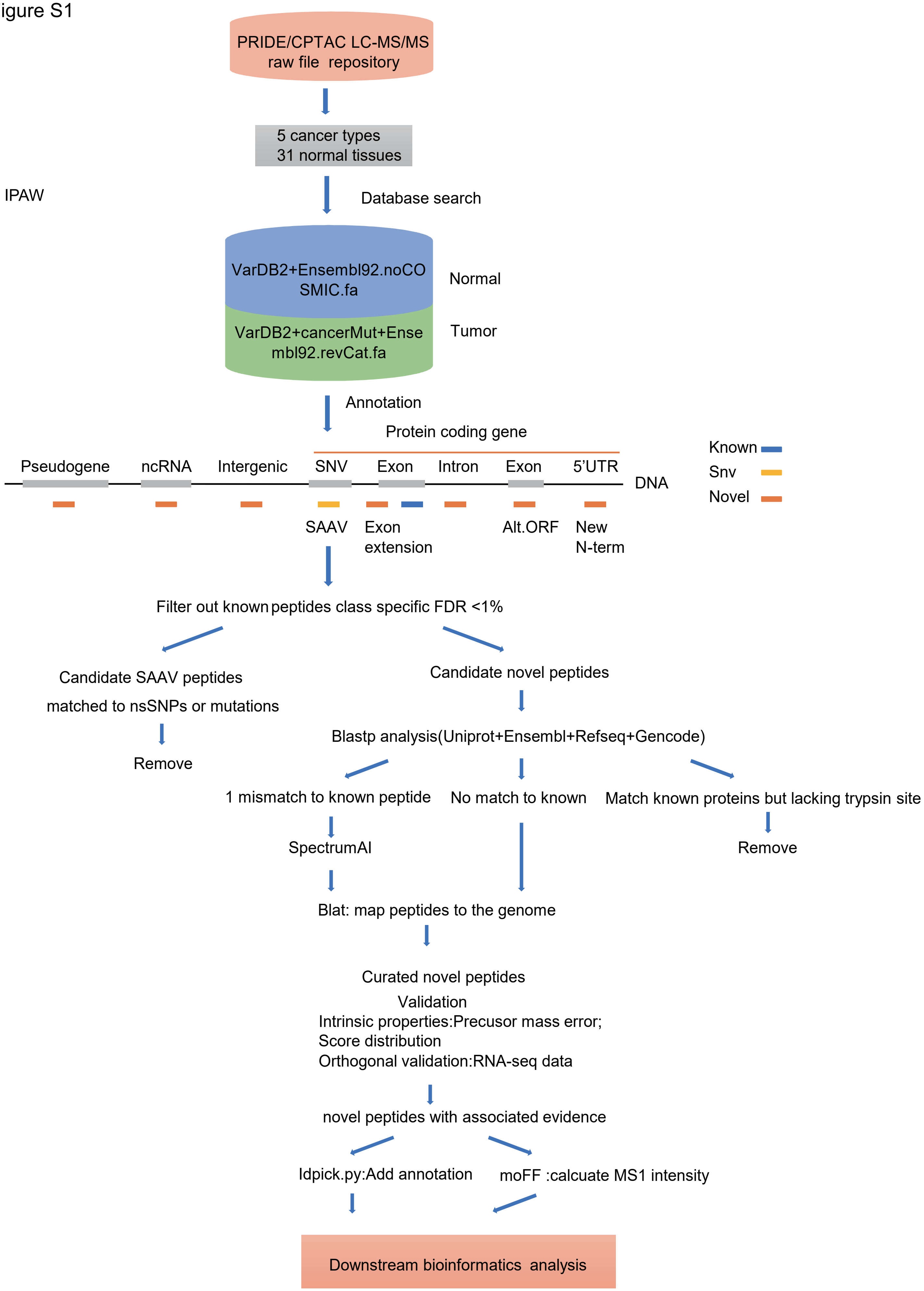
PRIDE/CPTAC LC-MS/MS raw file repository
5 cancer types
31 normal tissues
IPAW
Database search
VarDB2+Ensembl92.noCOSMIC.fa
Normal
VarDB2+cancerMut+Ensembl92.revCat.fa
Tumor
Annotation
Protein coding gene
Pseudogene
ncRNA
Intergenic
SNV
Exon
Intron
Exon
5'UTR
DNA
Known
Snv
Novel
SAAV
Exon extension
Alt.ORF
New N-term
Filter out known peptides class specific FDR <1%
Candidate SAAV peptides
matched to nsSNPs or mutations
Remove
Candidate novel peptides
Blastp analysis(Uniprot+Ensembl+Refseq+Gencode)
1 mismatch to known peptide
No match to known
Match known proteins but lacking trypsin site
SpectrumAI
Remove
Blat: map peptides to the genome
Curated novel peptides
Validation
Intrinsic properties:Precusor mass error;
Score distribution
Orthogonal validation:RNA-seq data
novel peptides with associated evidence
Idpick.py:Add annotation
moFF :calcuate MS1 intensity
Downstream bioinformatics analysis

Figure S1. The workflow of this study. This workflow is modified from a previously published integrated proteogenomics analysis workflow. Firstly, 13 data sets including 5 cancer type and 31 healthy tissues were retrieved from CPTAC and PRIDE database. Second step was to build a custom database. All MS/MS spectra were searched by MSGFPlus in target and decoy combined database. Peptide matches to known proteins, mutant peptide sequences from non-synonymous SNPs or mutations were removed through BLASTP. Then peptides mapping to multiple genomic loci were annotated using BLAT. The retained novel peptides were considered for downstream analysis.

Figure S2

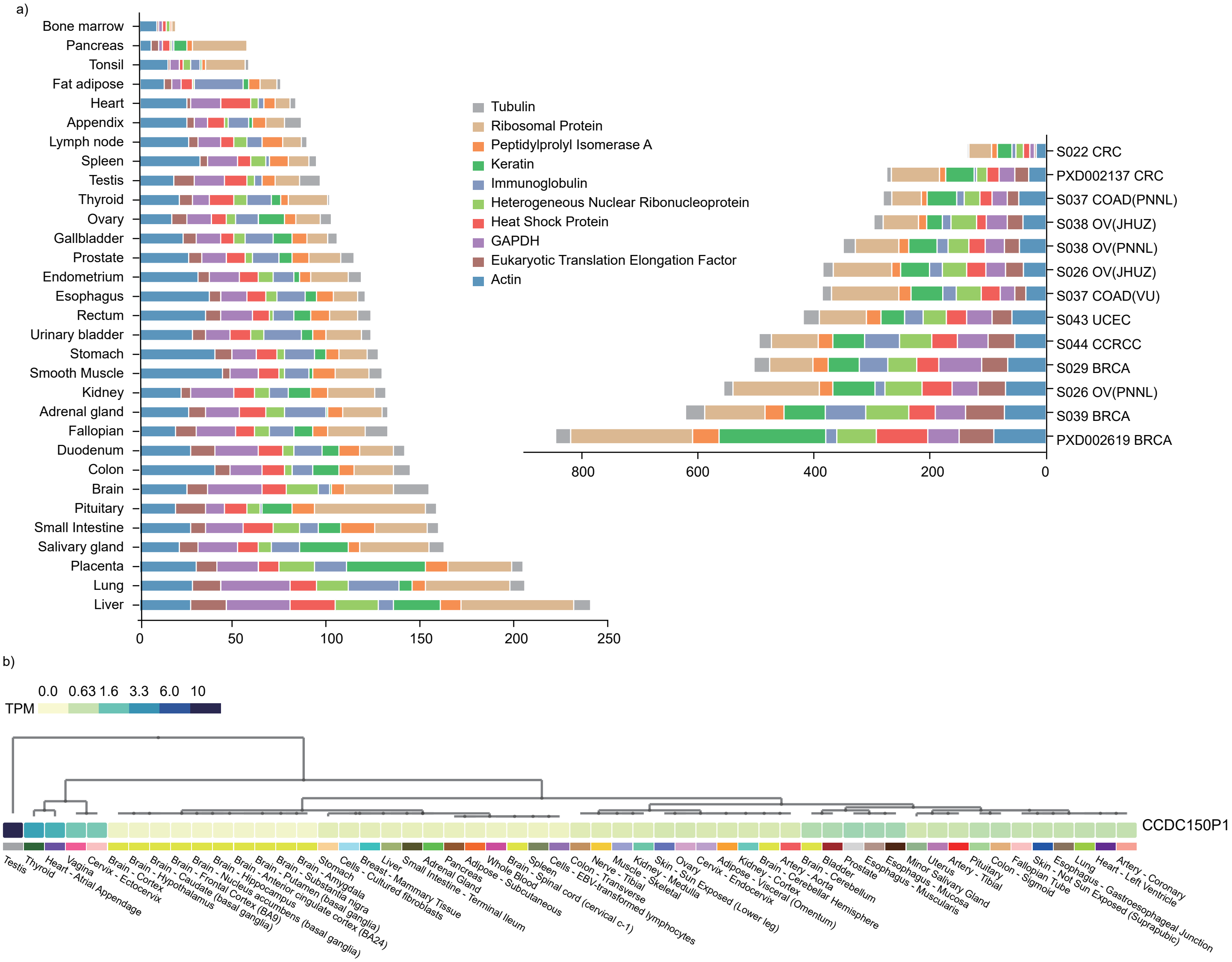

Figure S2. **Pseudogenes' function.** a) annotation of parental genes' function of unfiltered translated pseudogenes (includ single peptide supported). Left: 31 healthy tissues, right: cancer datasets. b) pseudogene CCDC150P1 transcript is specifically expressed in testis according to GTex data.

Figure S3

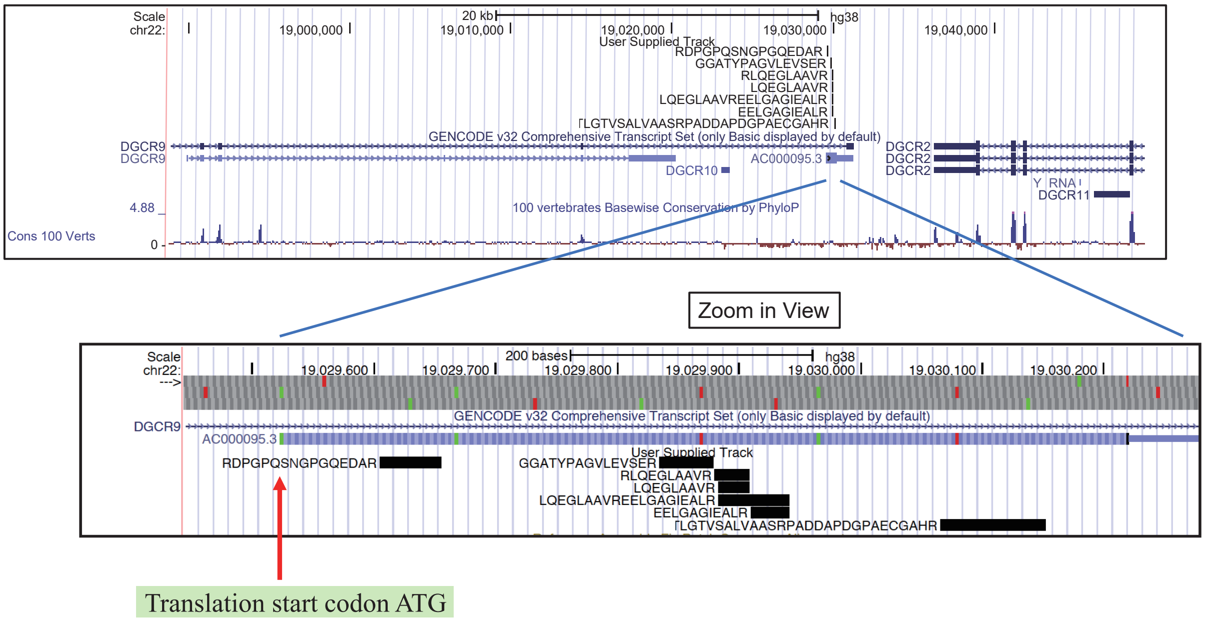

Figure S3. DGCR9 encoded peptides. Starting from 5'end, the peptide RRDPGPQSNGPGQEDAR shares the same reading frame (RF2) with the start codon ATG, however the rest of detected peptides are translated from another reading frame (RF1)
