# Supplementary material for: Increased expression of peptides from non-coding genes in cancer proteomics datasets suggests potential tumor neoantigens
Source: Commun Biol. 2021 Apr 22;4:496. doi: 10.1038/s42003-021-02007-2 (PMC8062694; doi:10.1038/s42003-021-02007-2)
Supplement: Supplementary file 3 — Description of Additional Supplementary Files [file 42003_2021_2007_MOESM3_ESM.pdf]

Description of Additional Supplementary Files

File name: Supplementary Data 1

Description:

File Name: Supplementary Data Table 1

Description: Detailed annotations of the downloaded datasets and sample clinic information.

File Name: Supplementary Data Table 2

Description: Detailed annotations of novel peptides identified from 31 healthy tissues

File Name: Supplementary Data Table 3

Description: Detailed annotations of novel peptides identified from tumor tissues

File Name: Supplementary Data Table 4

Description: Annotated the translated pseudogenes based on their parental genes' functions, related figure 1e.

File Name: Supplementary Data Table 5

Description: Compared our proteomics results with two recent studies

File Name: Supplementary Data Table 6

Description: Neoantigen candidates

File Name: Supplementary Data Table 7

Description: The RNA-seq BAM files (63 breast cancer samples and 10 normal adjacent tissues) were downloaded from TCGA.

File name: Supplementary Data 2

Description: Annotated spectra of peptides of interest (in total 123 spectra of 87 unique peptides).
