# Supplementary material for: Increased expression of peptides from non-coding genes in cancer proteomics datasets suggests potential tumor neoantigens
Source: Commun Biol. 2021 Apr 22;4:496. doi: 10.1038/s42003-021-02007-2 (PMC8062694; doi:10.1038/s42003-021-02007-2)
Supplement: Supplementary file 4 — Supplementary Data 1–2 [file 42003_2021_2007_MOESM4_ESM.zip › Supplementary data/Supplementary Data 2.pdf]

QAQLLAAALQVHQPPPGC+57.021PK

datasets: placenta Scan Number: 31424 precMass: 685.37292 precCharge: 3 Sequence: QAQLLAAALQVHQPPPGCPK Name: lncRNA_lnc−CACNG8−28

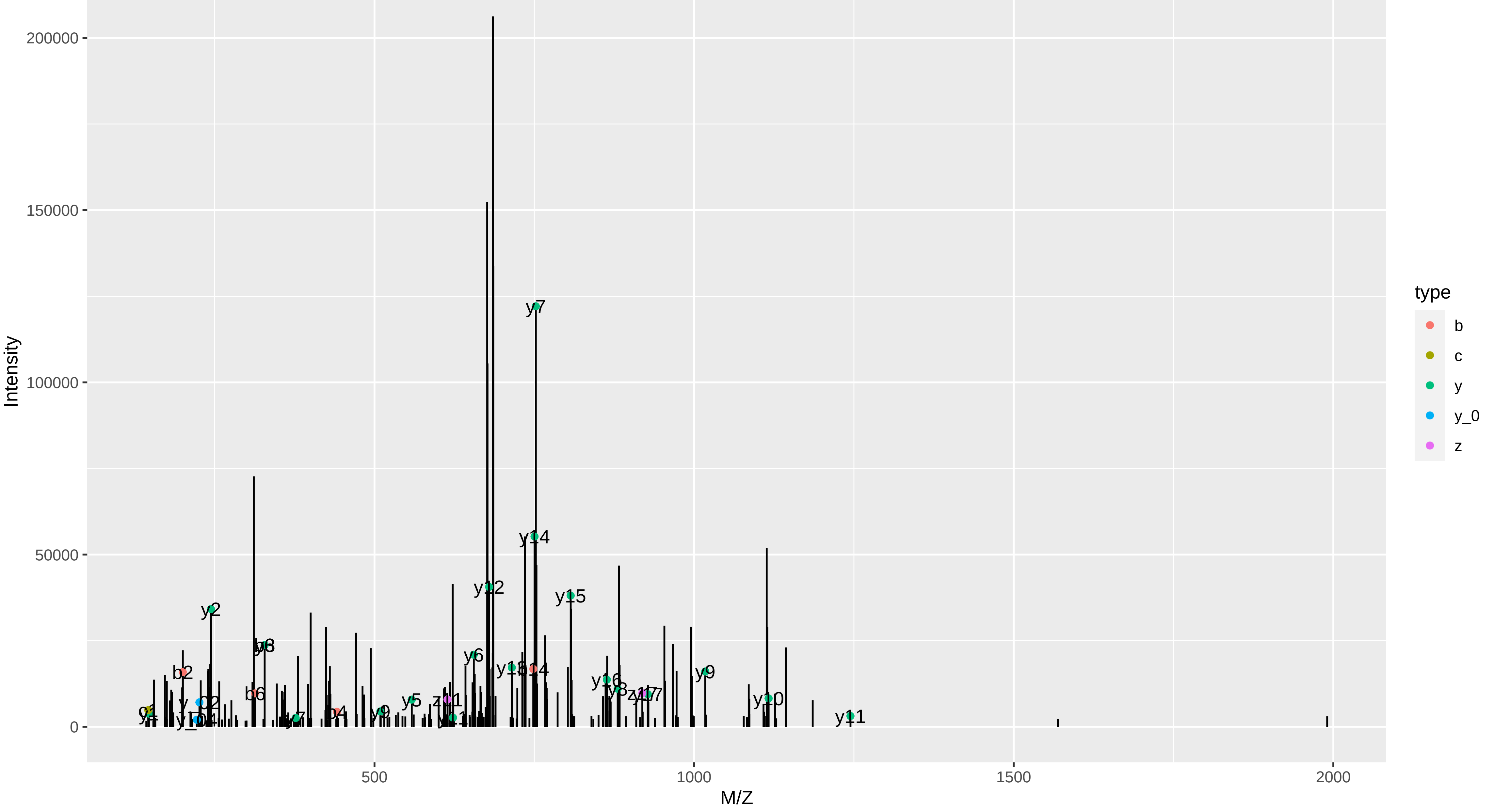

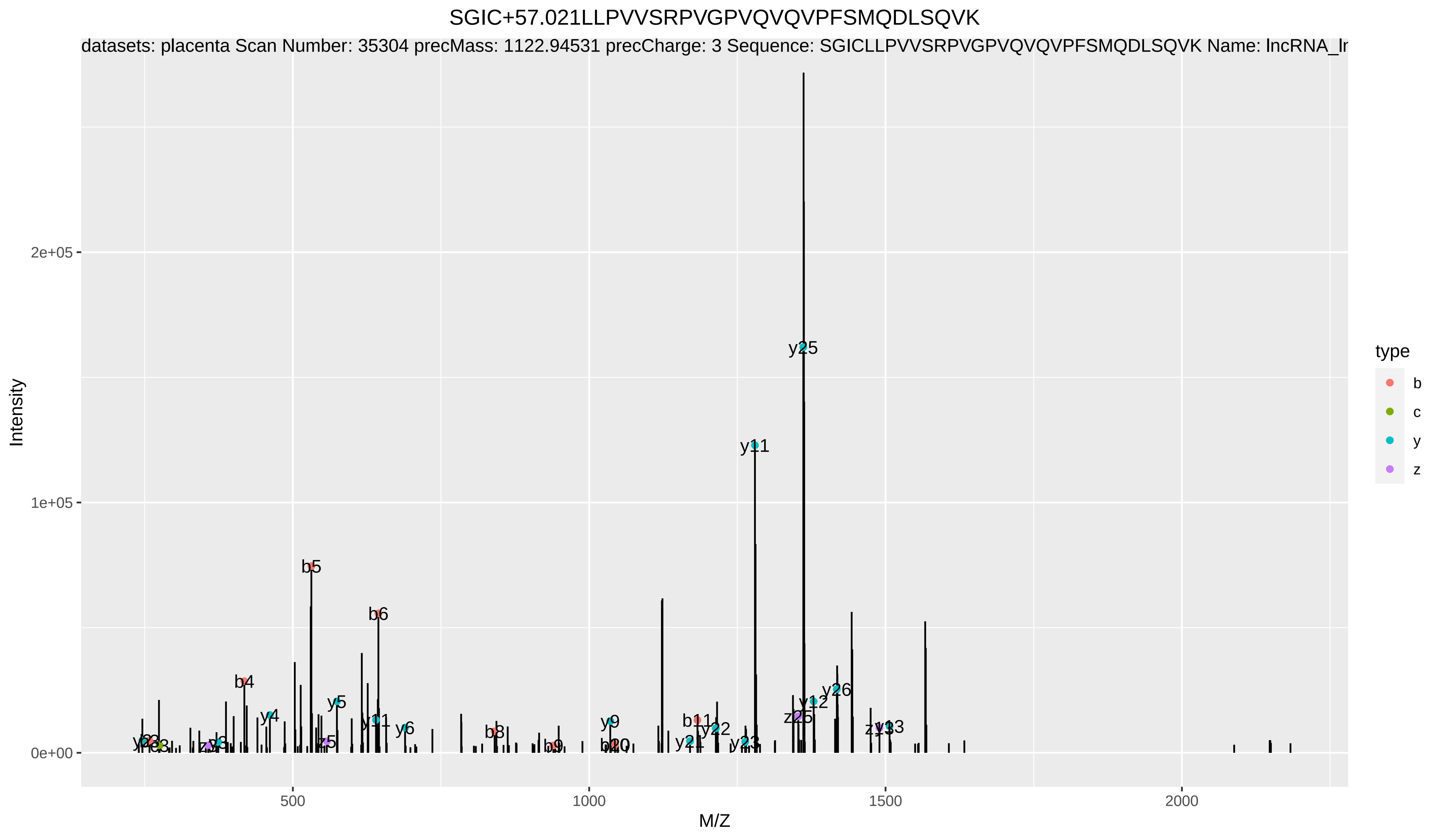

SGIC+57.021LLPVVSRPVGPVQVQVPFSMQDLSQVK
datasets: placenta Scan Number: 35304 precMass: 1122.94531 precCharge: 3 Sequence: SGICLLPVVSRPVGPVQVQVPFSMQDLSQVK Name: lncRNA_lr
Intensity
M/Z
2e+05
1e+05
0e+00
500
1000
1500
2000
type
b
c
y
z
y25
y11
b5
b6
b4
y5
y4
y11
y6
b8
b9
y9
b10
b11
y21
y22
y23
z25
y12
y26
y13

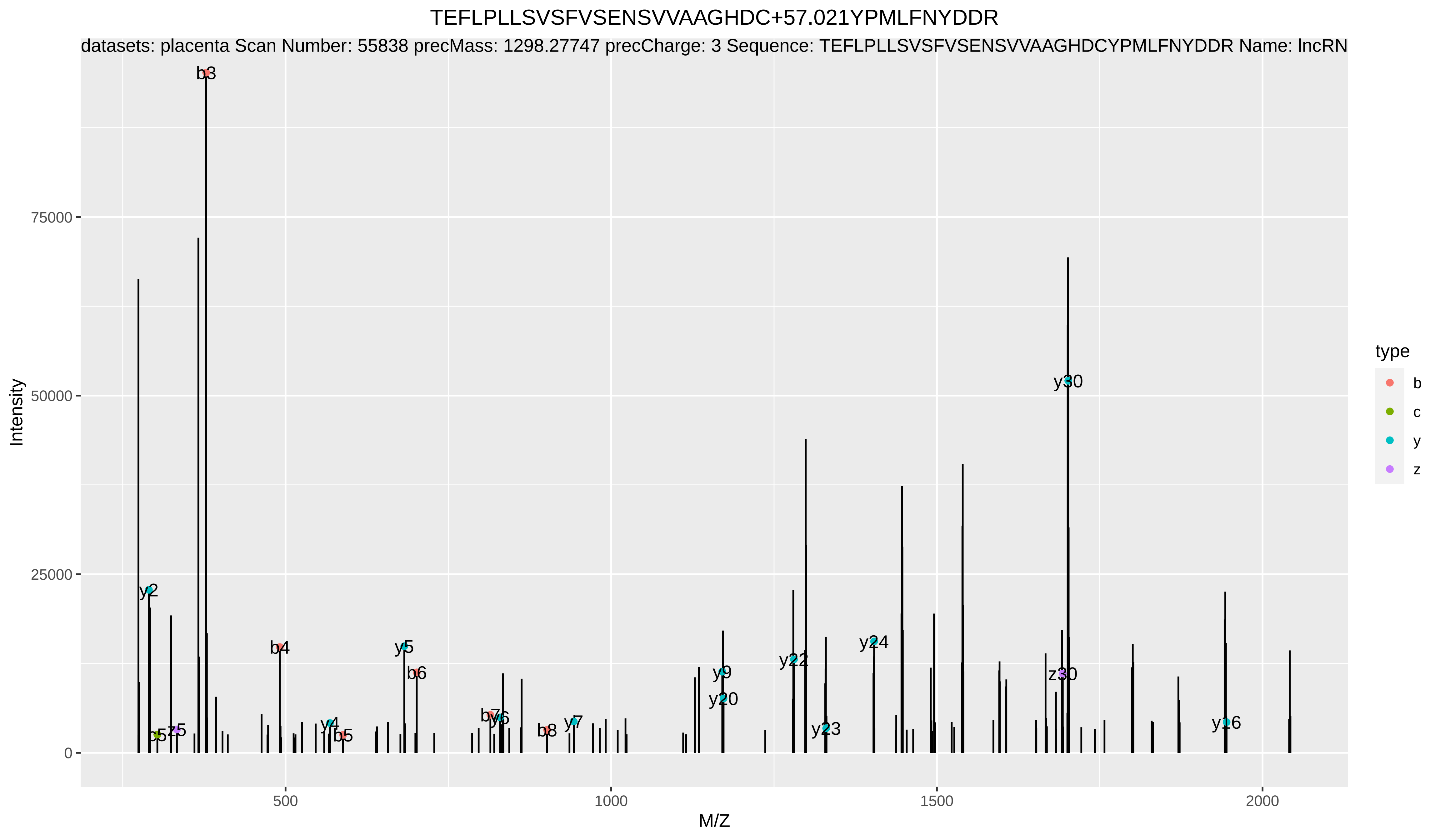
TEFLPLLSVSFVSENSVVAAGHDC+57.021YPMLFNYDDR
datasets: placenta Scan Number: 55838 precMass: 1298.27747 precCharge: 3 Sequence: TEFLPLLSVSFVSENSVVAAGHDCYPMLFNYDDR Name: lncRN
Intensity
M/Z
type
b
c
y
z
b3
y30
y2
b4
y5
b6
y24
y22
y9
z30
y20
b7
y6
y7
b8
y4
b5
c5
z5
y23
y16
75000
50000
25000
0
500
1000
1500
2000

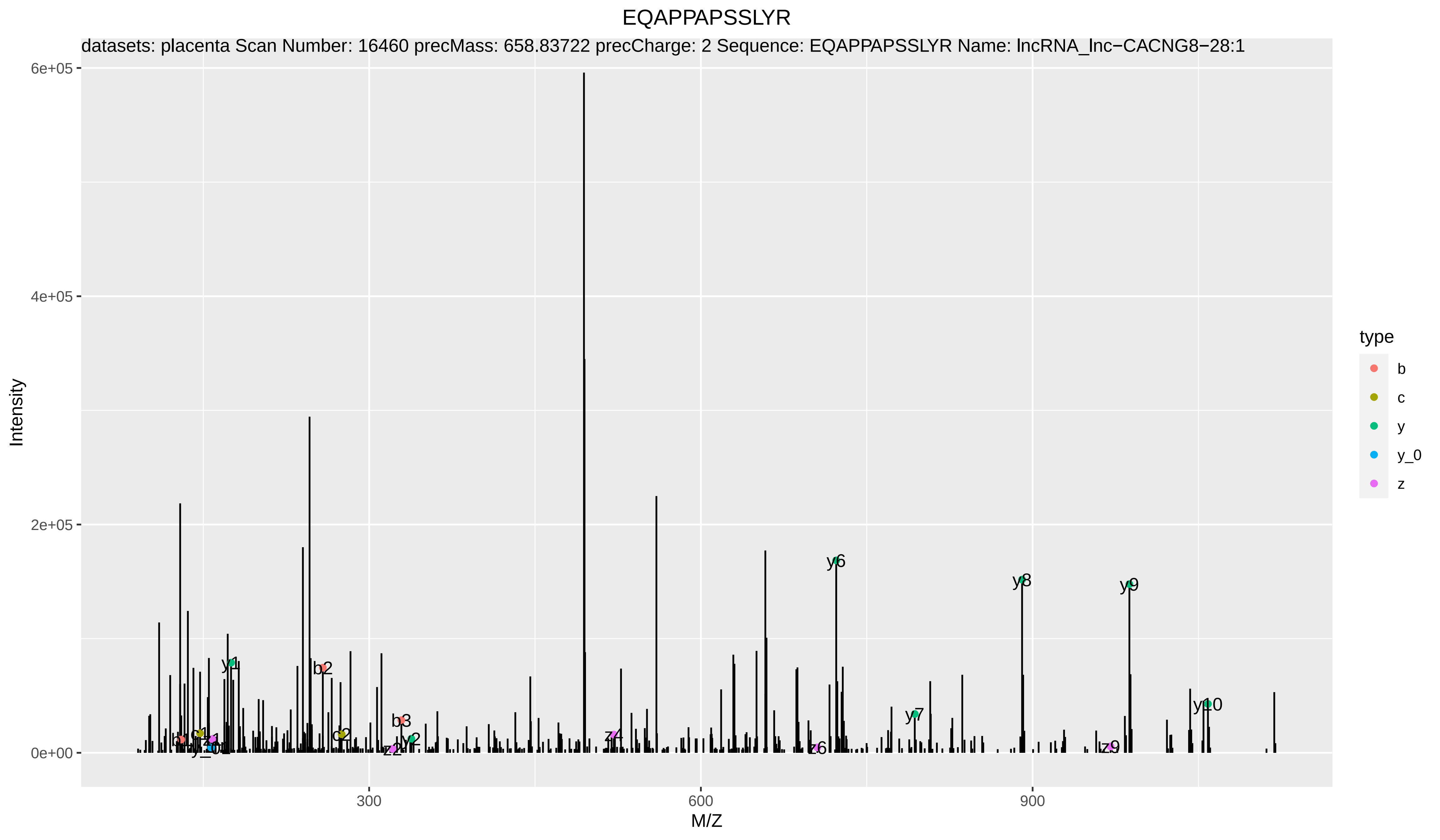

EQAPPAPSSLYR
datasets: placenta Scan Number: 16460 precMass: 658.83722 precCharge: 2 Sequence: EQAPPAPSSLYR Name: lncRNA_lnc−CACNG8−28:1
Intensity
M/Z
6e+05
4e+05
2e+05
0e+00
300
600
900
type
b
c
y
y_0
z
y6
y8
y9
y10
y7
y1
b2
b3
c2
y2
z2
z4
z6
z9
b1
c1
z1
y_0

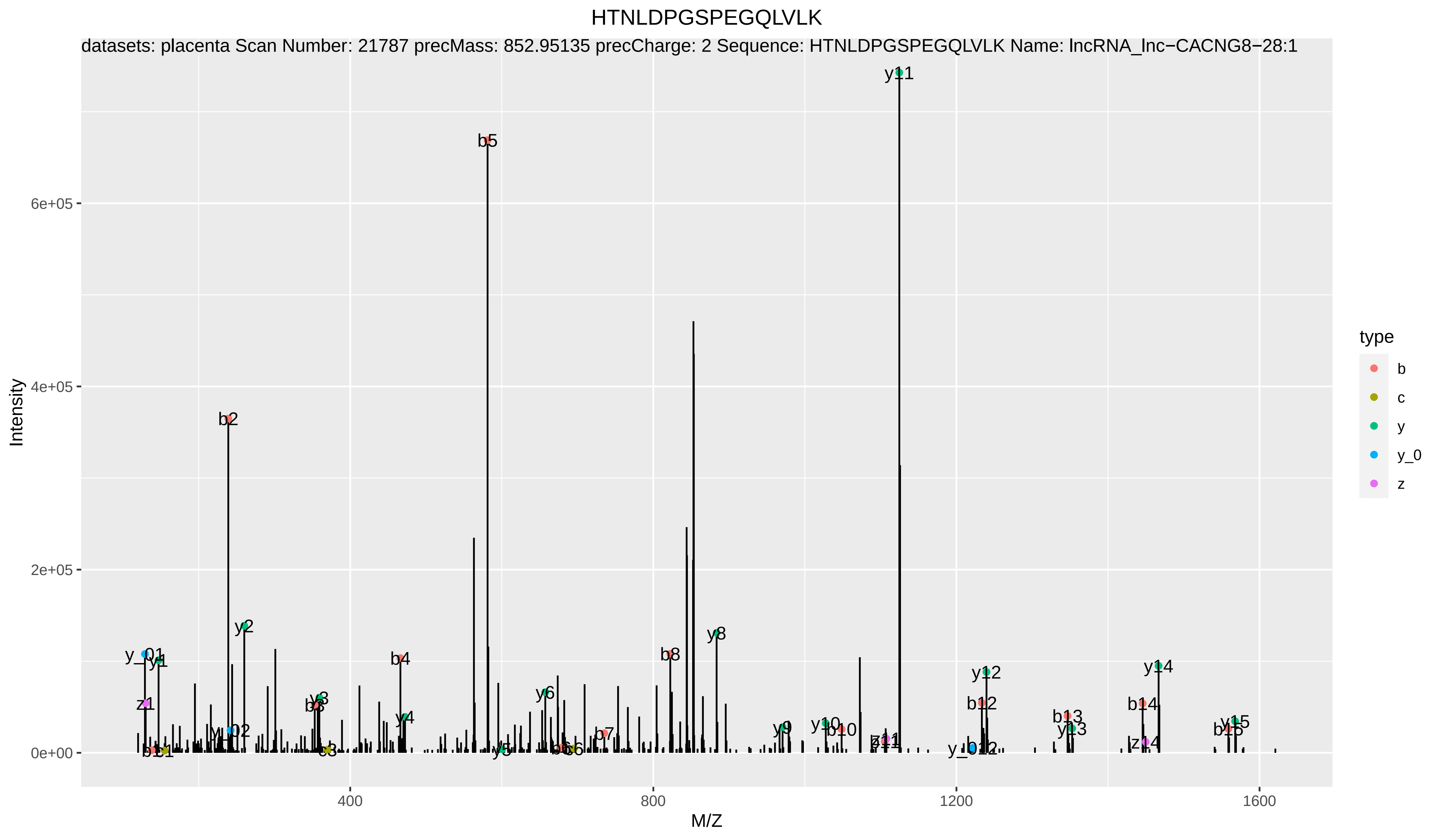
HTNLDPGSPEGQLVLK
datasets: placenta Scan Number: 21787 precMass: 852.95135 precCharge: 2 Sequence: HTNLDPGSPEGQLVLK Name: lncRNA_lnc−CACNG8−28:1
Intensity
M/Z
type
b
c
y
y_0
z
0e+00
2e+05
4e+05
6e+05
400
800
1200
1600
y11
b5
b2
y2
y_01
y1
z1
b4
y6
b8
y8
y12
y14
b12
b14
b13
y13
y15
b15
y10
b10
y9
y4
y3
b3
y_02
b7
b6
c6
c3
y5
b1
c1
z11
b11
y_012
z14

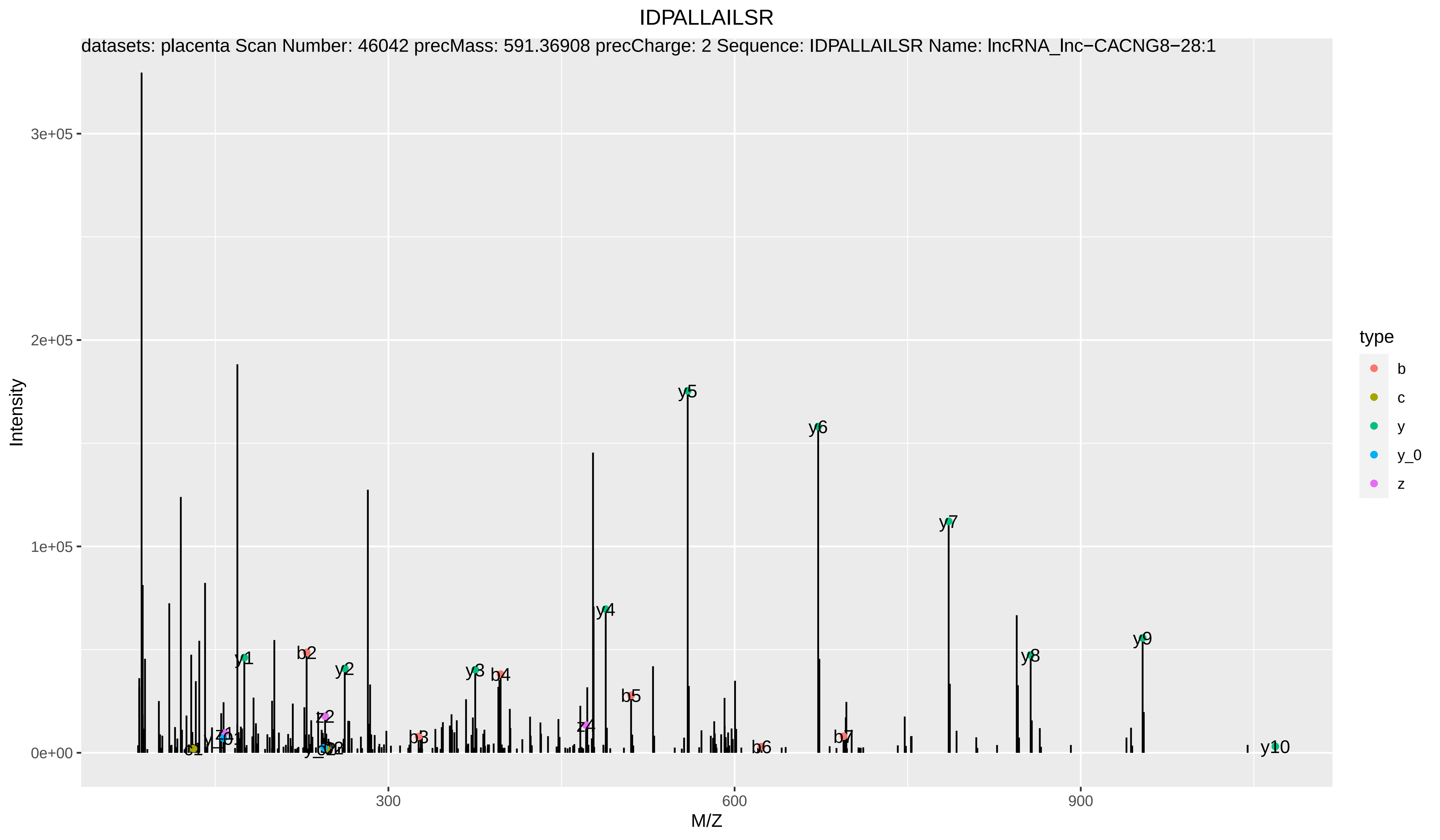

IDPALLAILSR
datasets: placenta Scan Number: 46042 precMass: 591.36908 precCharge: 2 Sequence: IDPALLAILSR Name: lncRNA_lnc−CACNG8−28:1
Intensity
M/Z
3e+05
2e+05
1e+05
0e+00
300
600
900
type
b
c
y
y_0
z
y1
b2
y2
z2
y3
b4
b3
z4
y4
b5
y5
b6
y6
b7
y7
y8
y9
y10
c1

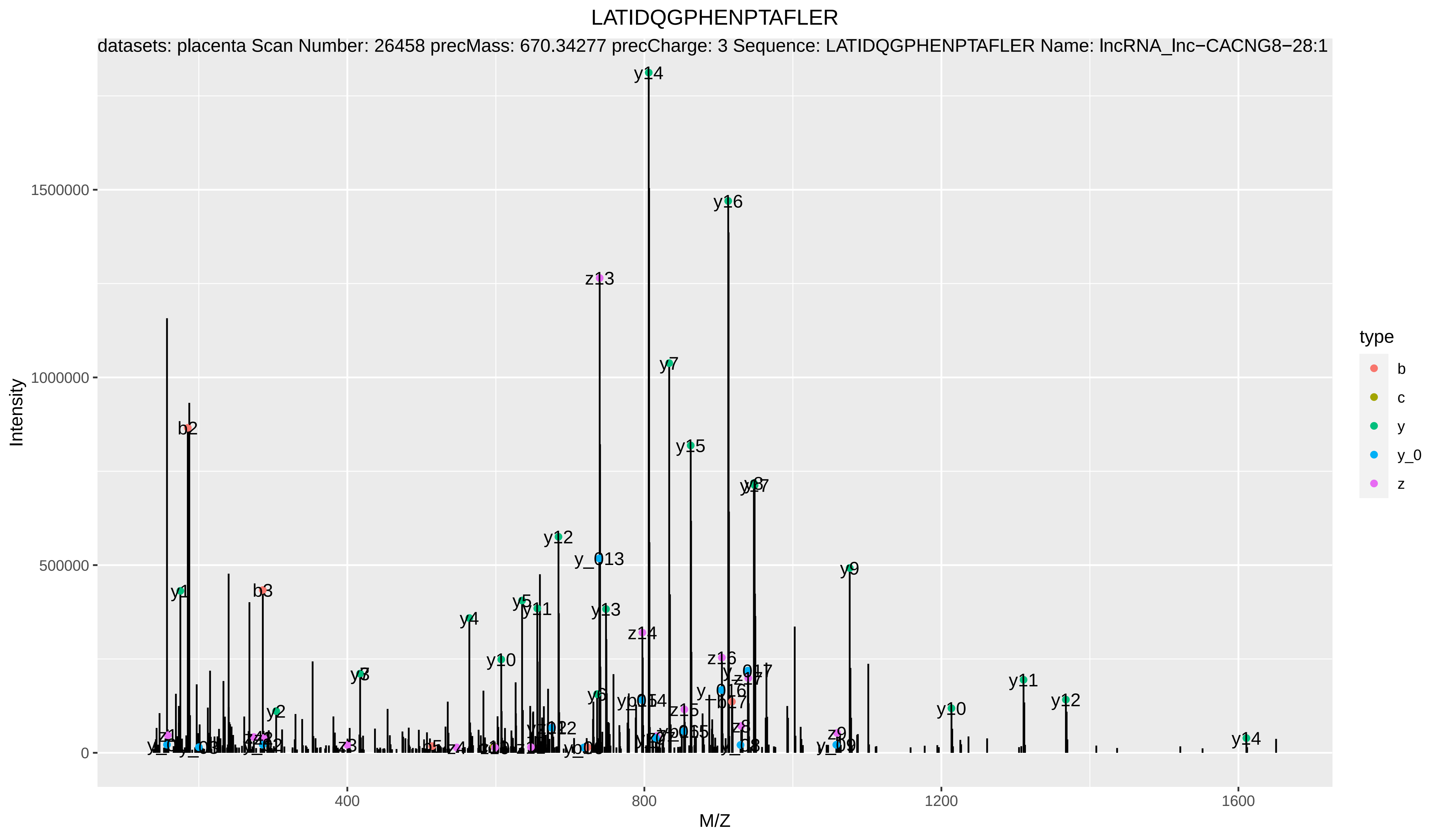
LATIDQGPHENPTAFLER
datasets: placenta Scan Number: 26458 precMass: 670.34277 precCharge: 3 Sequence: LATIDQGPHENPTAFLER Name: lncRNA_lnc−CACNG8−28:1
Intensity
1500000
1000000
500000
0
M/Z
400
800
1200
1600
type
b
c
y
y_0
z
y14
y16
z13
y7
y15
y12
y_013
y9
b2
y1
b3
y4
y5
y11
y13
z14
y10
z16
y3
y2
y6
y11
y12
y10
y14
z3
z9
z8
z15

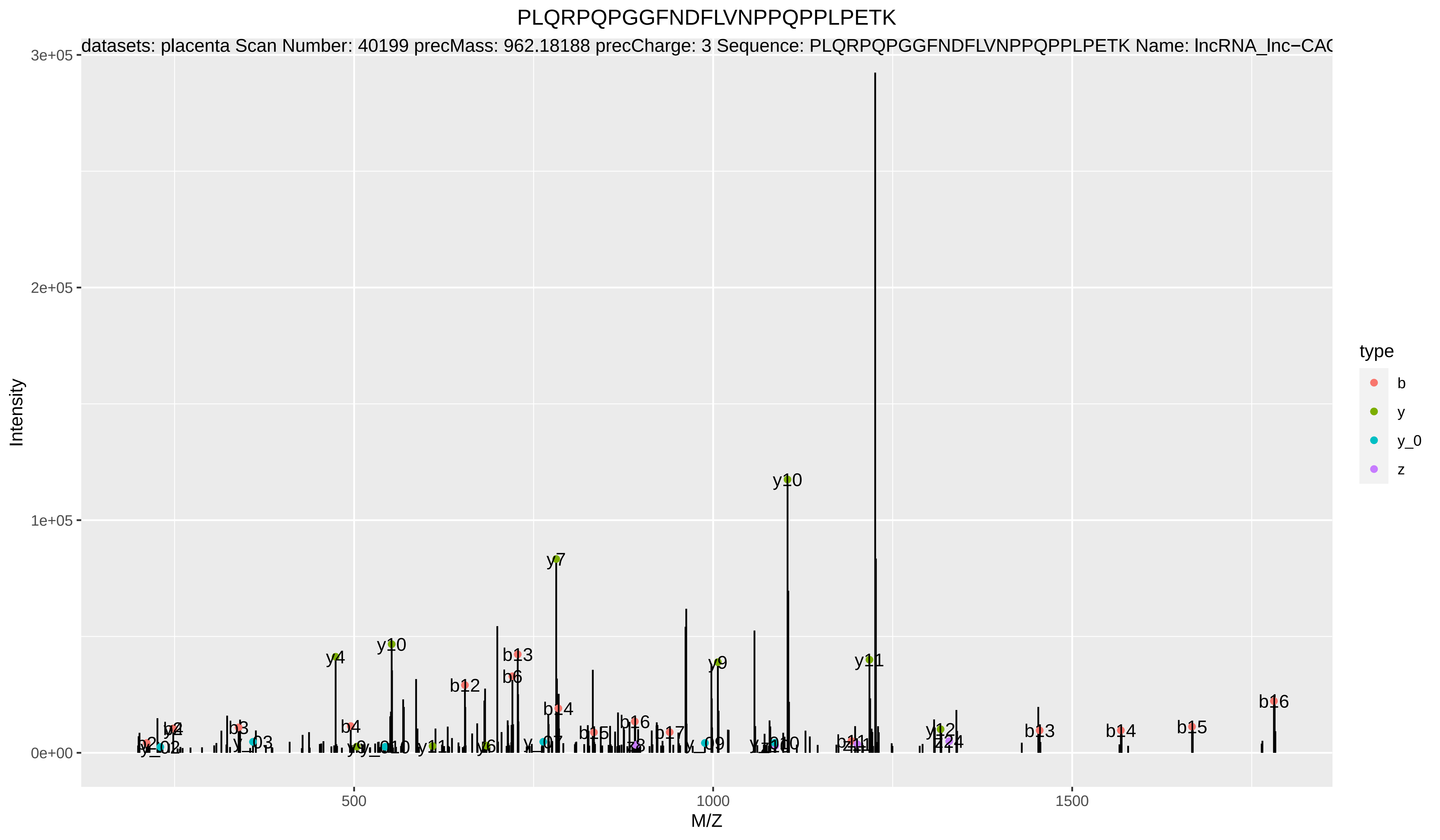
PLQRPQPGGFNDFLVNPPQPPLPETK
datasets: placenta Scan Number: 40199 precMass: 962.18188 precCharge: 3 Sequence: PLQRPQPGGFNDFLVNPPQPPLPETK Name: lncRNA_lnc−CAC
Intensity
M/Z
3e+05
2e+05
1e+05
0e+00
500
1000
1500
type
b
y
y_0
z
y10
y7
y4
b13
b6
b12
y9
y11
b14
b16
b15
b17
y12
b13
b14
b15
b16
b3
b4

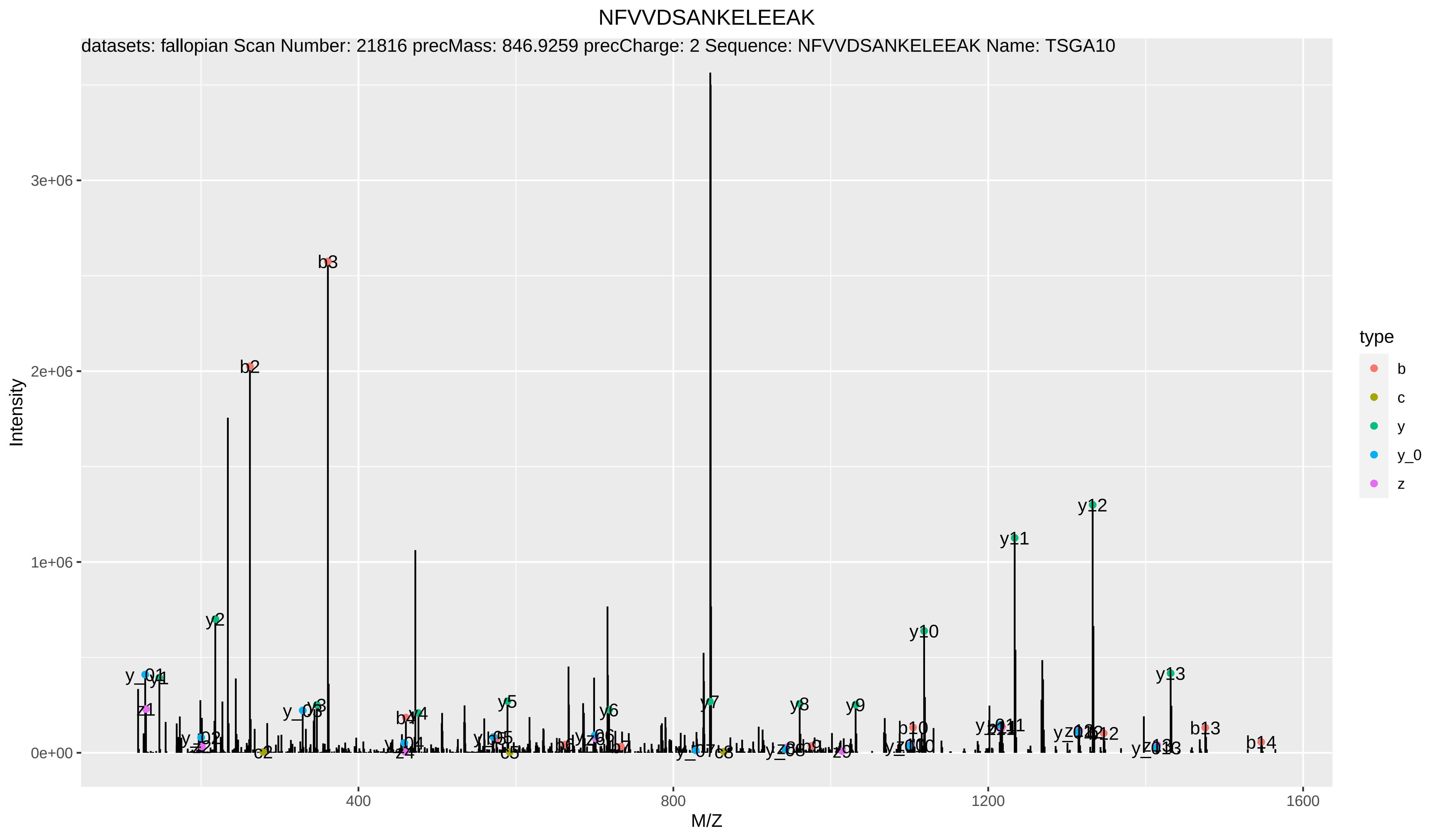
NFVVDSANKELEEAK
datasets: fallopian Scan Number: 21816 precMass: 846.9259 precCharge: 2 Sequence: NFVVDSANKELEEAK Name: TSGA10
Intensity
3e+06
2e+06
1e+06
0e+00
M/Z
400
800
1200
1600
type
b
c
y
y_0
z
b2
b3
y2
y5
y6
y7
y8
y9
b10
y10
y11
y12
y13
b13
b14

IDQLAEQLEKEK

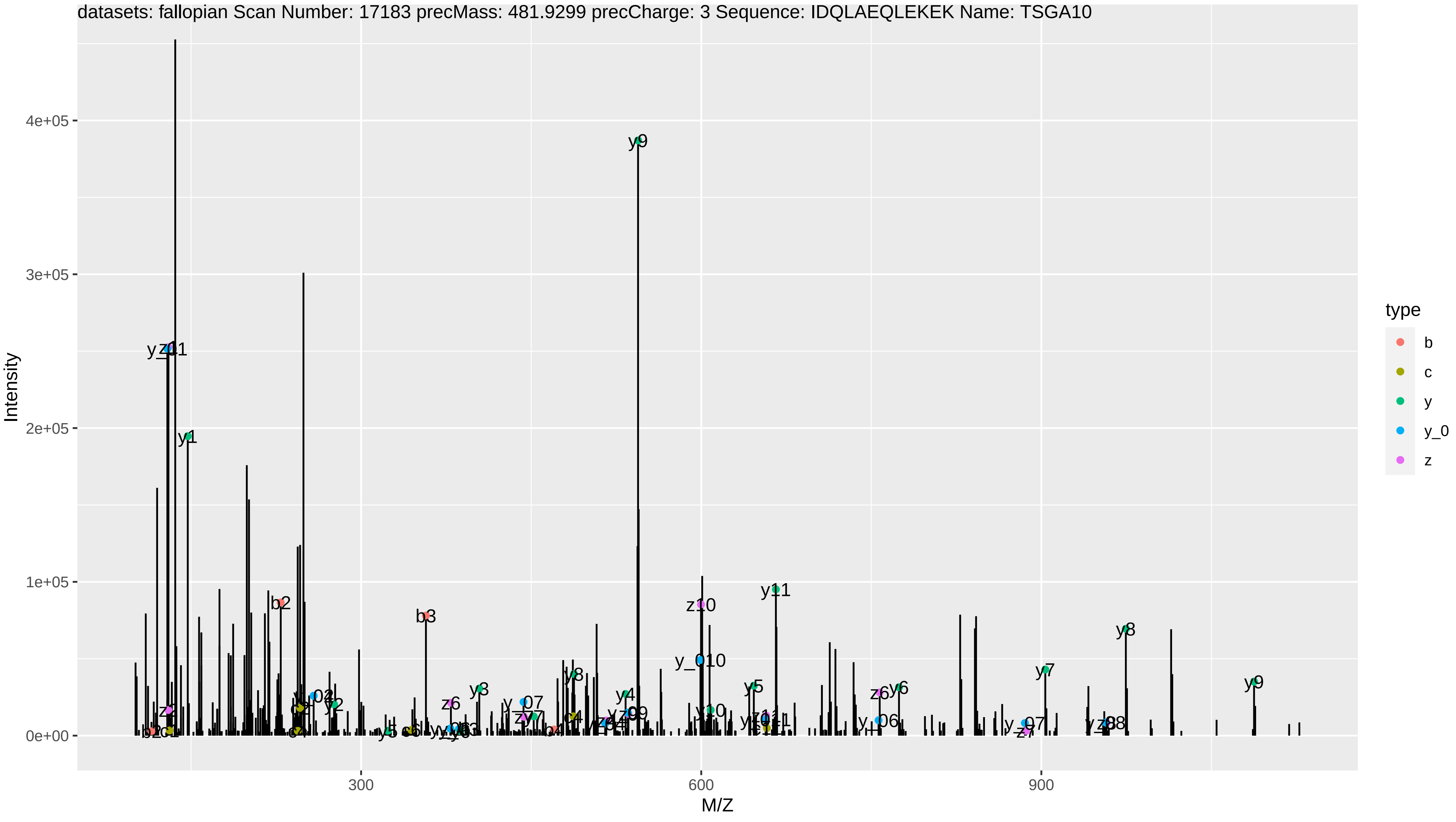

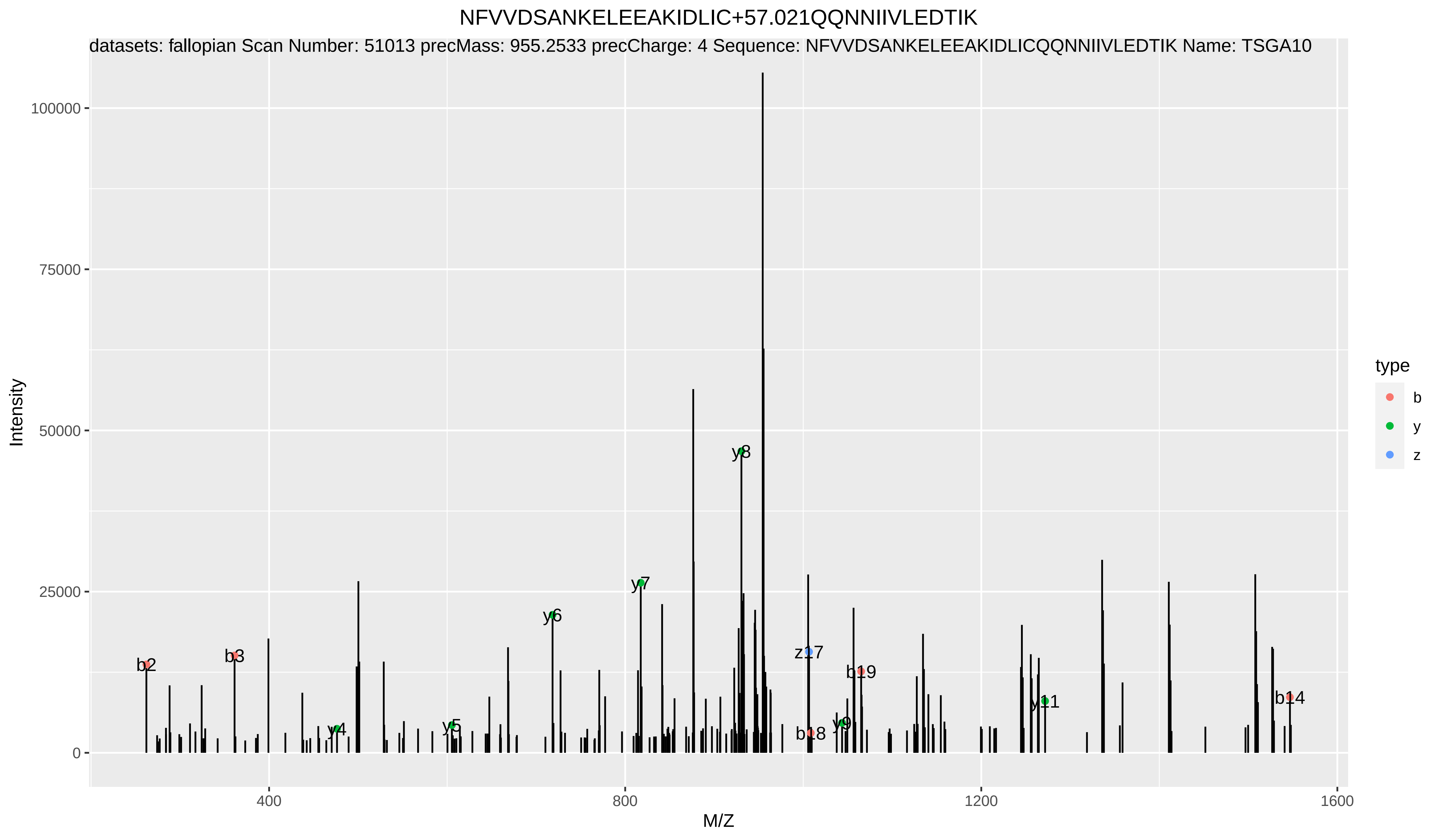
NFVVDSANKELEEAKIDLIC+57.021QQNNIIVLEDTIK
datasets: fallopian Scan Number: 51013 precMass: 955.2533 precCharge: 4 Sequence: NFVVDSANKELEEAKIDLICQQNNIIVLEDTIK Name: TSGA10
Intensity
100000
75000
50000
25000
0
400
800
1200
1600
M/Z
b2
b3
y4
y5
y6
y7
y8
z17
b18
y9
b19
y11
b14
type
b
y
z

QTNHGLEEYVR

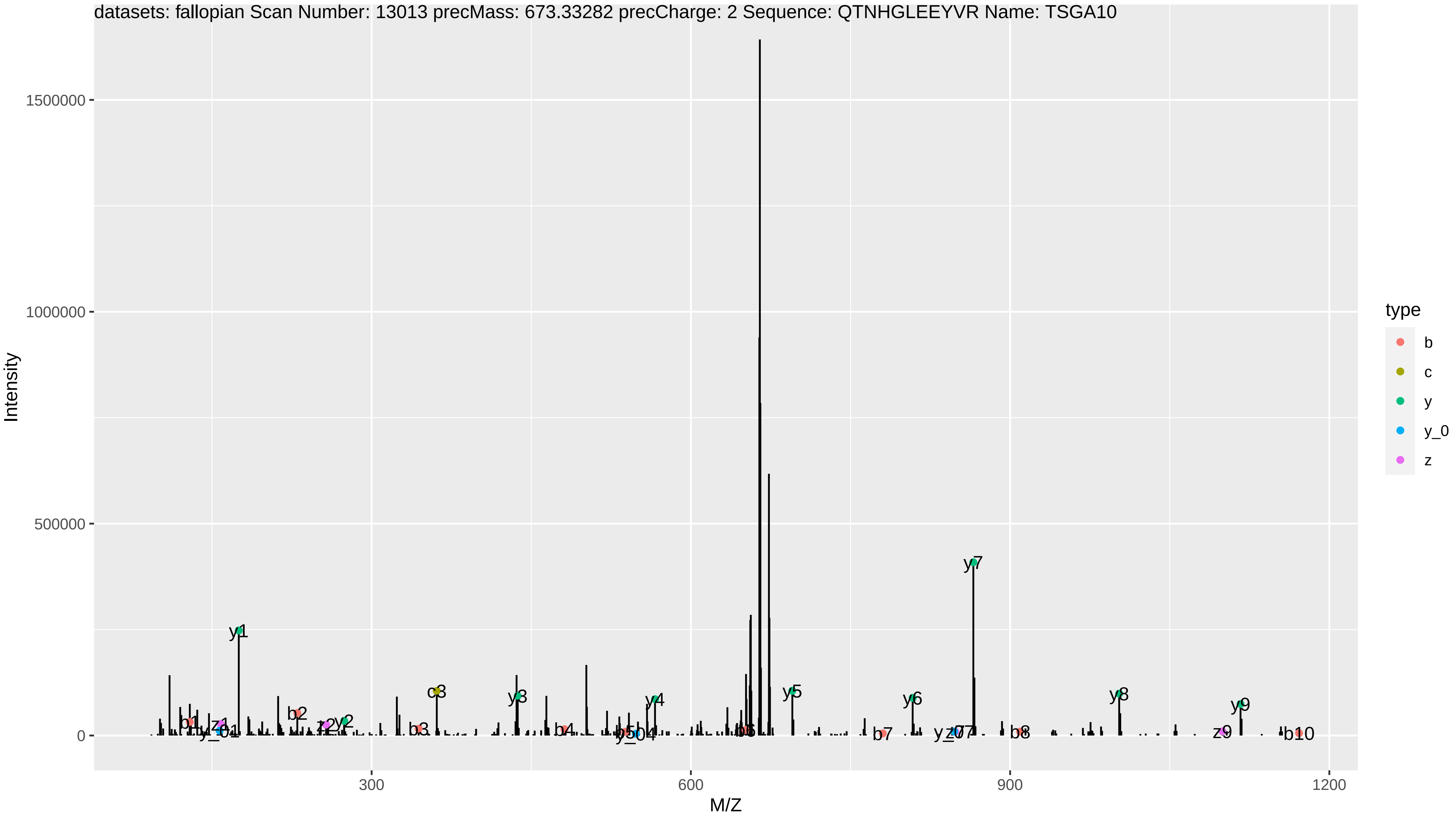

SPGRDPELQVEAAEVTTK

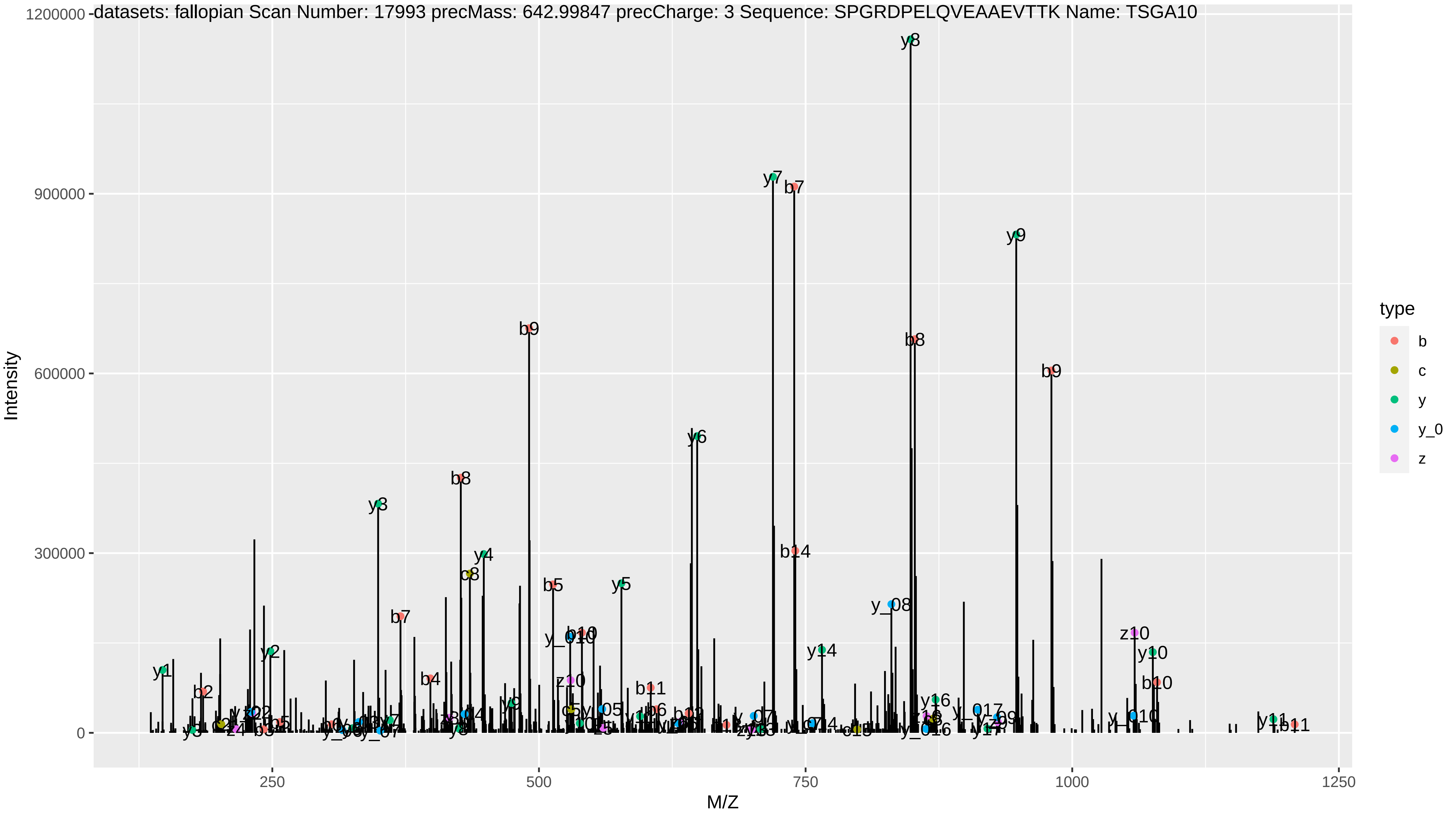

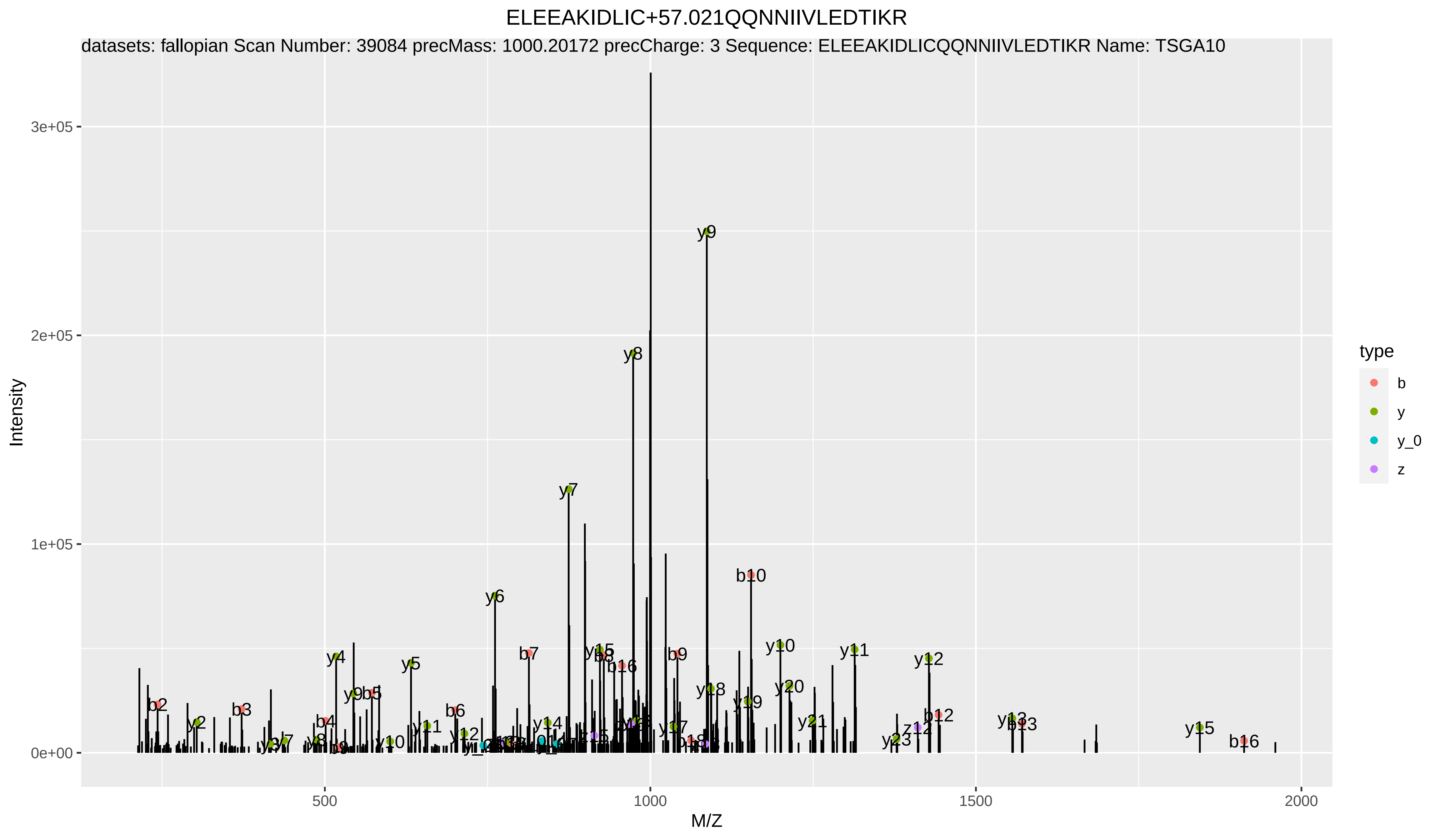
ELEEAKIDLIC+57.021QQNNIIVLEDTIKR
datasets: fallopian Scan Number: 39084 precMass: 1000.20172 precCharge: 3 Sequence: ELEEAKIDLICQQNNIIVLEDTIKR Name: TSGA10
Intensity
3e+05
2e+05
1e+05
0e+00
M/Z
500
1000
1500
2000
type
b
y
y_0
z
y9
y8
y7
y6
b10
y10
y11
y12
b7
b9
b16
y4
y5
y20
y18
y19
b2
b3
b6
b12
y13
b13
y15
b16
y21
y23
y14
y17
y10
y11
y12

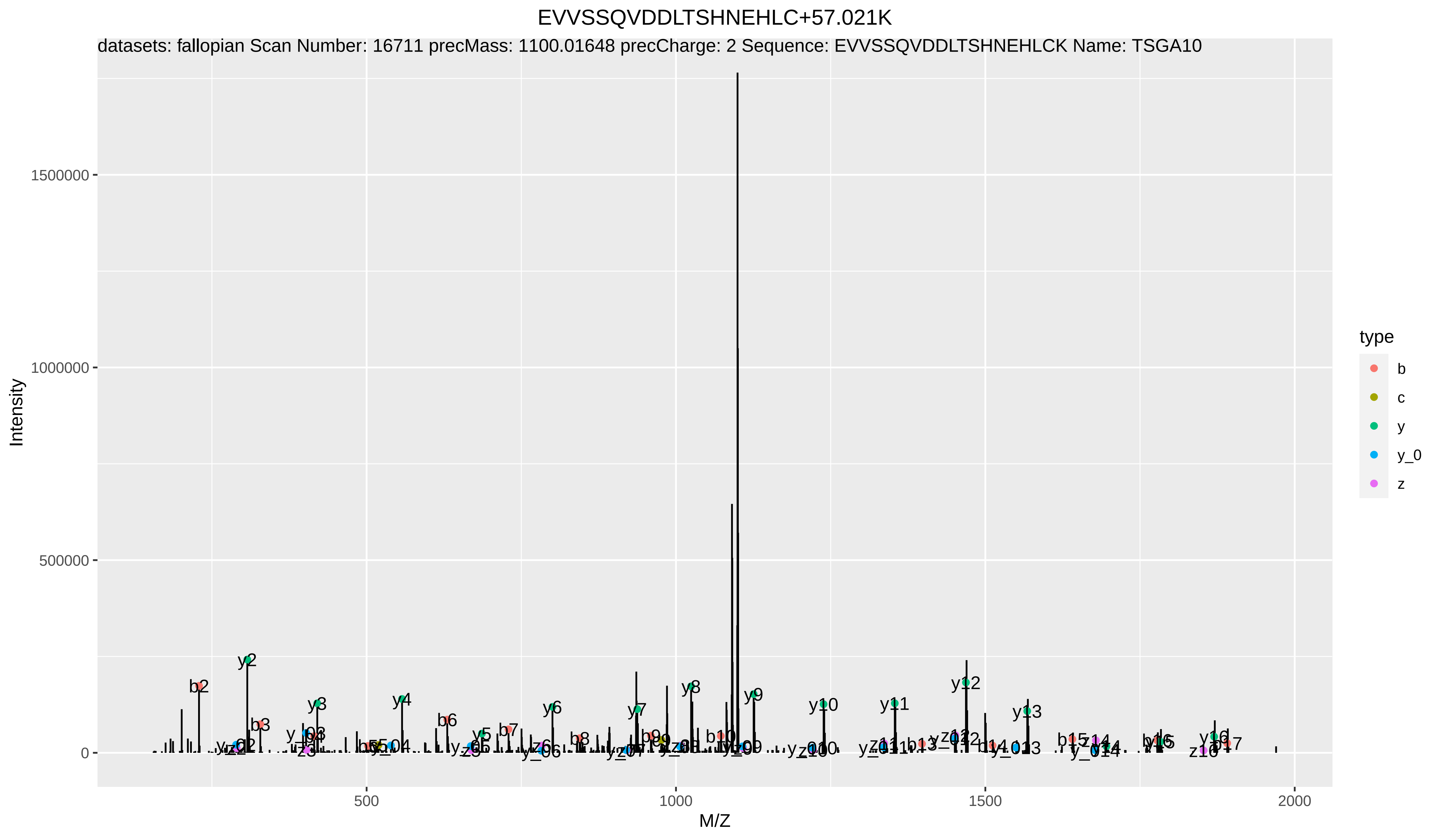
EVVSSQVDDLTSHNEHLC+57.021K
datasets: fallopian Scan Number: 16711 precMass: 1100.01648 precCharge: 2 Sequence: EVVSSQVDDLTSHNEHLCK Name: TSGA10
Intensity
M/Z
0
500000
1000000
1500000
500
1000
1500
2000
type
b
c
y
y_0
z
b2
y2
y3
b3
y4
b6
y5
b7
y6
b8
y7
y8
b10
y9
y10
y11
y12
y13
b15
y16
b17
z16

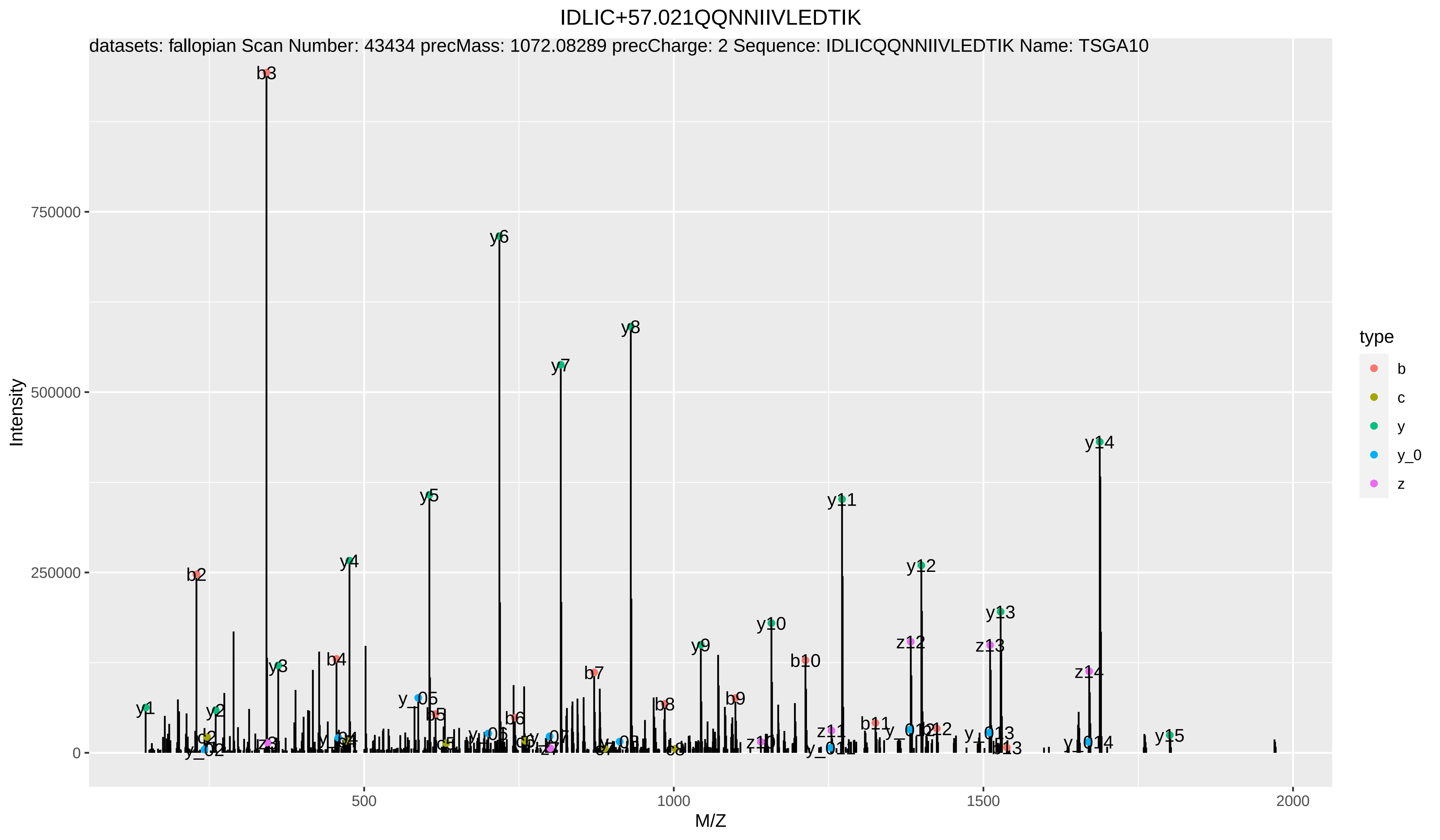
IDLIC+57.021QQNNIIVLEDTIK
datasets: fallopian Scan Number: 43434 precMass: 1072.08289 precCharge: 2 Sequence: IDLICQQNNIIVLEDTIK Name: TSGA10
Intensity
M/Z
0
250000
500000
750000
500
1000
1500
2000
type
b
c
y
y_0
z
b2
b3
b4
b5
b6
b7
b8
b9
b10
b11
b13
y1
y2
y3
y4
y5
y6
y7
y8
y9
y10
y11
y12
y13
y14
y15
z12
z13
z14
z11

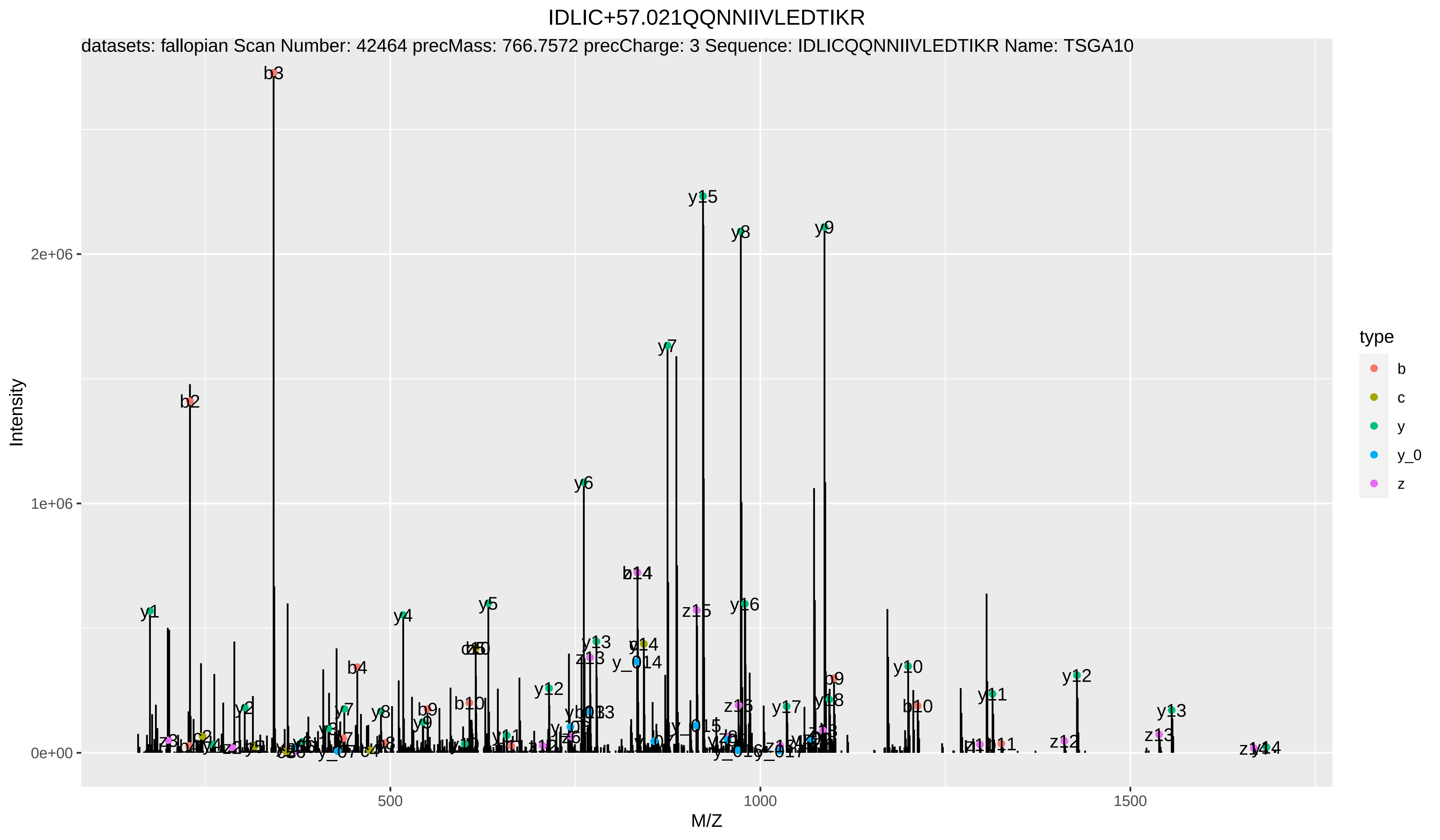
IDLIC+57.021QQNNIIVLEDTIKR
datasets: fallopian Scan Number: 42464 precMass: 766.7572 precCharge: 3 Sequence: IDLICQQNNIIVLEDTIKR Name: TSGA10
Intensity
M/Z
0e+00
1e+06
2e+06
500
1000
1500
type
b
c
y
y_0
z
b3
b2
y15
y8
y9
y7
y6
y1
y4
y5
b4
z15
y16
y10
y12
y11
y13
b10
y17
y18
b9
z12
z13
y14

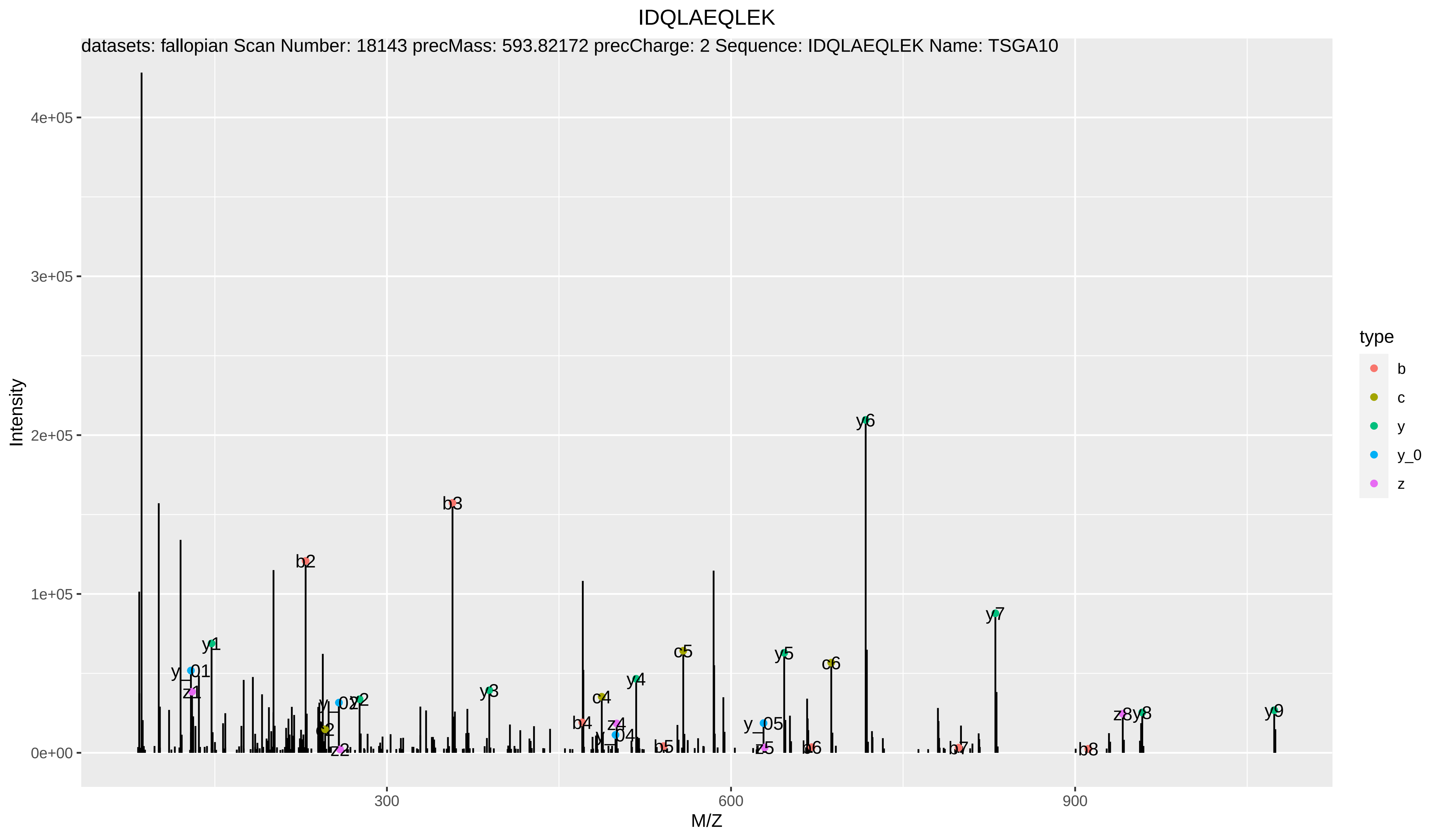

IDQLAEQLEK
datasets: fallopian Scan Number: 18143 precMass: 593.82172 precCharge: 2 Sequence: IDQLAEQLEK Name: TSGA10
Intensity
4e+05
3e+05
2e+05
1e+05
0e+00
300
600
900
M/Z
type
b
c
y
y_0
z
b2
b3
b4
b5
b6
b7
b8
c2
c4
c5
c6
y1
y2
y3
y4
y5
y6
y7
y8
y9
y_01
y_02
y_04
y_05
z1
z2
z4
z5
z8

IIVETDAPFFLPR

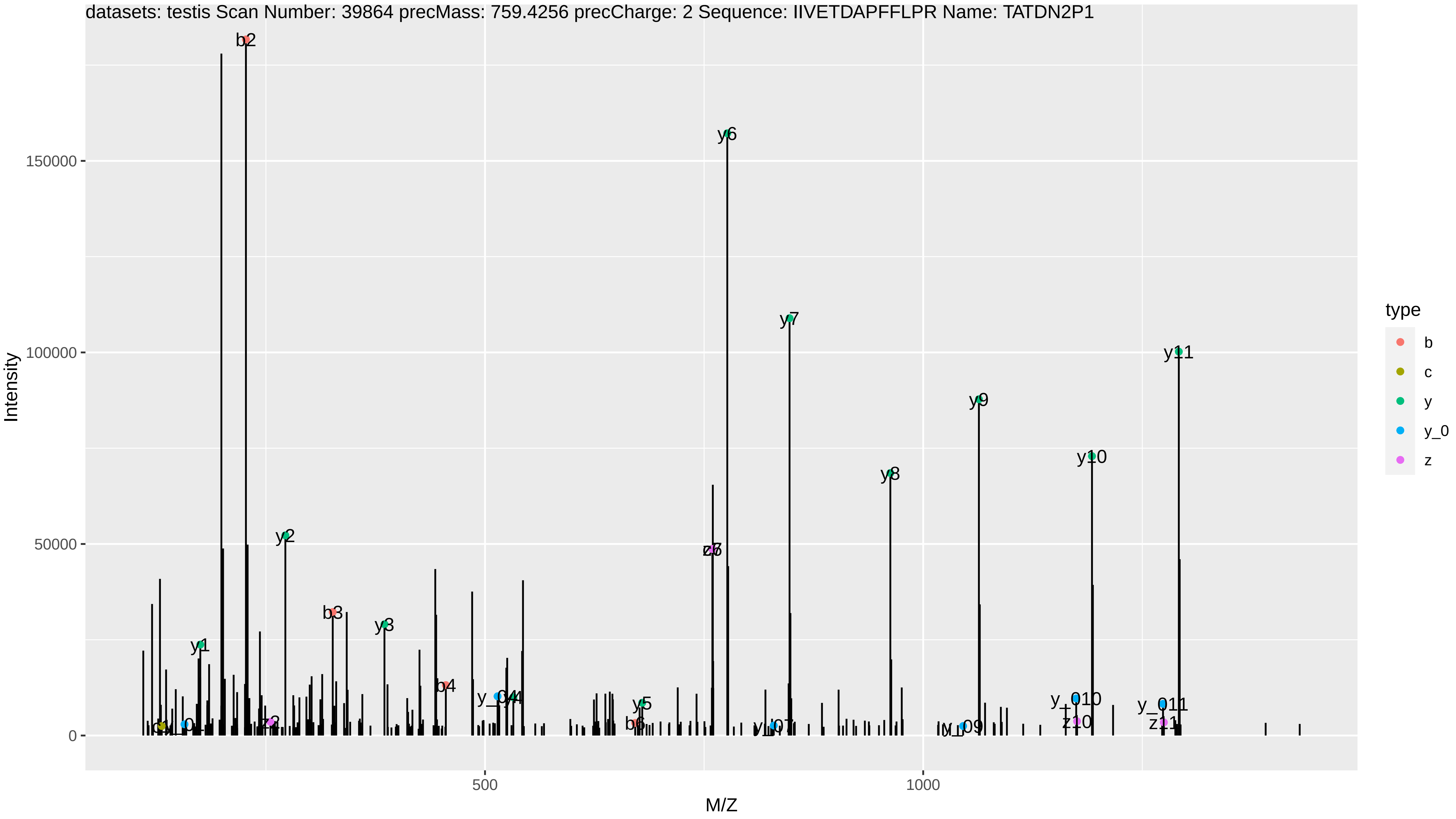

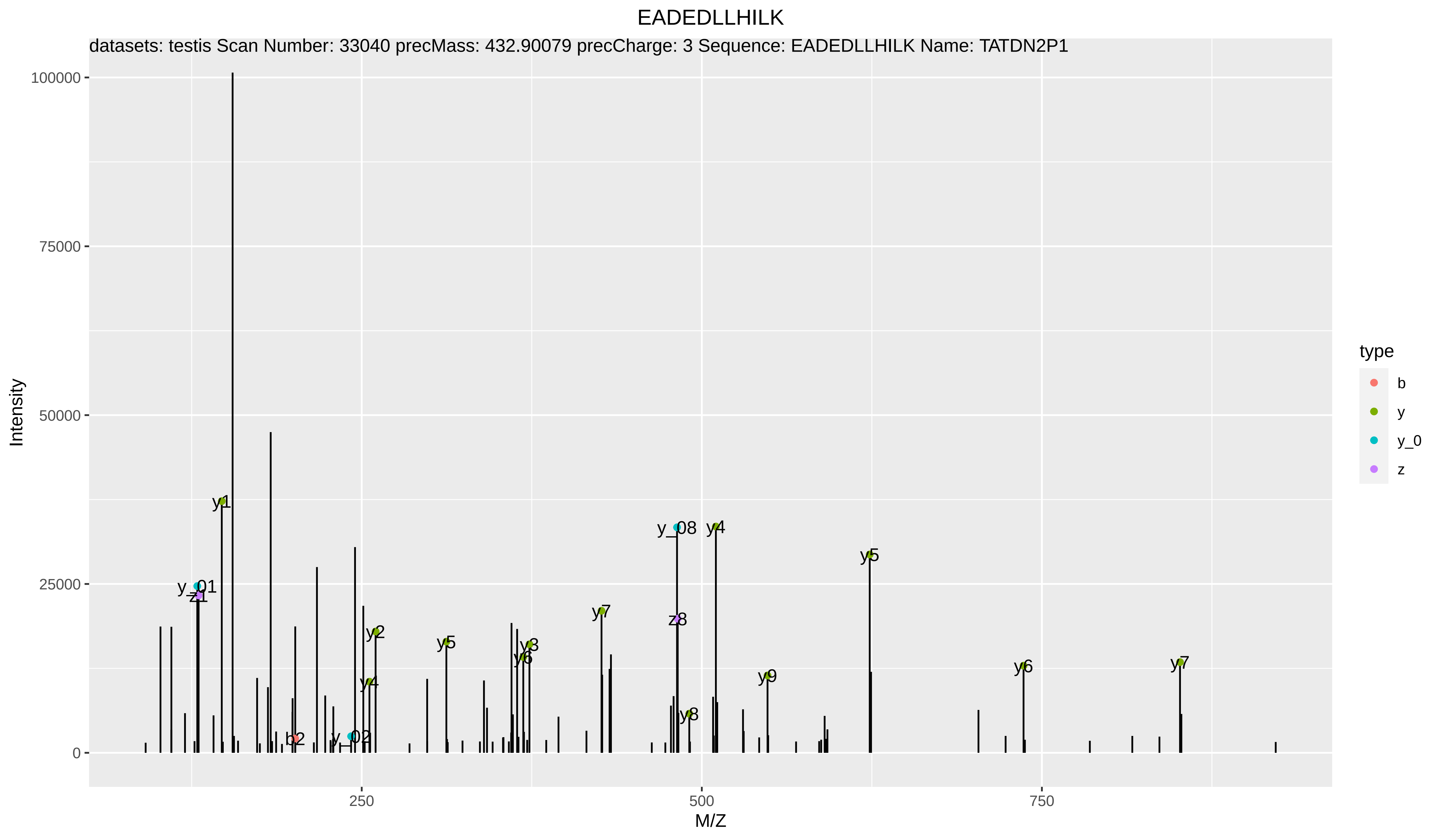

EADEDLLHILK
datasets: testis Scan Number: 33040 precMass: 432.90079 precCharge: 3 Sequence: EADEDLLHILK Name: TATDN2P1
Intensity
M/Z
0
25000
50000
75000
100000
250
500
750
type
b
y
y_0
z
y1
y_01
z1
b2
y_02
y4
y2
y5
y6
y3
y7
y_08
z8
y4
y8
y9
y5
y6
y7

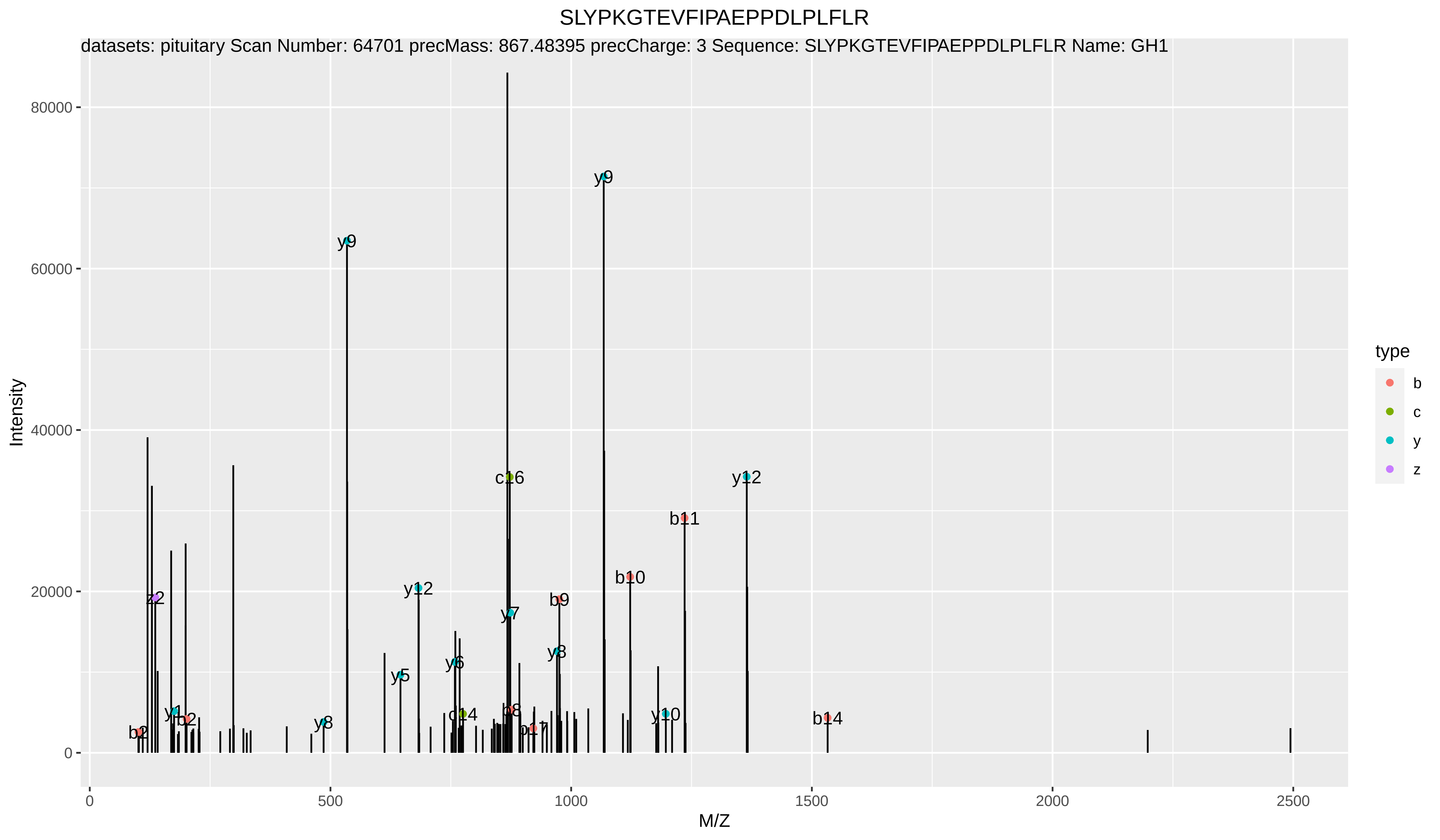

SLYPKGTEVFIPAEPPDLPLFLR
datasets: pituitary Scan Number: 64701 precMass: 867.48395 precCharge: 3 Sequence: SLYPKGTEVFIPAEPPDLPLFLR Name: GH1
Intensity
M/Z
type
b
c
y
z
y9
y9
c16
y12
b11
b10
b9
y7
y12
z2
y8
y6
y5
y1
b2
b2
y8
c14
b8
b17
y10
b14
0
20000
40000
60000
80000
0
500
1000
1500
2000
2500

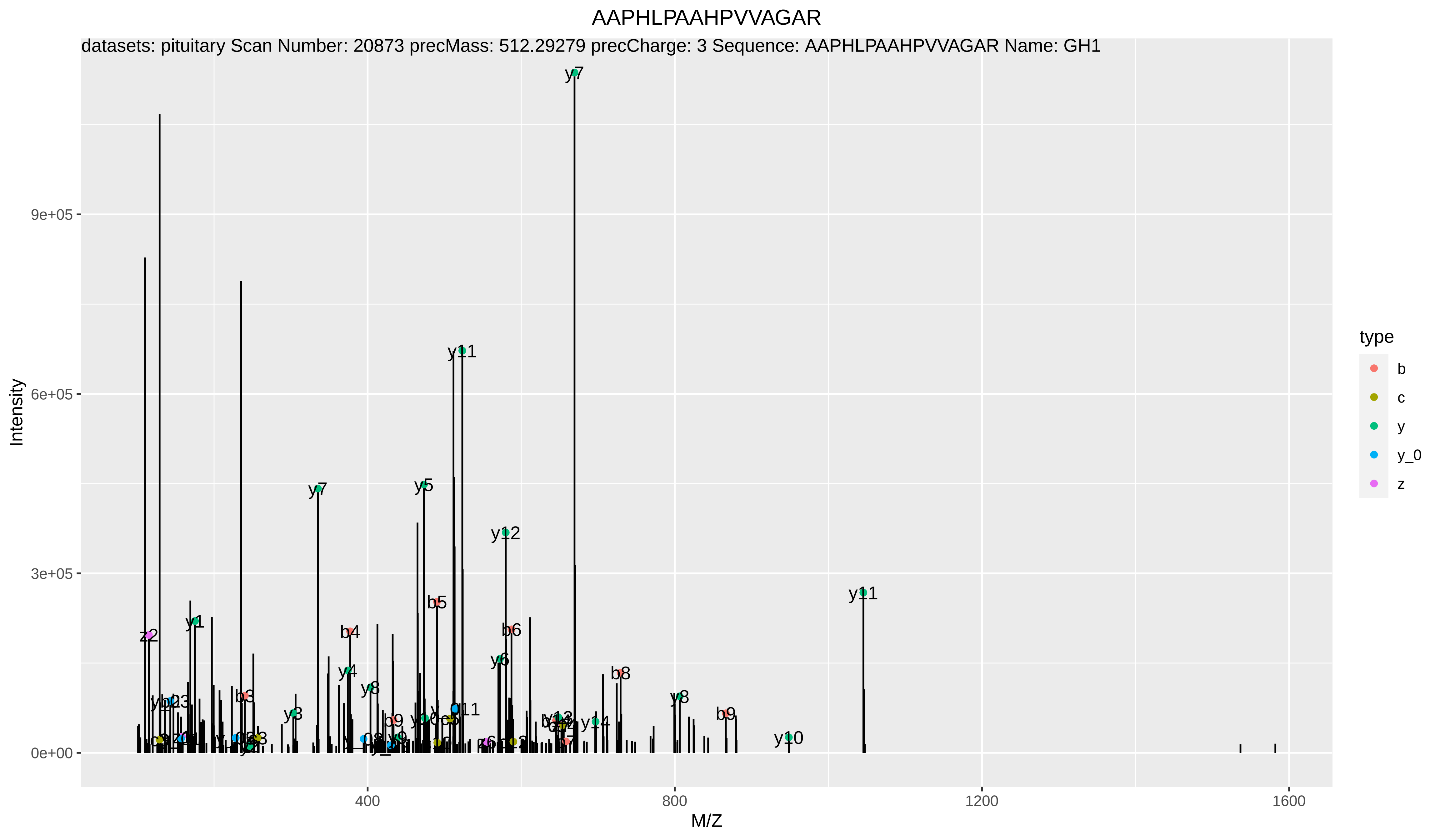
AAPHLPAAHPVVAGAR
datasets: pituitary Scan Number: 20873 precMass: 512.29279 precCharge: 3 Sequence: AAPHLPAAHPVVAGAR Name: GH1
Intensity
M/Z
type
b
c
y
y_0
z
0e+00
3e+05
6e+05
9e+05
400
800
1200
1600
y7
y11
y7
y5
y12
b5
y11
y1
z2
b4
b6
y6
y4
b8
y8
b3
y8
b9
y3
y10

GTEVFIPAEPPDLPLFLR

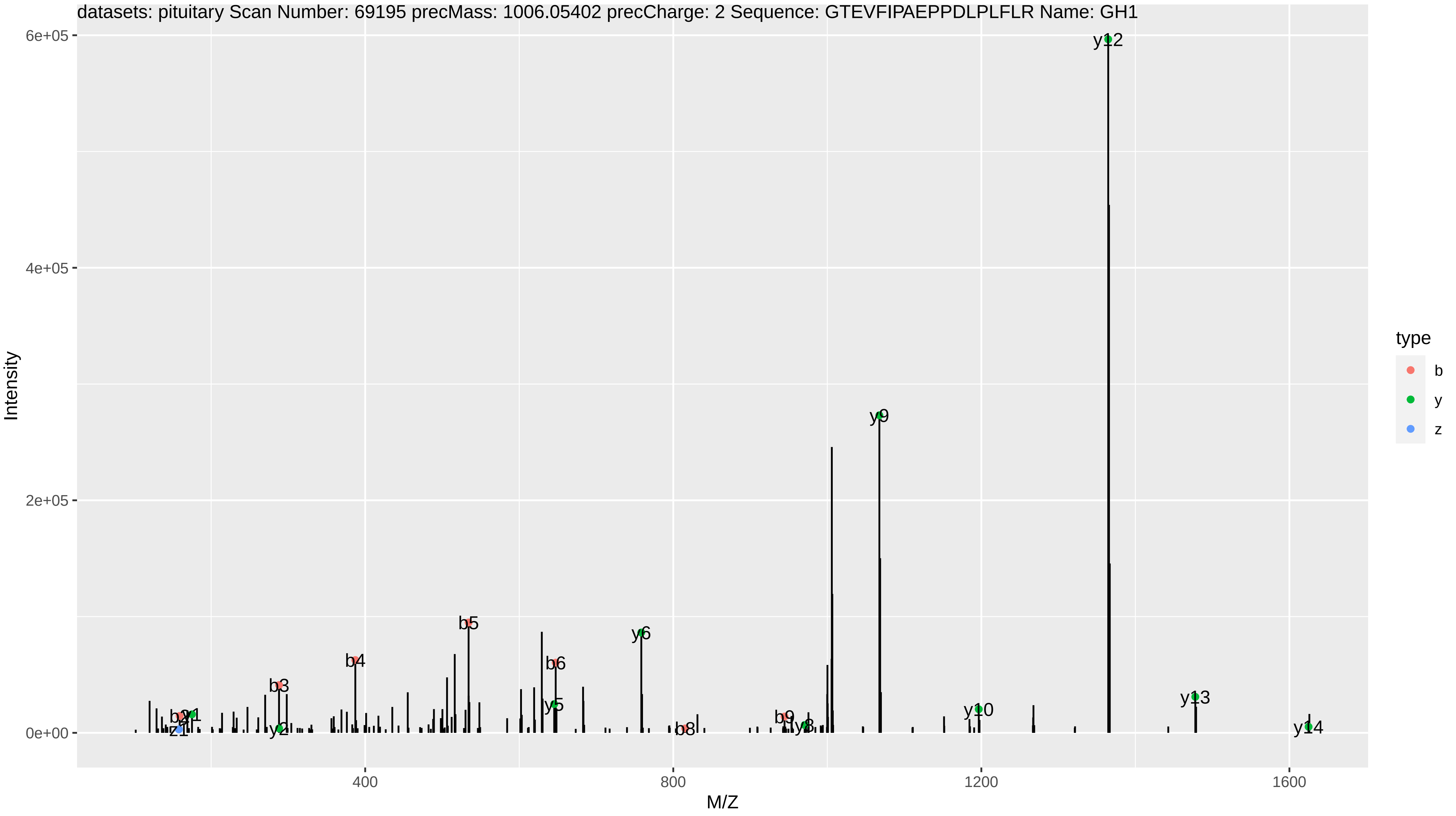

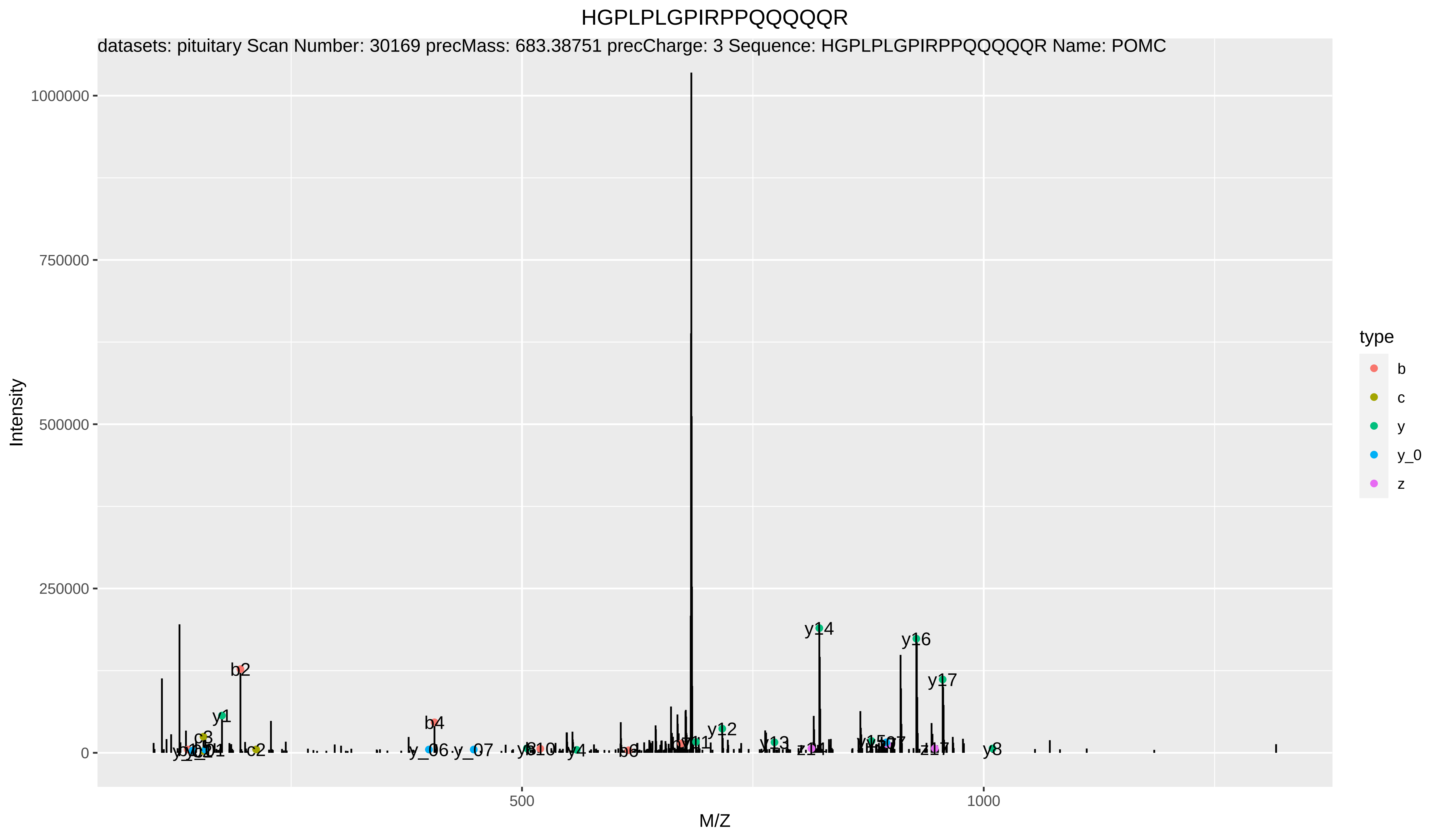
HGPLPLGPIRPPQQQQR
datasets: pituitary Scan Number: 30169 precMass: 683.38751 precCharge: 3 Sequence: HGPLPLGPIRPPQQQQR Name: POMC
Intensity
1000000
750000
500000
250000
0
M/Z
500
1000
type
b
c
y
y_0
z
b2
y1
c3
c2
b4
y_06
y_07
b10
y4
b6
y12
y13
y14
y16
y17
z14
z17
y8

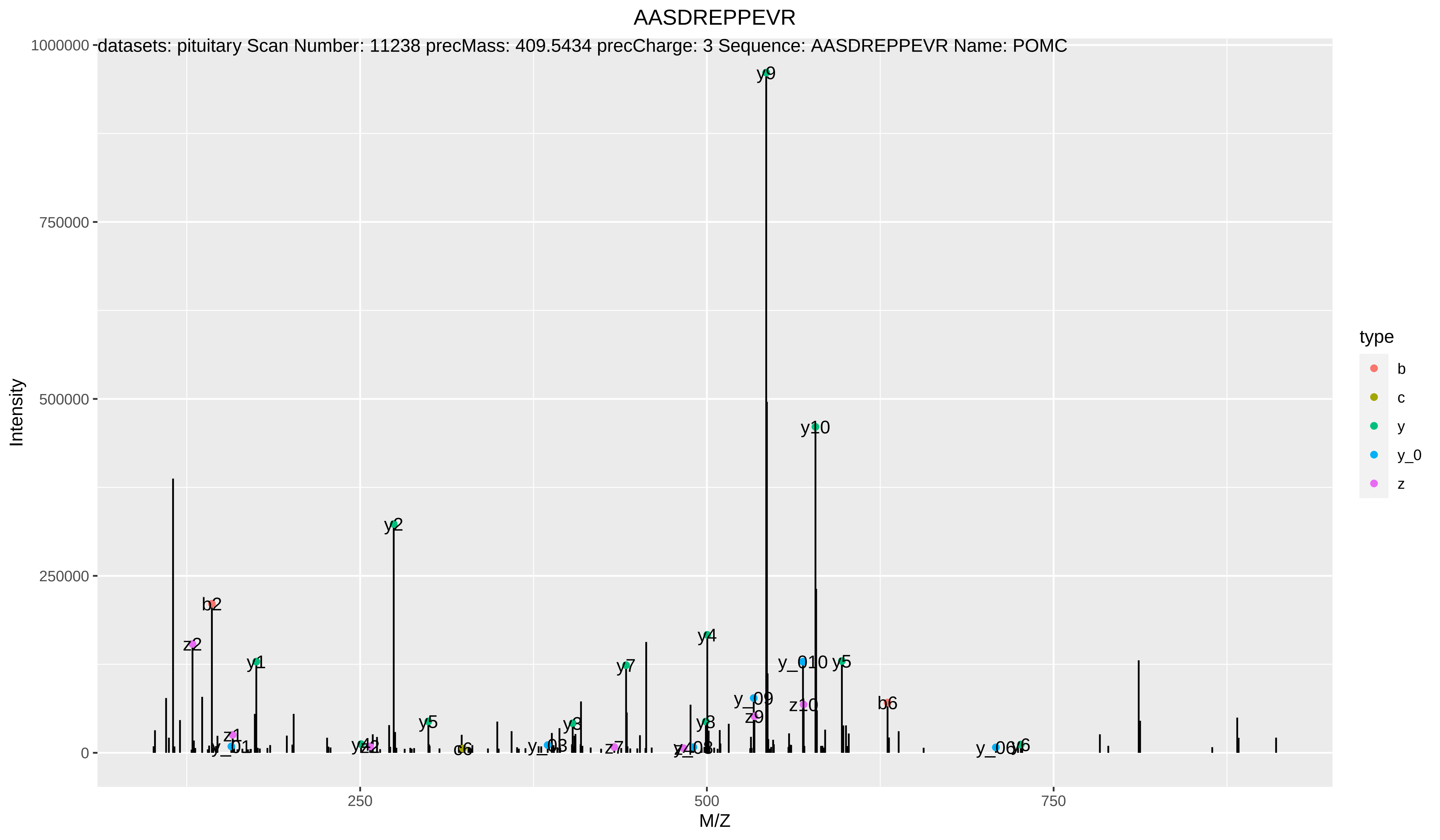
AASDREPPEVR
datasets: pituitary Scan Number: 11238 precMass: 409.5434 precCharge: 3 Sequence: AASDREPPEVR Name: POMC
Intensity
M/Z
type
b
c
y
y_0
z
y9
y10
y2
b2
z2
y1
y4
y7
y_010
y5
y_09
z10
b6
z9
y5
y3
y8
z1
c6
y_03
z7
y_06
y6
0
250000
500000
750000
1000000
250
500
750

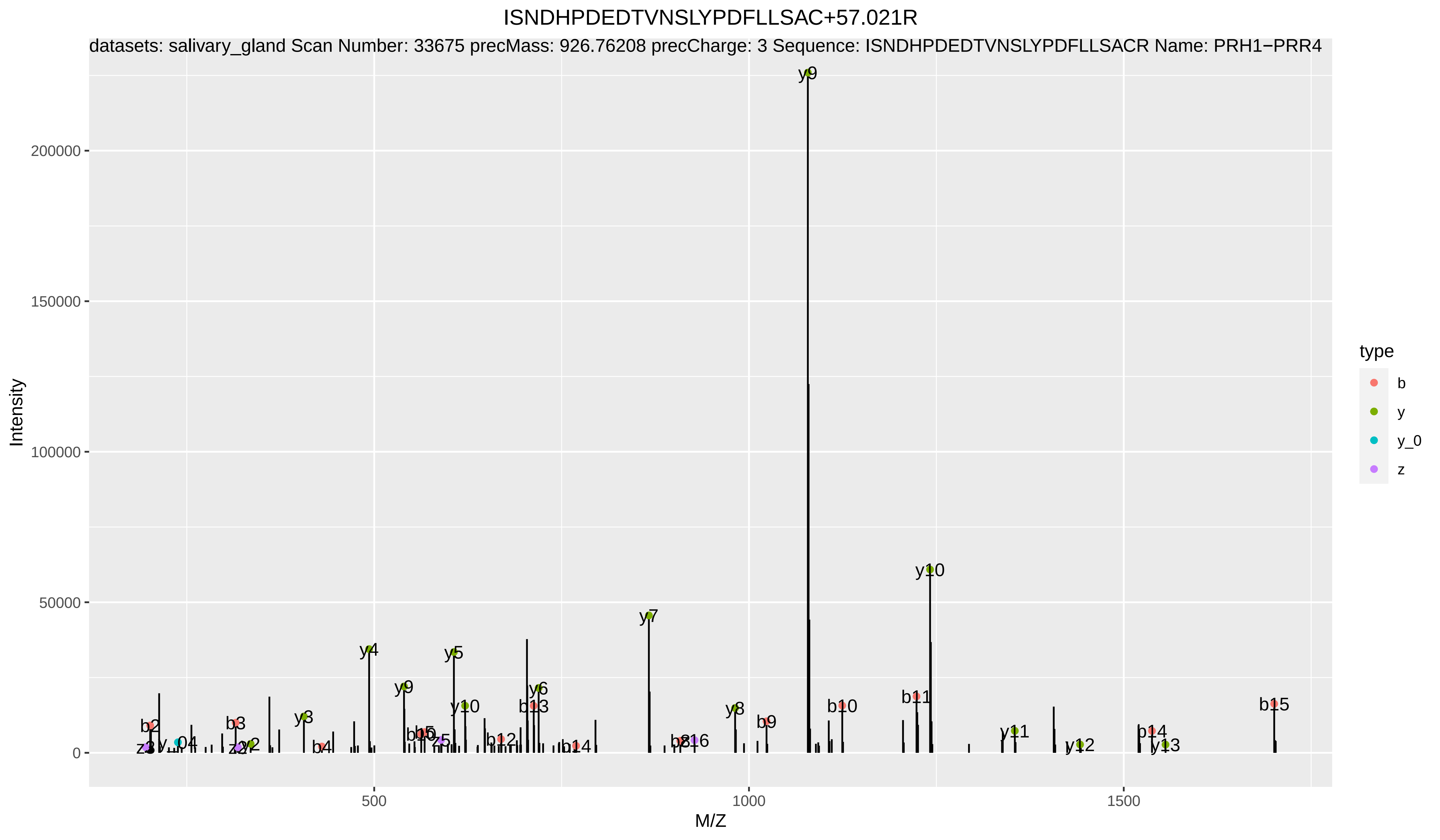
ISNDHPDEDTVNSLYPDFLLSAC+57.021R
datasets: salivary_gland Scan Number: 33675 precMass: 926.76208 precCharge: 3 Sequence: ISNDHPDEDTVNSLYPDFLLSACR Name: PRH1−PRR4
Intensity
M/Z
type
b
y
y_0
z
0
50000
100000
150000
200000
500
1000
1500

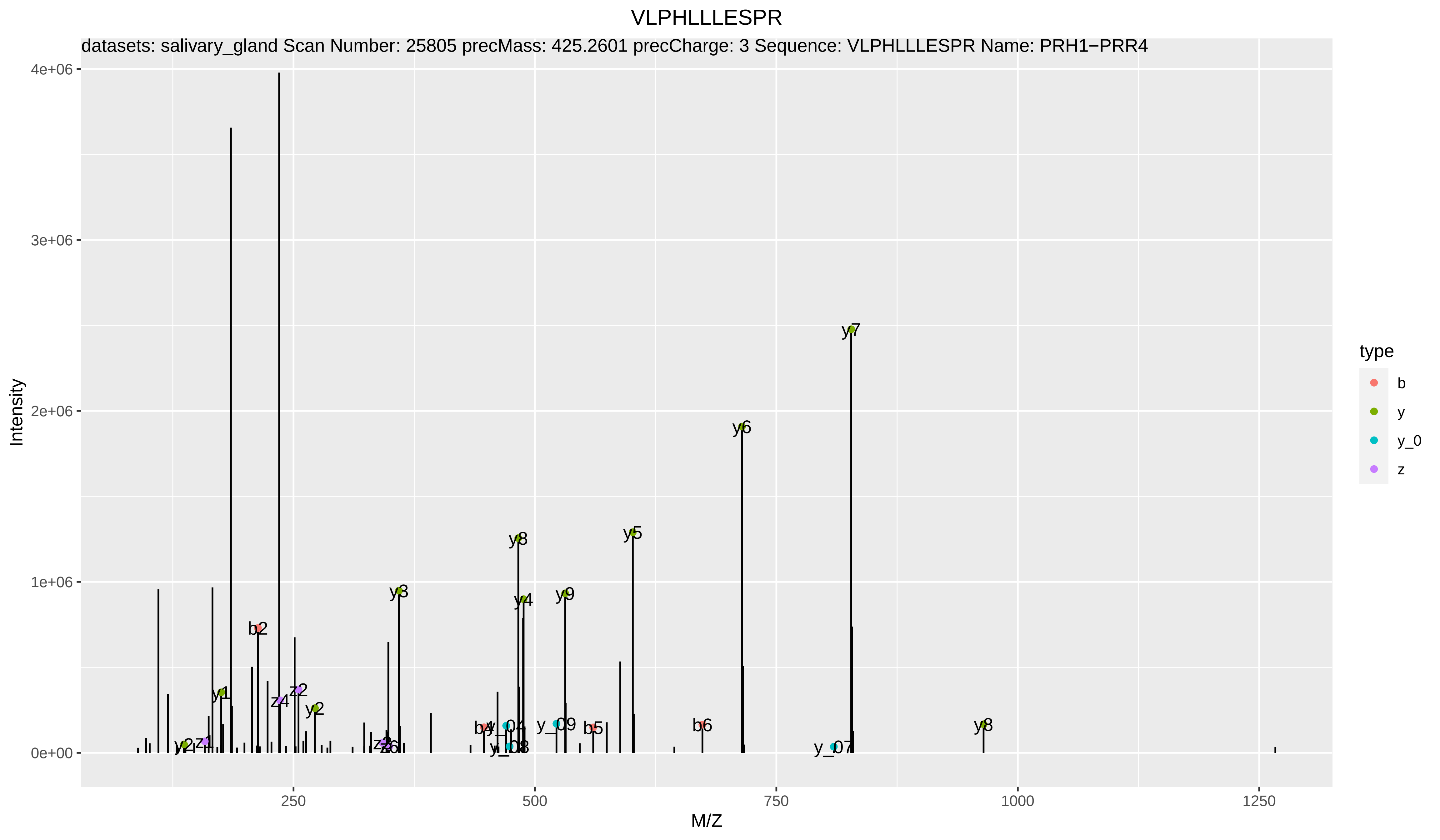
VLPHLLLESPR
datasets: salivary_gland Scan Number: 25805 precMass: 425.2601 precCharge: 3 Sequence: VLPHLLLESPR Name: PRH1−PRR4
Intensity
M/Z
type
b
y
y_0
z
y7
y6
y8
y5
y3
y9
y4
b2
y1
z2
z4
y2
b4
y_04
y_09
b5
b6
y8
y_08
y_07
0e+00
1e+06
2e+06
3e+06
4e+06
250
500
750
1000
1250

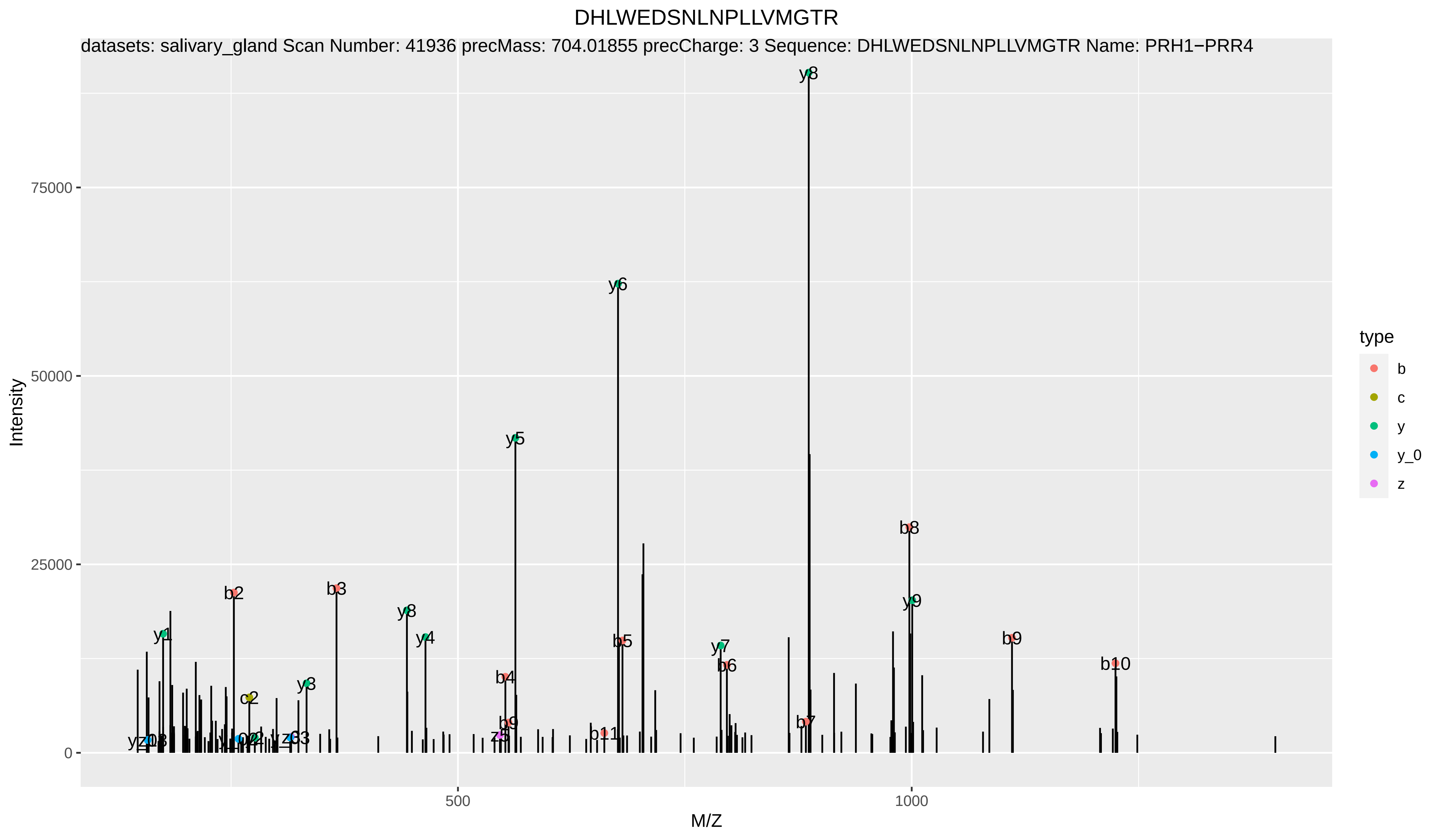

DHLWEDSNLNPLLVMGTR
datasets: salivary_gland Scan Number: 41936 precMass: 704.01855 precCharge: 3 Sequence: DHLWEDSNLNPLLVMGTR Name: PRH1−PRR4
Intensity
M/Z
type
b
c
y
y_0
z

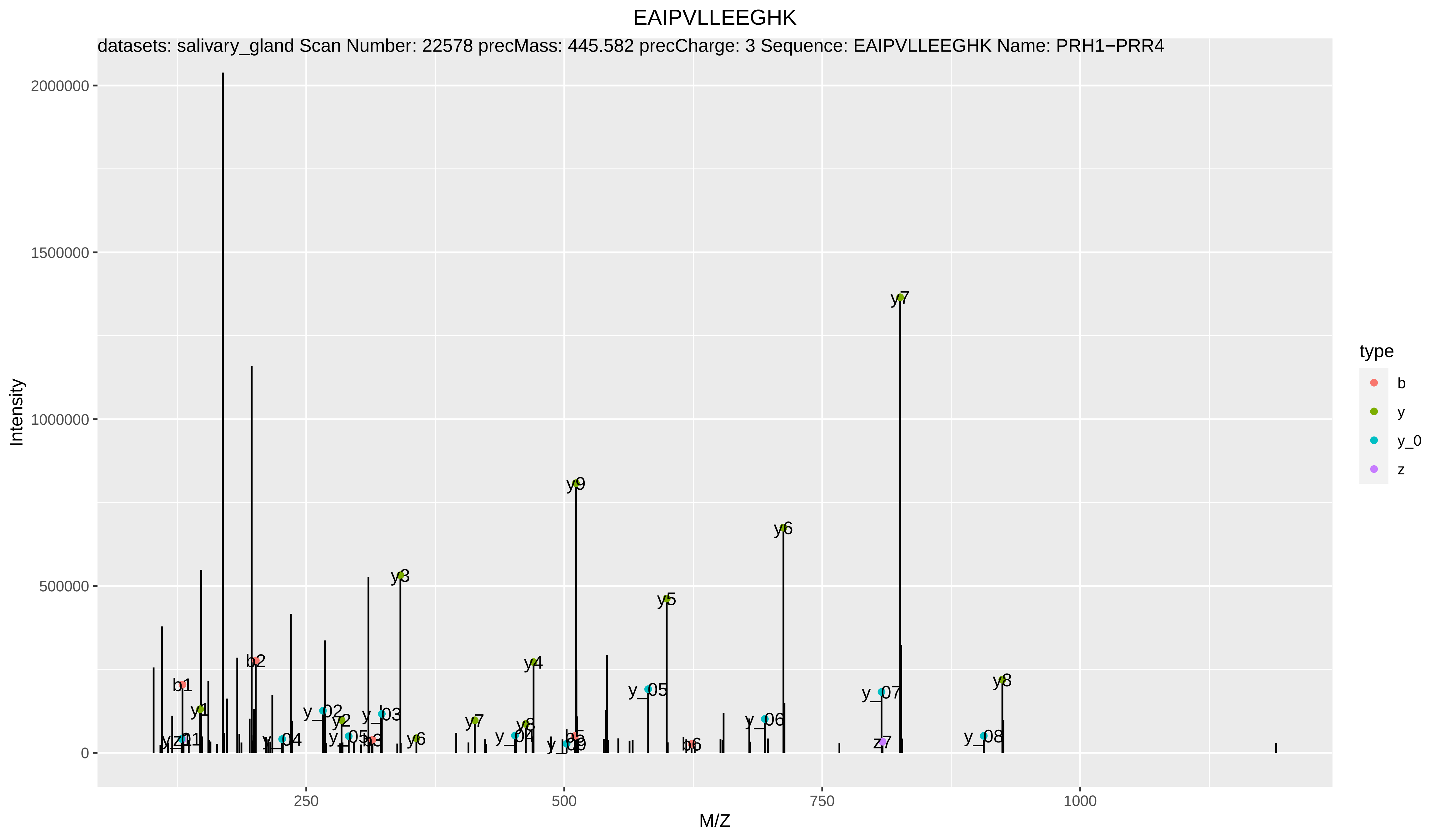
EAIPVLLEEGHK
datasets: salivary_gland Scan Number: 22578 precMass: 445.582 precCharge: 3 Sequence: EAIPVLLEEGHK Name: PRH1−PRR4
Intensity
M/Z
type
b
y
y_0
z

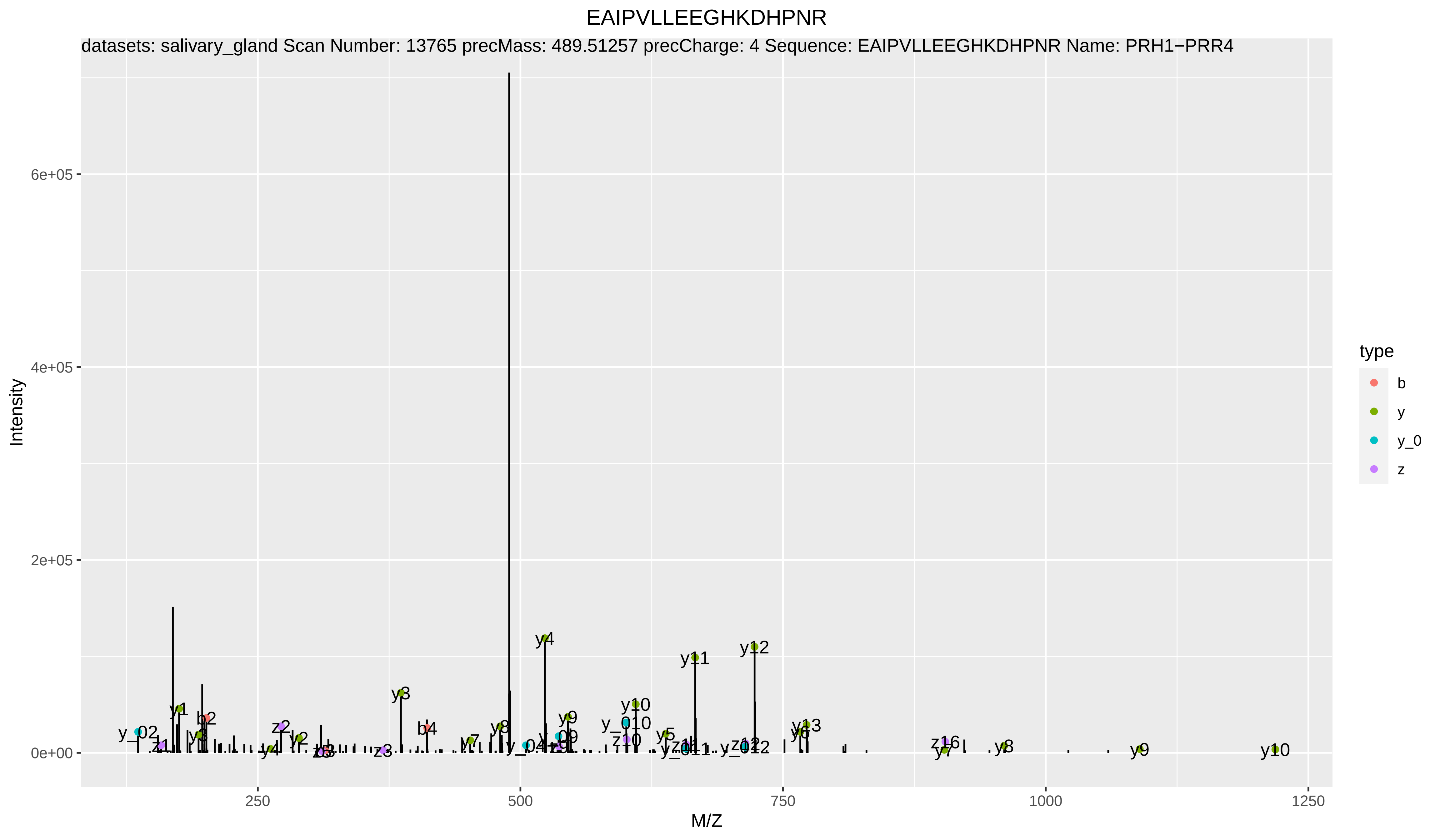
EAIPVLLEEGHKDHPNR
datasets: salivary_gland Scan Number: 13765 precMass: 489.51257 precCharge: 4 Sequence: EAIPVLLEEGHKDHPNR Name: PRH1−PRR4
Intensity
6e+05
4e+05
2e+05
0e+00
M/Z
250
500
750
1000
1250
type
b
y
y_0
z
y4
y11
y12
y3
y10
y_010
y9
y1
b2
b4
y13
y8
y7
y5
y6
z2
z3
z10
z16
y_02
y_011
y_012
y10

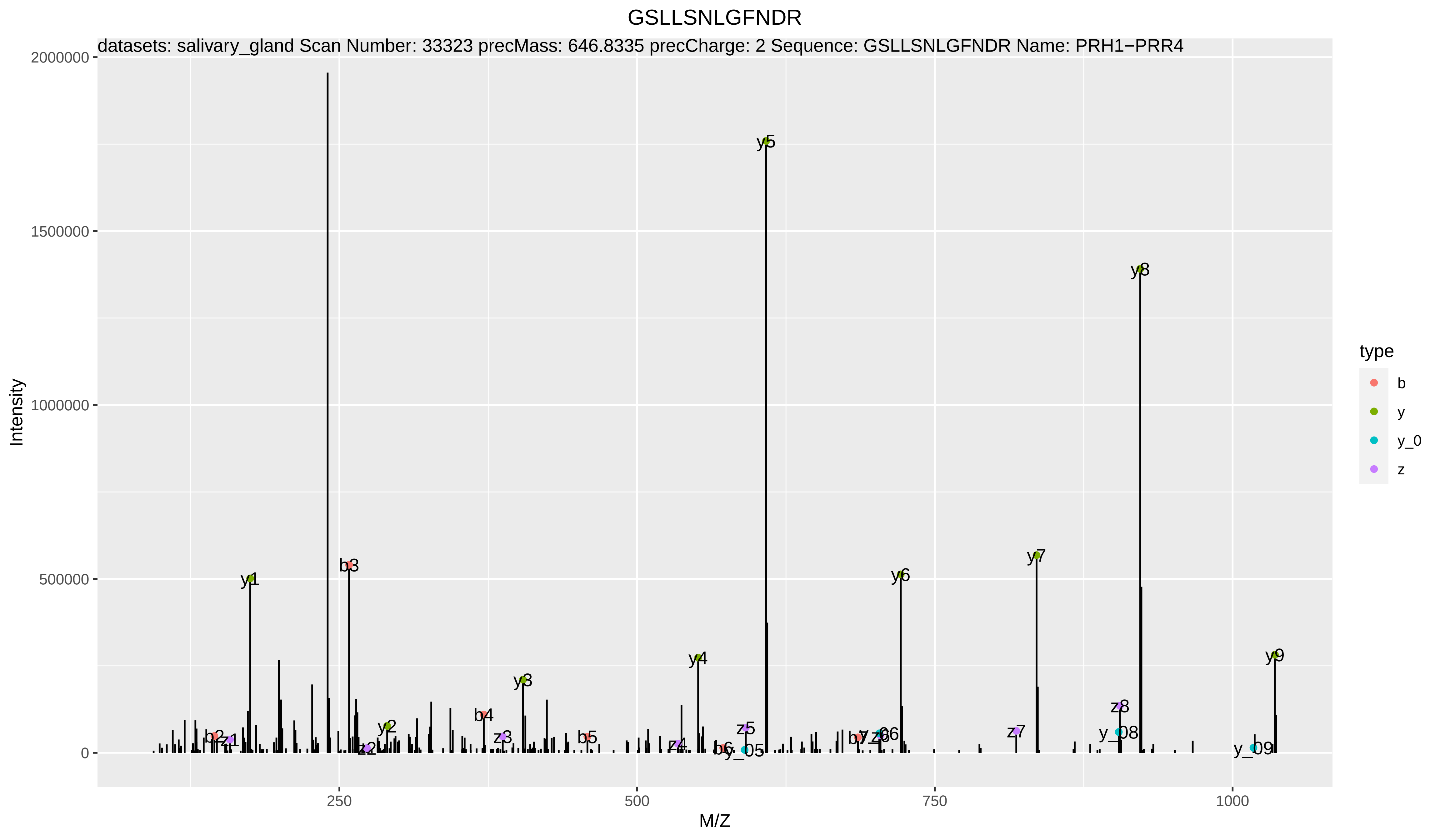
GSLLSNLGFNDR
datasets: salivary_gland Scan Number: 33323 precMass: 646.8335 precCharge: 2 Sequence: GSLLSNLGFNDR Name: PRH1−PRR4
Intensity
2000000
1500000
1000000
500000
0
M/Z
250
500
750
1000
y5
y8
y7
y6
b3
y1
y9
y4
y3
b4
z8
y2
b2
z1
z2
z3
b5
z4
b6
y_05
z5
b7
y_06
z6
z7
y_08
y_09
type
b
y
y_0
z

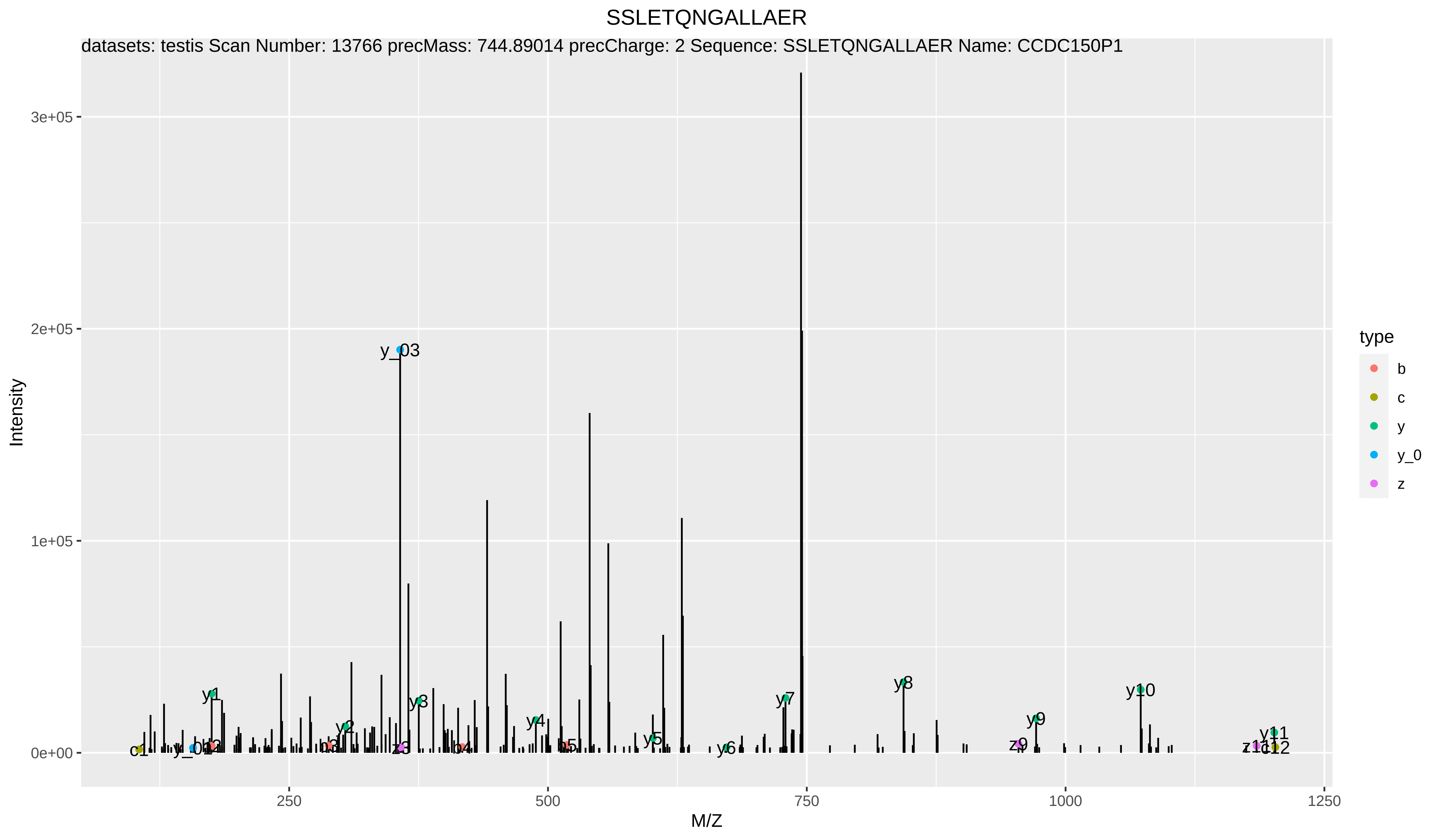

SSLETQNGALLAER
datasets: testis Scan Number: 13766 precMass: 744.89014 precCharge: 2 Sequence: SSLETQNGALLAER Name: CCDC150P1
Intensity
M/Z
type
b
c
y
y_0
z
y_03
y1
y2
y3
y4
y5
y6
y7
y8
y9
y10
y11
z9
z3
b4
c1
c12

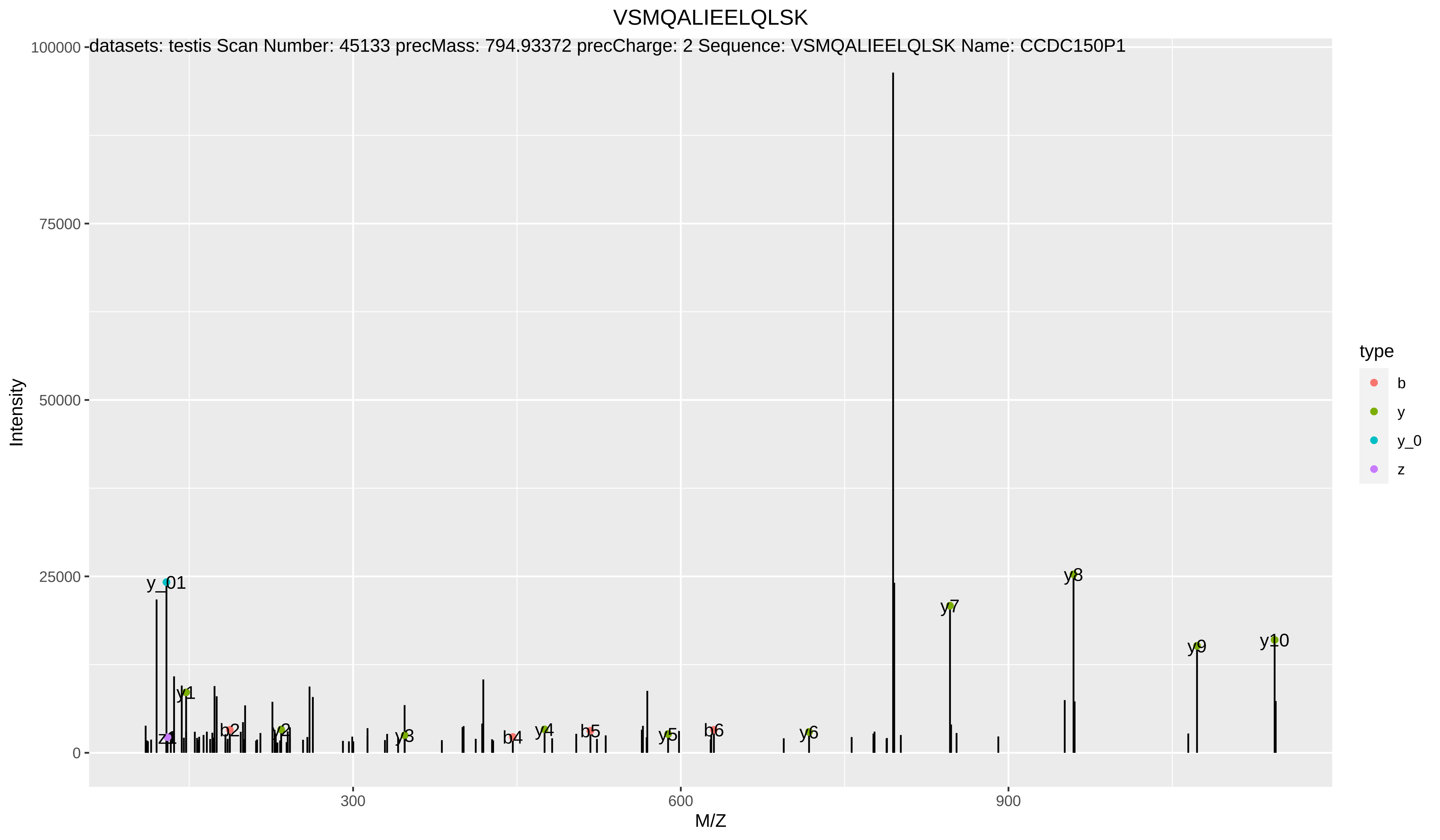
VSMQALIEELQLSK
datasets: testis Scan Number: 45133 precMass: 794.93372 precCharge: 2 Sequence: VSMQALIEELQLSK Name: CCDC150P1
Intensity
M/Z
type
b
y
y_0
z
y_01
z1
y1
b2
y2
y3
b4
y4
b5
y5
b6
y6
y7
y8
y9
y10

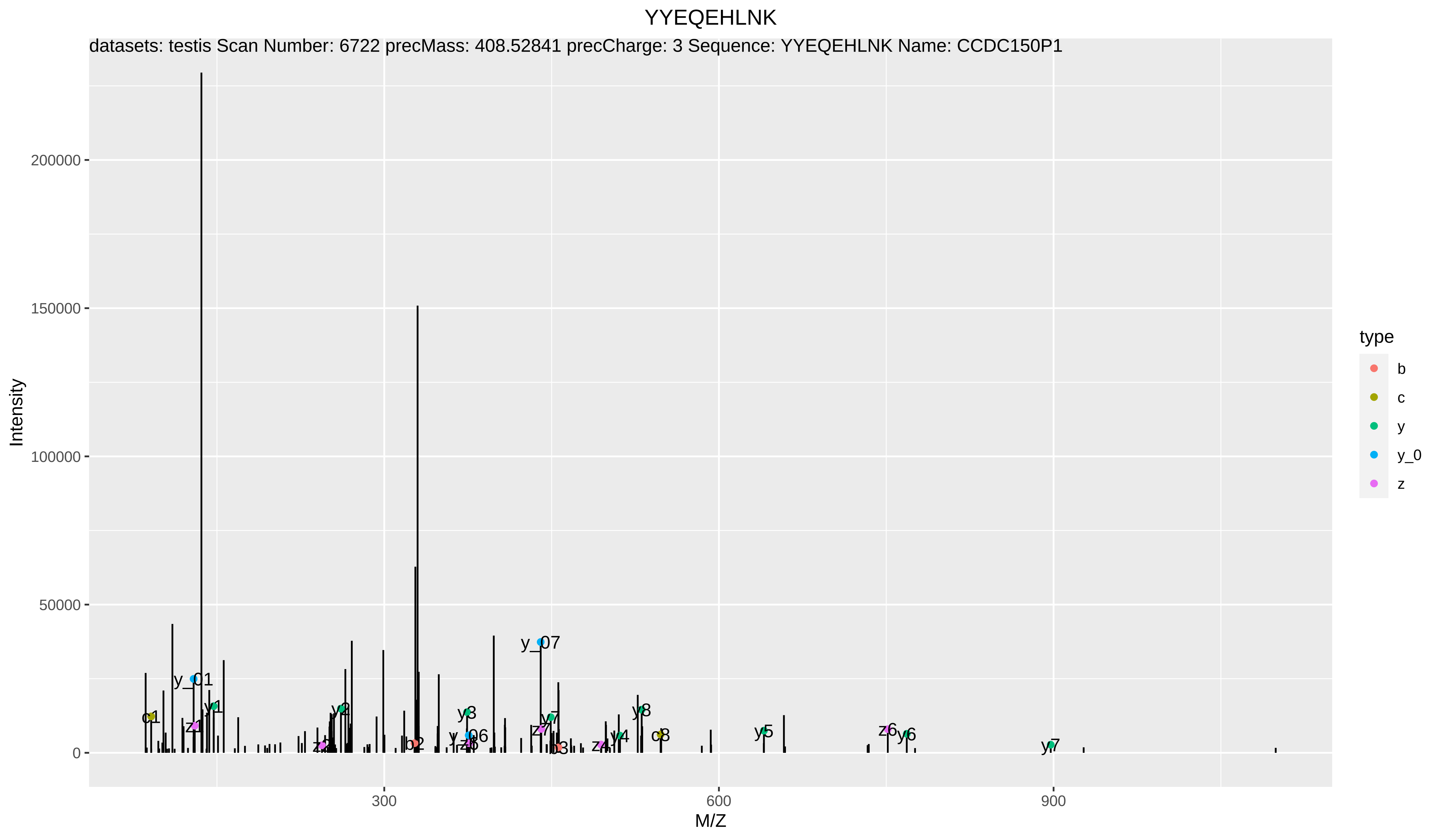
YYEQEHLNK
datasets: testis Scan Number: 6722 precMass: 408.52841 precCharge: 3 Sequence: YYEQEHLNK Name: CCDC150P1
Intensity
M/Z
type
b
c
y
y_0
z
0
50000
100000
150000
200000
300
600
900

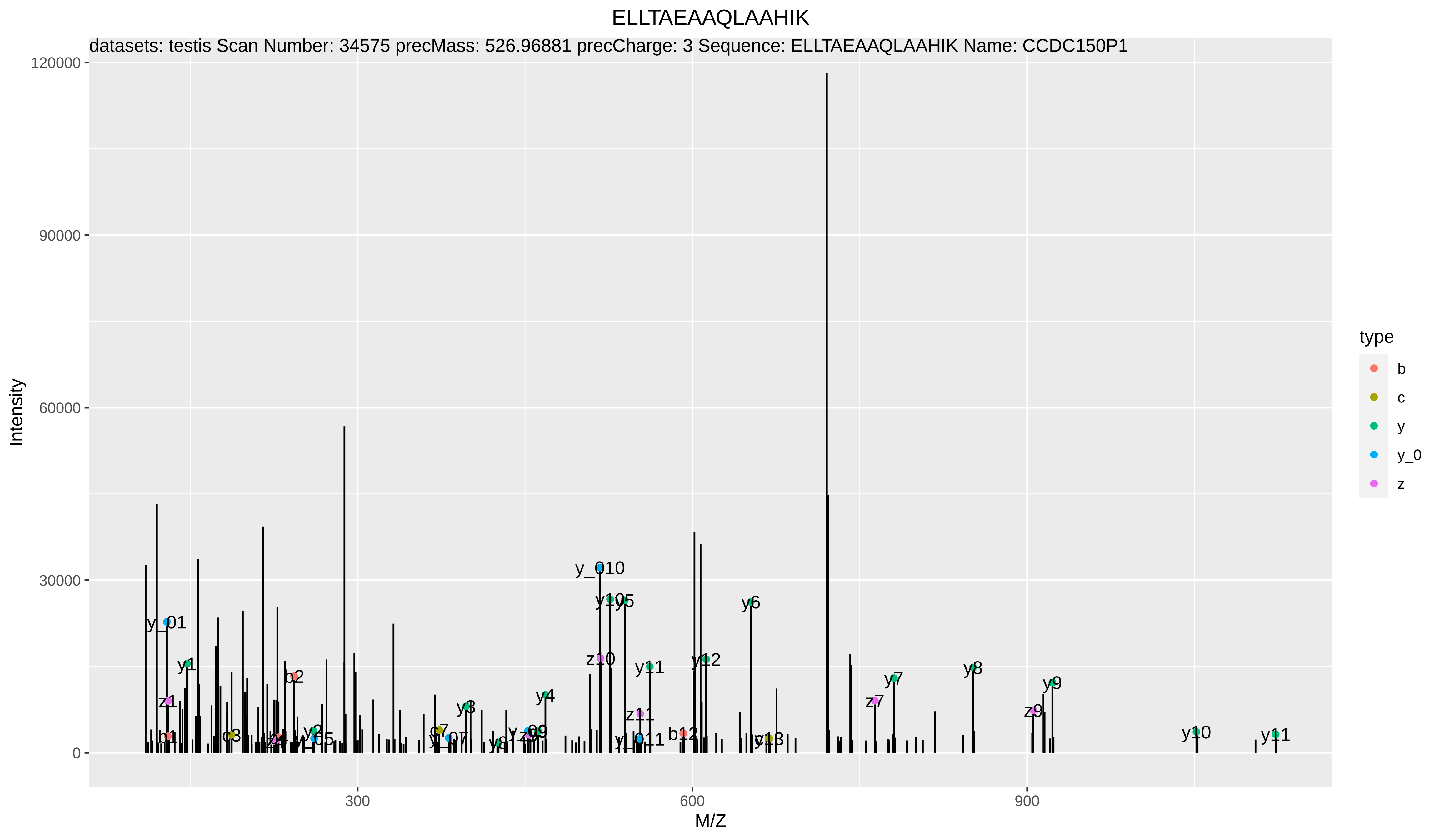
ELLTAEAAQLAAHIK
datasets: testis Scan Number: 34575 precMass: 526.96881 precCharge: 3 Sequence: ELLTAEAAQLAAHIK Name: CCDC150P1
Intensity
120000
90000
60000
30000
0
M/Z
300
600
900
type
b
c
y
y_0
z
y_01
y1
z1
b2
y_010
z10
y11
z11
y12
y6
y7
z7
y8
y9
z9
y10
y11
b12
y4
y3

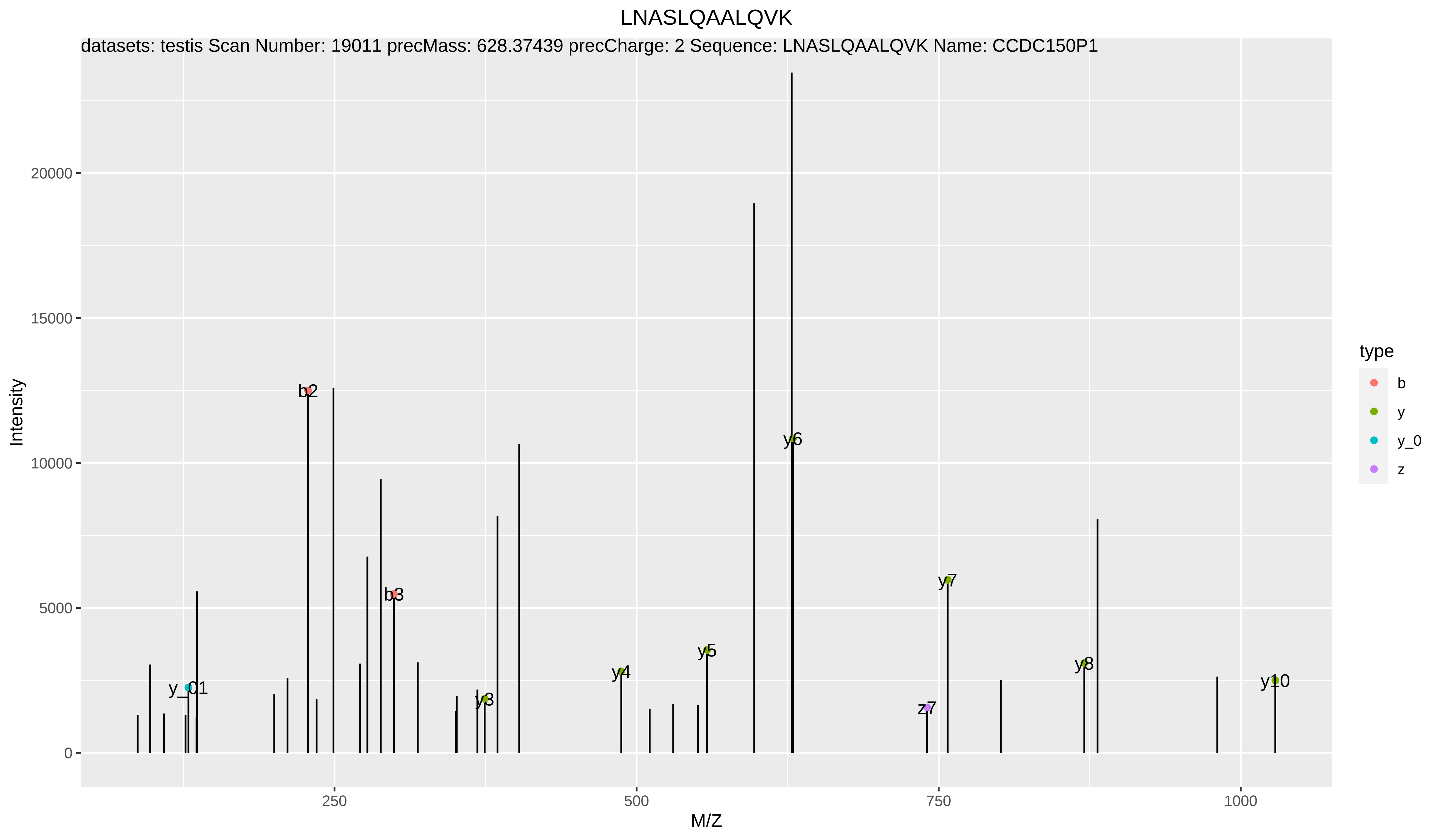

LNASLQAALQVK
datasets: testis Scan Number: 19011 precMass: 628.37439 precCharge: 2 Sequence: LNASLQAALQVK Name: CCDC150P1
Intensity
M/Z
0
5000
10000
15000
20000
250
500
750
1000
b2
b3
y_01
y3
y4
y5
y6
z7
y7
y8
y10
type
b
y
y_0
z

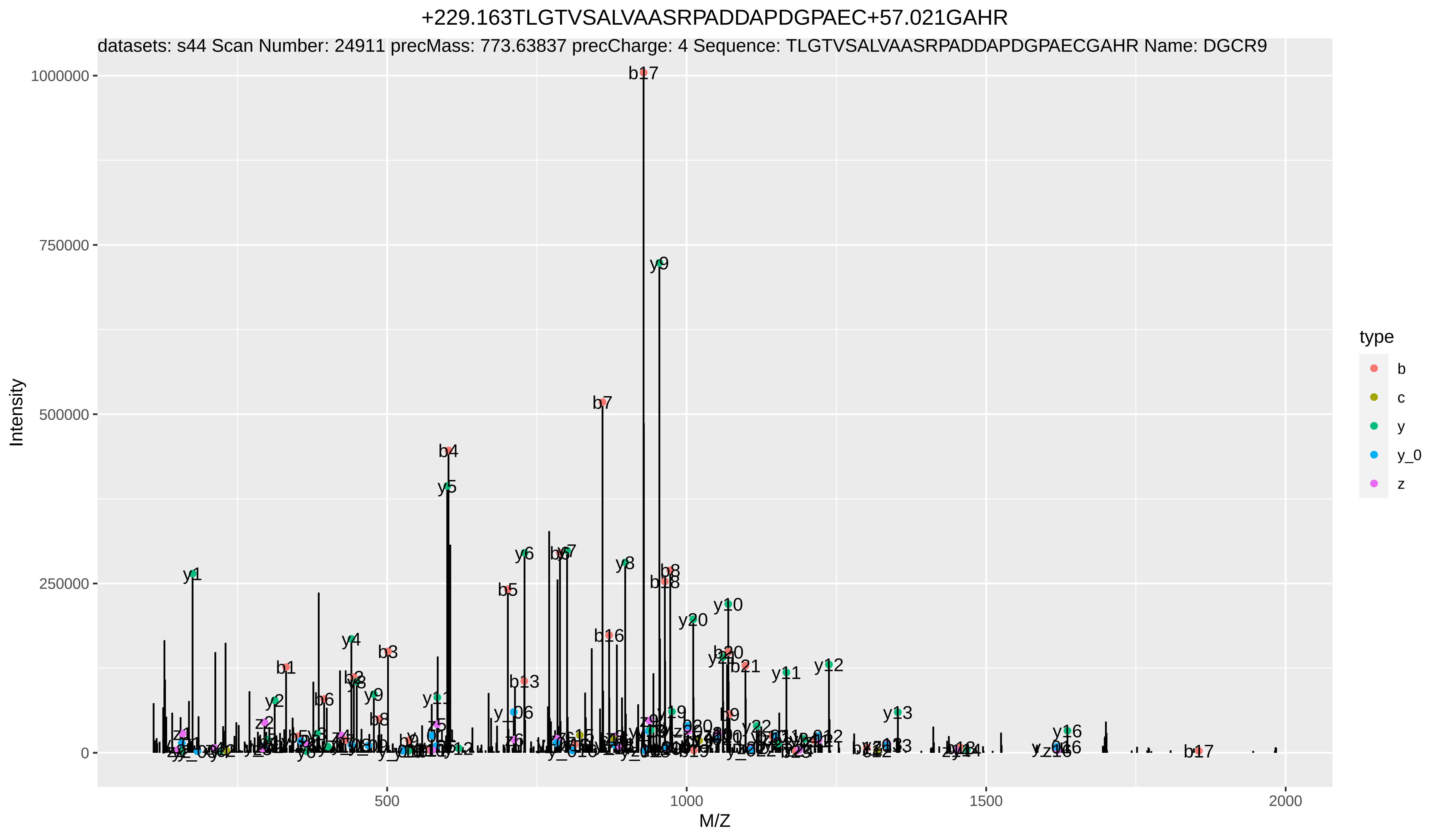
+229.163TLGTVSALVAASRPADDAPDGPAEC+57.021GAHR
datasets: s44 Scan Number: 24911 precMass: 773.63837 precCharge: 4 Sequence: TLGTVSALVAASRPADDAPDGPAECGAHR Name: DGCR9
Intensity
M/Z
type
b
c
y
y_0
z

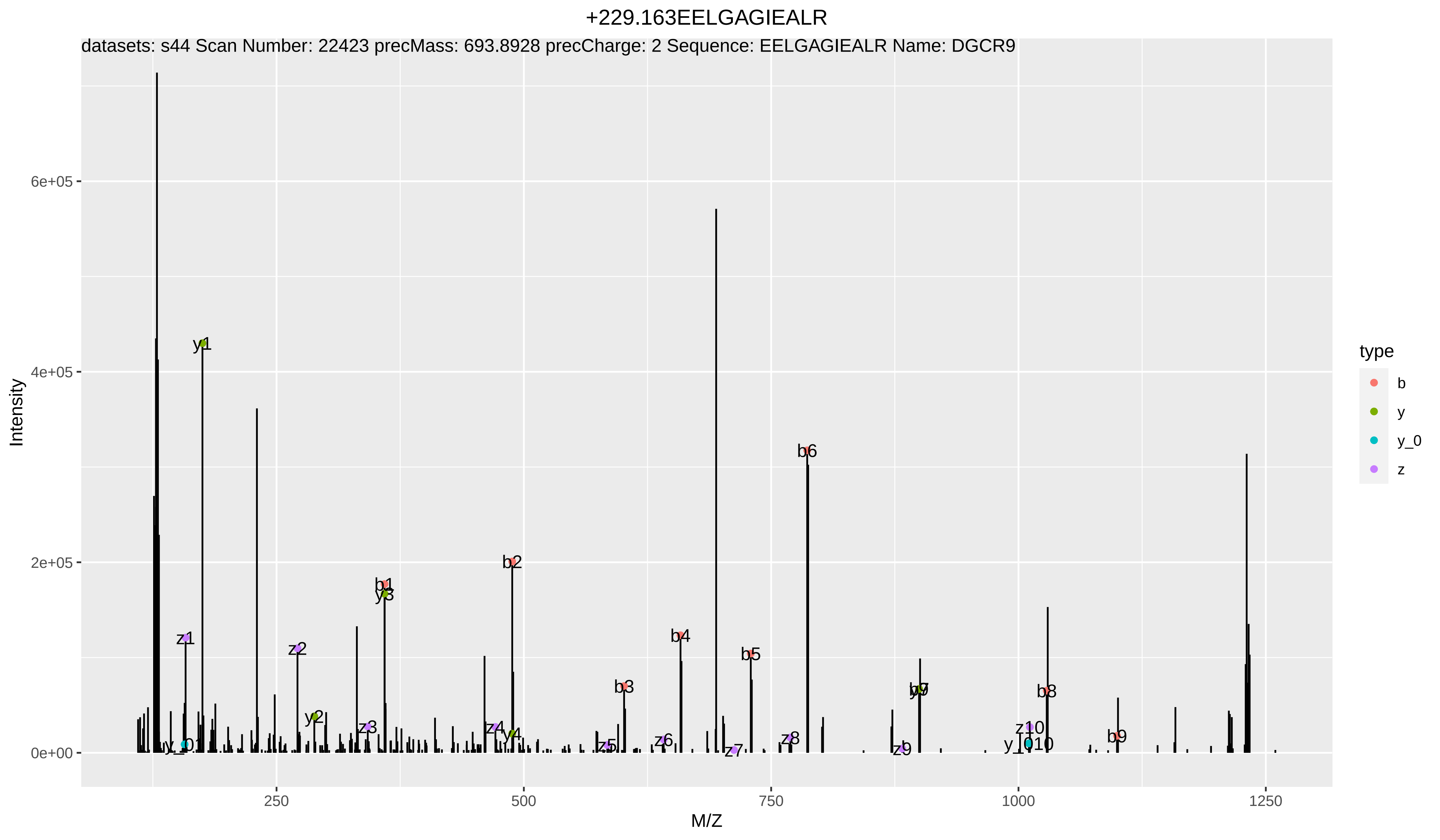
+229.163EELGAGIEALR
datasets: s44 Scan Number: 22423 precMass: 693.8928 precCharge: 2 Sequence: EELGAGIEALR Name: DGCR9
Intensity
M/Z
type
b
y
y_0
z
0e+00
2e+05
4e+05
6e+05
250
500
750
1000
1250
y1
z1
z2
y2
z3
b1
y3
z4
y4
b2
z5
b3
z6
b4
z7
b5
b6
z8
z9
b7
y_010
z10
b8
b9

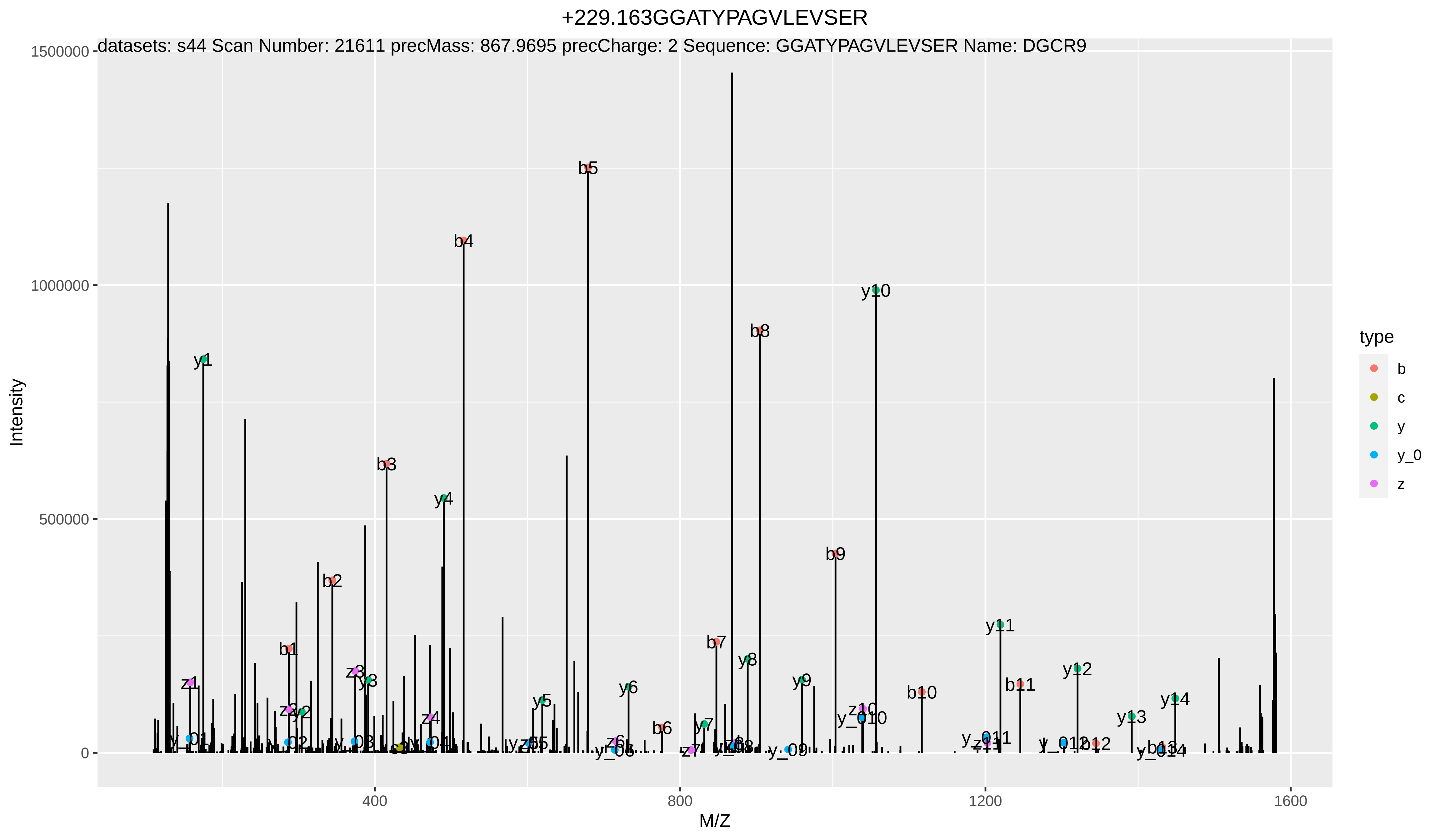
+229.163GGATYPAGVLEVSER
datasets: s44 Scan Number: 21611 precMass: 867.9695 precCharge: 2 Sequence: GGATYPAGVLEVSER Name: DGCR9
Intensity
1500000
1000000
500000
0
M/Z
400
800
1200
1600
type
b
c
y
y_0
z
b1
b2
b3
b4
b5
b6
b7
b8
b9
b10
b11
b12
b13
y1
y2
y3
y4
y5
y6
y7
y8
y9
y10
y11
y12
y13
y14
z1
z2
z3
z4
z6
z7
z10
z11
y_06
y_09
y_010
y_011
y_012
y_014

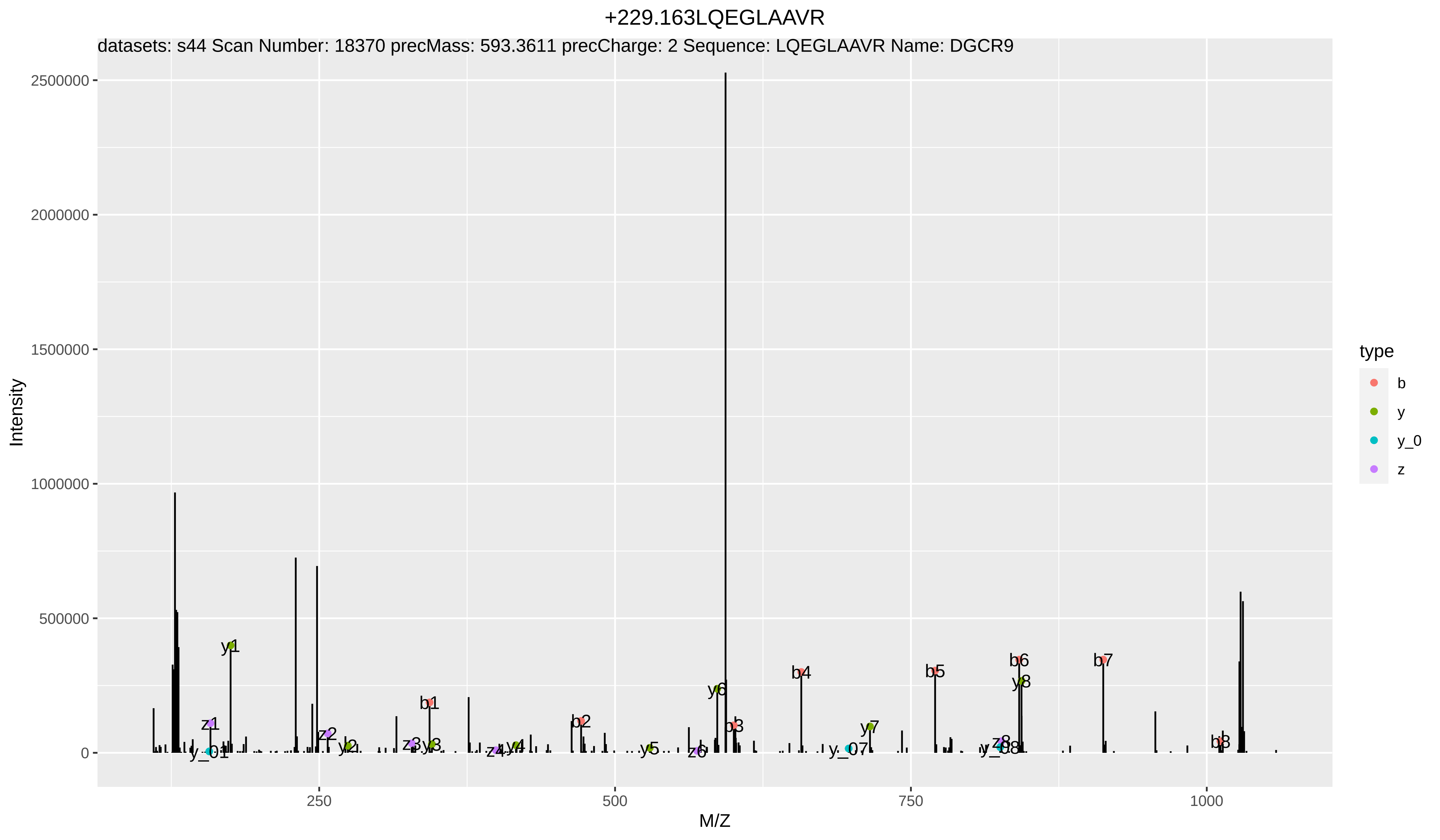

+229.163LQEGLAAVR
datasets: s44 Scan Number: 18370 precMass: 593.3611 precCharge: 2 Sequence: LQEGLAAVR Name: DGCR9
Intensity
2500000
2000000
1500000
1000000
500000
0
250
500
750
1000
M/Z
type
b
y
y_0
z
y1
z1
y_01
z2
y2
b1
z3
y3
z4
y4
b2
y5
z6
y6
b3
b4
y_07
y7
b5
b6
y8
z8
y_08
b7
b8

+229.163LQEGLAAVREELGAGIEALR

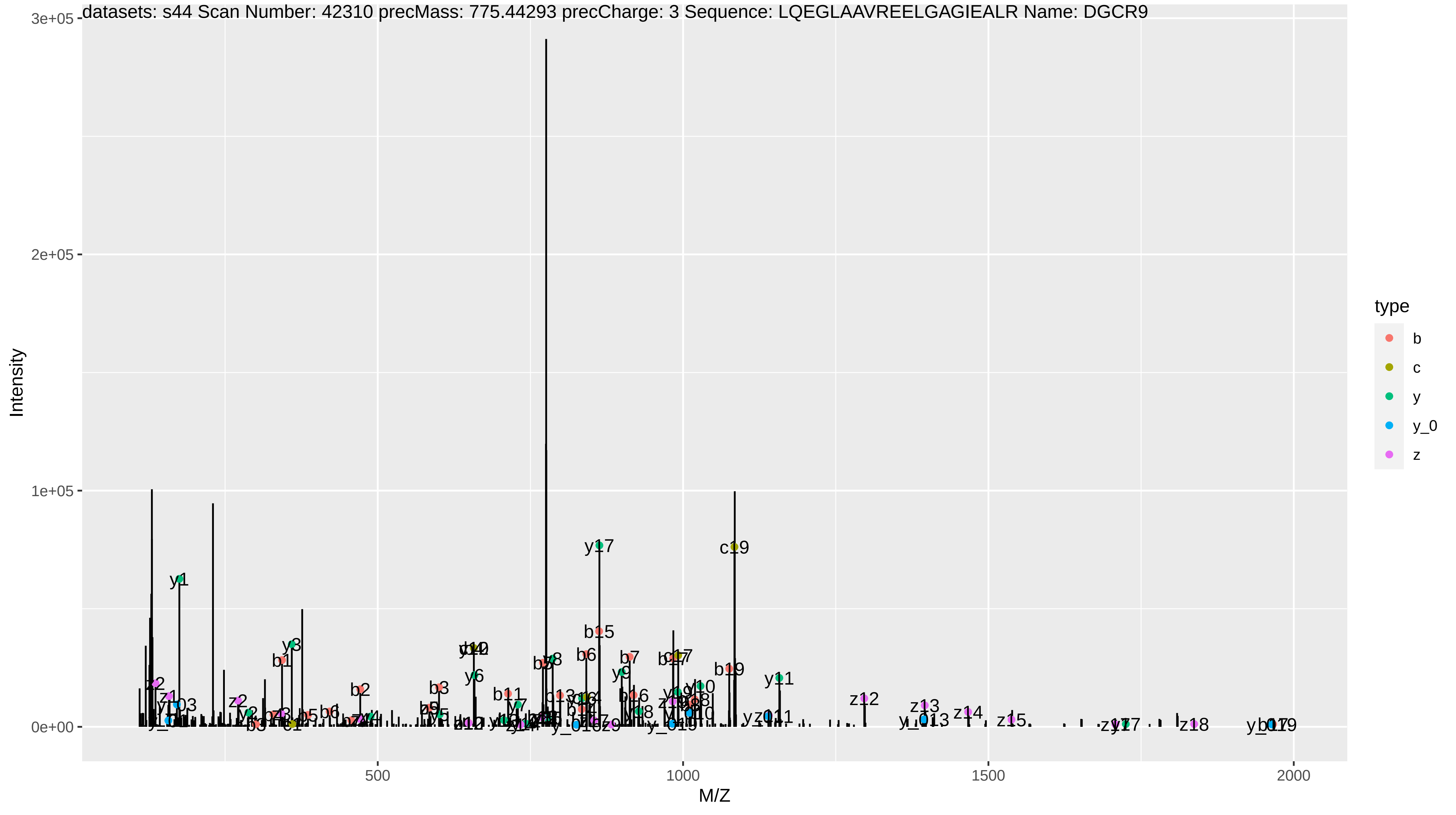

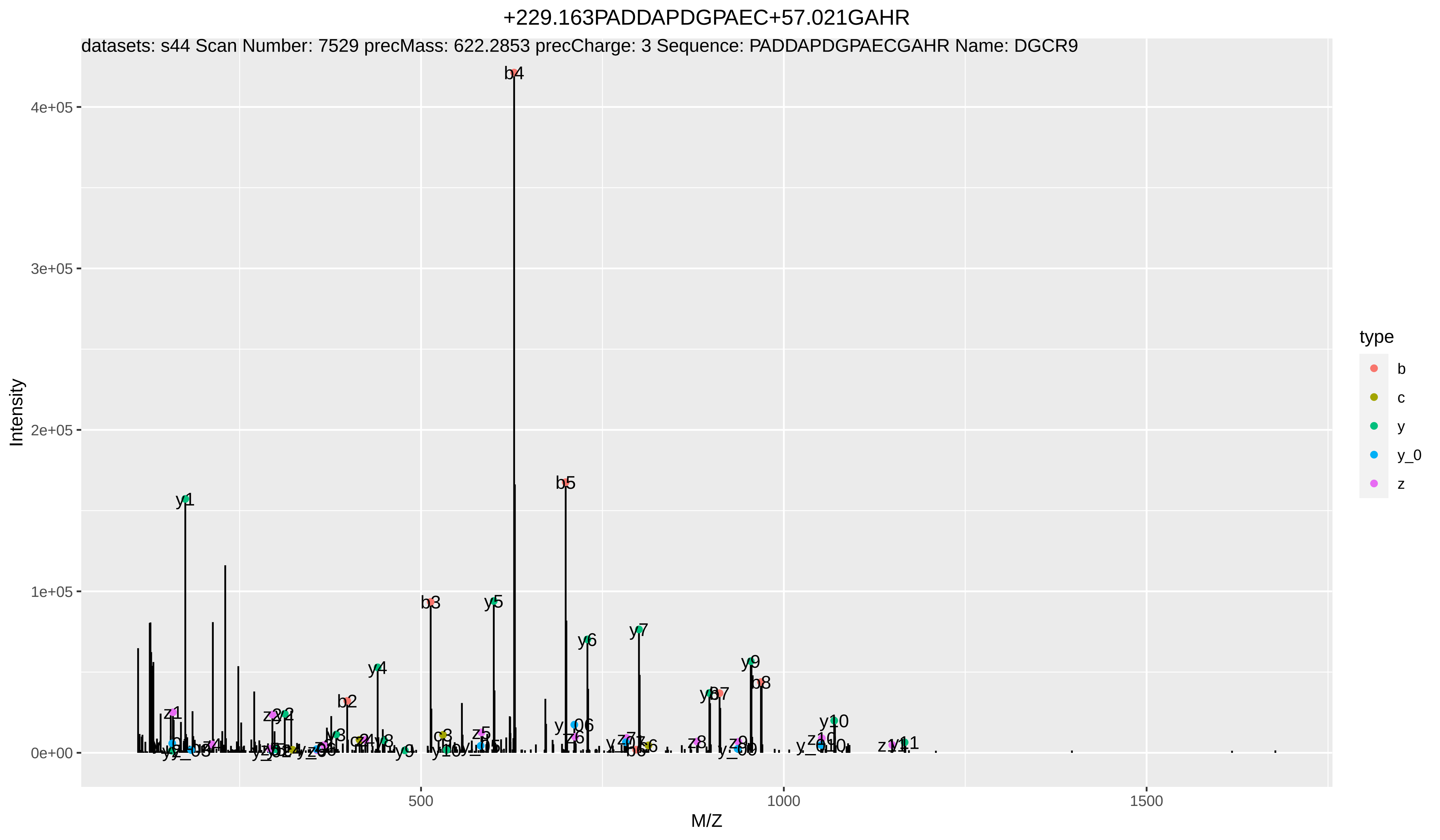
+229.163PADDAPDGPAEC+57.021GAHR
datasets: s44 Scan Number: 7529 precMass: 622.2853 precCharge: 3 Sequence: PADDAPDGPAECGAHR Name: DGCR9
Intensity
M/Z
0e+00
1e+05
2e+05
3e+05
4e+05
500
1000
1500
type
b
c
y
y_0
z
b4
b5
y1
b3
y5
y6
y7
y4
y9
b8
b7
b2
y10
y_06
z1
z2
y2
z5
c3
y11
z8
z10

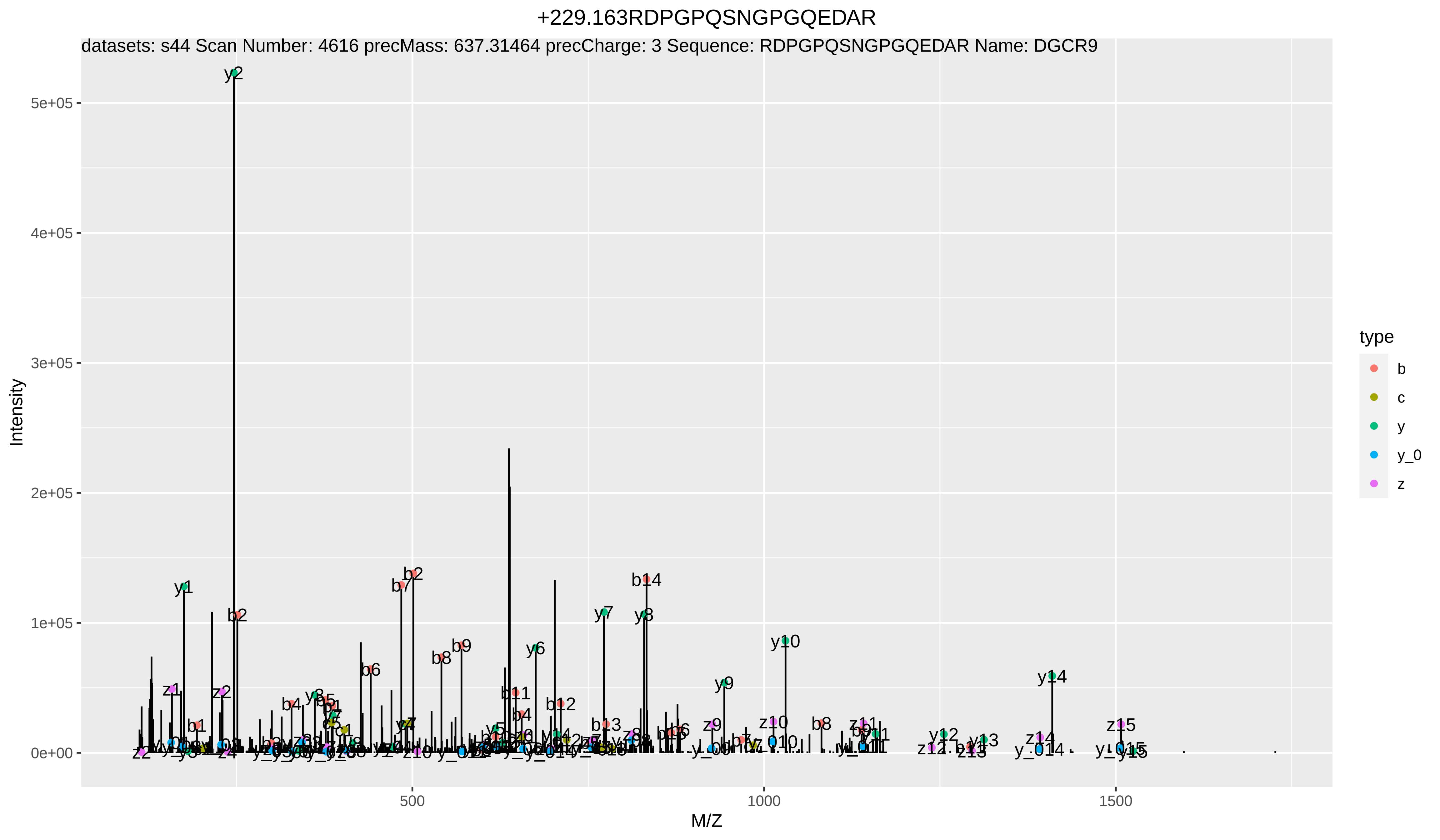
+229.163RDPGPQSNGPGQEDAR
datasets: s44 Scan Number: 4616 precMass: 637.31464 precCharge: 3 Sequence: RDPGPQSNGPGQEDAR Name: DGCR9
Intensity
M/Z
type
b
c
y
y_0
z
0e+00
1e+05
2e+05
3e+05
4e+05
5e+05
500
1000
1500
y2
y1
b2
b7
b8
b9
b6
y6
y7
b14
y8
y10
y9
y14
b11
b12
b13
z15
z10
z11
y12
y13
z14

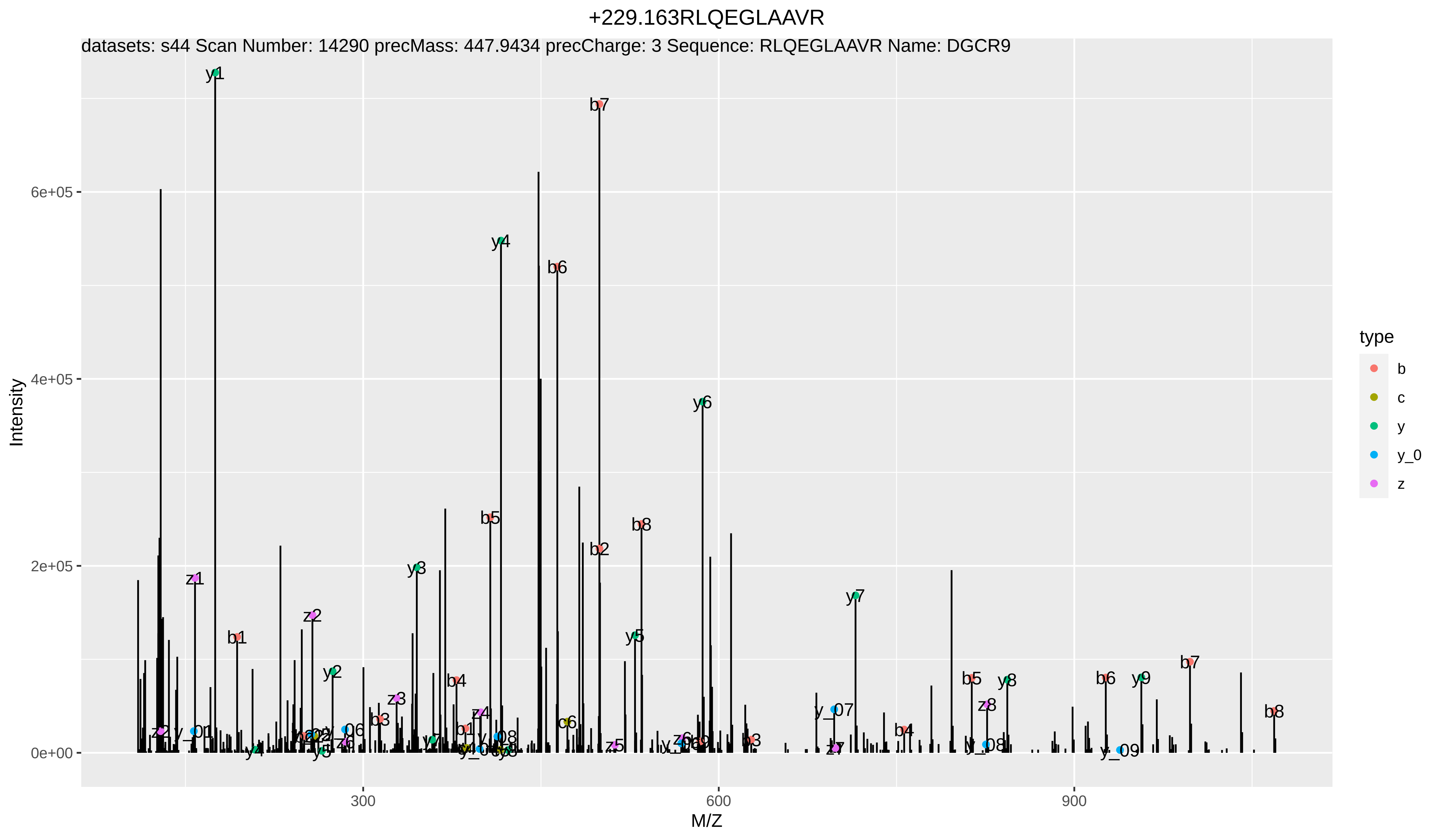
+229.163RLQEGLAAVR
datasets: s44 Scan Number: 14290 precMass: 447.9434 precCharge: 3 Sequence: RLQEGLAAVR Name: DGCR9
Intensity
M/Z
type
b
c
y
y_0
z
y1
b7
y4
b6
y6
b5
b8
b2
y3
z1
y7
z2
b1
y5
y2
b4
z3
b3
c6
b5
y8
b6
y9
b7
z8
y_07
b8
b4
y_08
y_09
z7
0e+00
2e+05
4e+05
6e+05
300
600
900

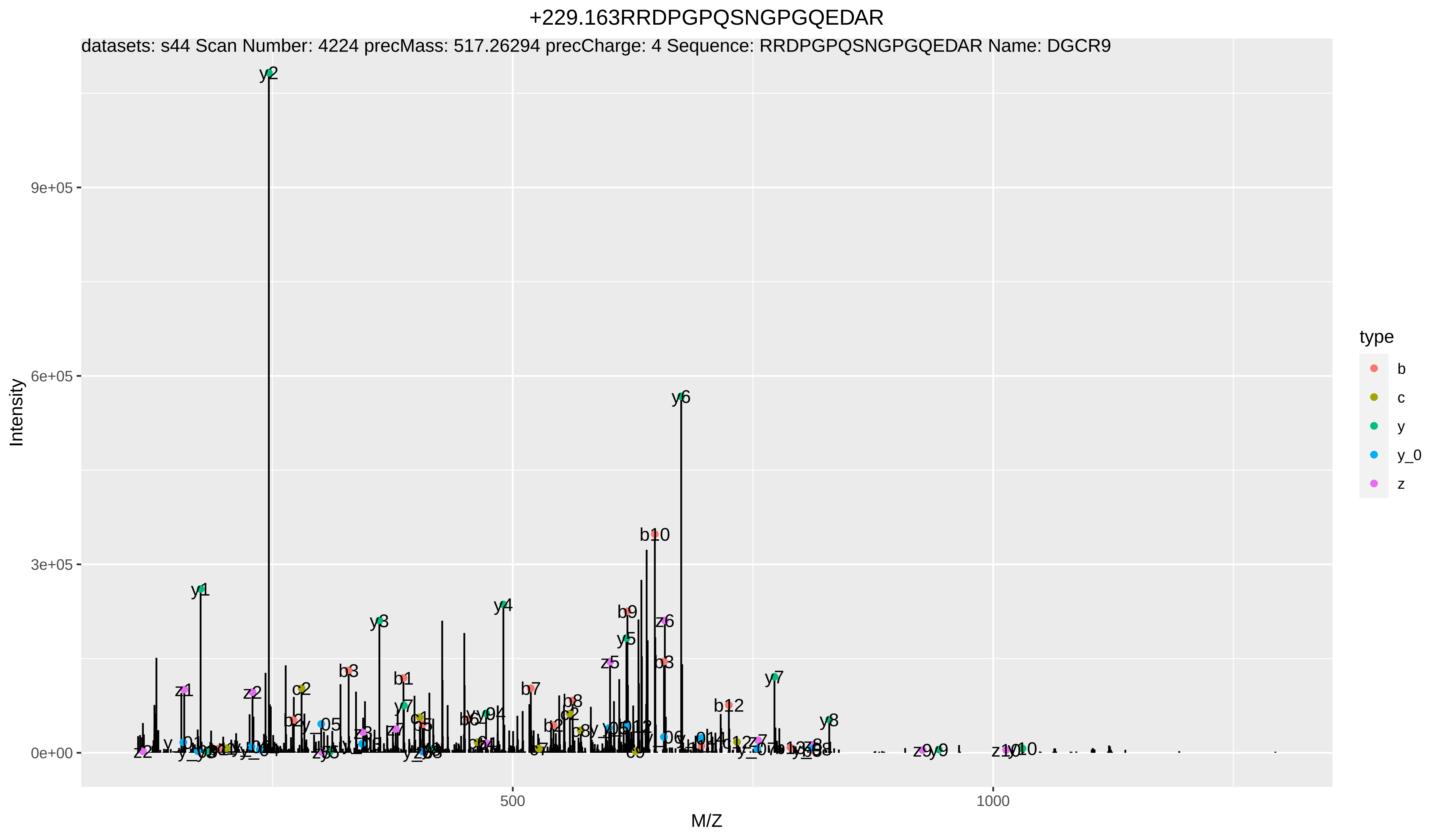

+229.163RRDPGPQSNGPGQEDAR
datasets: s44 Scan Number: 4224 precMass: 517.26294 precCharge: 4 Sequence: RRDPGPQSNGPGQEDAR Name: DGCR9
Intensity
9e+05
6e+05
3e+05
0e+00
M/Z
500
1000
type
b
c
y
y_0
z
y2
y6
b10
y1
y3
y4
b9
z6
y5
z5
y7
b12
y8
b7
z1
z2
c2
b3

+229.163DPGPQSNGPGQEDAR

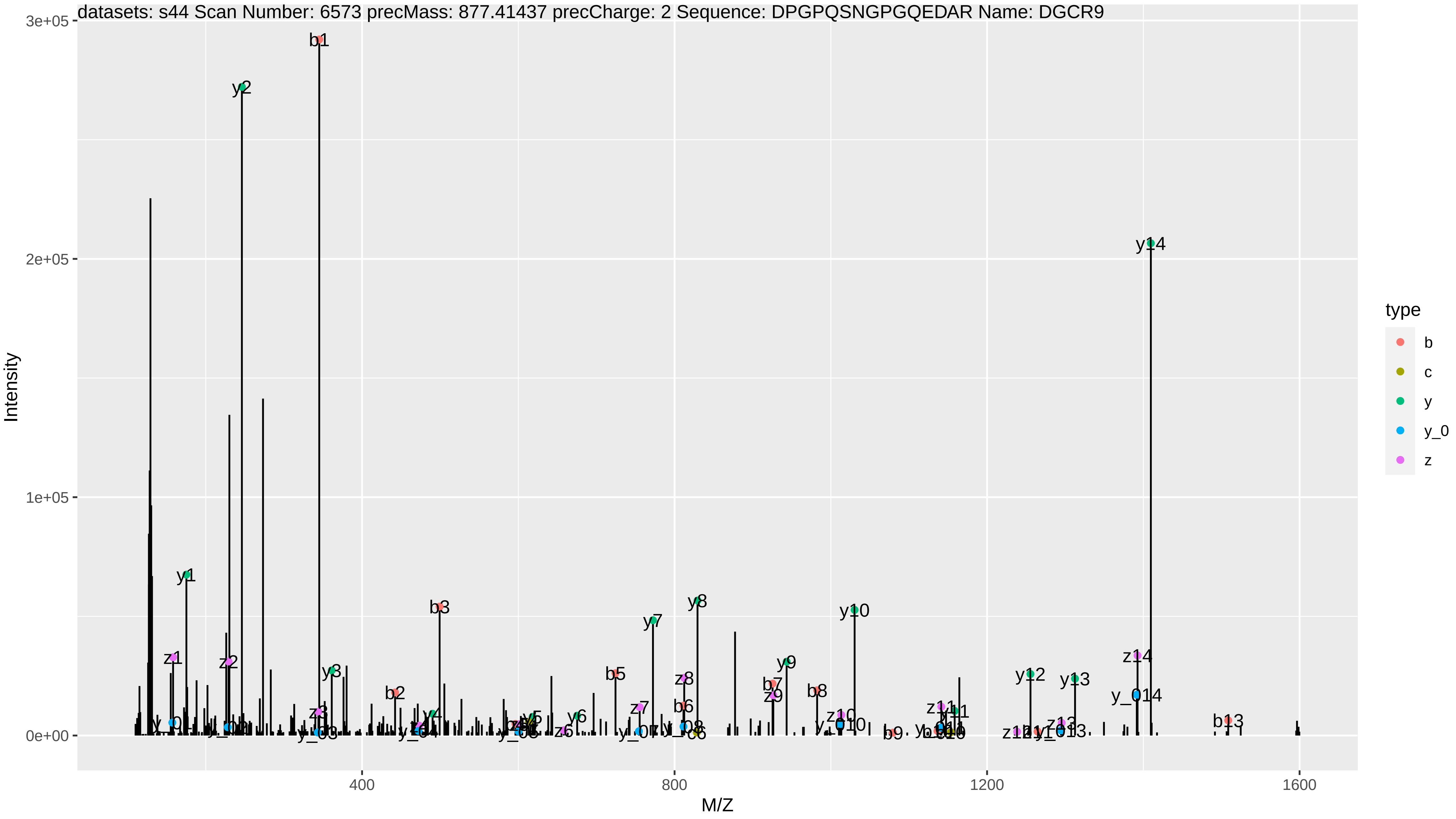

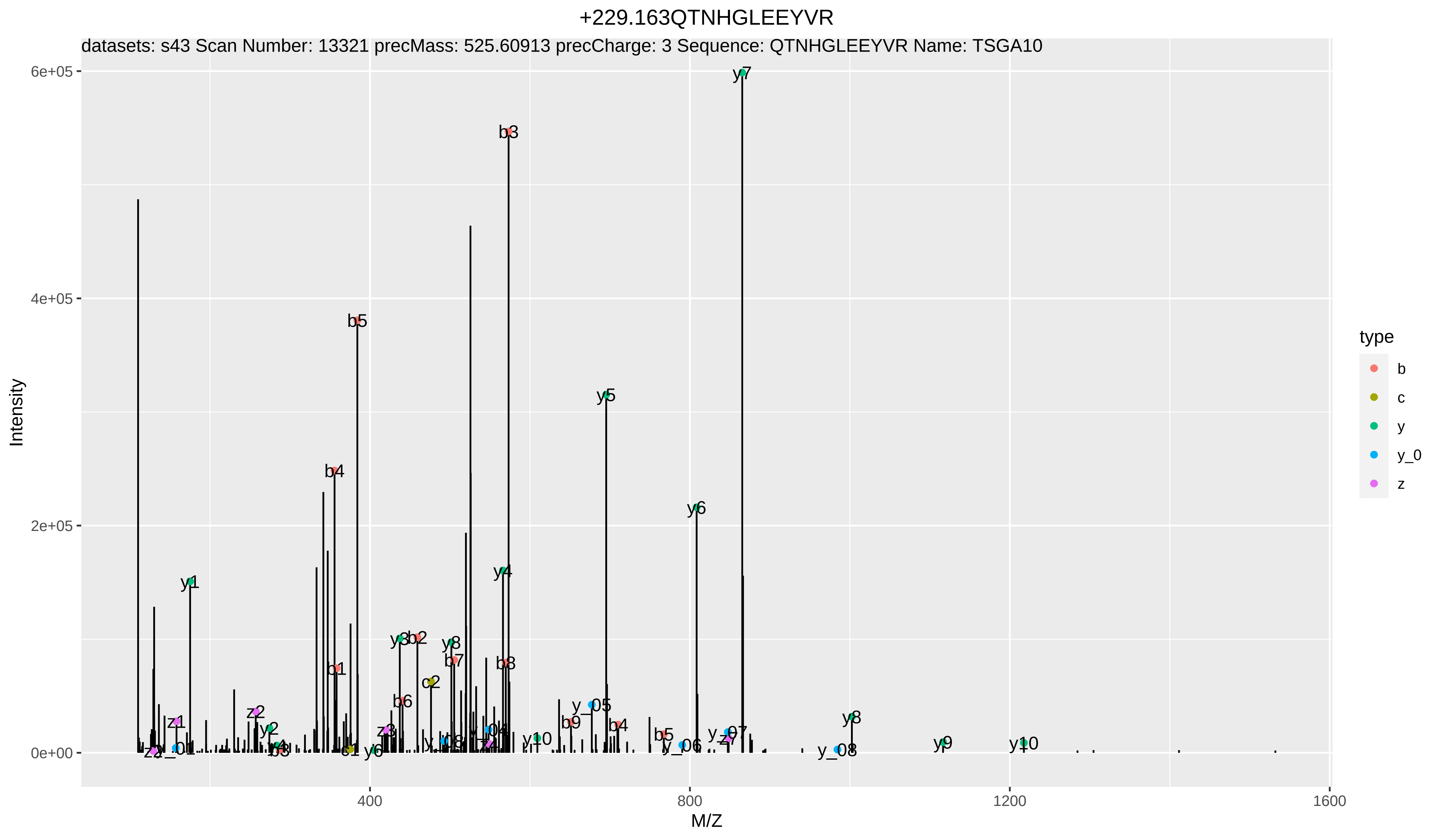

+229.163QTNHGLEEYVR
datasets: s43 Scan Number: 13321 precMass: 525.60913 precCharge: 3 Sequence: QTNHGLEEYVR Name: TSGA10
Intensity
M/Z
type
b
c
y
y_0
z

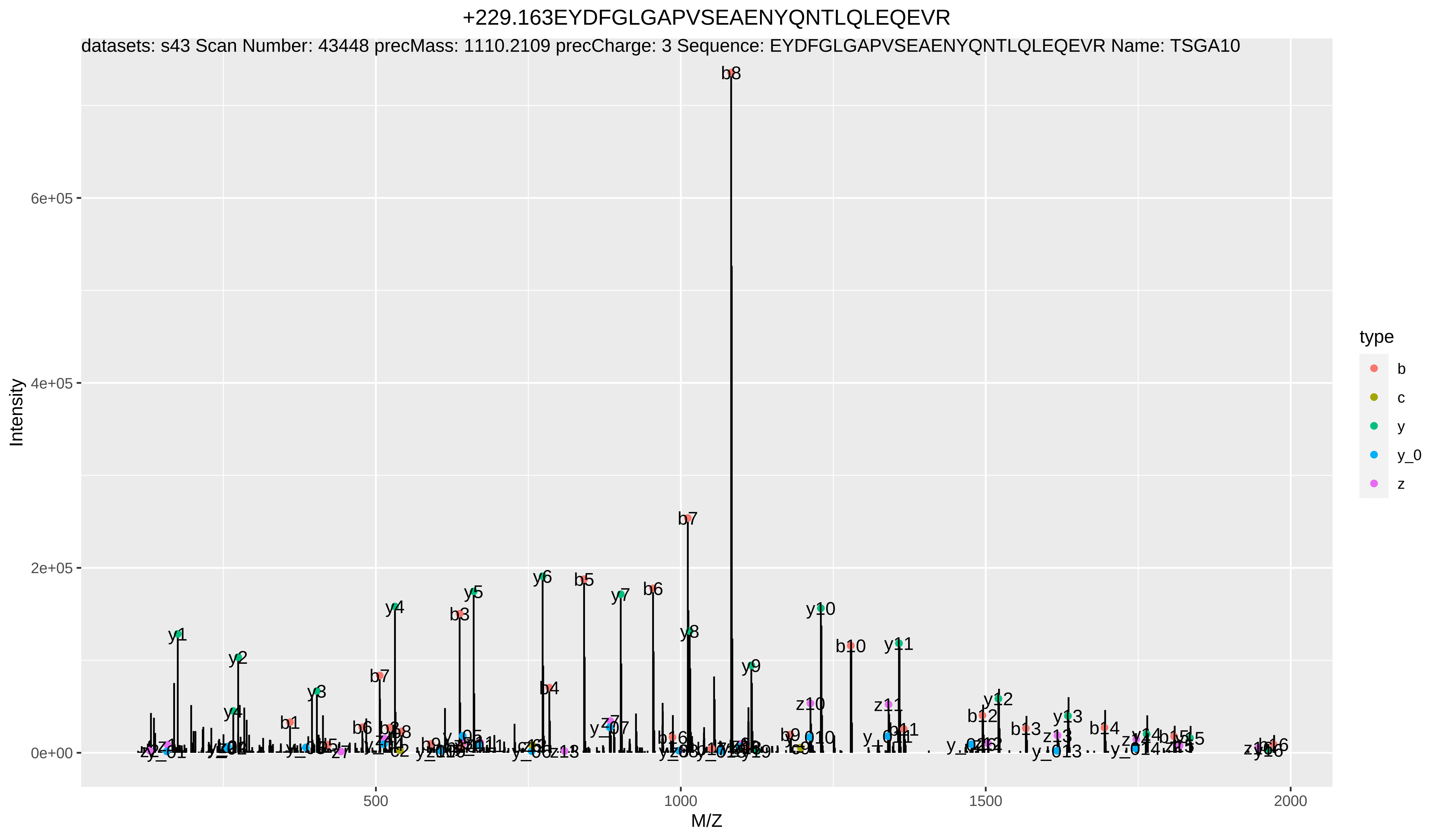

+229.163EYDFGLGAPVSEAENYQNTLQLEQEVR
datasets: s43 Scan Number: 43448 precMass: 1110.2109 precCharge: 3 Sequence: EYDFGLGAPVSEAENYQNTLQLEQEVR Name: TSGA10
Intensity
M/Z
type
b
c
y
y_0
z

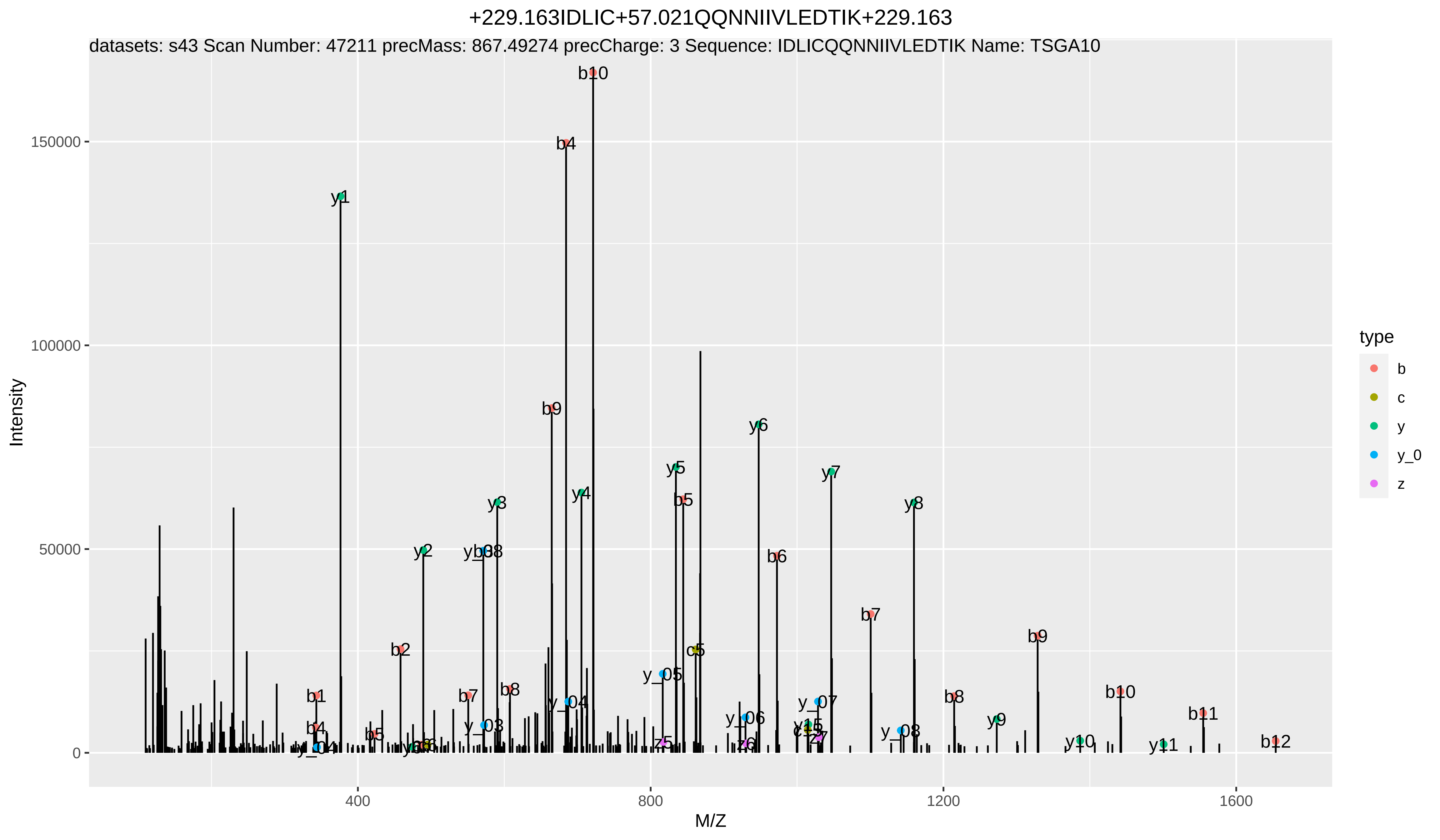
+229.163IDLIC+57.021QQNNIIVLEDTIK+229.163
datasets: s43 Scan Number: 47211 precMass: 867.49274 precCharge: 3 Sequence: IDLICQQNNIIVLEDTIK Name: TSGA10
Intensity
M/Z
type
b
c
y
y_0
z
0
50000
100000
150000
400
800
1200
1600

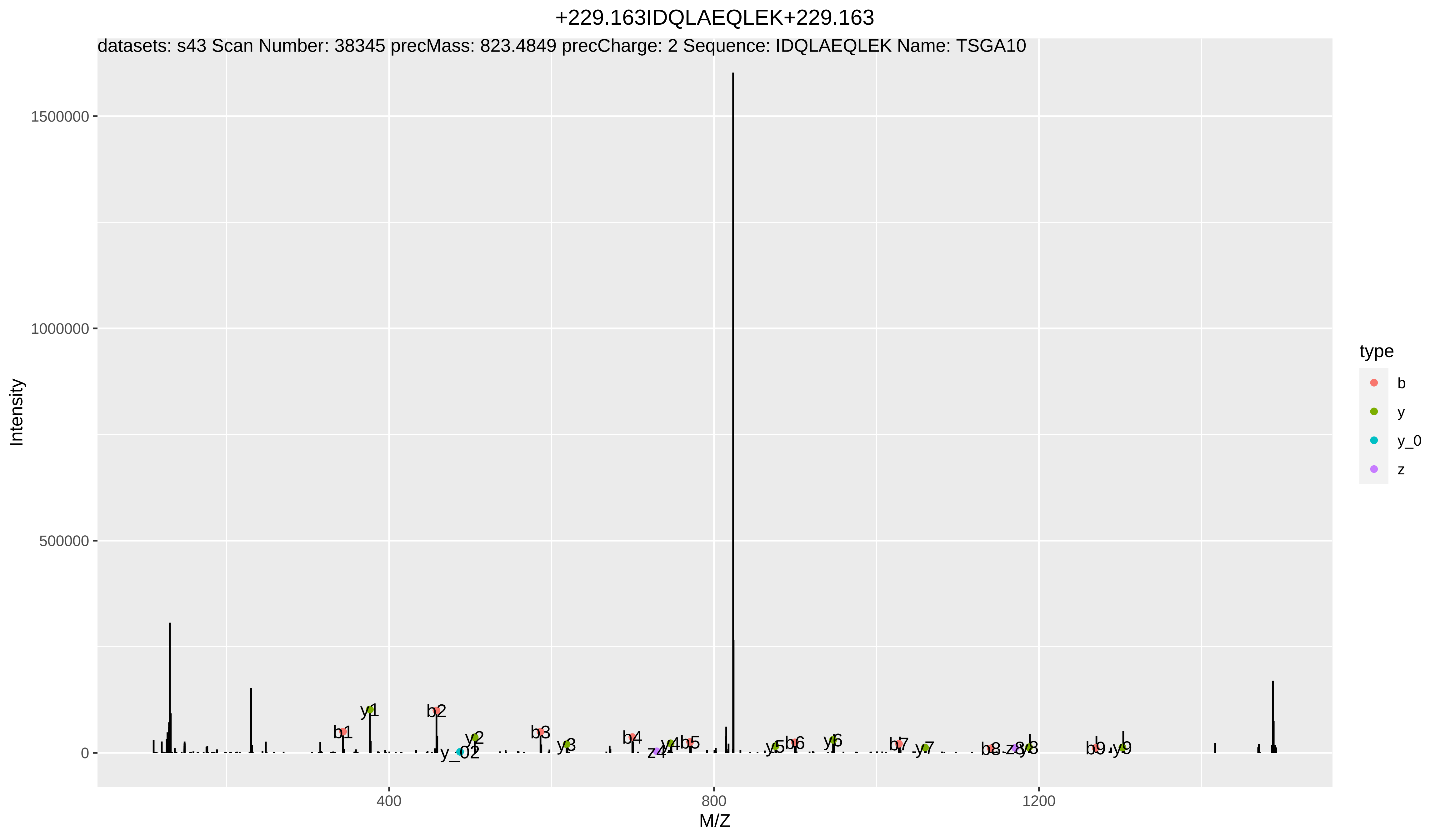
+229.163IDQLAEQLEK+229.163
datasets: s43 Scan Number: 38345 precMass: 823.4849 precCharge: 2 Sequence: IDQLAEQLEK Name: TSGA10
Intensity
1500000
1000000
500000
0
M/Z
400
800
1200
type
b
y
y_0
z
y1
b1
b2
y2
y_02
b3
y3
b4
z4
y4
b5
y5
b6
y6
b7
y7
b8
z8
y8
b9
y9

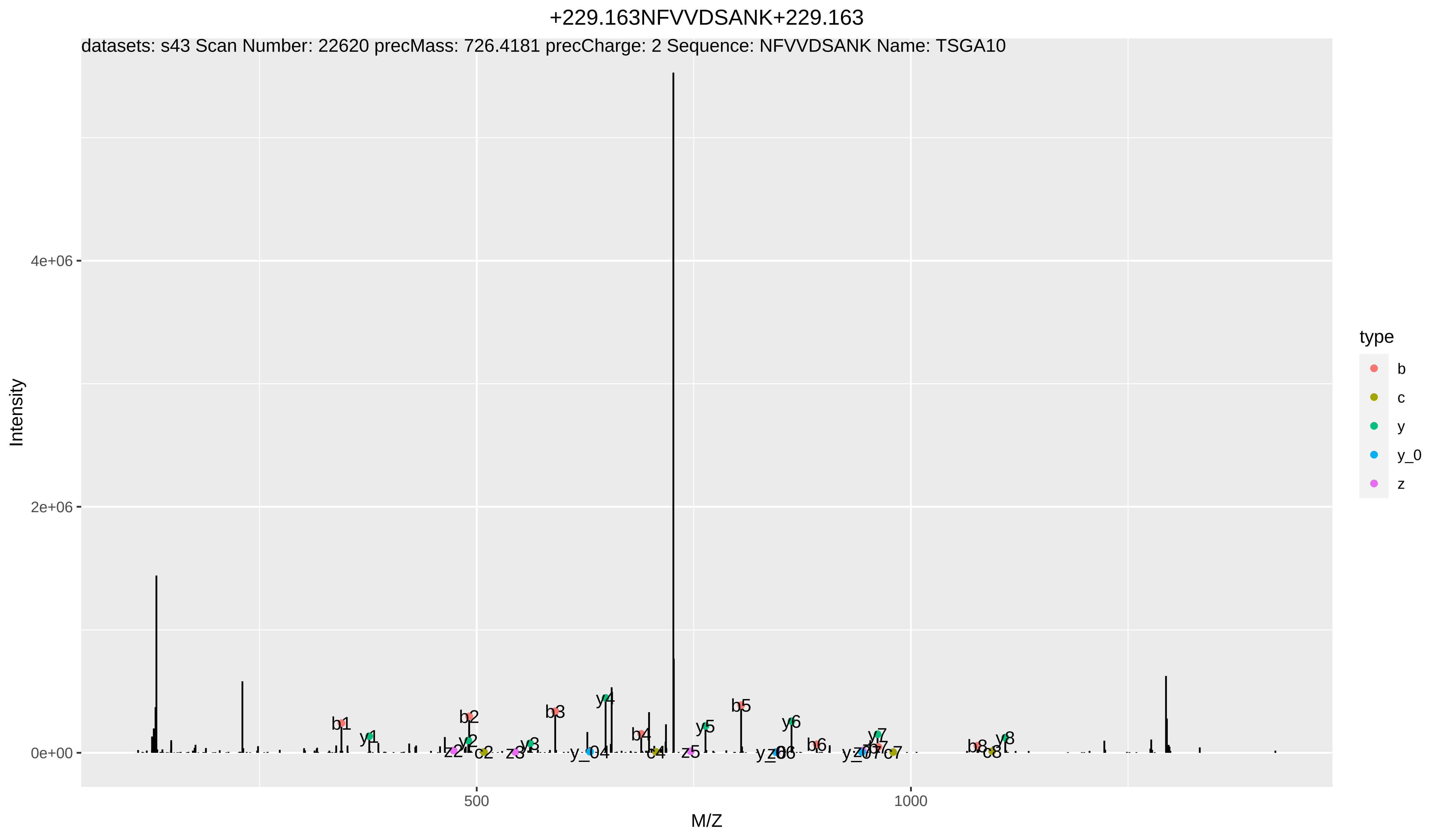
+229.163NFVVDSANK+229.163
datasets: s43 Scan Number: 22620 precMass: 726.4181 precCharge: 2 Sequence: NFVVDSANK Name: TSGA10
Intensity
4e+06
2e+06
0e+00
500
1000
M/Z
type
b
c
y
y_0
z
b1
y1
b2
y2
z2
c2
z3
y3
b3
y4
y_04
b4
c4
z5
y5
b5
y6
y_06
b6
y7
y_07
c7
b8
c8
y8

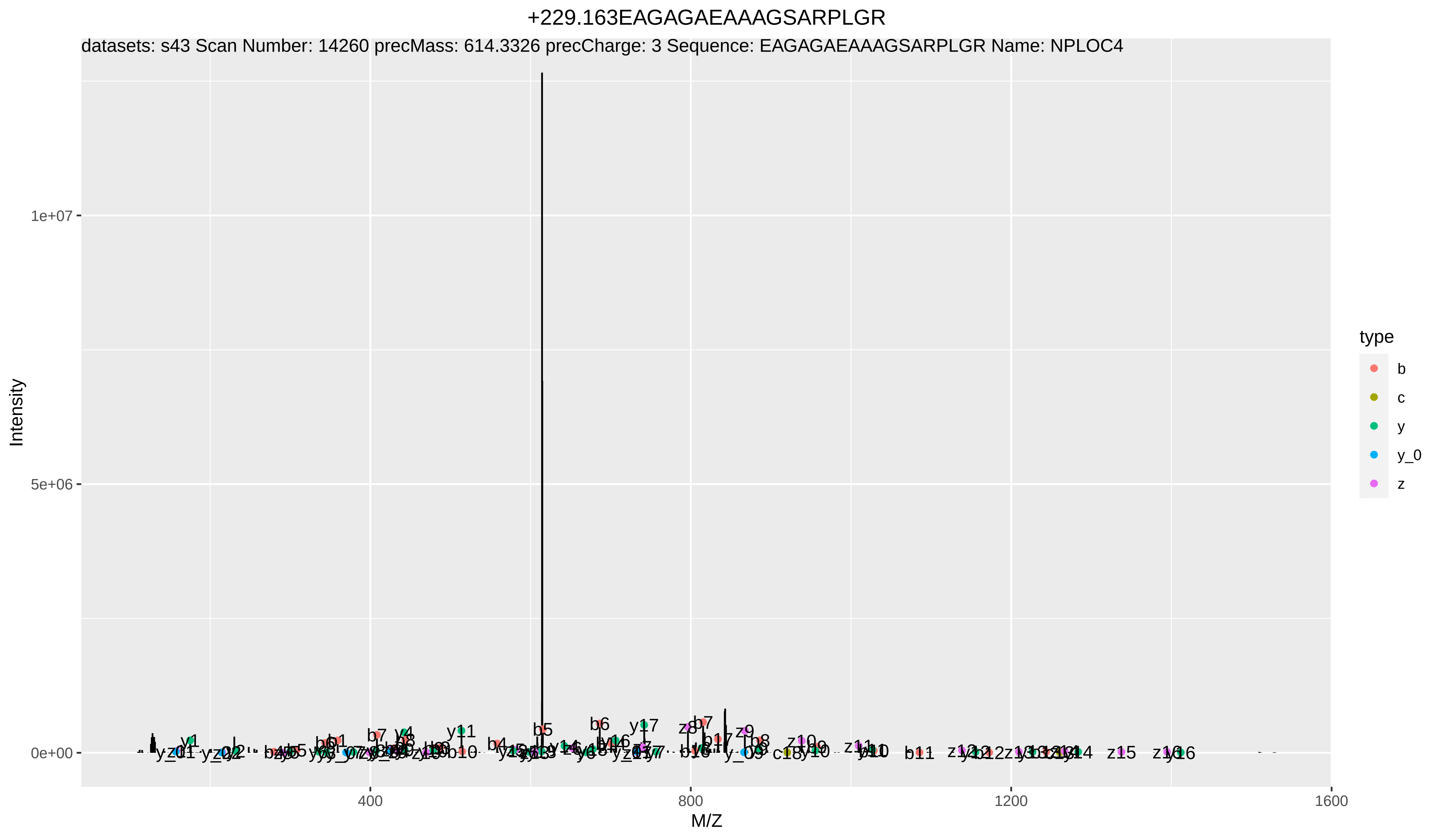
+229.163EAGAGAEAAAGSARPLGR
datasets: s43 Scan Number: 14260 precMass: 614.3326 precCharge: 3 Sequence: EAGAGAEAAAGSARPLGR Name: NPLOC4
Intensity
M/Z
type
b
c
y
y_0
z

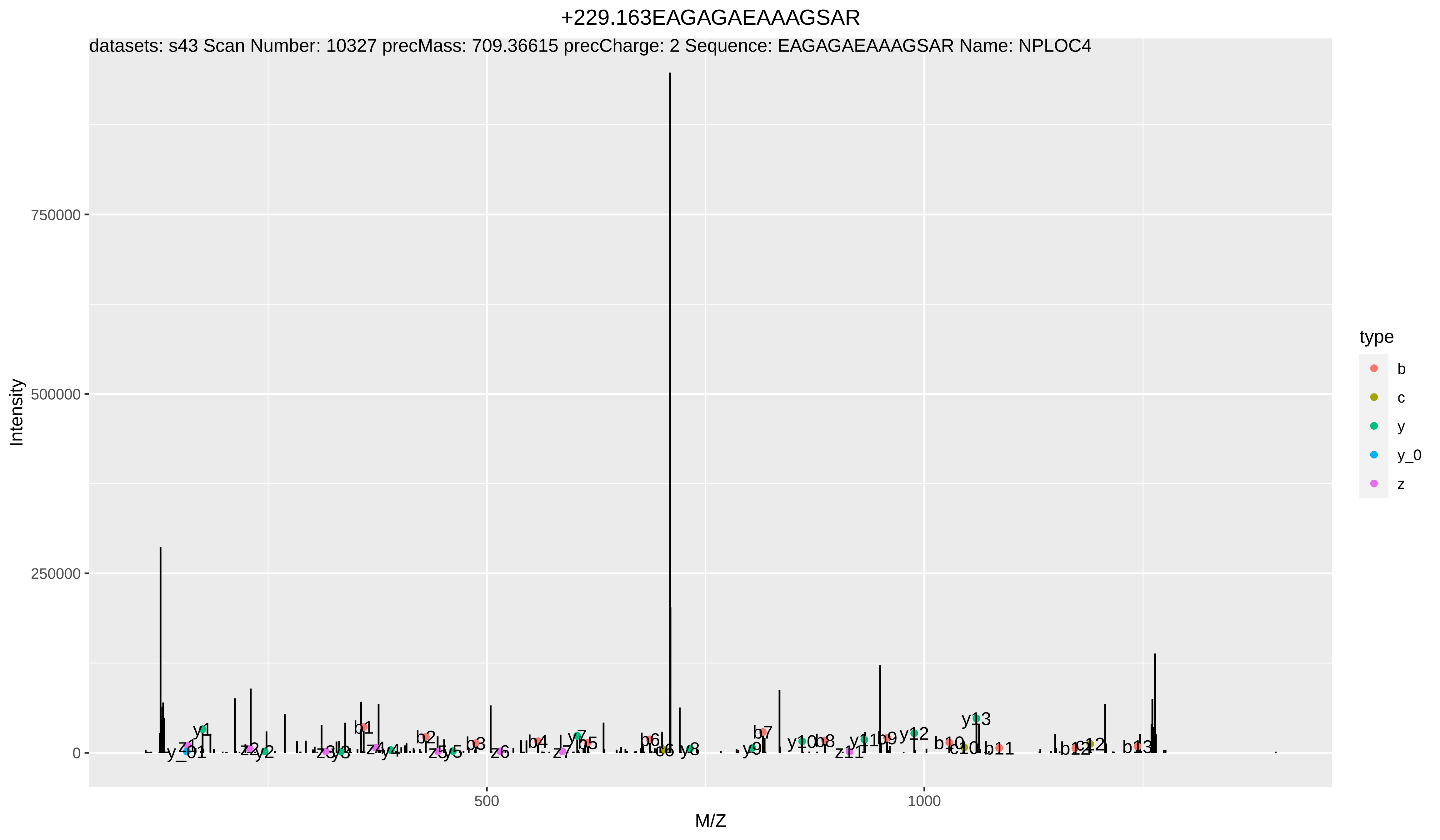
+229.163EAGAGAEAAAGSAR
datasets: s43 Scan Number: 10327 precMass: 709.36615 precCharge: 2 Sequence: EAGAGAEAAAGSAR Name: NPLOC4
Intensity
M/Z
type
b
c
y
y_0
z

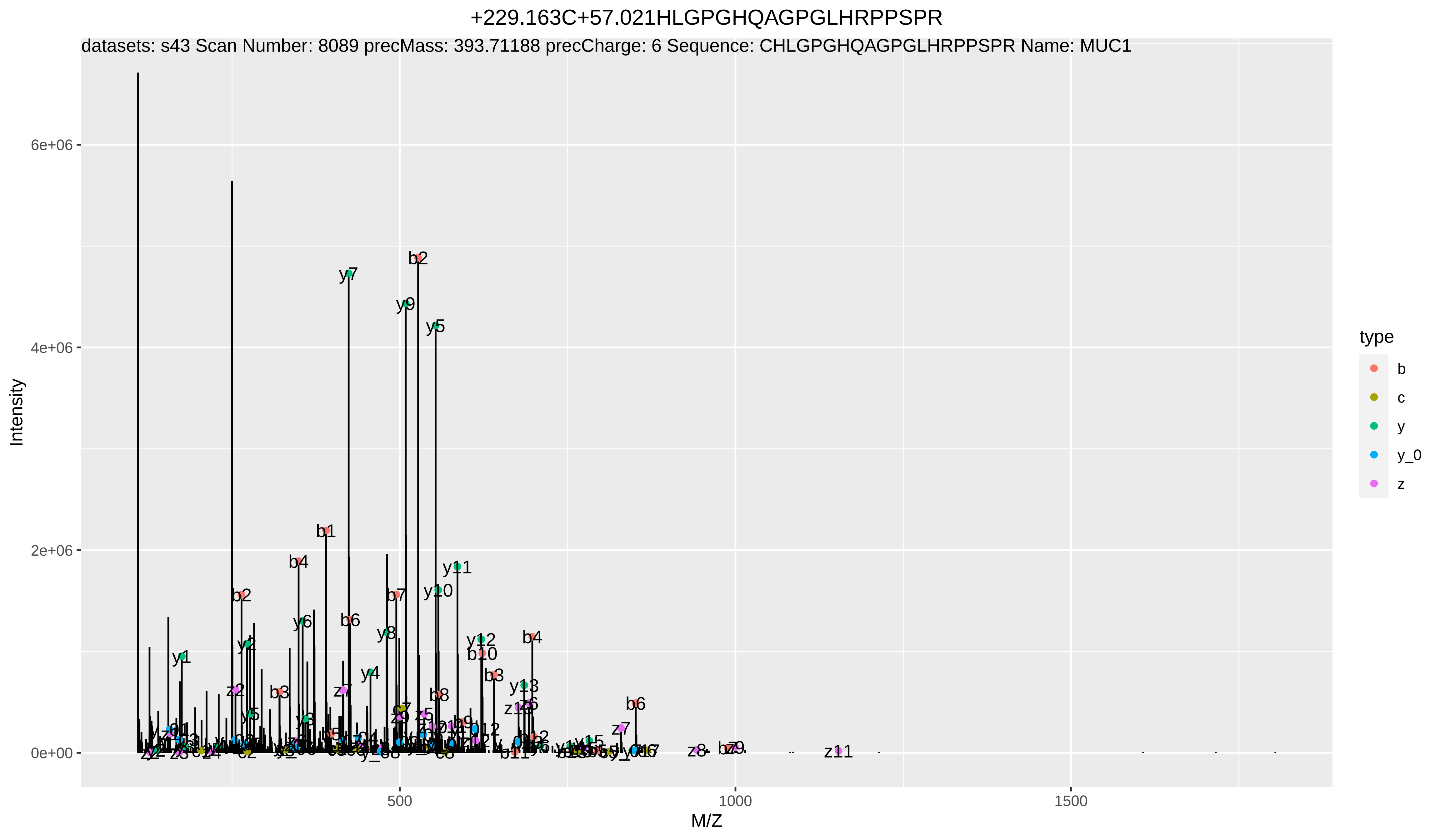
+229.163C+57.021HLGPGHQAGPGLHRPPSPR
datasets: s43 Scan Number: 8089 precMass: 393.71188 precCharge: 6 Sequence: CHLGPGHQAGPGLHRPPSPR Name: MUC1
Intensity
M/Z
6e+06
4e+06
2e+06
0e+00
500
1000
1500
type
b
c
y
y_0
z
b2
y7
y9
y5
b1
b4
y11
y10
b2
b7
y6
b6
y8
y2
y12
b4
b10
y1
y4
b3
y13
z2
b3
z7
b8
b6
z7
z8
z11

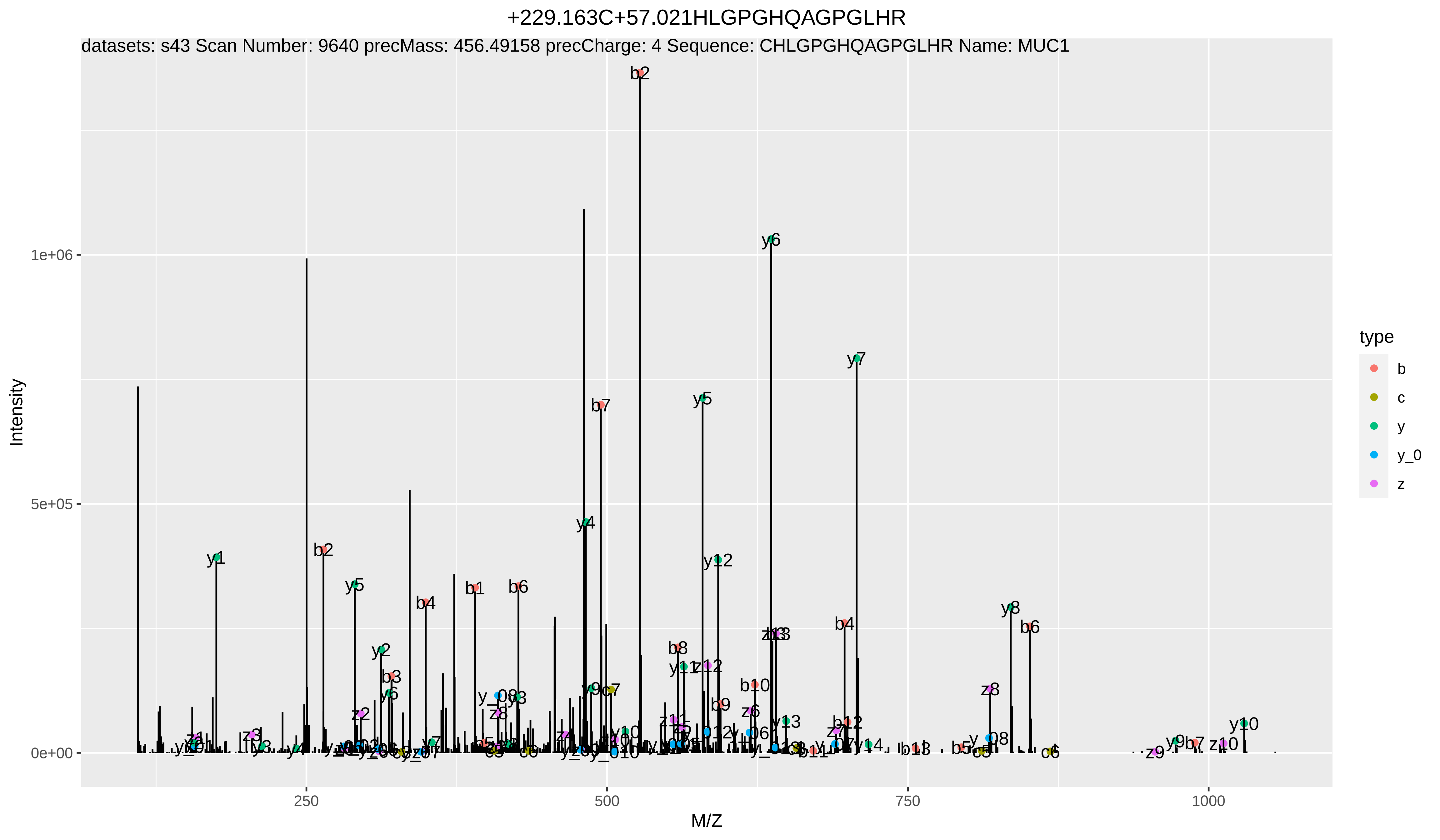
+229.163C+57.021HLGPGHQAGPGLHR
datasets: s43 Scan Number: 9640 precMass: 456.49158 precCharge: 4 Sequence: CHLGPGHQAGPGLHR Name: MUC1
Intensity
M/Z
0e+00
5e+05
1e+06
250
500
750
1000
type
b
c
y
y_0
z

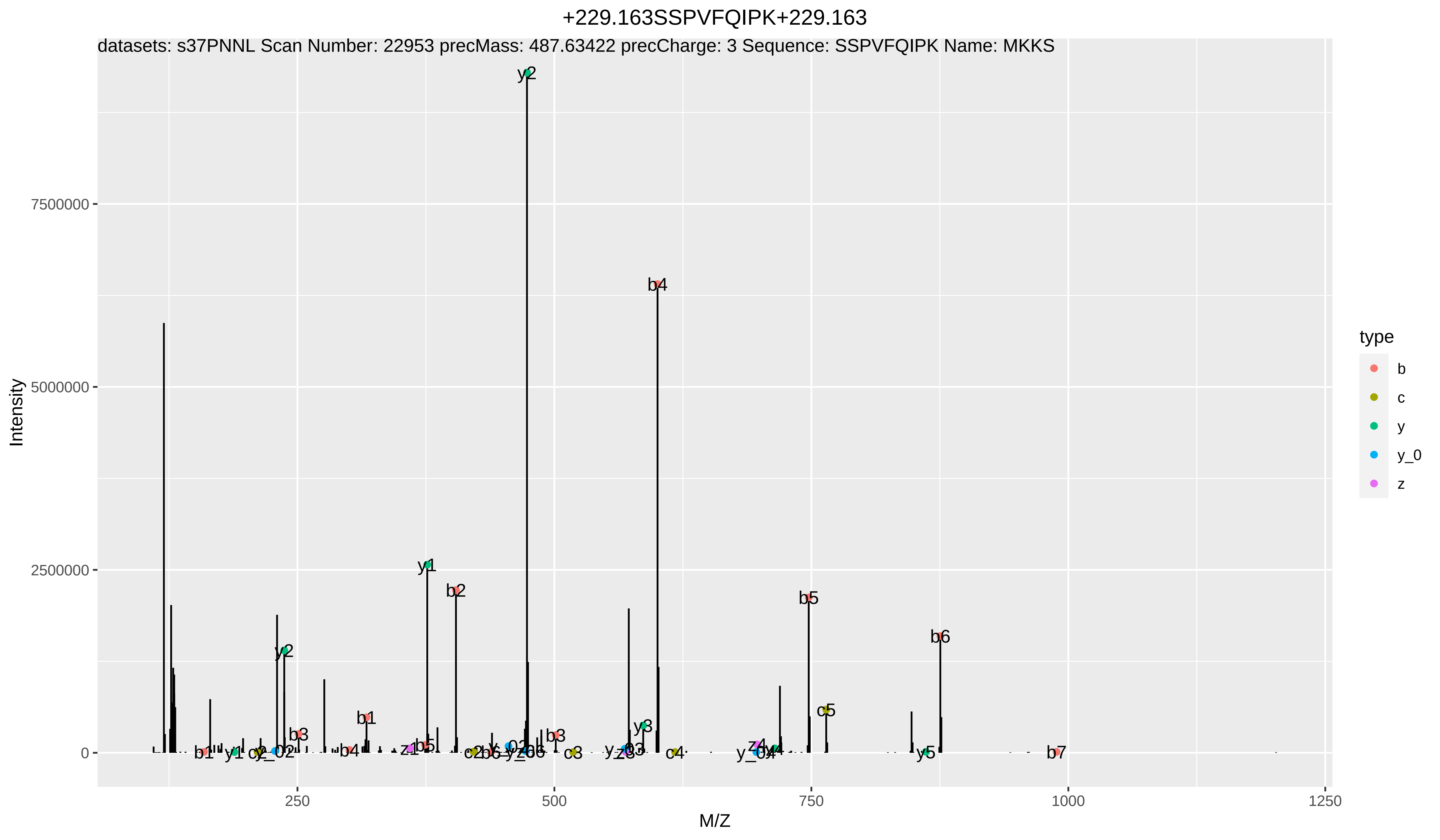
+229.163SSPVFQIPK+229.163
datasets: s37PNNL Scan Number: 22953 precMass: 487.63422 precCharge: 3 Sequence: SSPVFQIPK Name: MKKS
Intensity
M/Z
type
b
c
y
y_0
z
y2
b4
y1
b2
b5
b6
y2
c5
b1
b3
y3
b3
z4
y5
b7
c3
c4
b1
y1
b4
z1
b5
0
2500000
5000000
7500000
250
500
750
1000
1250

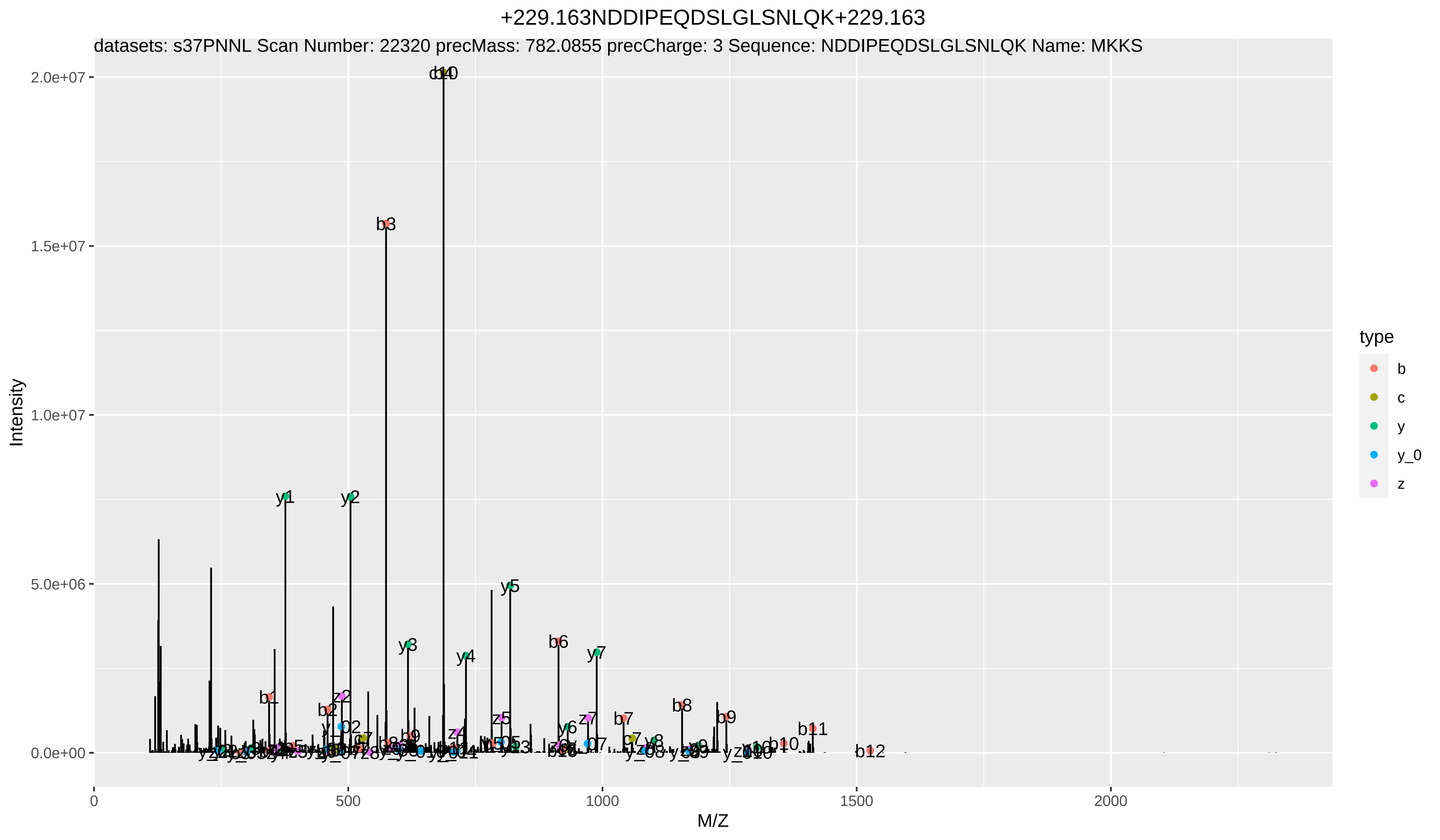
+229.163NDDIPEQDSLGLSNLQK+229.163
datasets: s37PNNL Scan Number: 22320 precMass: 782.0855 precCharge: 3 Sequence: NDDIPEQDSLGLSNLQK Name: MKKS
Intensity
M/Z
0.0e+00
5.0e+06
1.0e+07
1.5e+07
2.0e+07
0
500
1000
1500
2000
type
b
c
y
y_0
z
b3
y1
y2
y3
y4
y5
b6
y7
b1
b2
z2
z5
z7
b7
b8
b9
b11
b10
b12
y6
y8
y9

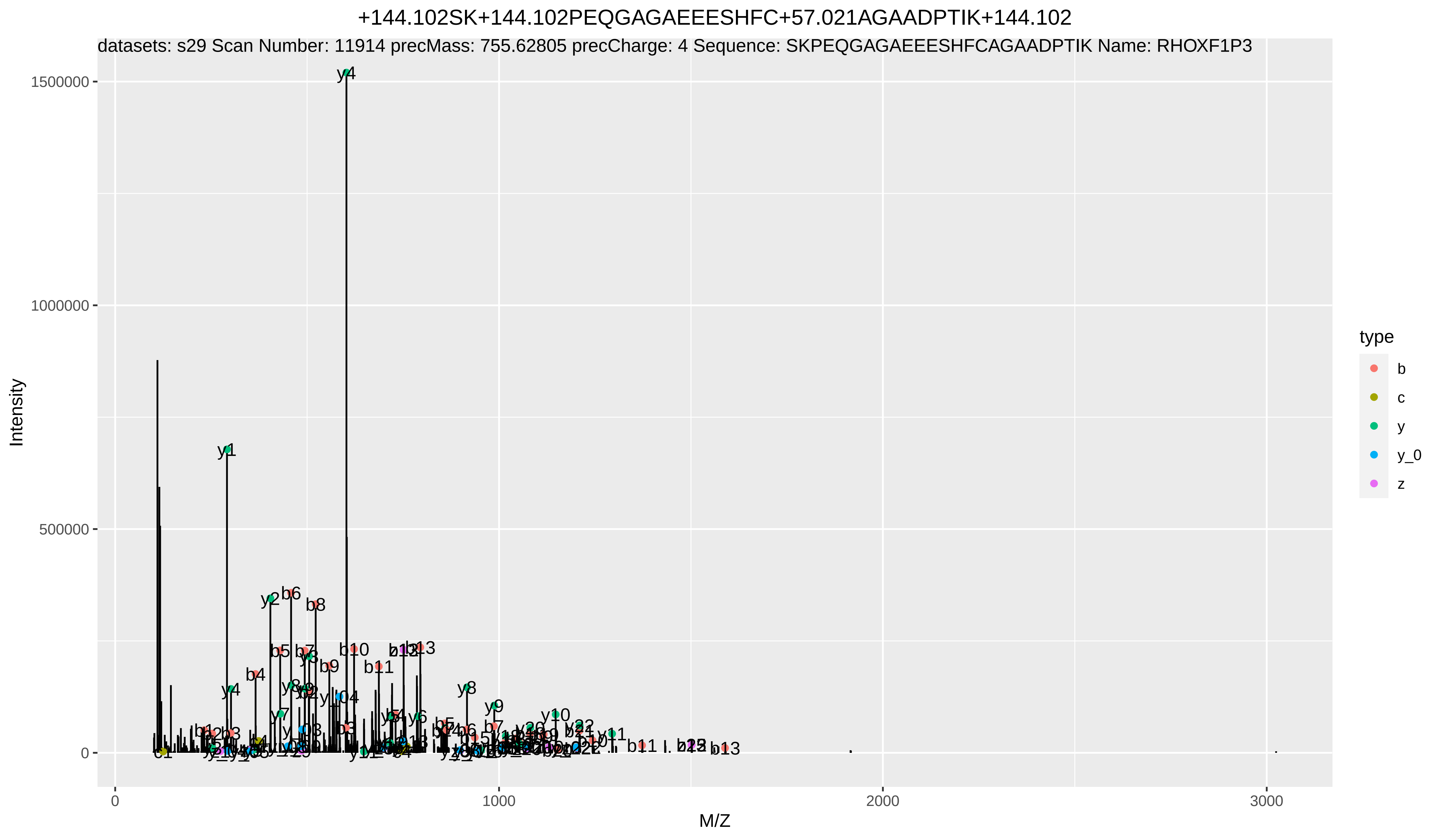

+144.102SK+144.102PEQGAGAEEESHFC+57.021AGAADPTIK+144.102
datasets: s29 Scan Number: 11914 precMass: 755.62805 precCharge: 4 Sequence: SKPEQGAGAEEESHFCAGAADPTIK Name: RHOXF1P3
Intensity
1500000
1000000
500000
0
0
1000
2000
3000
M/Z
type
b
c
y
y_0
z

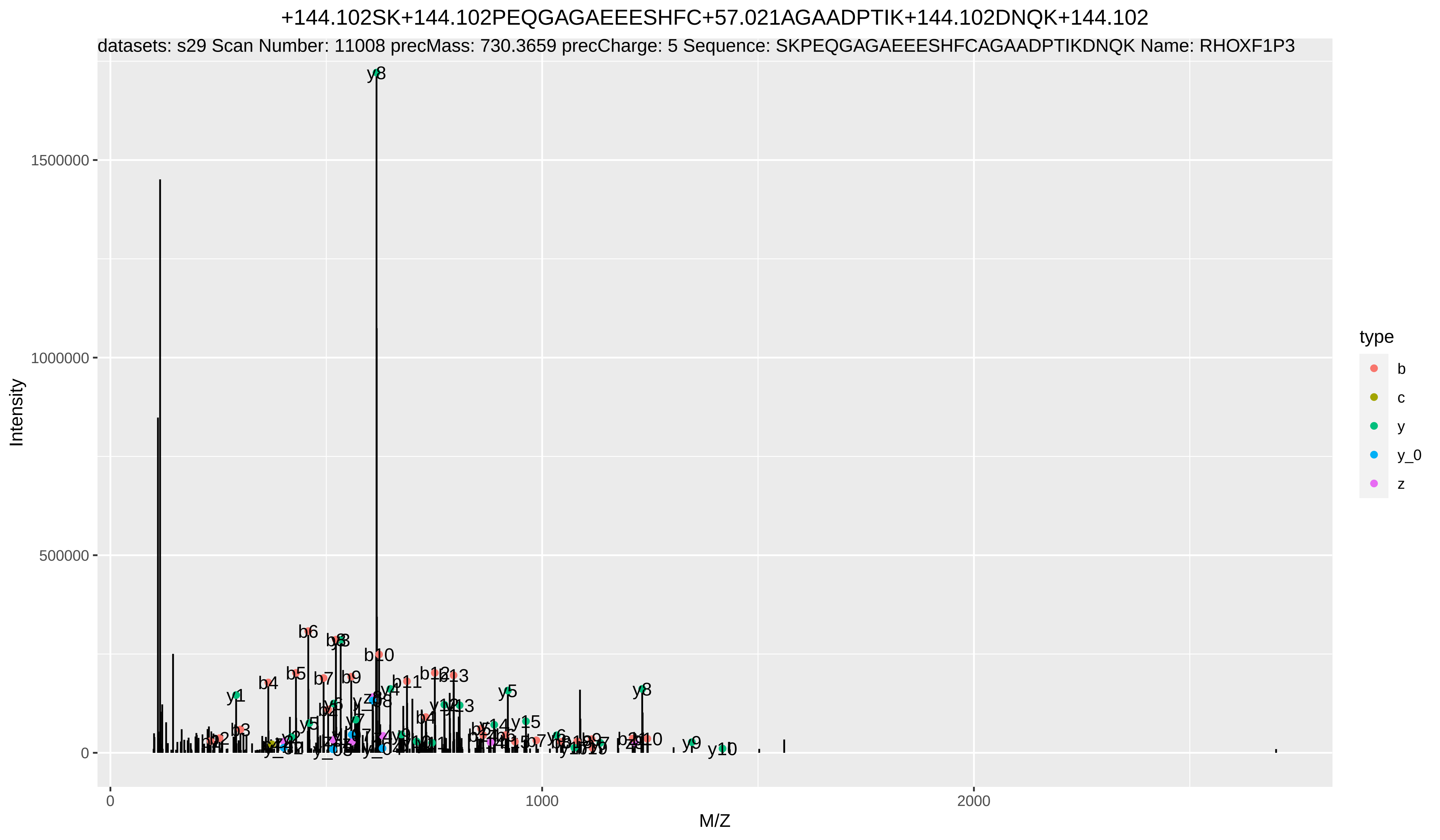

+144.102SK+144.102PEQGAGAEEESHFC+57.021AGAADPTIK+144.102DNQK+144.102
datasets: s29 Scan Number: 11008 precMass: 730.3659 precCharge: 5 Sequence: SKPEQGAGAEEESHFCAGAADPTIKDNQK Name: RHOXF1P3
Intensity
1500000
1000000
500000
0
M/Z
0
1000
2000
type
b
c
y
y_0
z

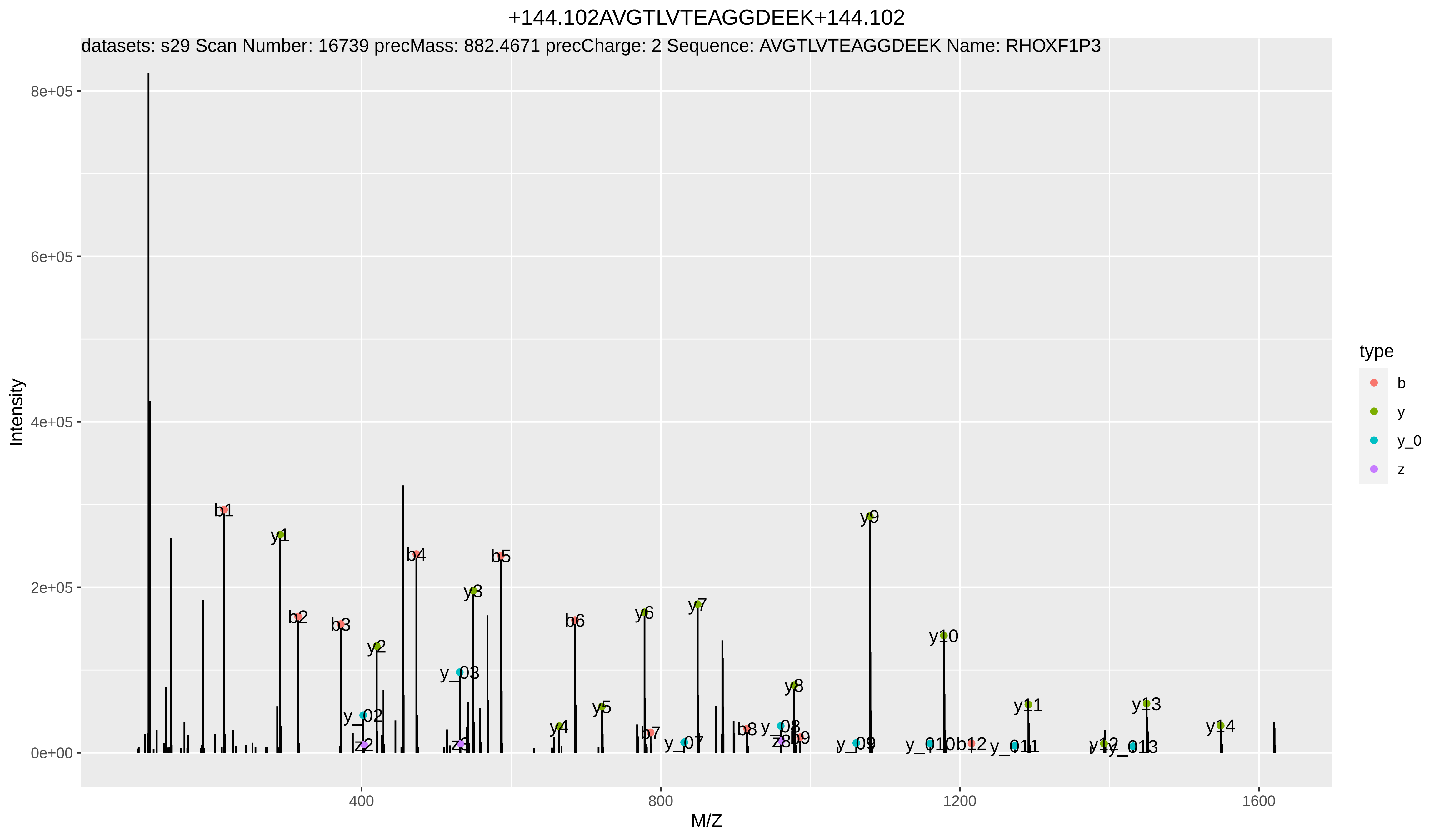
+144.102AVGTLVTEAGGDEEK+144.102
datasets: s29 Scan Number: 16739 precMass: 882.4671 precCharge: 2 Sequence: AVGTLVTEAGGDEEK Name: RHOXF1P3
Intensity
8e+05
6e+05
4e+05
2e+05
0e+00
400
800
1200
1600
M/Z
type
b
y
y_0
z
b1
y1
b2
b3
y2
b4
b5
y3
y_03
y_02
z2
z3
y4
y5
b6
y6
b7
y7
y_07
b8
y_08
y8
y9
y_09
y10
y_010
b12
y11
y_011
y12
y13
y_013
y14

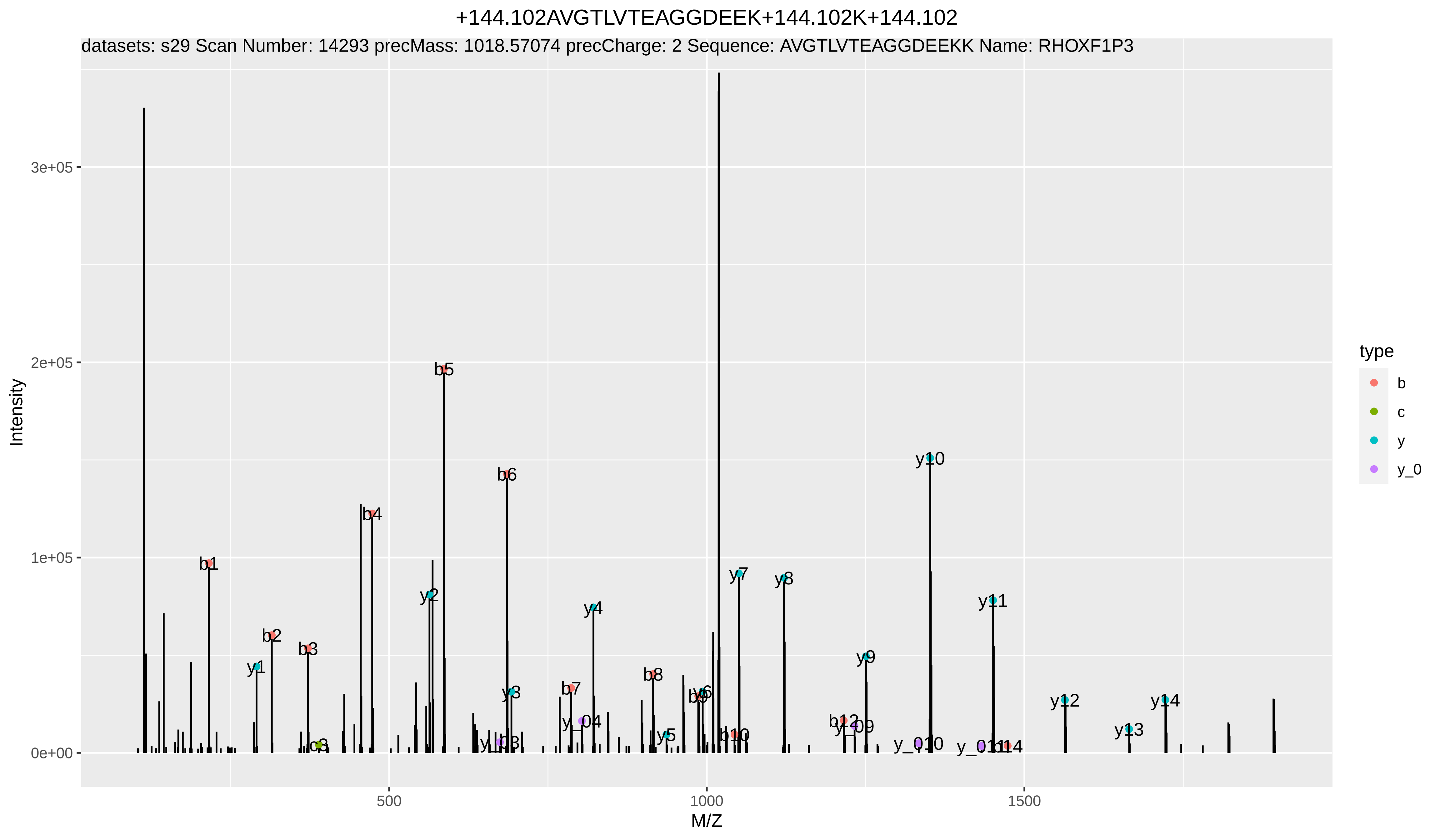

+144.102AVGTLVTEAGGDEEK+144.102K+144.102
datasets: s29 Scan Number: 14293 precMass: 1018.57074 precCharge: 2 Sequence: AVGTLVTEAGGDEEKK Name: RHOXF1P3
Intensity
M/Z
type
b
c
y
y_0

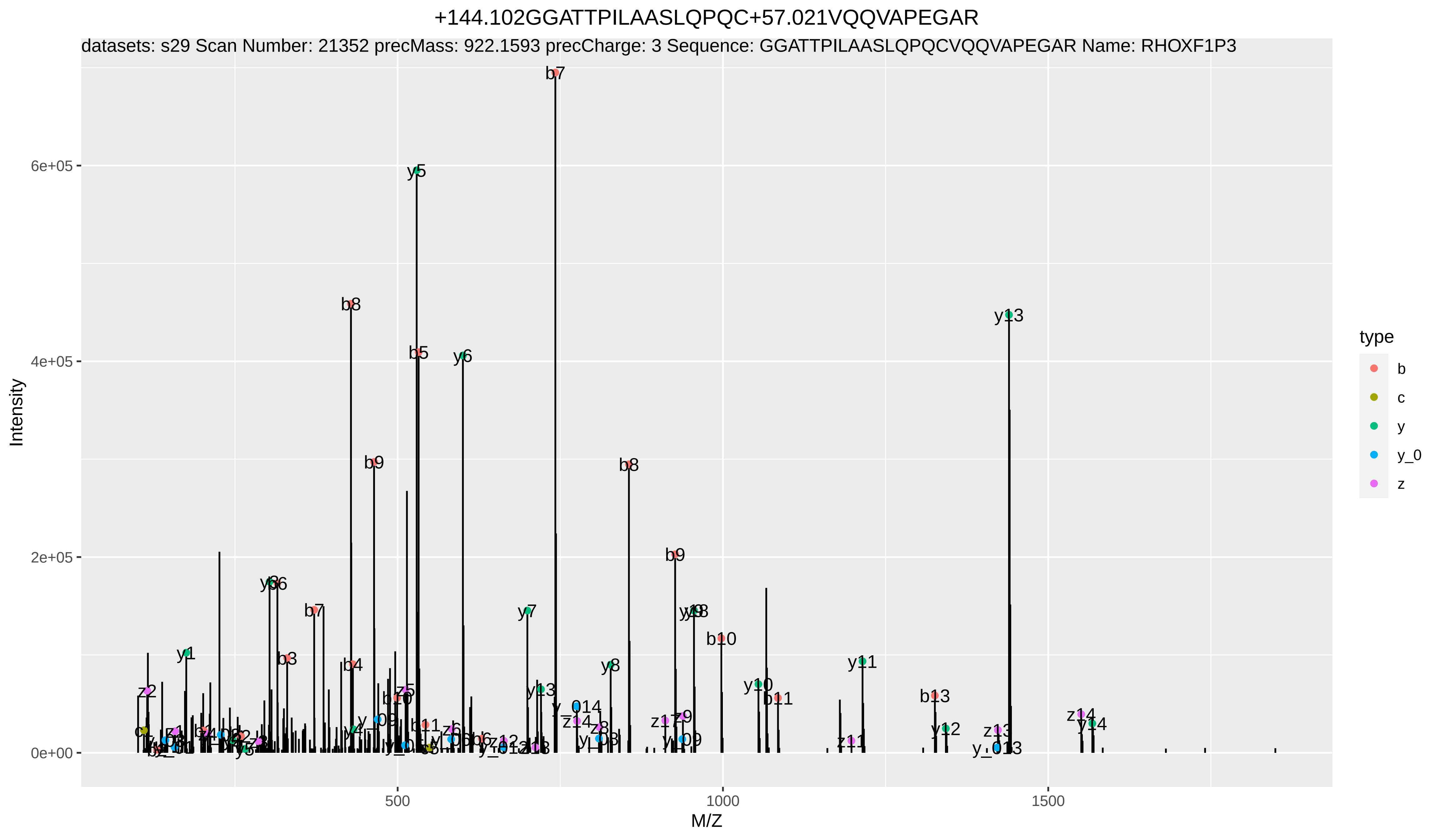
+144.102GGATTPILAASLQPQC+57.021VQQVAPEGAR
datasets: s29 Scan Number: 21352 precMass: 922.1593 precCharge: 3 Sequence: GGATTPILAASLQPQCVQQVAPEGAR Name: RHOXF1P3
Intensity
M/Z
type
b
c
y
y_0
z

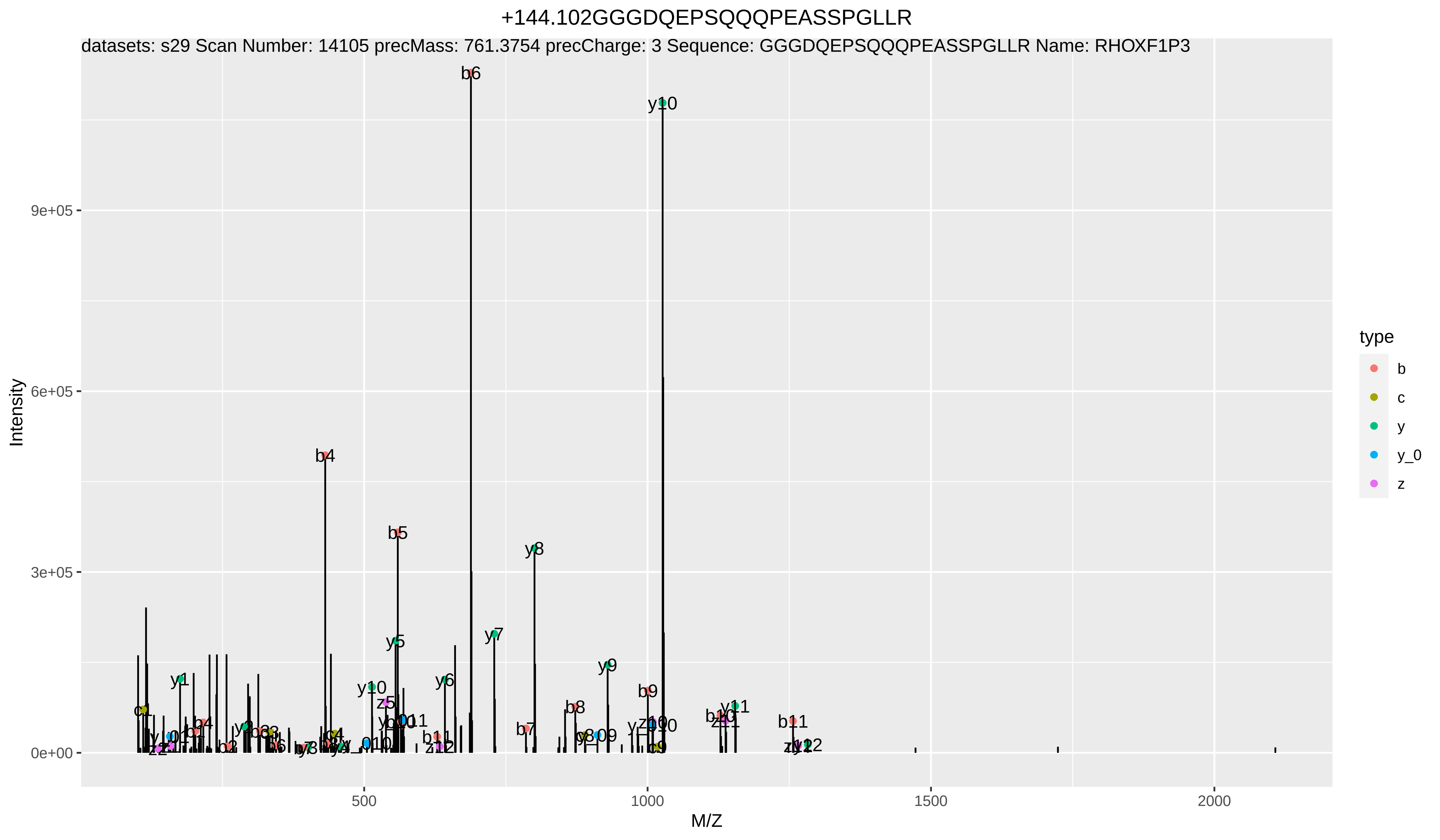
+144.102GGGDQEPSQQQPEASSPGLLR
datasets: s29 Scan Number: 14105 precMass: 761.3754 precCharge: 3 Sequence: GGGDQEPSQQQPEASSPGLLR Name: RHOXF1P3
Intensity
M/Z
0e+00
3e+05
6e+05
9e+05
500
1000
1500
2000
type
b
c
y
y_0
z
b6
y10
b4
b5
y8
y7
y5
y9
y6
y1
b9
b8
y11
b11
b7
y12

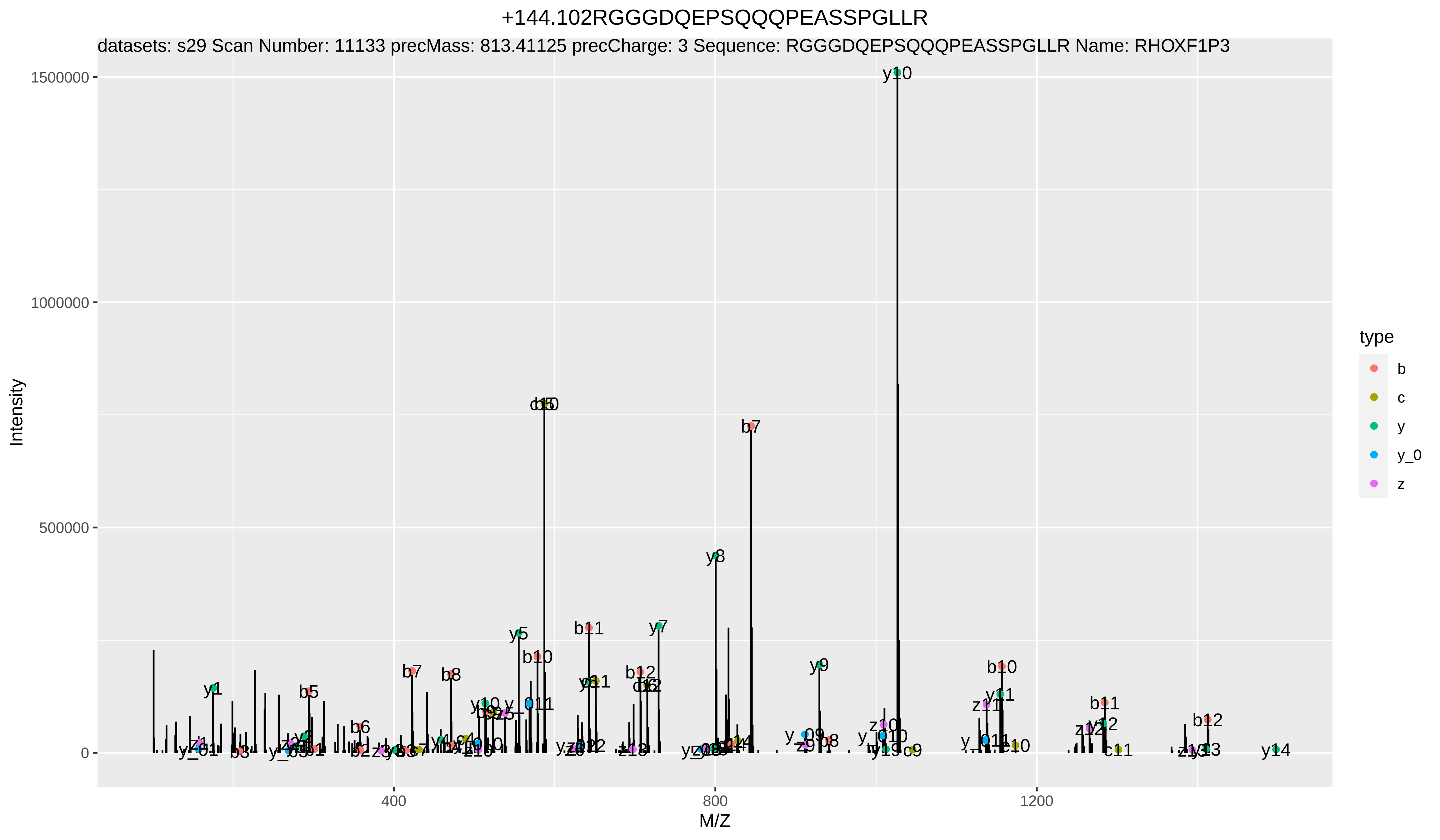
+144.102RGGGDQEPSQQQPEASSPGLLR
datasets: s29 Scan Number: 11133 precMass: 813.41125 precCharge: 3 Sequence: RGGGDQEPSQQQPEASSPGLLR Name: RHOXF1P3
Intensity
1500000
1000000
500000
0
400
800
1200
M/Z
type
b
c
y
y_0
z
y10
b7
y8
y5
b11
y7
b10
y9
y1
b5
b6
b8
b12
b11
y14

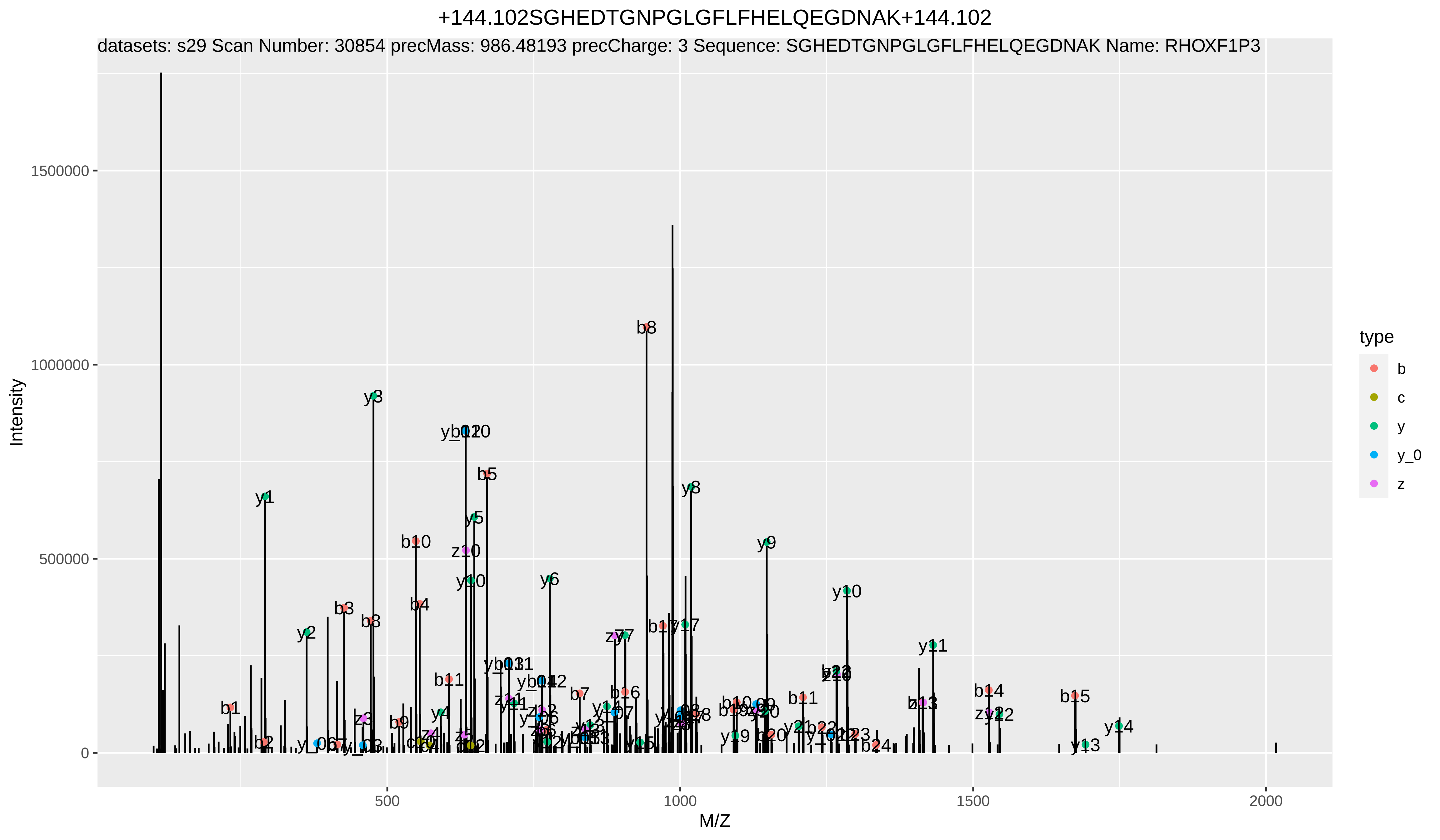
+144.102SGHEDTGNPGLGFLFHELQEGDNAK+144.102
datasets: s29 Scan Number: 30854 precMass: 986.48193 precCharge: 3 Sequence: SGHEDTGNPGLGFLFHELQEGDNAK Name: RHOXF1P3
Intensity
1500000
1000000
500000
0
M/Z
500
1000
1500
2000
type
b
c
y
y_0
z

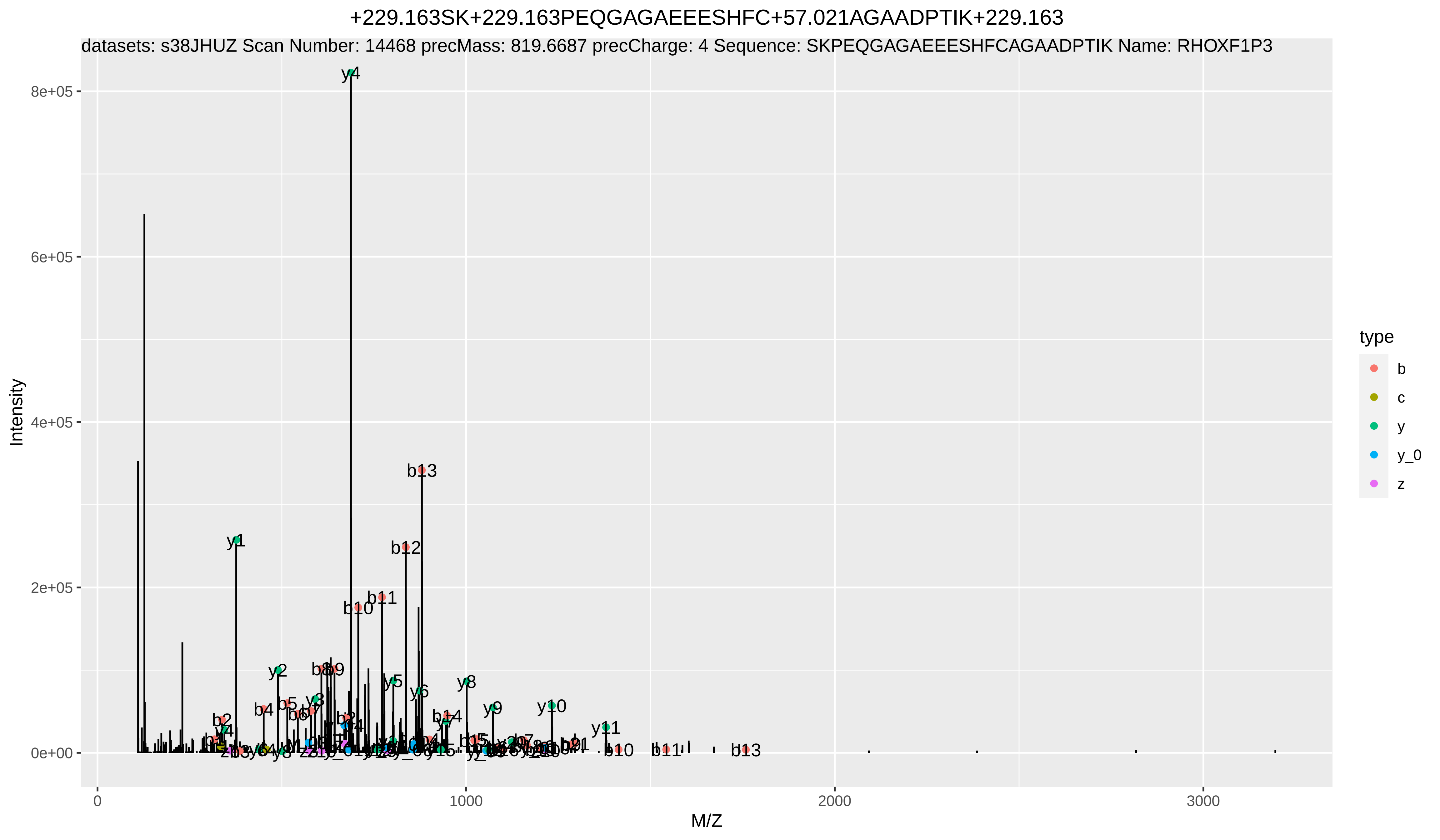
+229.163SK+229.163PEQGAGAEEESHFC+57.021AGAADPTIK+229.163
datasets: s38JHUZ Scan Number: 14468 precMass: 819.6687 precCharge: 4 Sequence: SKPEQGAGAEEESHFCAGAADPTIK Name: RHOXF1P3
Intensity
8e+05
6e+05
4e+05
2e+05
0e+00
M/Z
0
1000
2000
3000
type
b
c
y
y_0
z
y4
b13
y1
b12
b11
b10
y2
b8
b9
y5
y8
y6
y3
b5
b4
b6
b7
b2
y9
y10
y11
b14
b10
b11
b13

+229.163SGHEDTGNPGLGFLFHELQEGDNAK+229.163

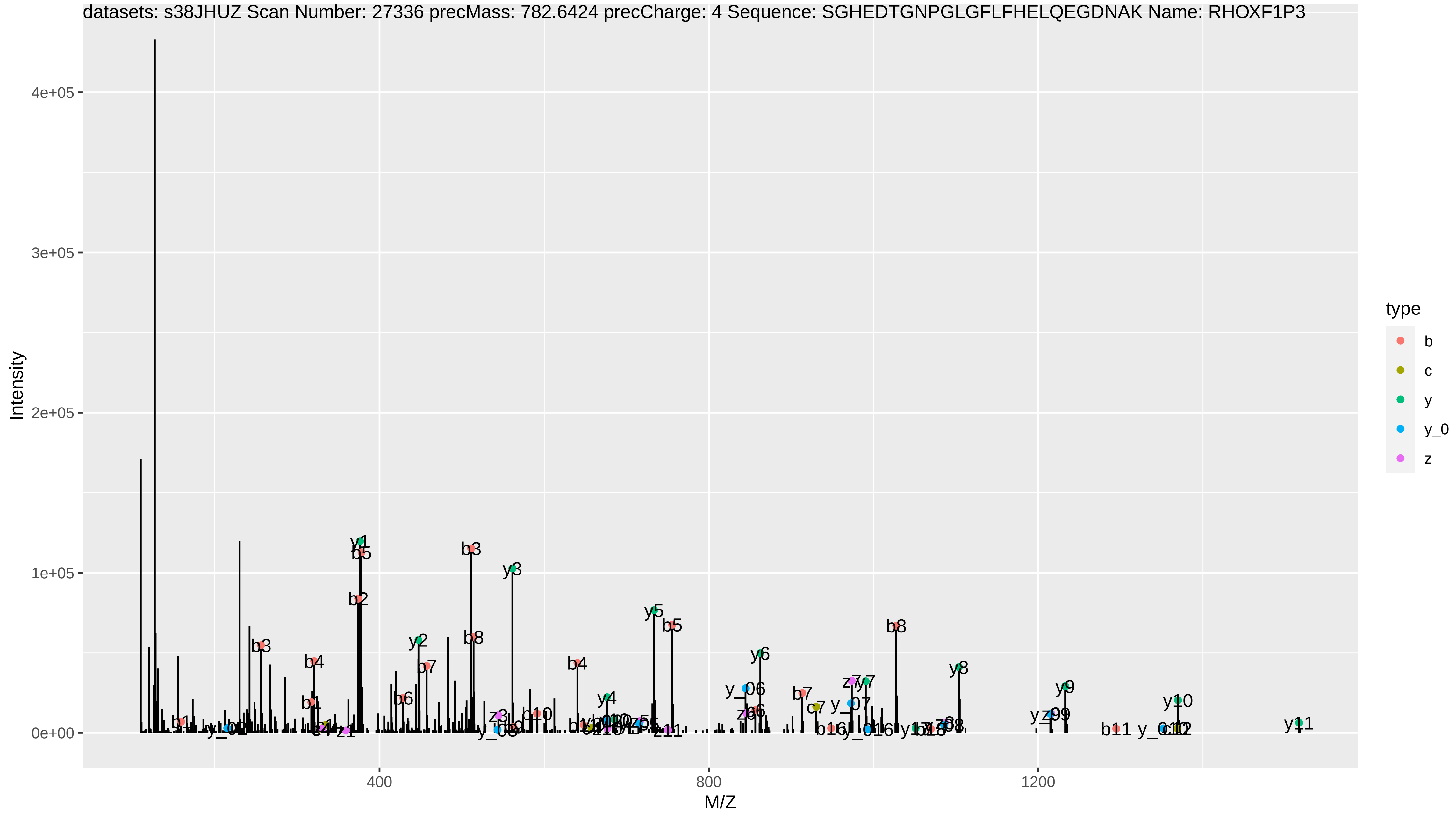

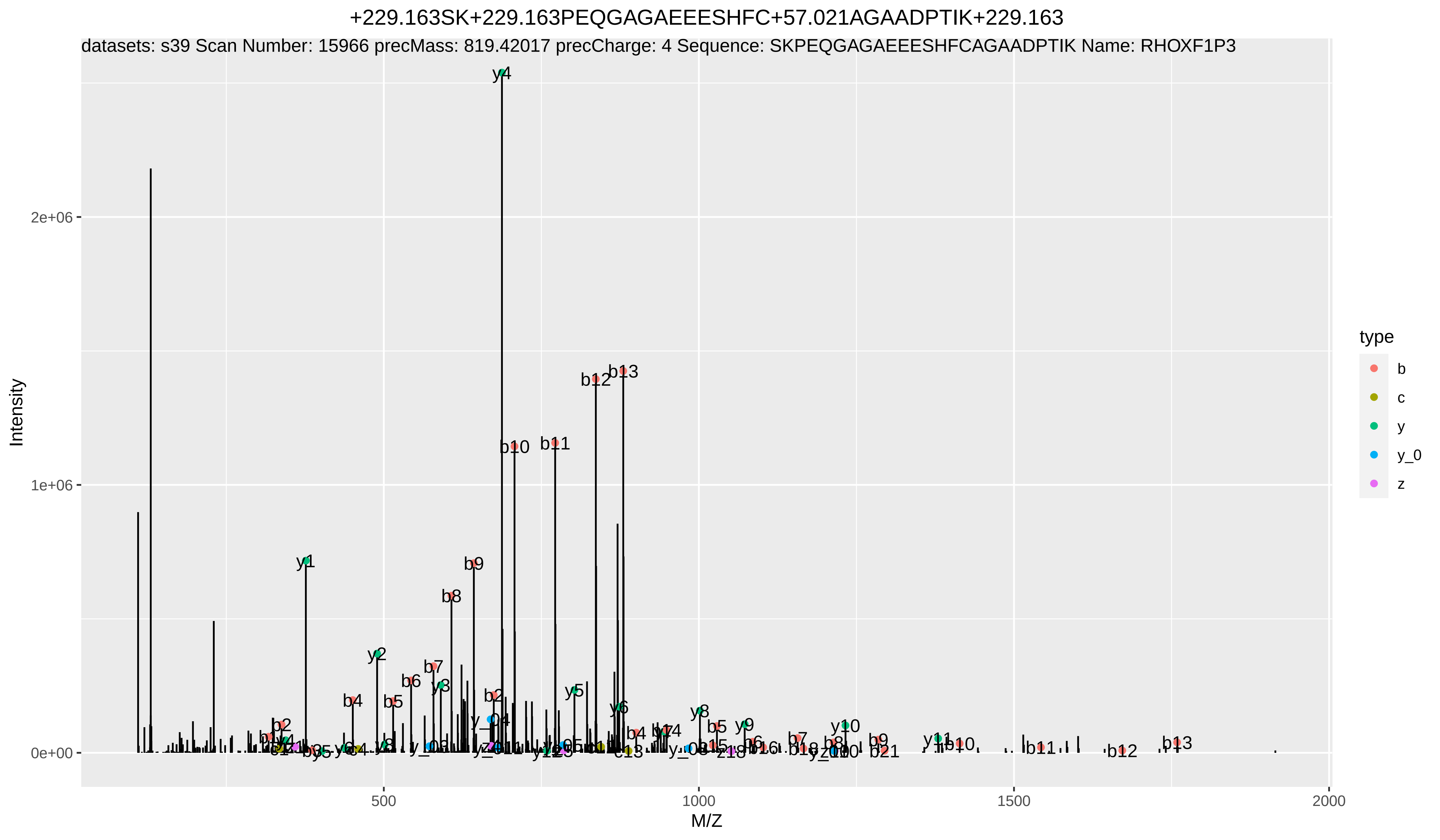

+229.163SK+229.163PEQGAGAEEESHFC+57.021AGAADPTIK+229.163
datasets: s39 Scan Number: 15966 precMass: 819.42017 precCharge: 4 Sequence: SKPEQGAGAEEESHFCAGAADPTIK Name: RHOXF1P3
Intensity
2e+06
1e+06
0e+00
M/Z
500
1000
1500
2000
type
b
c
y
y_0
z
y4
b12
b13
b10
b11
y1
b9
b8
y2
b7
b6
y3
b4
b5
b2
y5
y6
y8
y9
y10
y11
b13

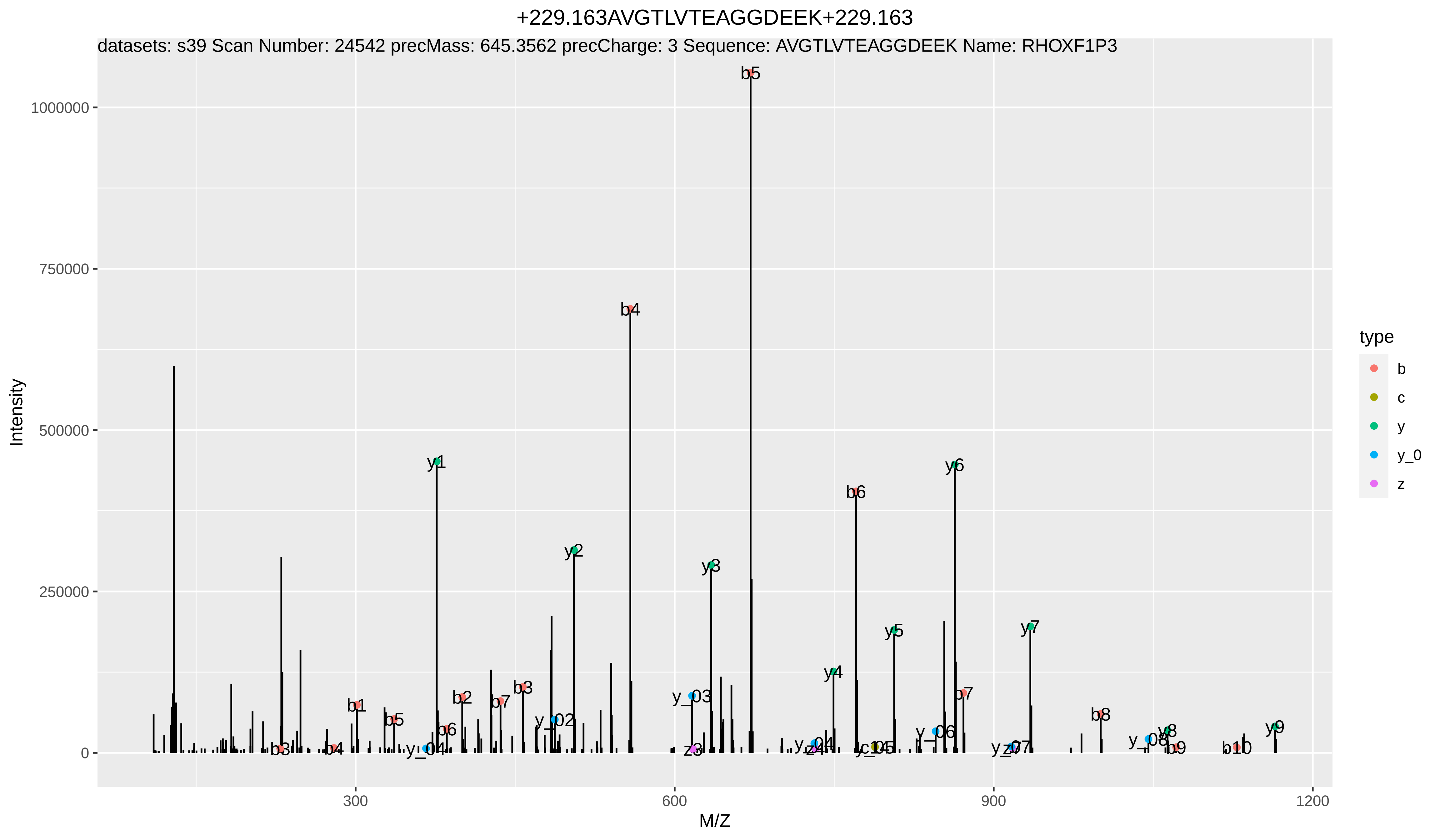

+229.163AVGTLVTEAGGDEEK+229.163
datasets: s39 Scan Number: 24542 precMass: 645.3562 precCharge: 3 Sequence: AVGTLVTEAGGDEEK Name: RHOXF1P3
Intensity
M/Z
type
b
c
y
y_0
z

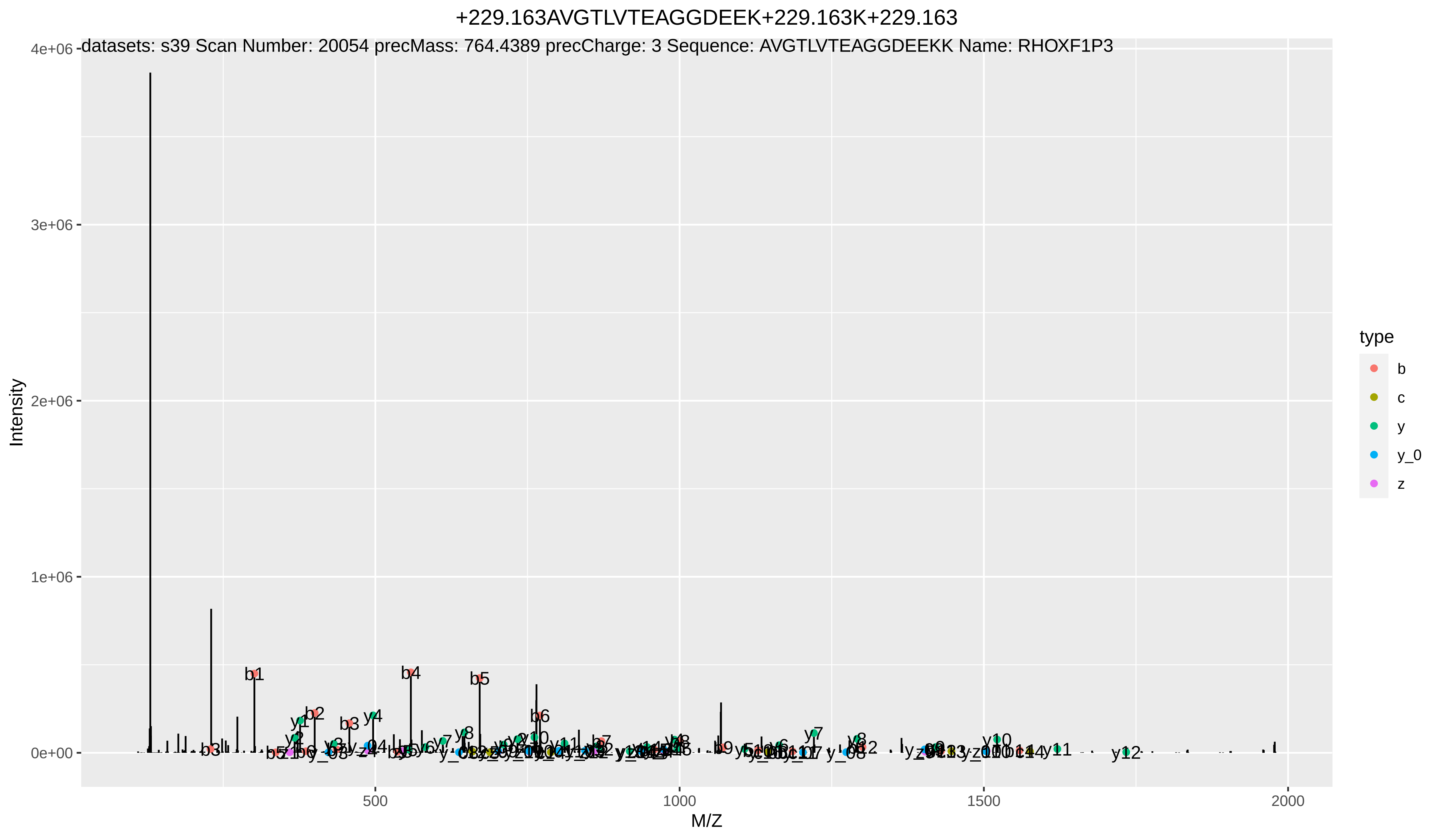
+229.163AVGTLVTEAGGDEEK+229.163K+229.163
datasets: s39 Scan Number: 20054 precMass: 764.4389 precCharge: 3 Sequence: AVGTLVTEAGGDEEKK Name: RHOXF1P3
Intensity
4e+06
3e+06
2e+06
1e+06
0e+00
M/Z
500
1000
1500
2000
type
b
c
y
y_0
z

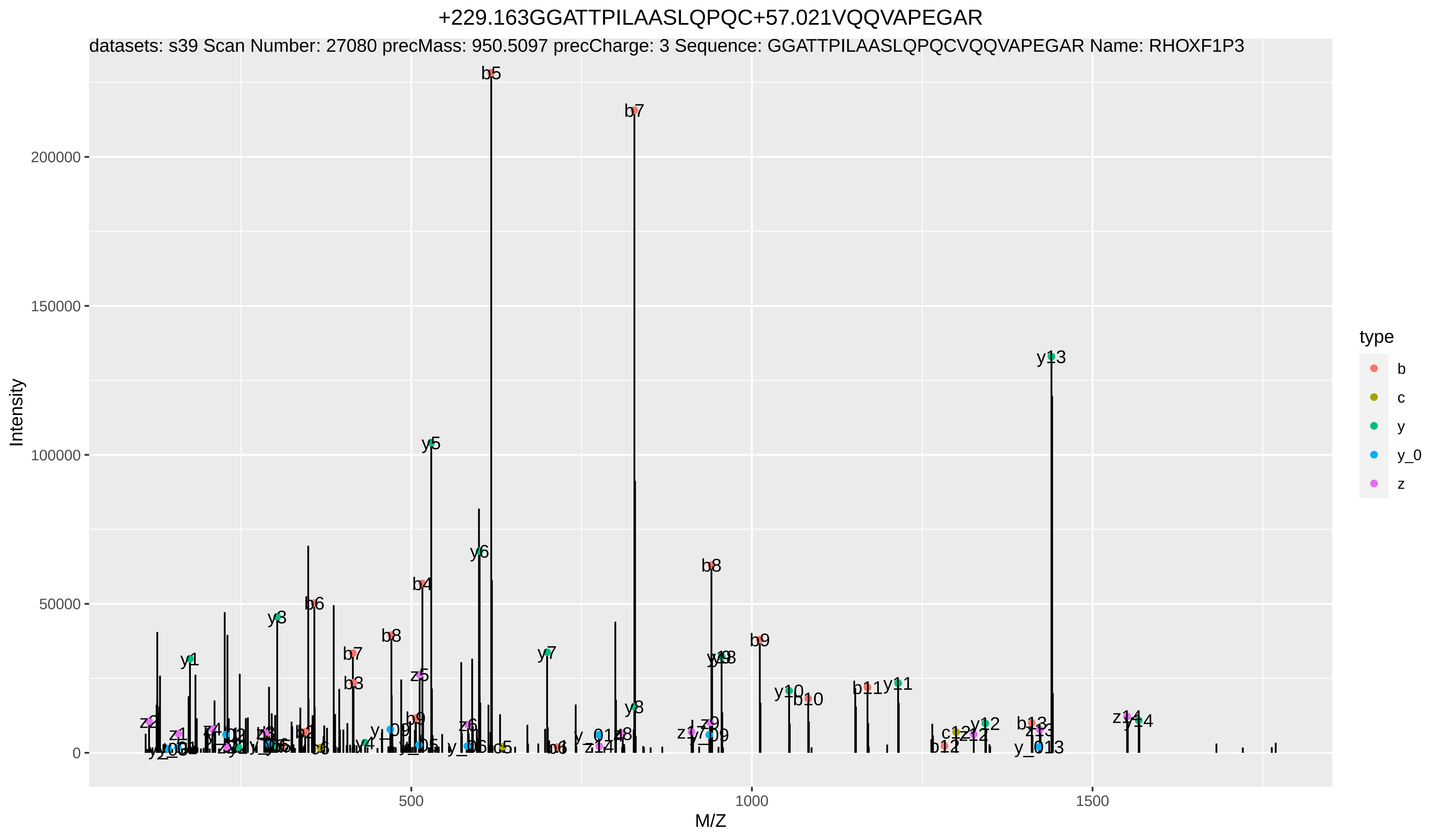

+229.163GGATTPILAASLQPQC+57.021VQQVAPEGAR
datasets: s39 Scan Number: 27080 precMass: 950.5097 precCharge: 3 Sequence: GGATTPILAASLQPQCVQQVAPEGAR Name: RHOXF1P3
Intensity
M/Z
0
50000
100000
150000
200000
500
1000
1500
type
b
c
y
y_0
z
b5
b7
y13
y5
y6
b8
b4
b6
y3
y1
y7
b9
y10
b10
b11
y11
y12
b13
z14
y14

+229.163GGGDQEPSQQQPEASSPGLLR

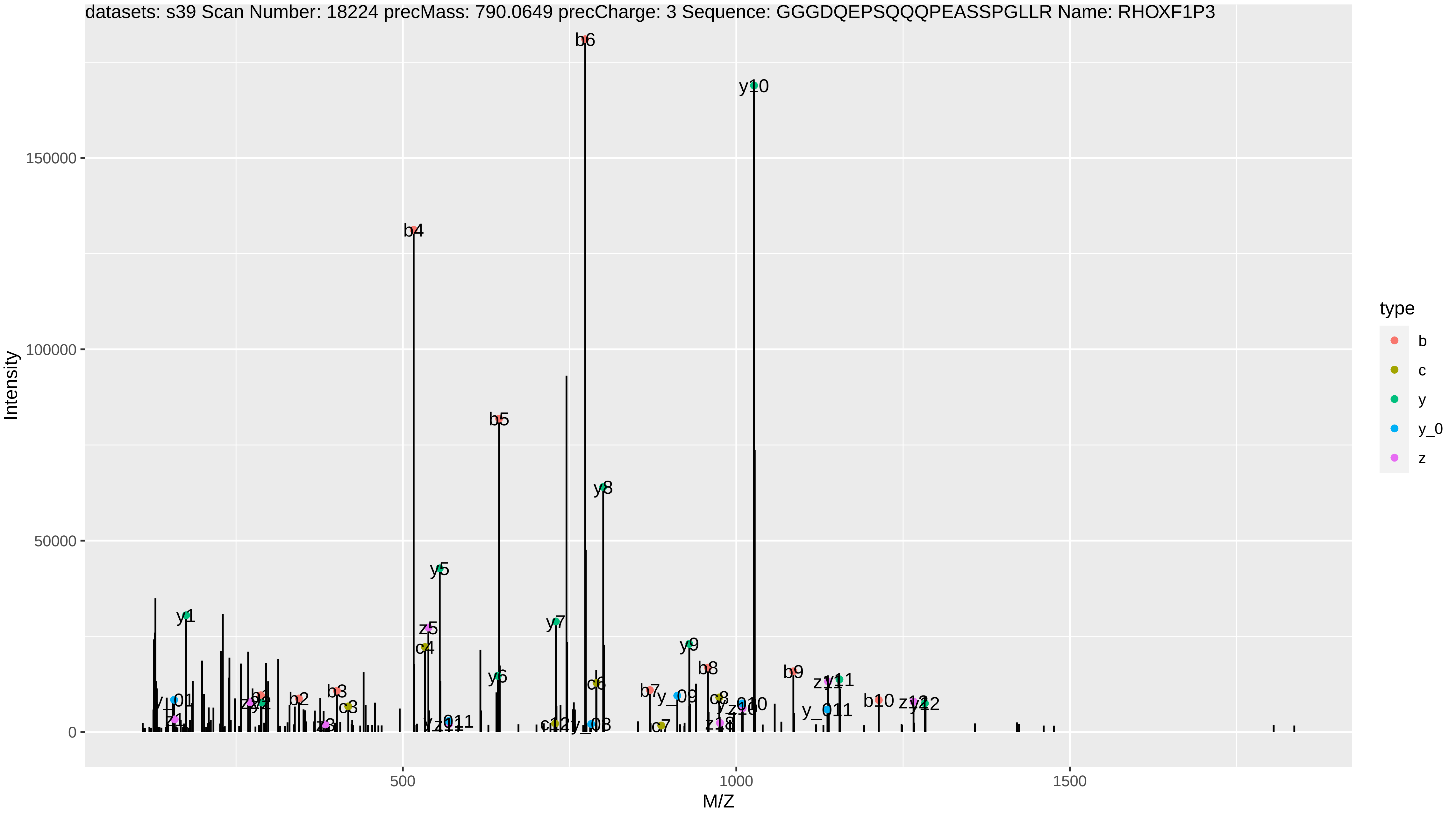

+229.163RGGGDQEPSQQQPEASSPGLLR

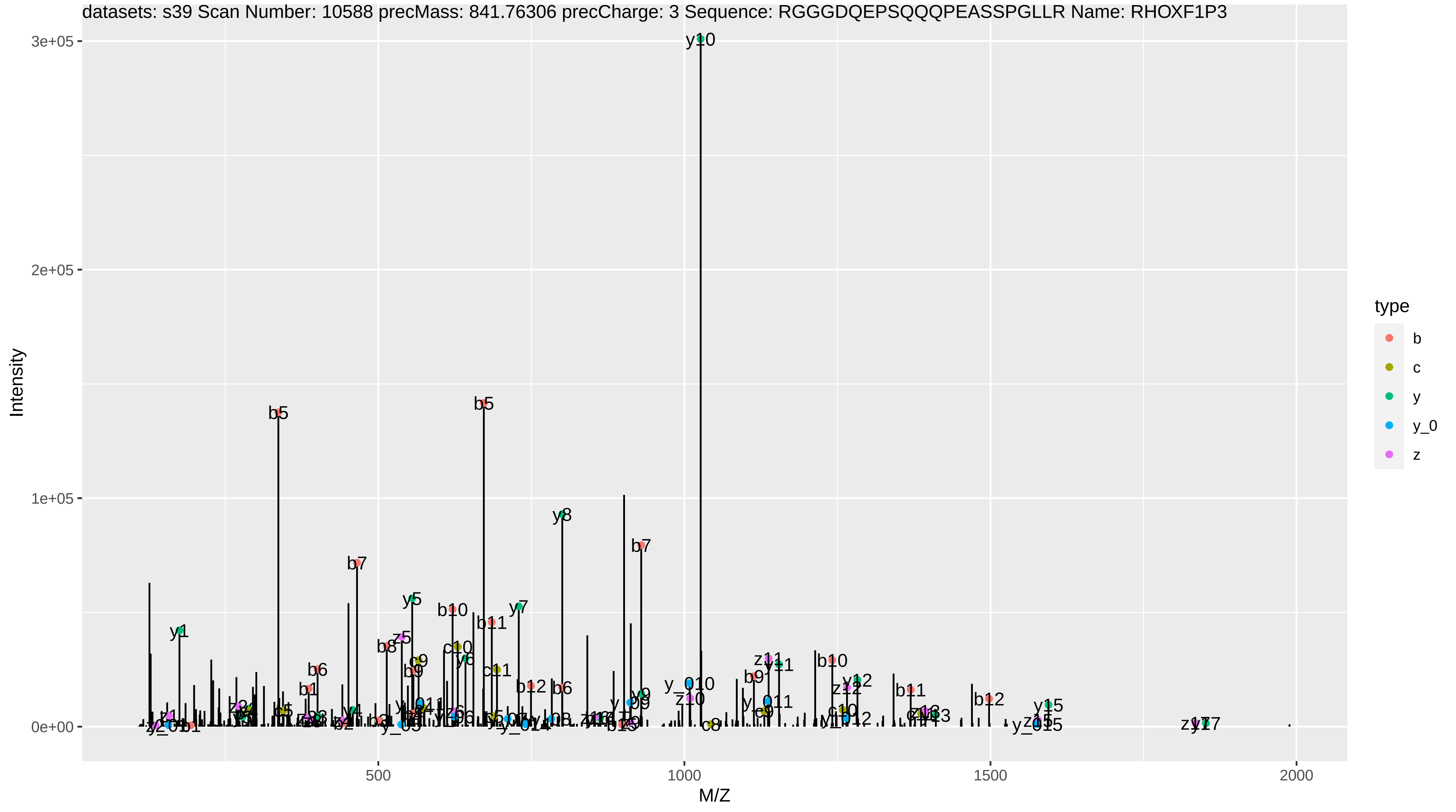

+229.163SGHEDTGNPGLGFLFHELQEGDNAK+229.163

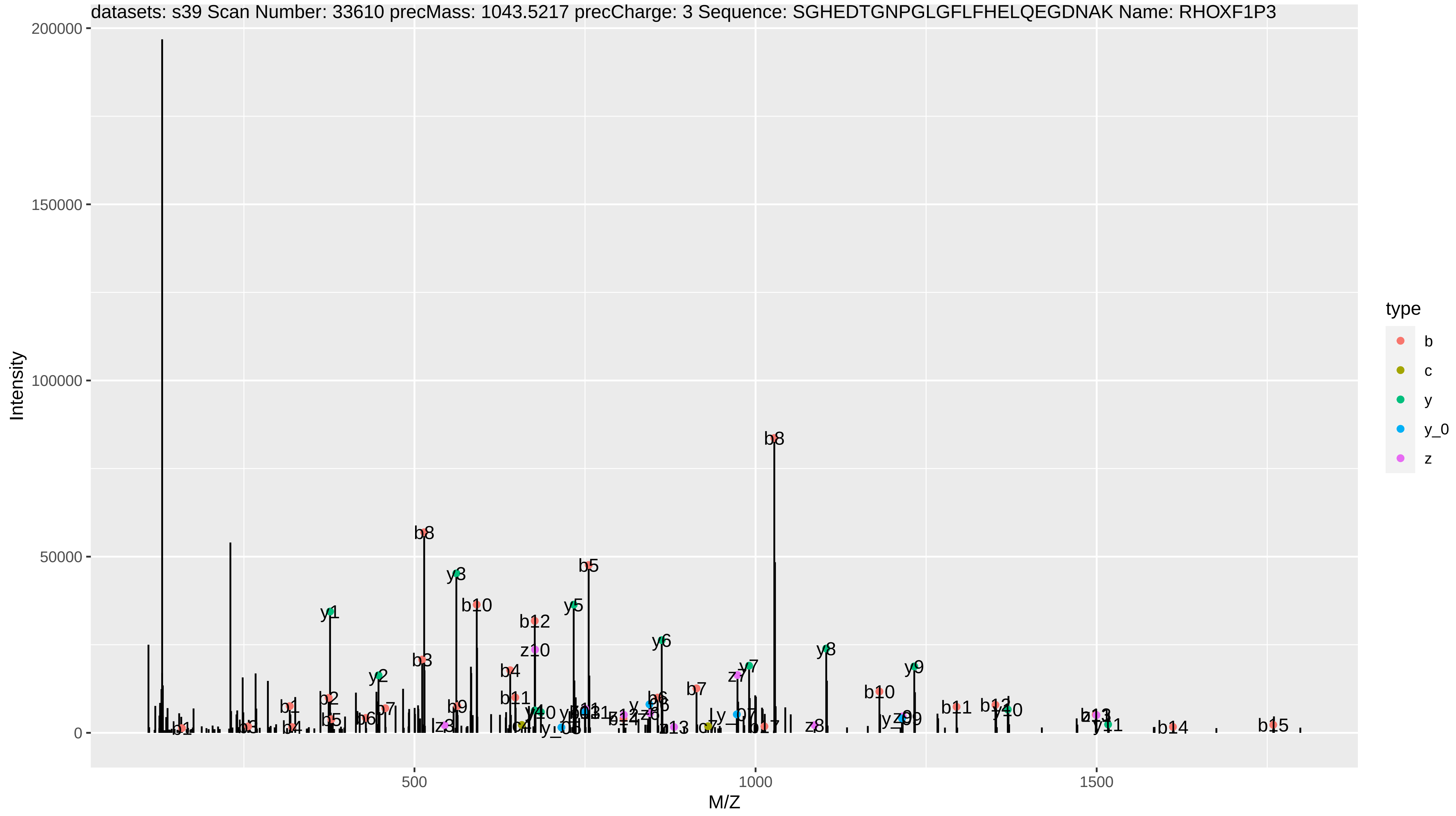

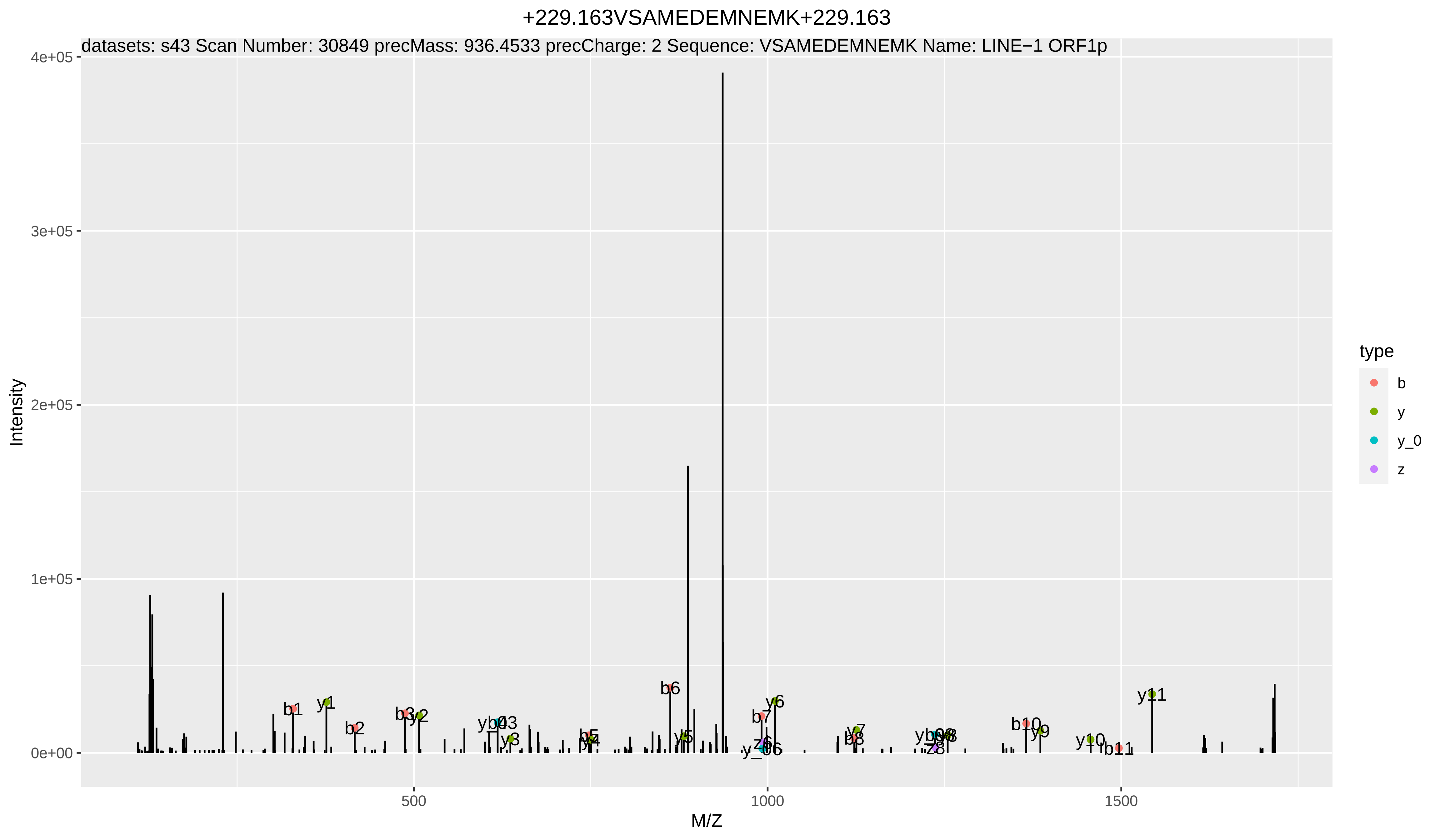

+229.163VSAMEDEMNEMK+229.163
datasets: s43 Scan Number: 30849 precMass: 936.4533 precCharge: 2 Sequence: VSAMEDEMNEMK Name: LINE−1 ORF1p
Intensity
M/Z
type
b
y
y_0
z

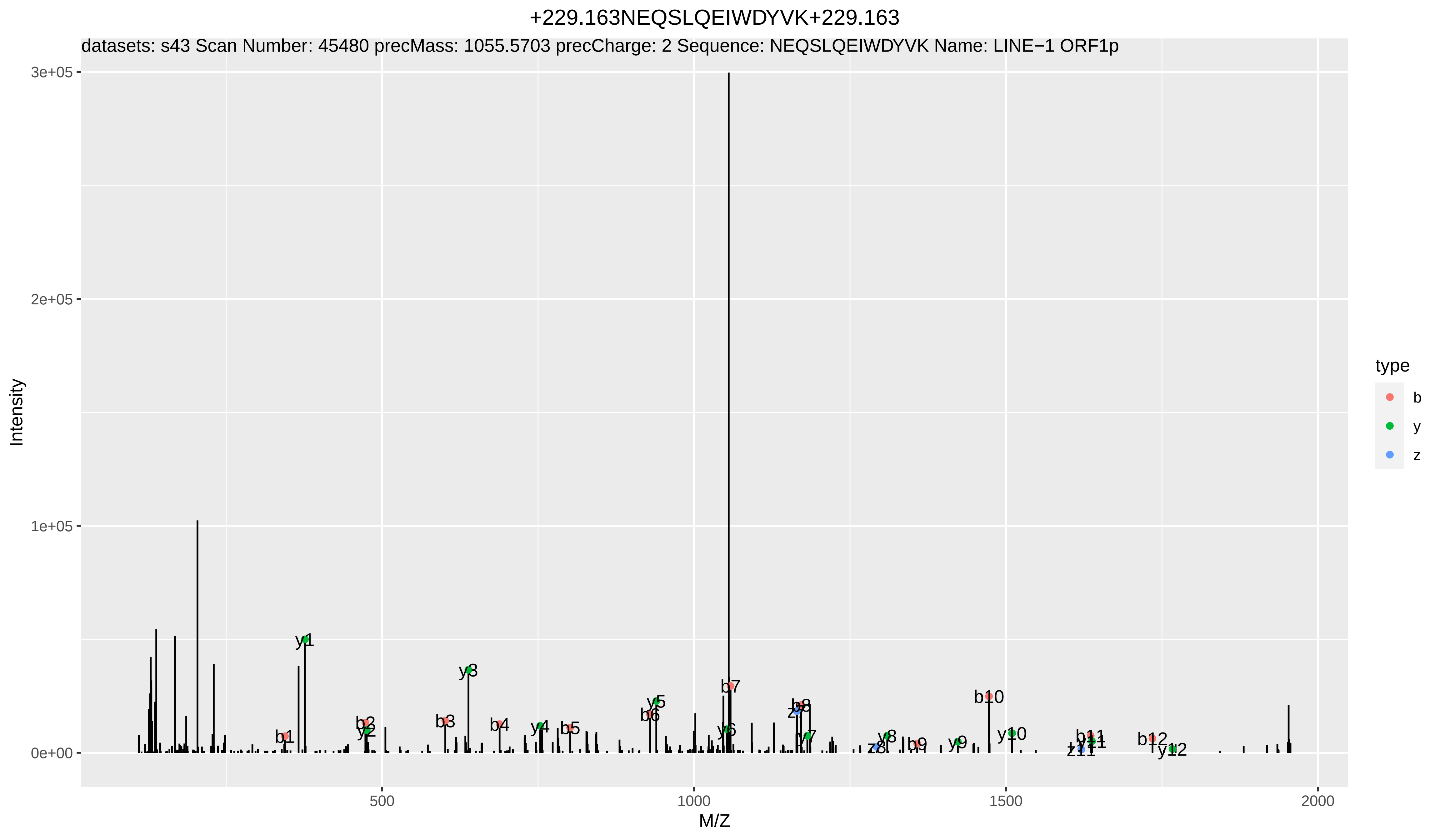
+229.163NEQSLQEIWDYVK+229.163
datasets: s43 Scan Number: 45480 precMass: 1055.5703 precCharge: 2 Sequence: NEQSLQEIWDYVK Name: LINE−1 ORF1p
Intensity
M/Z
type
b
y
z

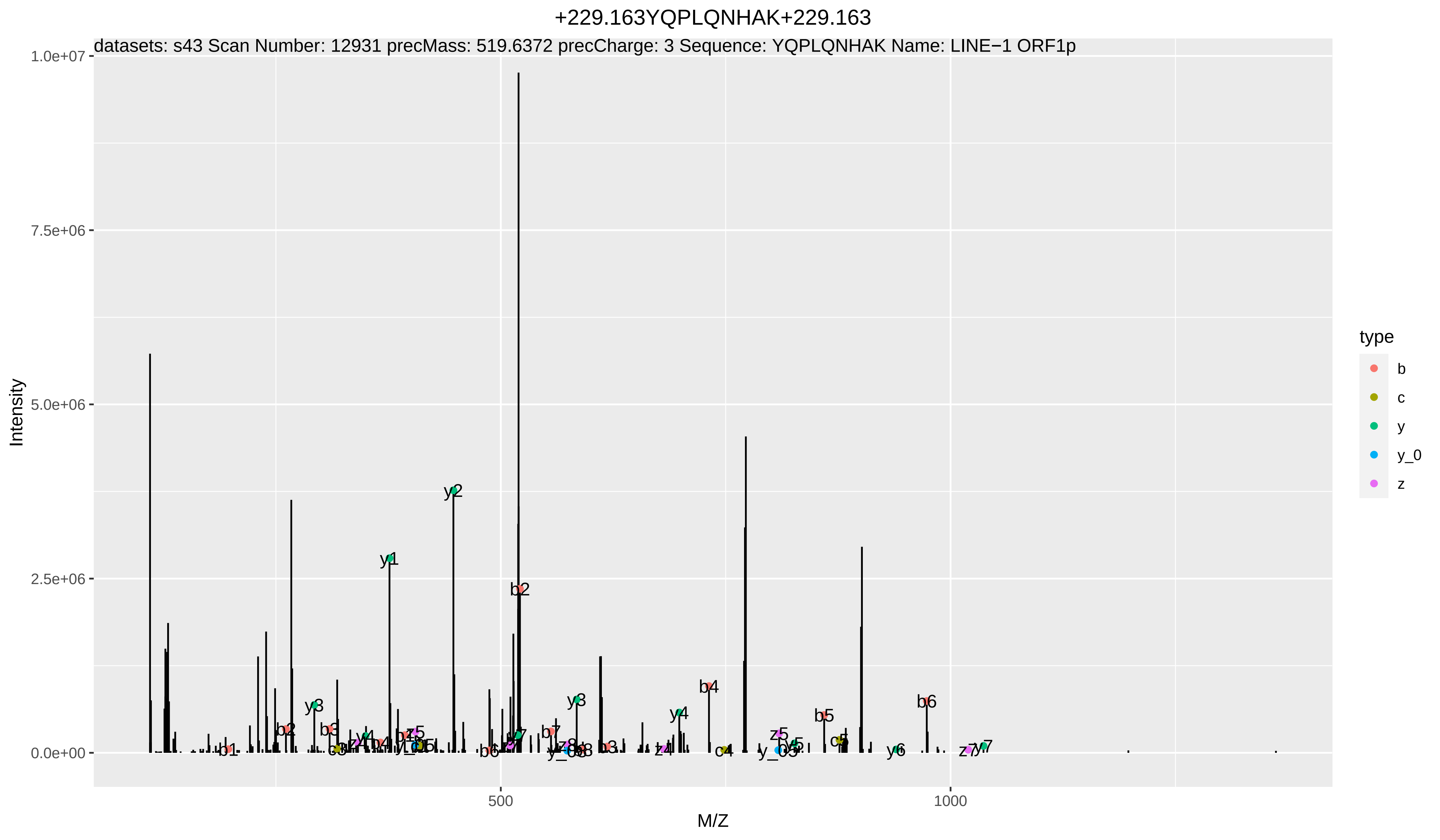
+229.163YQPLQNHAK+229.163
datasets: s43 Scan Number: 12931 precMass: 519.6372 precCharge: 3 Sequence: YQPLQNHAK Name: LINE−1 ORF1p
Intensity
M/Z
type
b
c
y
y_0
z

+229.163DFVTTRPALK+229.163

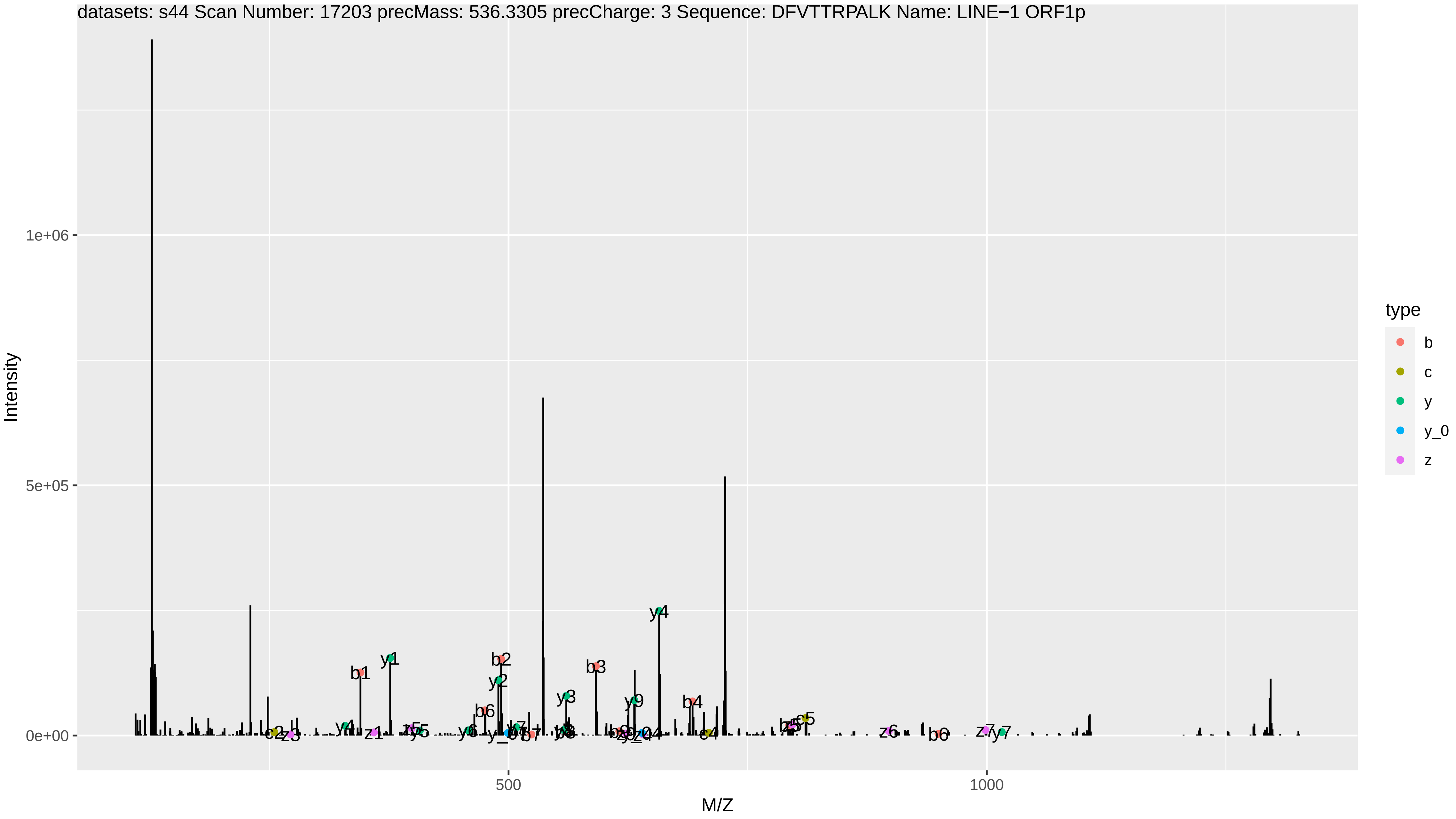

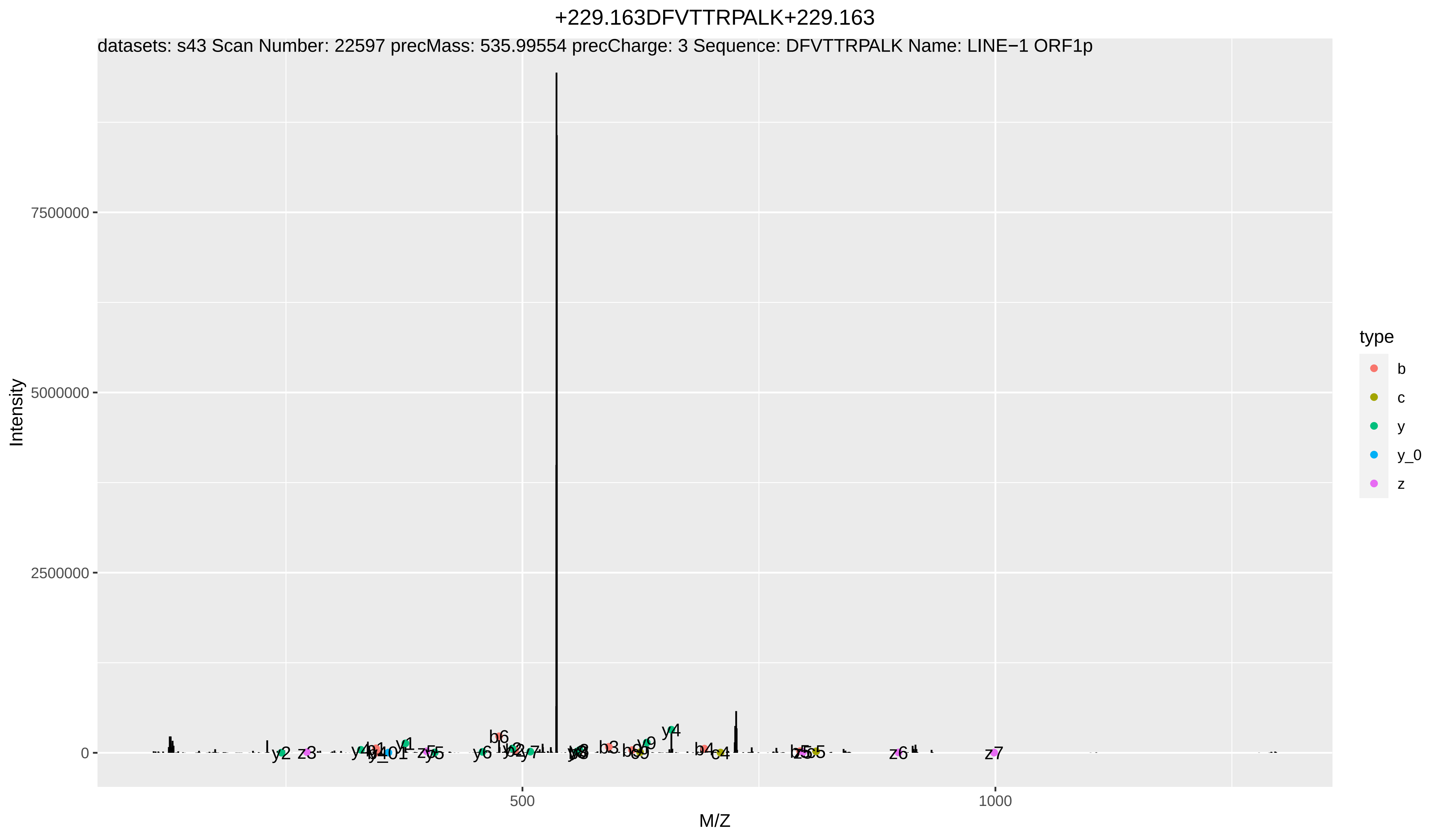
+229.163DFVTTRPALK+229.163
datasets: s43 Scan Number: 22597 precMass: 535.99554 precCharge: 3 Sequence: DFVTTRPALK Name: LINE−1 ORF1p
Intensity
7500000
5000000
2500000
0
500
1000
M/Z
type
b
c
y
y_0
z

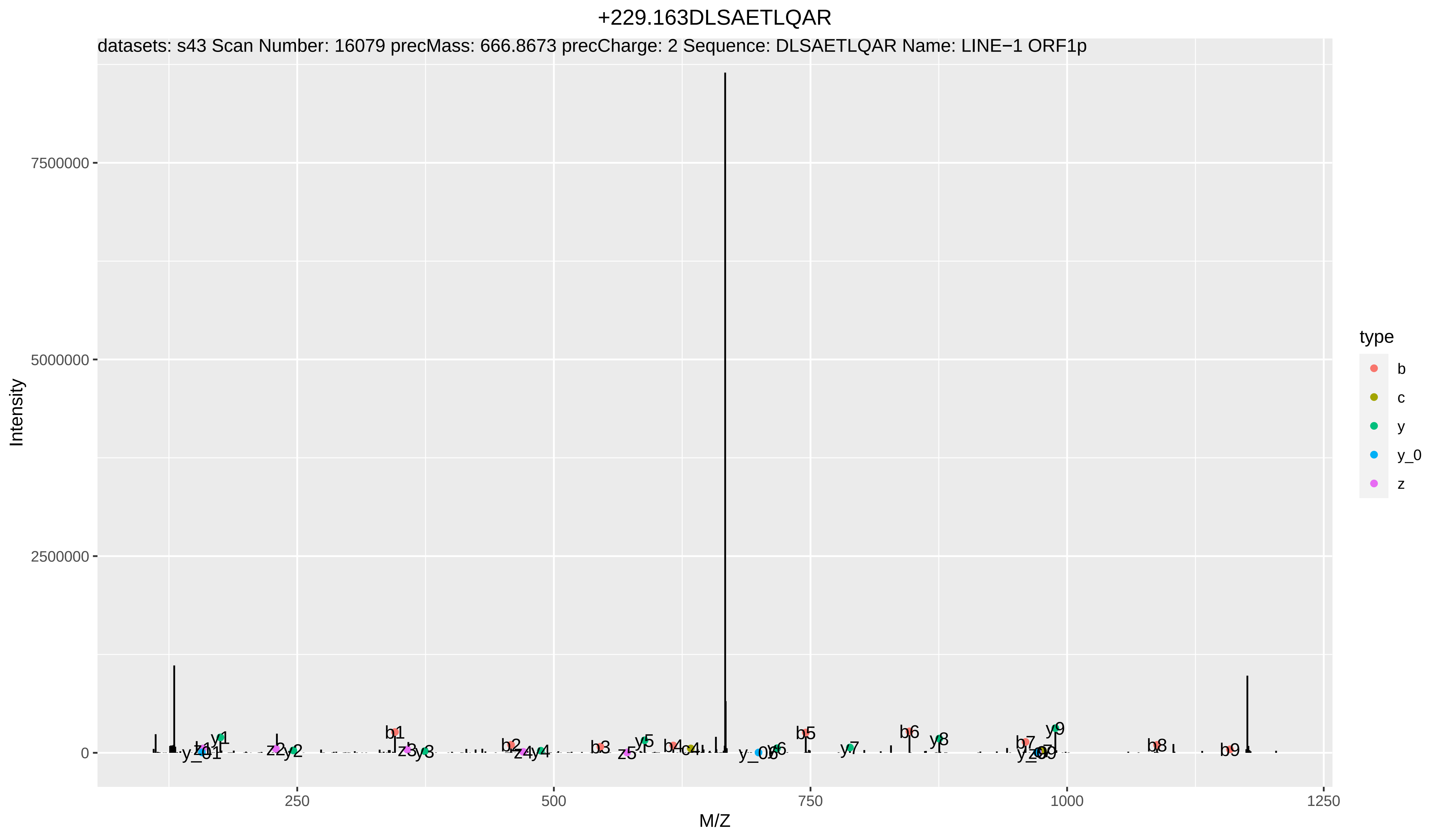
+229.163DLSAETLQAR
datasets: s43 Scan Number: 16079 precMass: 666.8673 precCharge: 2 Sequence: DLSAETLQAR Name: LINE−1 ORF1p
Intensity
M/Z
7500000
5000000
2500000
0
250
500
750
1000
1250
type
b
c
y
y_0
z

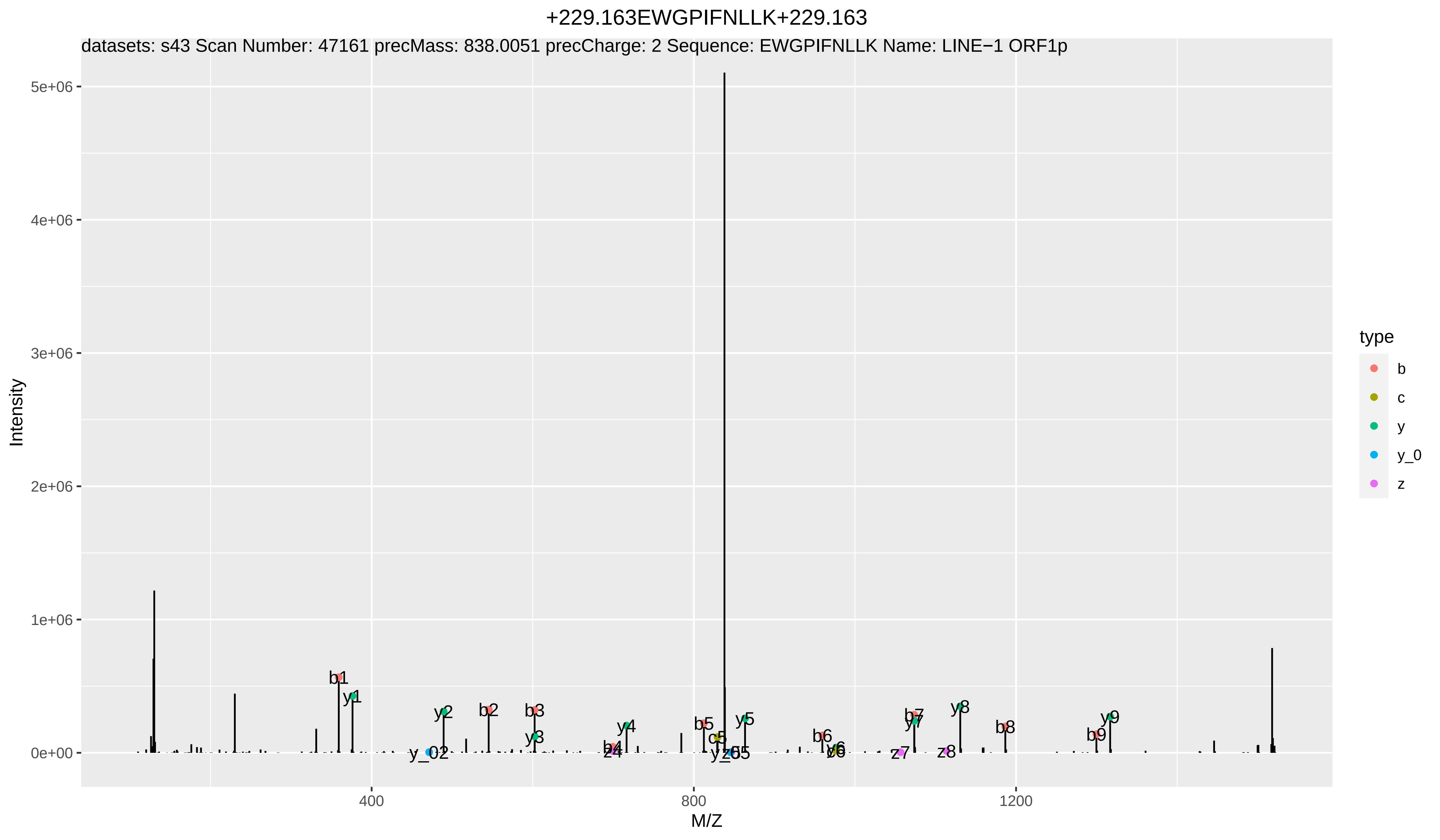
+229.163EWGPIFNLLK+229.163
datasets: s43 Scan Number: 47161 precMass: 838.0051 precCharge: 2 Sequence: EWGPIFNLLK Name: LINE−1 ORF1p
Intensity
5e+06
4e+06
3e+06
2e+06
1e+06
0e+00
M/Z
400
800
1200
type
b
c
y
y_0
z
b1
y1
y2
b2
b3
y3
y_02
y4
z4
b5
c5
y5
b6
b7
y7
y8
b8
b9
y9
z7
z8

+229.163LENTLQDIIQENFPNLAR

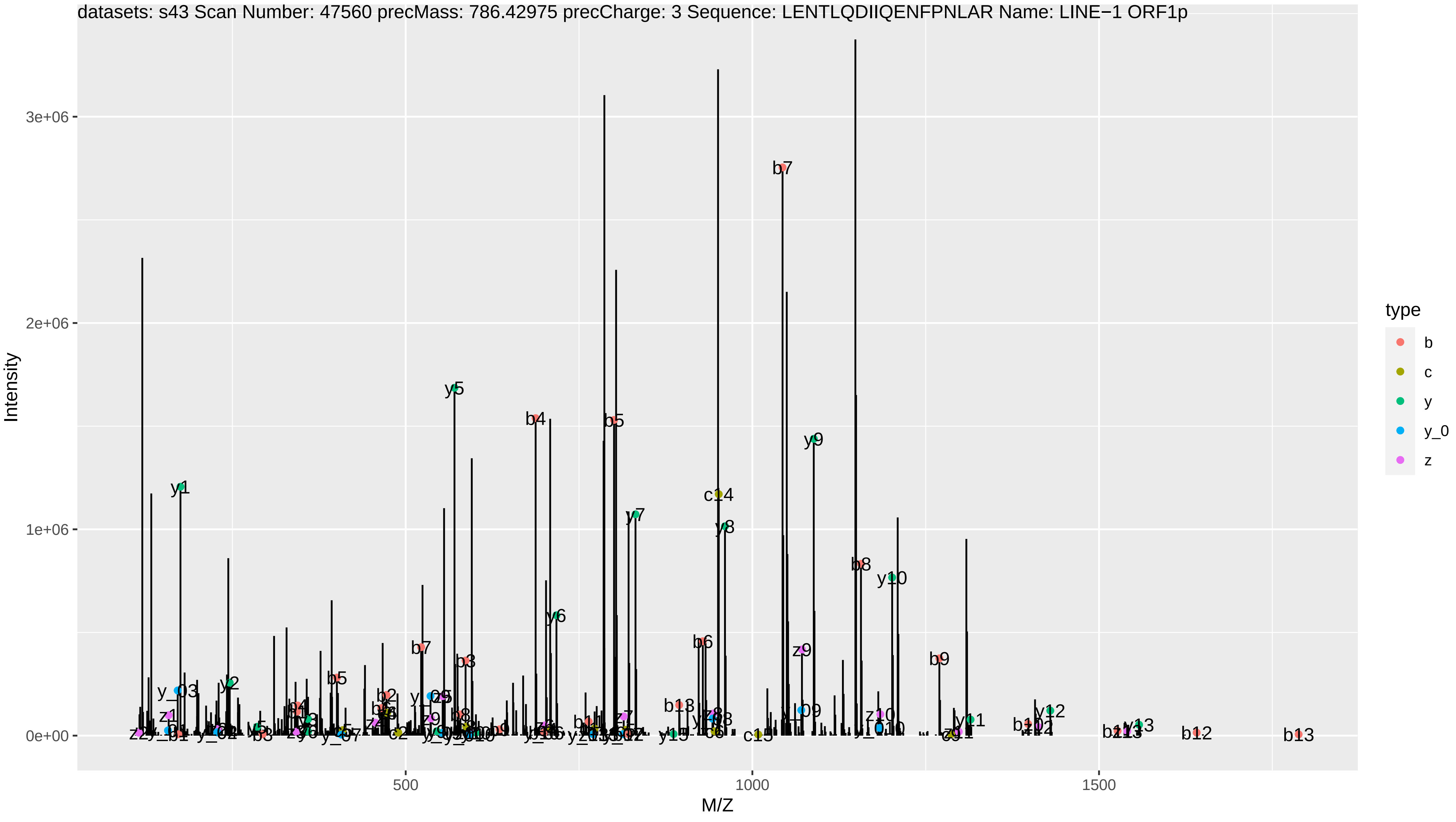

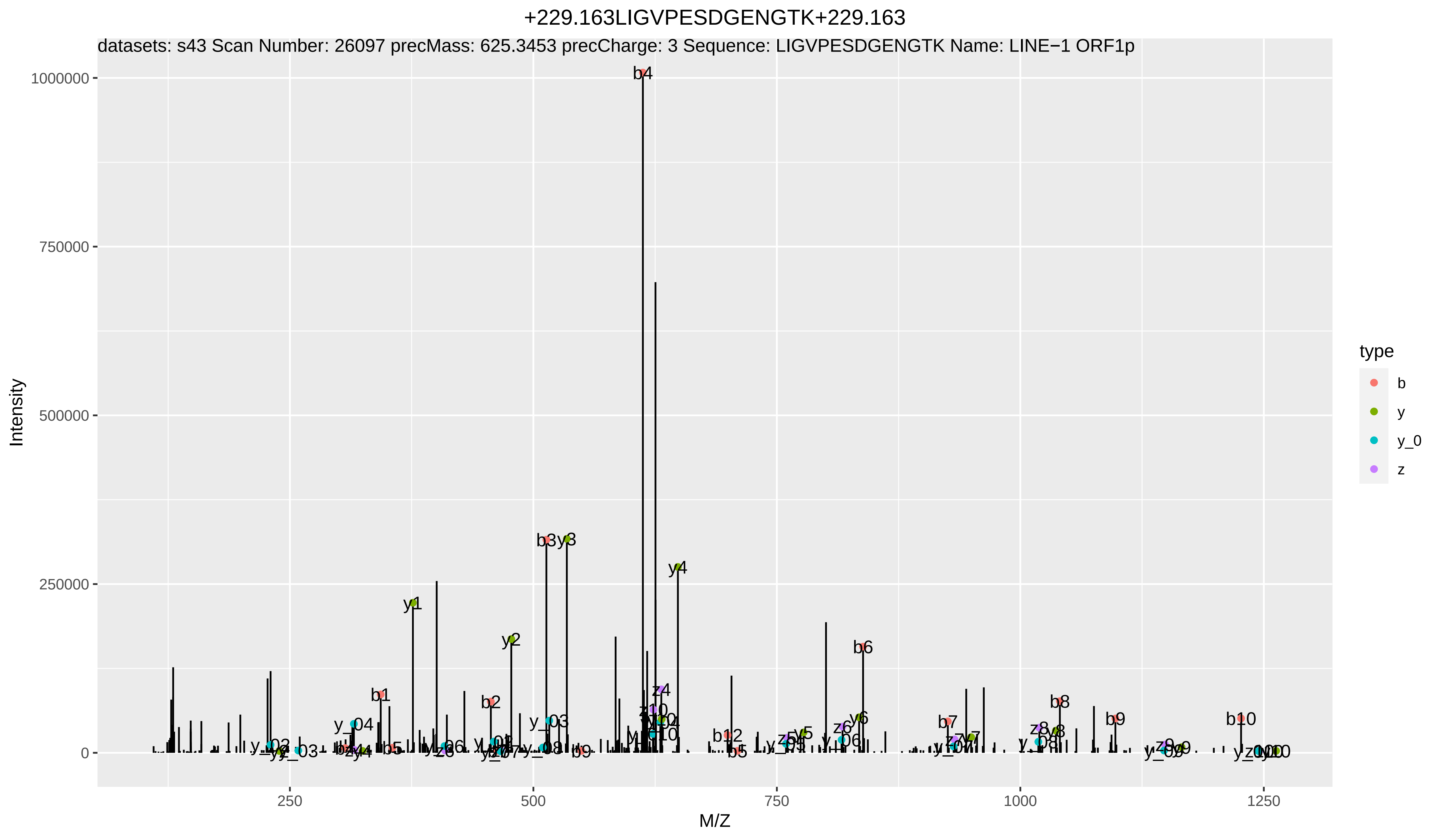
+229.163LIGVPESDGENGTK+229.163
datasets: s43 Scan Number: 26097 precMass: 625.3453 precCharge: 3 Sequence: LIGVPESDGENGTK Name: LINE−1 ORF1p
Intensity
M/Z
type
b
y
y_0
z

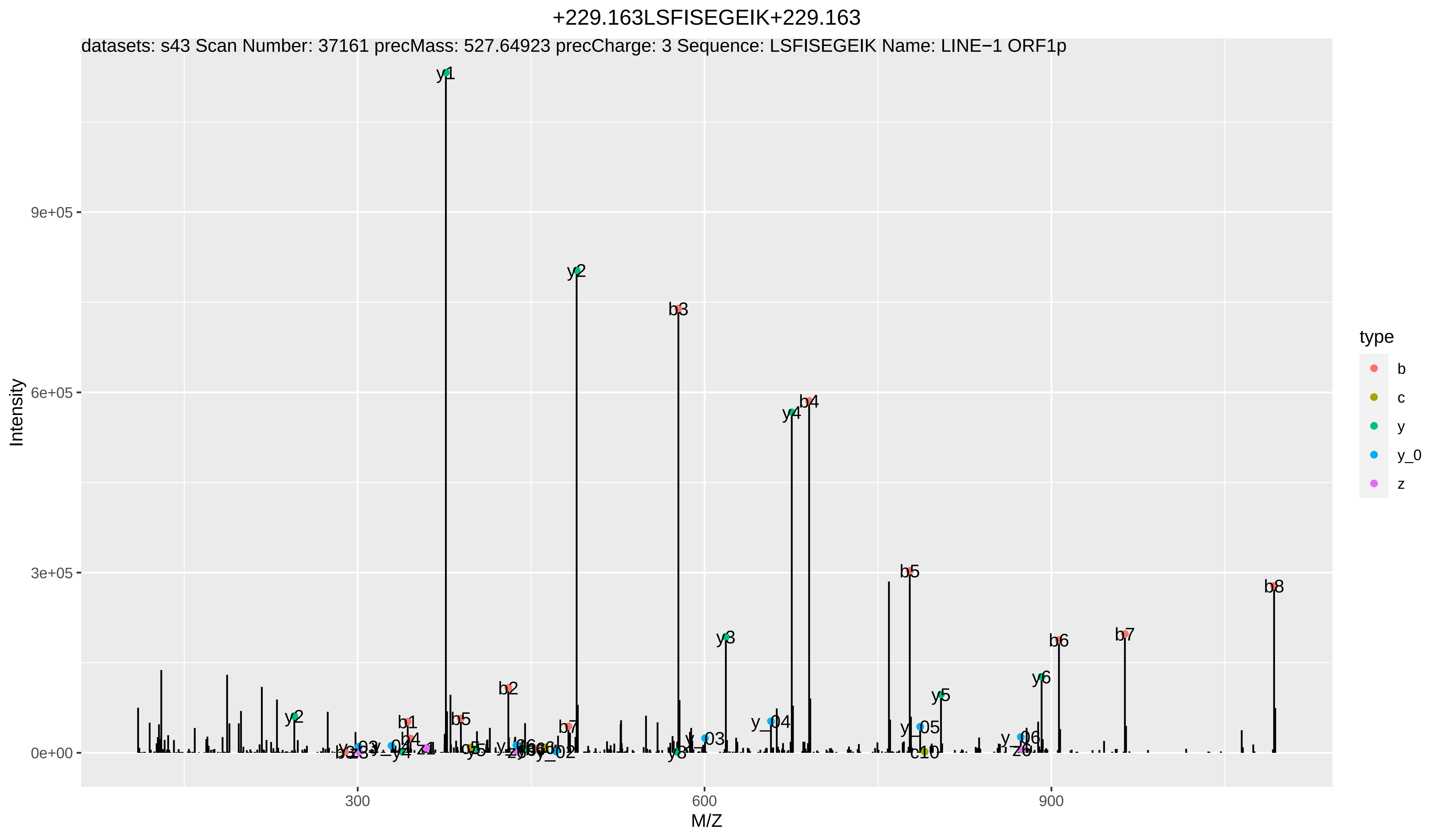
+229.163LSFISEGEIK+229.163
datasets: s43 Scan Number: 37161 precMass: 527.64923 precCharge: 3 Sequence: LSFISEGEIK Name: LINE−1 ORF1p
Intensity
9e+05
6e+05
3e+05
0e+00
M/Z
300
600
900
y1
y2
b3
y4
b4
b5
b8
y3
b6
b7
y6
y5
b2
y2
b1
b5
b7
y_04
y_05
y_06
y_03
c10
z6
y8
y5
y_02
type
b
c
y
y_0
z

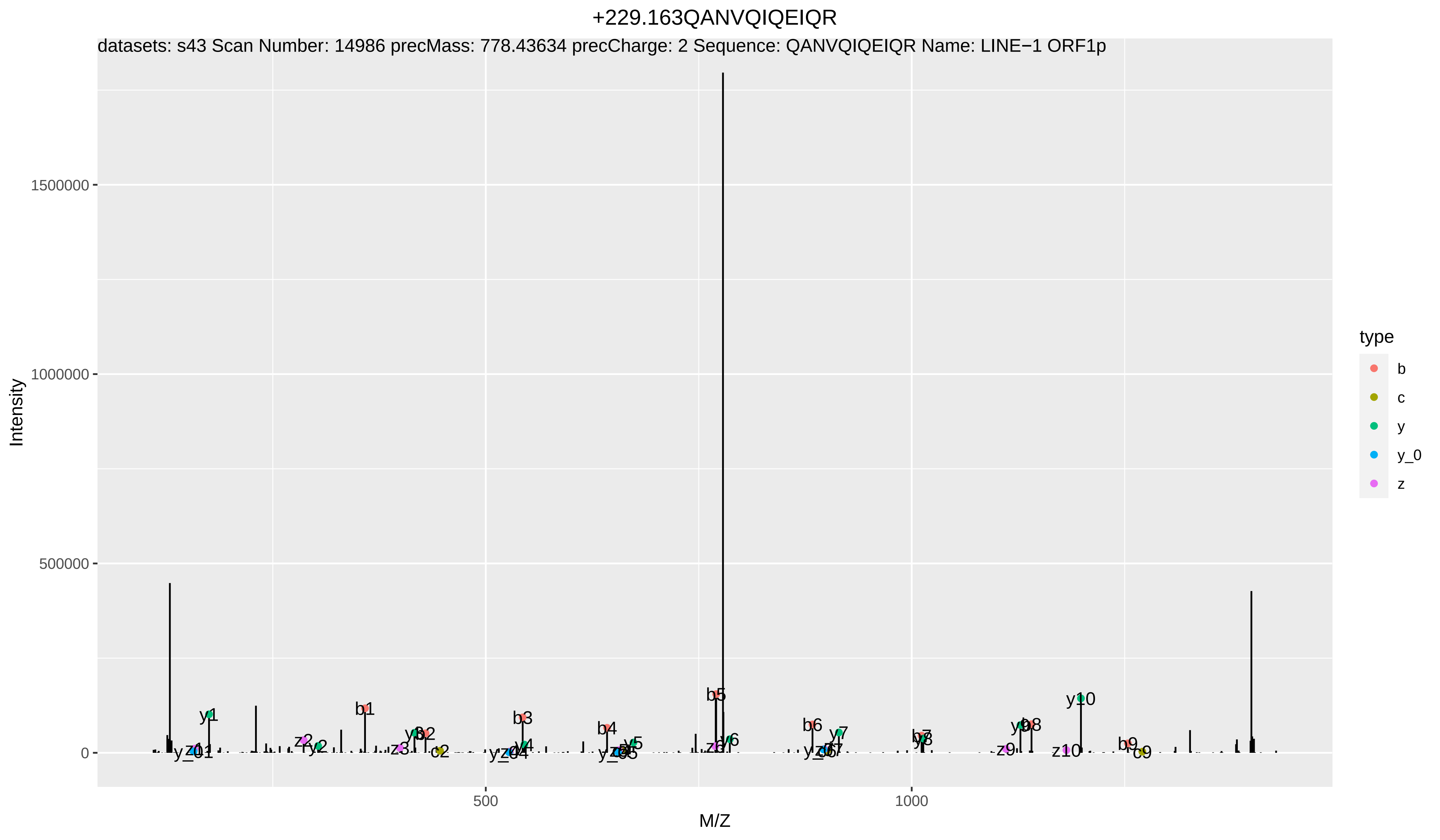
+229.163QANVQIQEIQR
datasets: s43 Scan Number: 14986 precMass: 778.43634 precCharge: 2 Sequence: QANVQIQEIQR Name: LINE−1 ORF1p
Intensity
1500000
1000000
500000
0
M/Z
500
1000
type
b
c
y
y_0
z
y1
b1
b3
b4
b5
b6
y7
y10
b9
c9
z9
z10
y4
y5
y6
z2
z3
c2

+229.163REWGPIFNLLK+229.163

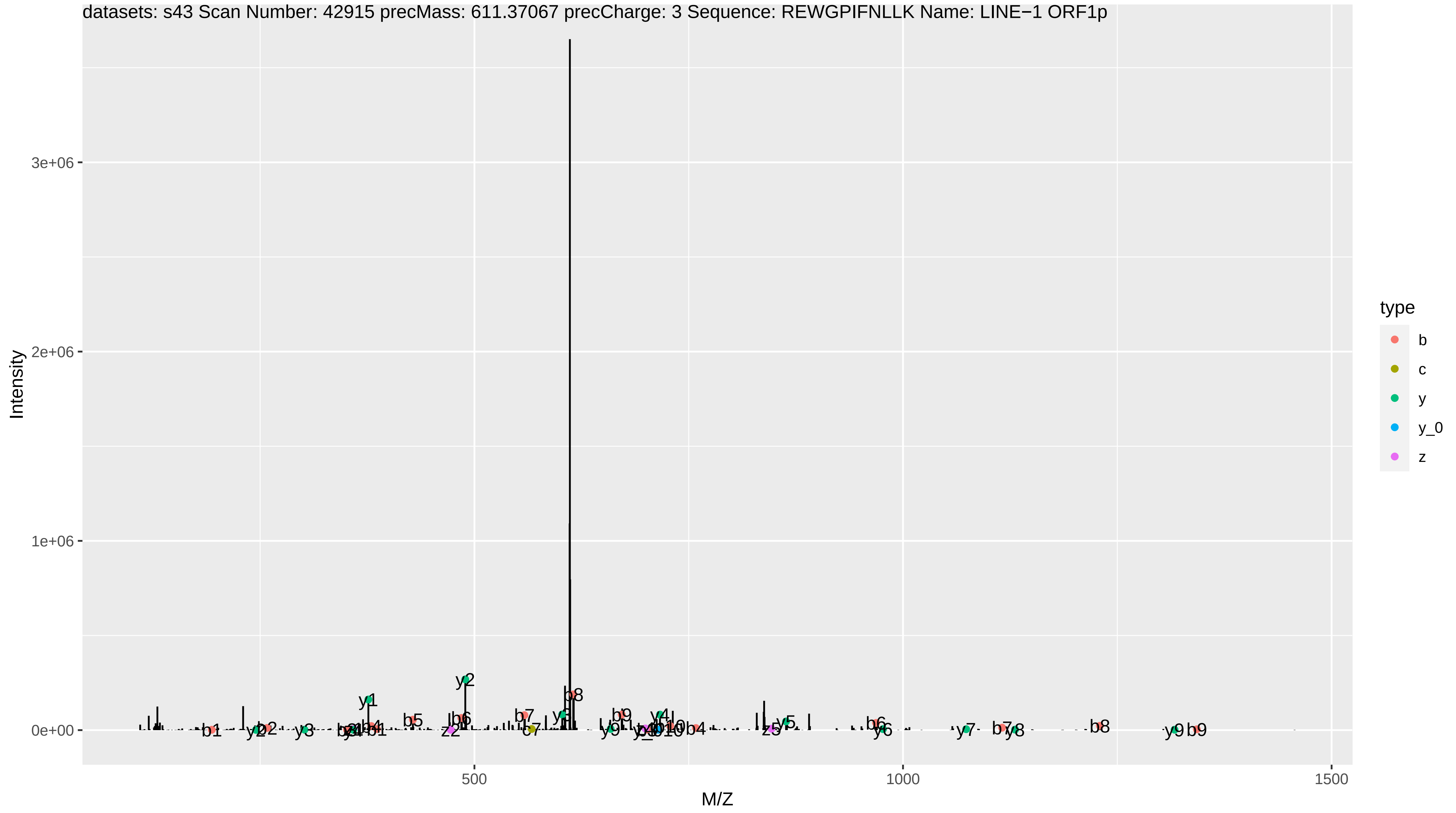

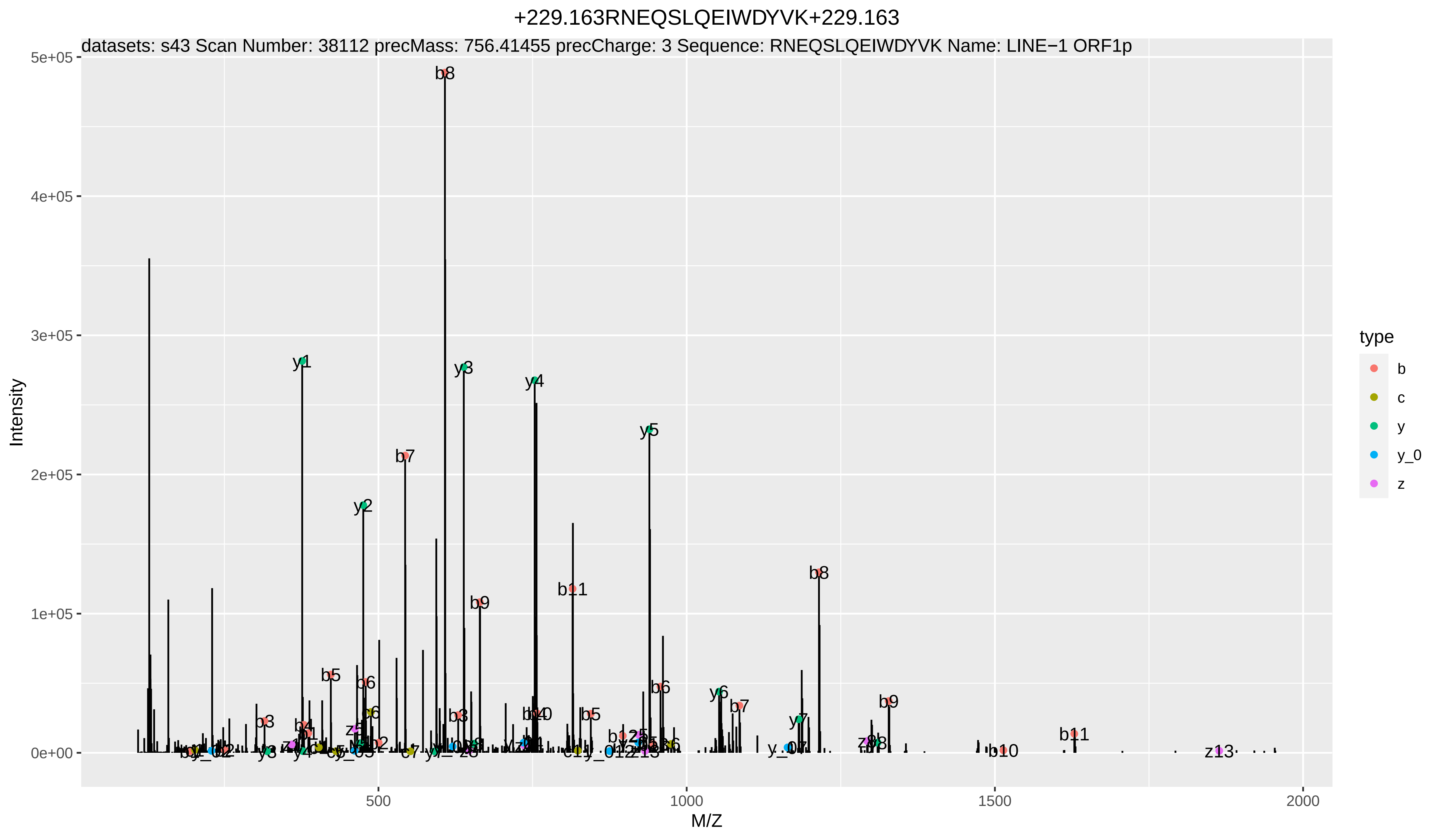
+229.163RNEQSLQEIWDYVK+229.163
datasets: s43 Scan Number: 38112 precMass: 756.41455 precCharge: 3 Sequence: RNEQSLQEIWDYVK Name: LINE−1 ORF1p
Intensity
M/Z
0e+00
1e+05
2e+05
3e+05
4e+05
5e+05
500
1000
1500
2000
type
b
c
y
y_0
z
b8
y1
y3
y4
y5
b7
y2
b11
b9
b8
b5
b6
c6
y6
b7
y7
b9
b11
b10
z13

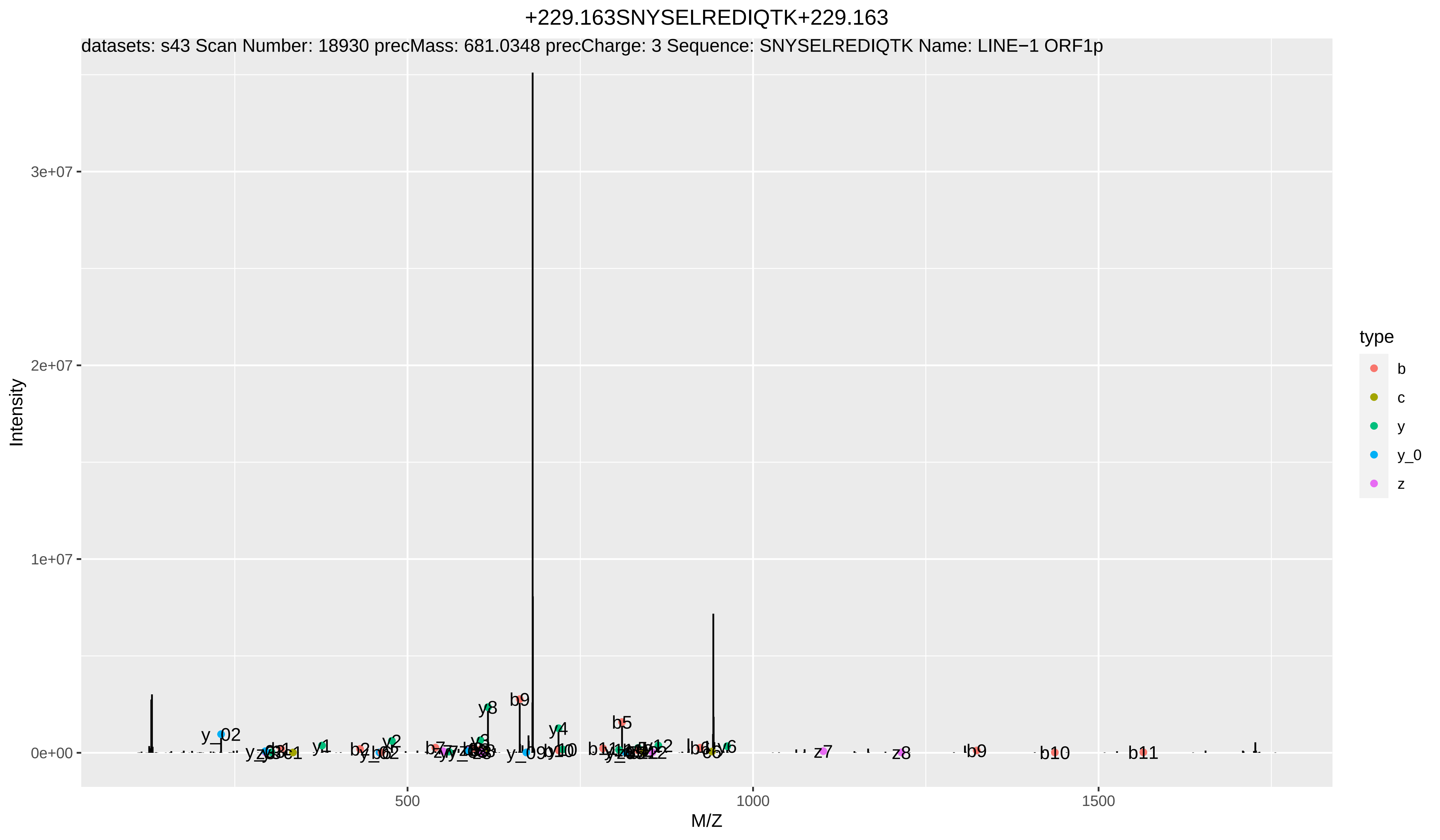
+229.163SNYSELREDIQTK+229.163
datasets: s43 Scan Number: 18930 precMass: 681.0348 precCharge: 3 Sequence: SNYSELREDIQTK Name: LINE−1 ORF1p
Intensity
3e+07
2e+07
1e+07
0e+00
M/Z
500
1000
1500
y_02
y1
b2
y2
y8
b9
y4
b5
y10
y12
y6
z7
z8
b9
b10
b11
type
b
c
y
y_0
z

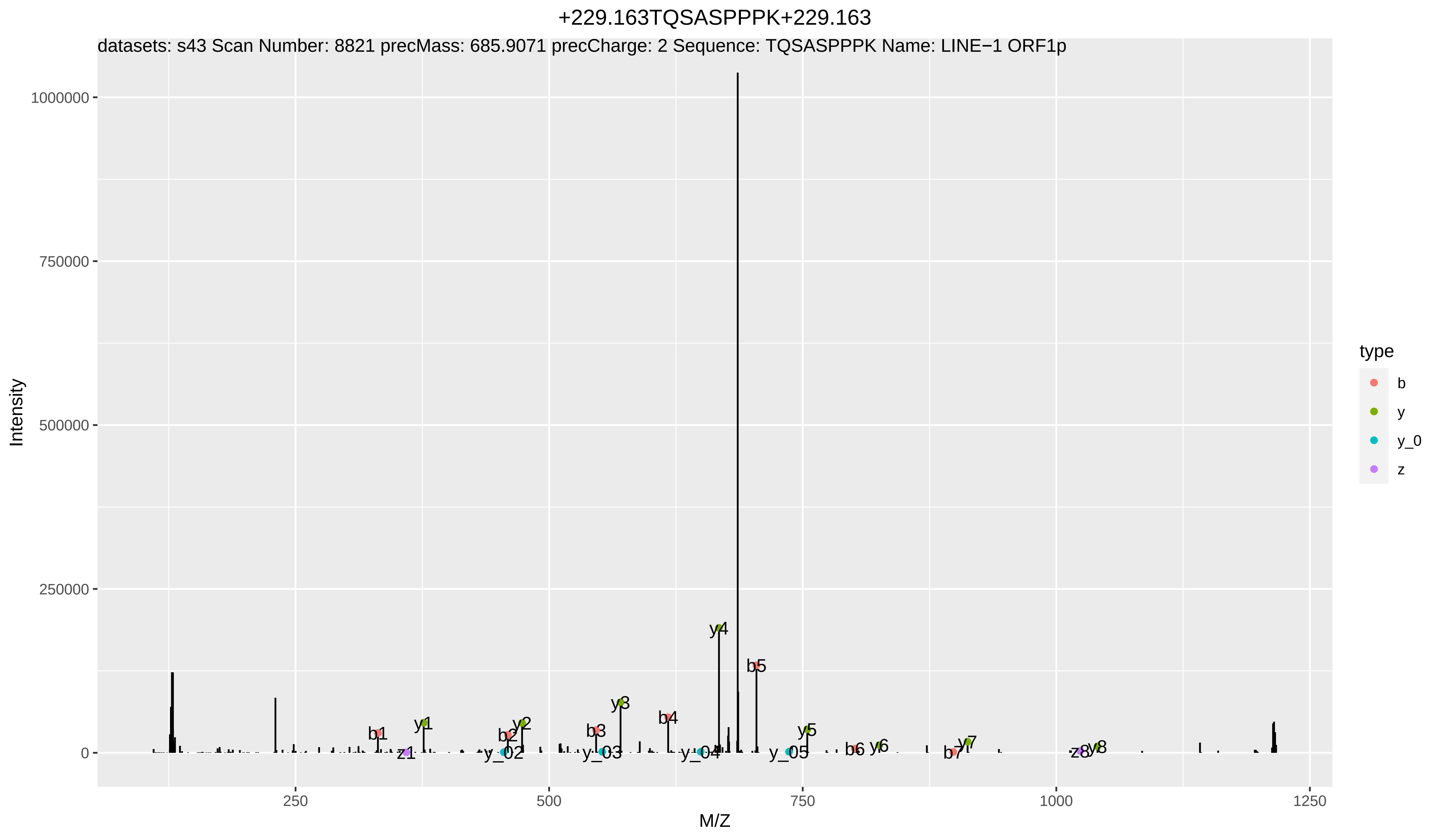

+229.163TQSASPPPK+229.163
datasets: s43 Scan Number: 8821 precMass: 685.9071 precCharge: 2 Sequence: TQSASPPPK Name: LINE−1 ORF1p
Intensity
1000000
750000
500000
250000
0
250
500
750
1000
1250
M/Z
type
b
y
y_0
z
b1
y1
z1
b2
y2
y_02
b3
y3
y_03
b4
y4
y_04
b5
y5
y_05
b6
y6
b7
y7
z8
y8

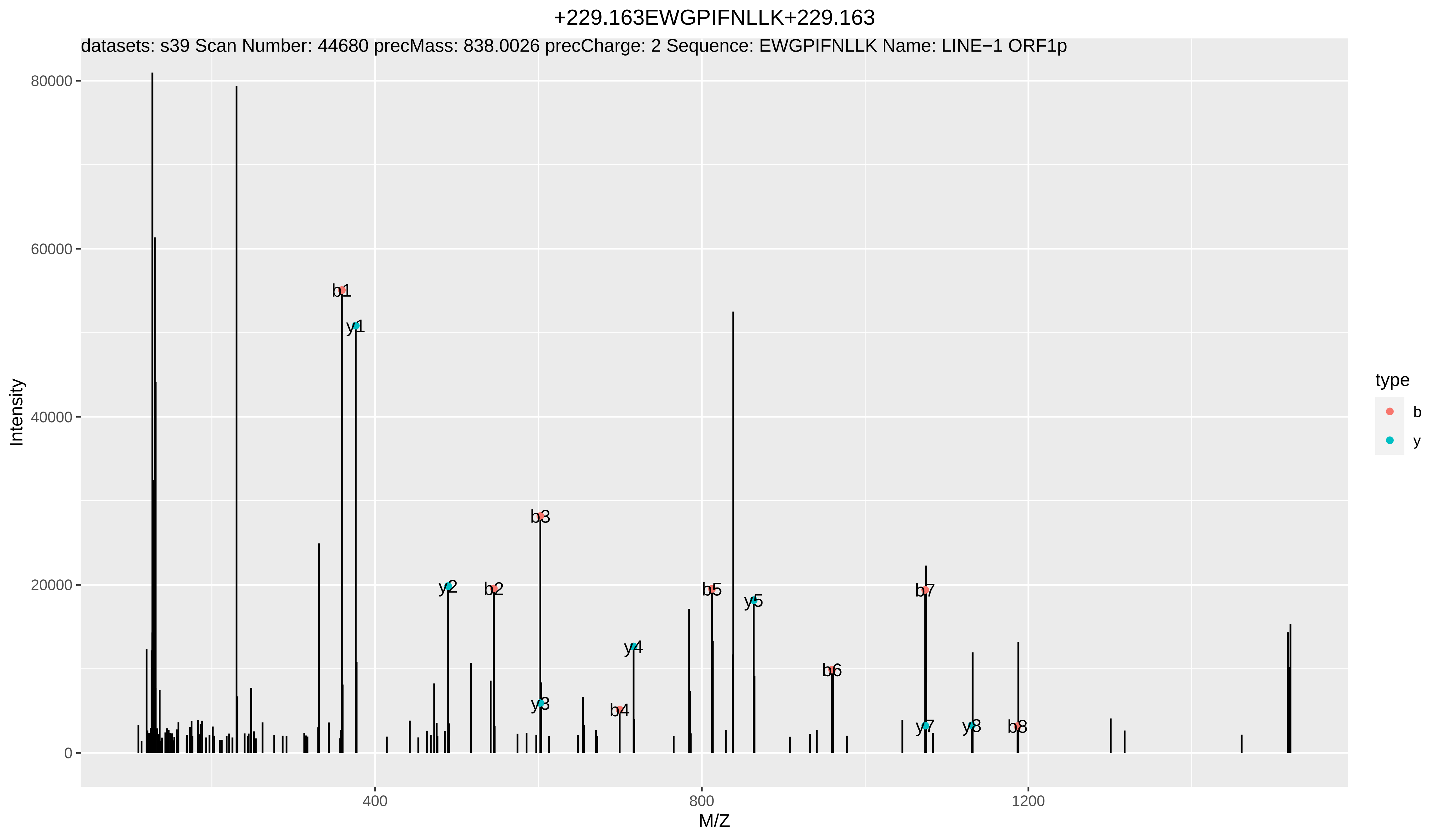

+229.163EWGPIFNLLK+229.163
datasets: s39 Scan Number: 44680 precMass: 838.0026 precCharge: 2 Sequence: EWGPIFNLLK Name: LINE−1 ORF1p
Intensity
80000
60000
40000
20000
0
M/Z
400
800
1200
b1
y1
y2
b2
b3
y3
b4
y4
b5
y5
b6
b7
y7
y8
b8
type
b
y

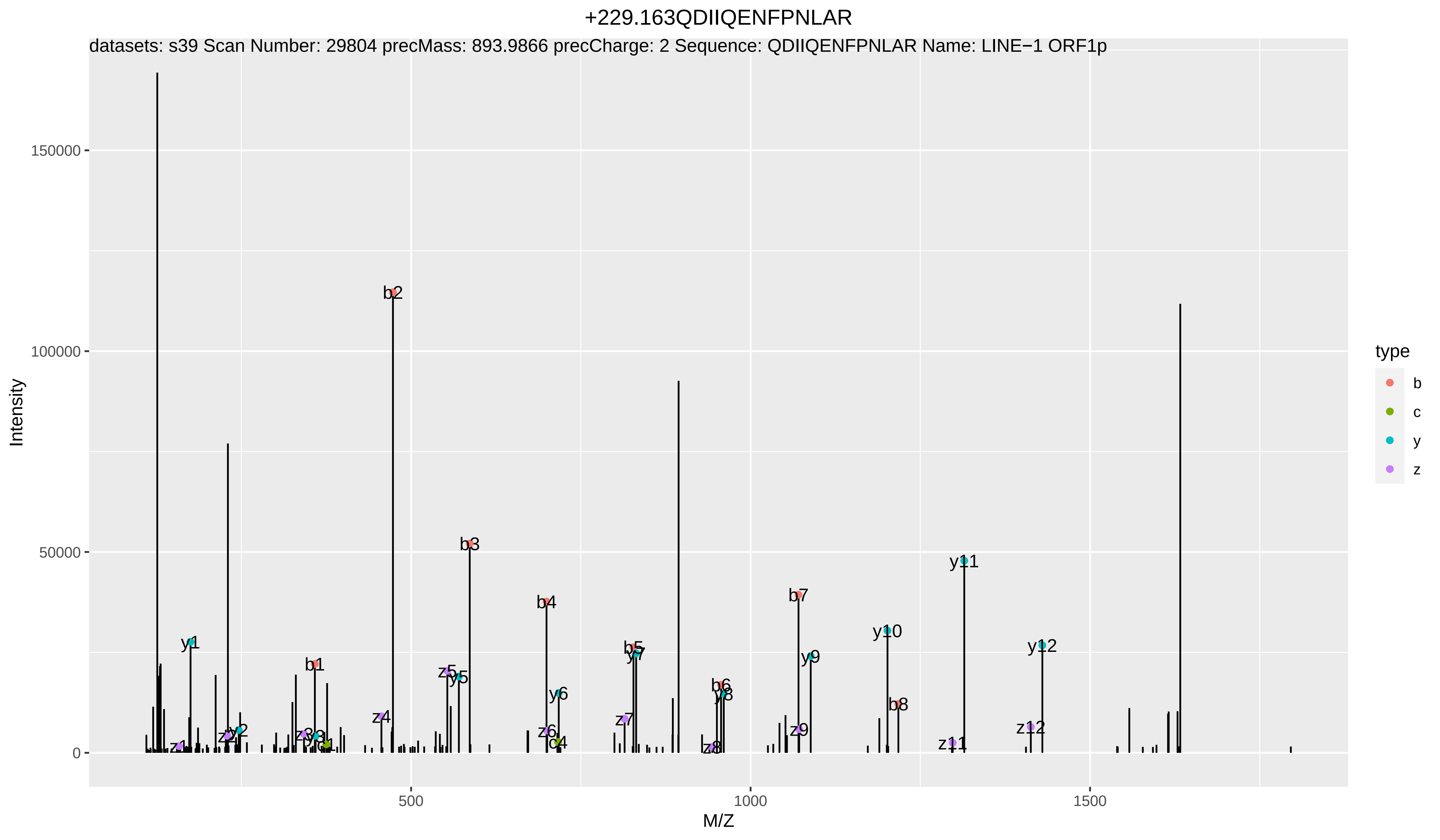
+229.163QDIIQENFPNLAR
datasets: s39 Scan Number: 29804 precMass: 893.9866 precCharge: 2 Sequence: QDIIQENFPNLAR Name: LINE−1 ORF1p
Intensity
M/Z
type
b
c
y
z
b1
b2
b3
b4
b5
b6
b7
b8
y1
y2
y3
y5
y6
y7
y8
y9
y10
y11
y12
z1
z2
z3
z4
z5
z6
z7
z8
z9
z11
z12
c1
c4
0
50000
100000
150000
500
1000
1500

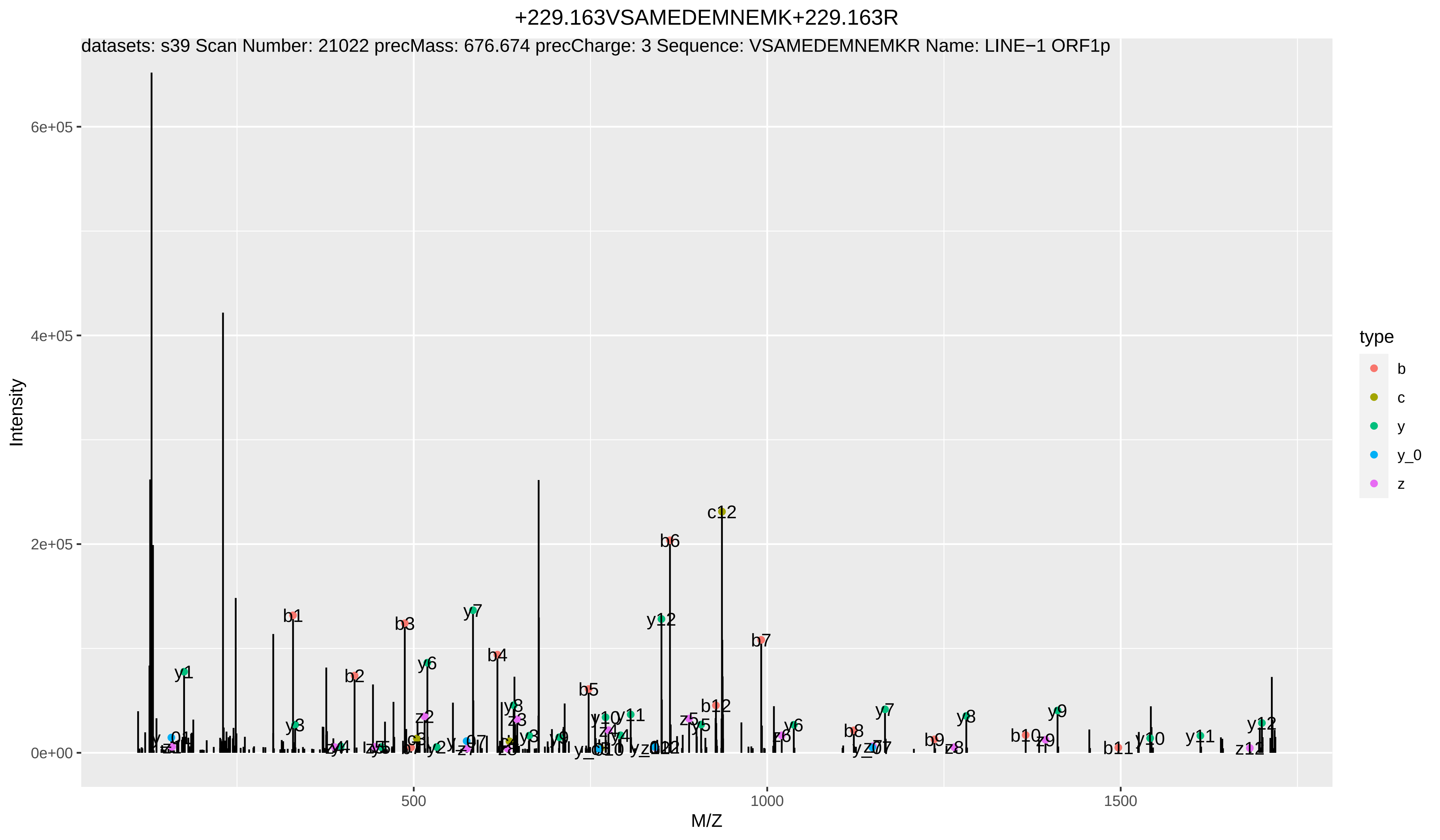

+229.163VSAMEDEMNEMK+229.163R
datasets: s39 Scan Number: 21022 precMass: 676.674 precCharge: 3 Sequence: VSAMEDEMNEMKR Name: LINE−1 ORF1p
Intensity
M/Z
type
b
c
y
y_0
z

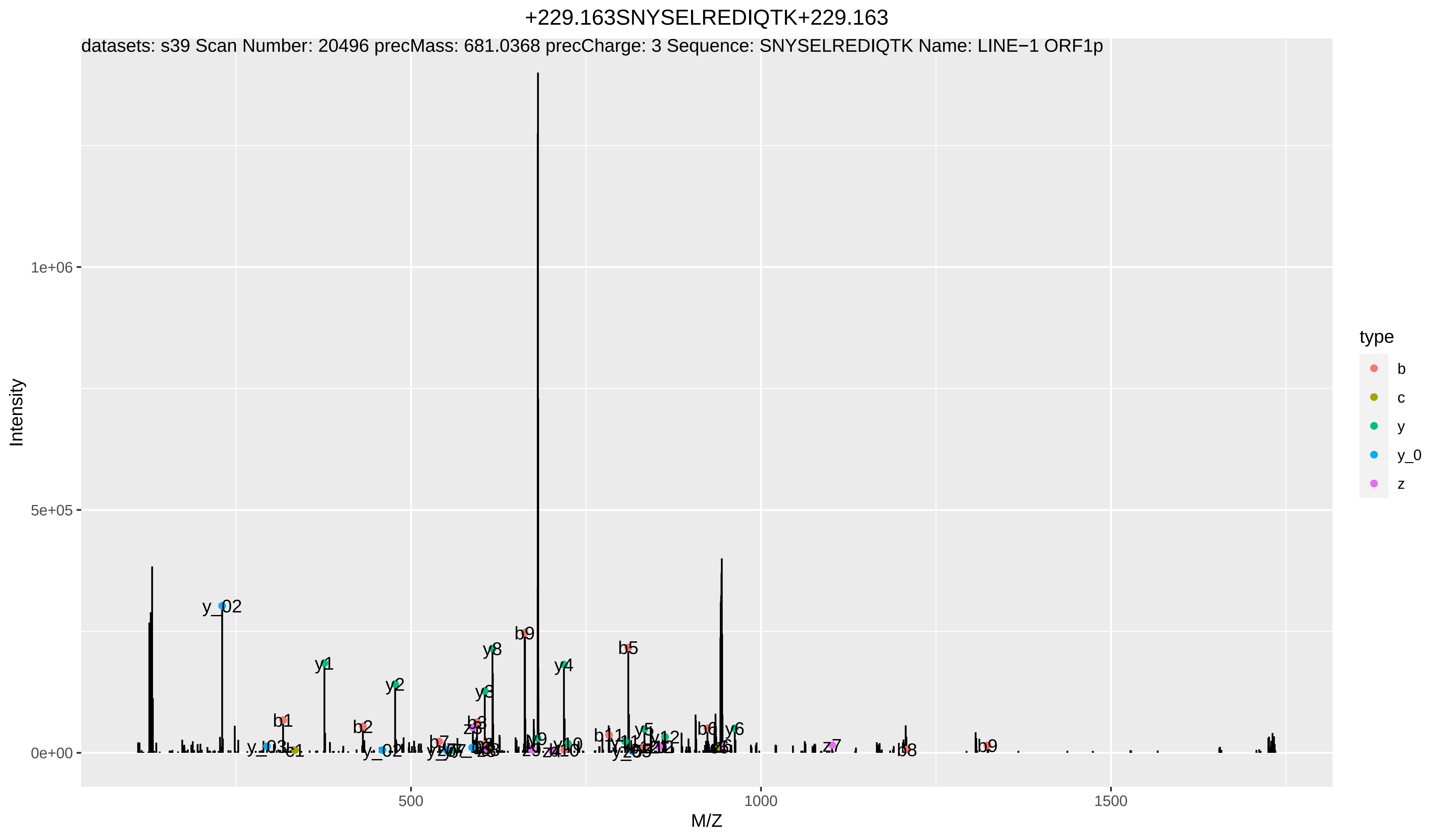
+229.163SNYSELREDIQTK+229.163
datasets: s39 Scan Number: 20496 precMass: 681.0368 precCharge: 3 Sequence: SNYSELREDIQTK Name: LINE−1 ORF1p
Intensity
M/Z
type
b
c
y
y_0
z

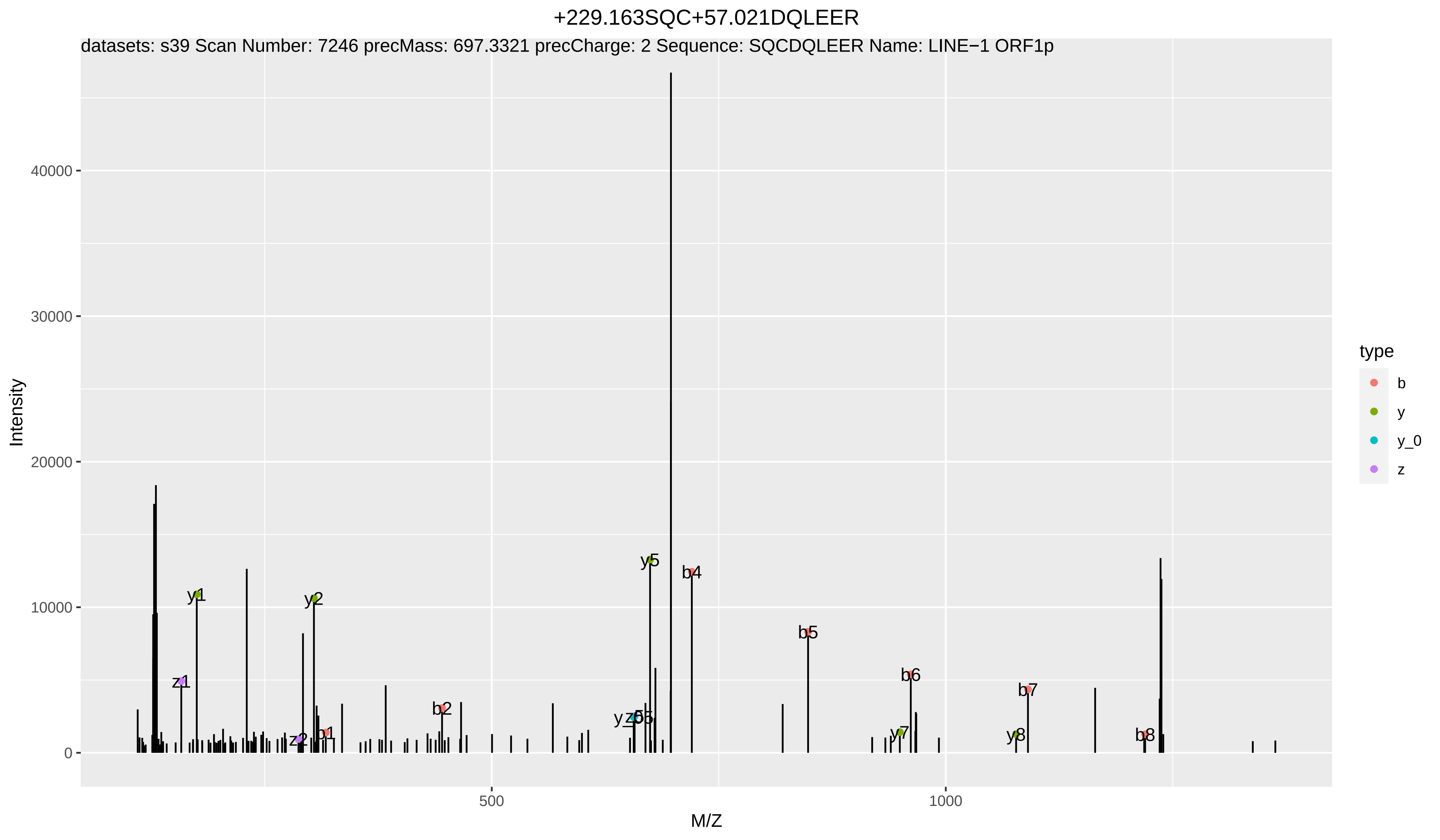

+229.163SQC+57.021DQLEER
datasets: s39 Scan Number: 7246 precMass: 697.3321 precCharge: 2 Sequence: SQCDQLEER Name: LINE−1 ORF1p
Intensity
M/Z
type
b
y
y_0
z

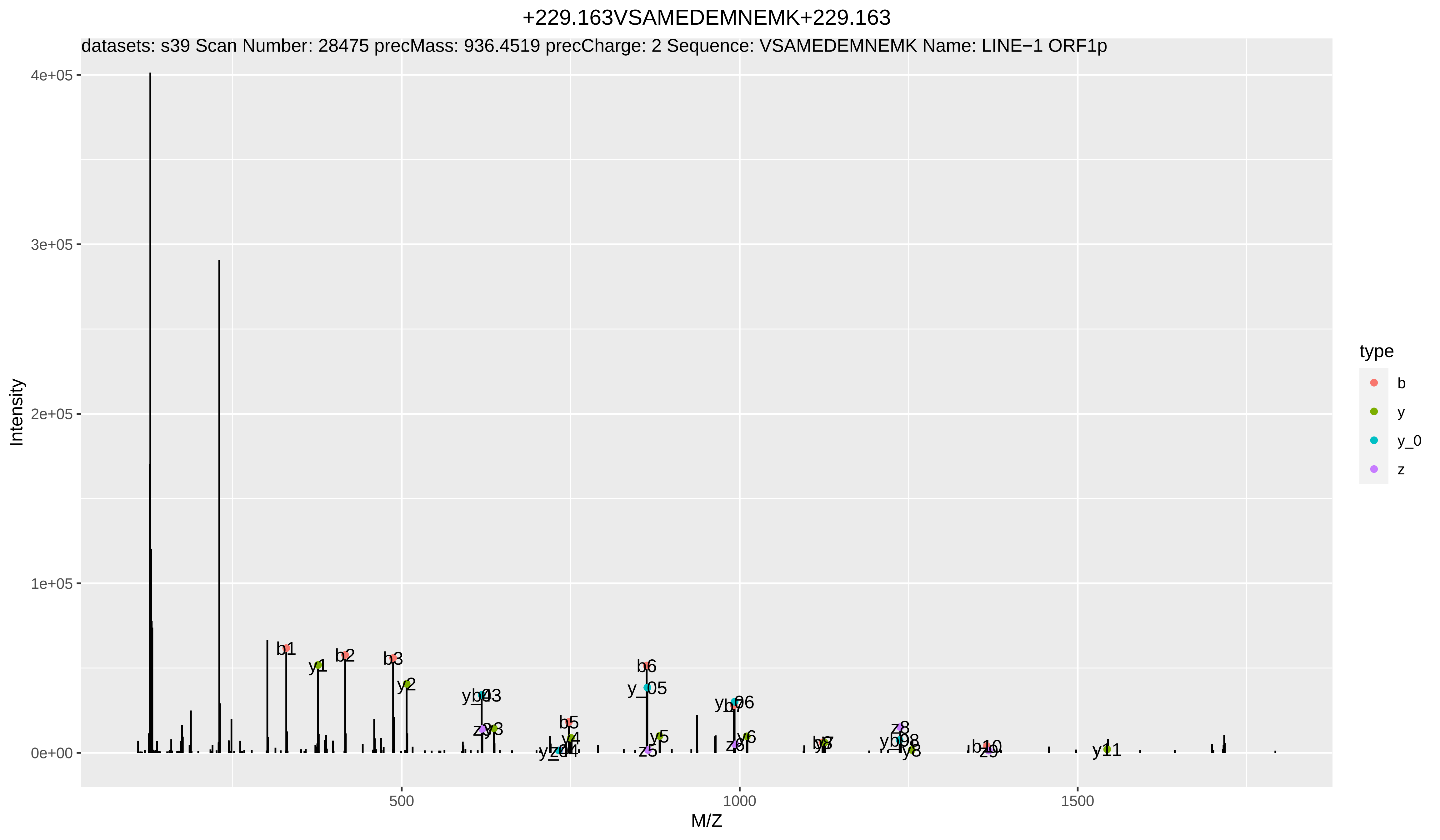
+229.163VSAMEDEMNEMK+229.163
datasets: s39 Scan Number: 28475 precMass: 936.4519 precCharge: 2 Sequence: VSAMEDEMNEMK Name: LINE−1 ORF1p
Intensity
4e+05
3e+05
2e+05
1e+05
0e+00
M/Z
500
1000
1500
type
b
y
y_0
z
b1
y1
b2
b3
y2
b6
y_05
b5
y4
y5
y6
y11
z8
z9
b10

+229.163LENTLQDIIQENFPNLAR

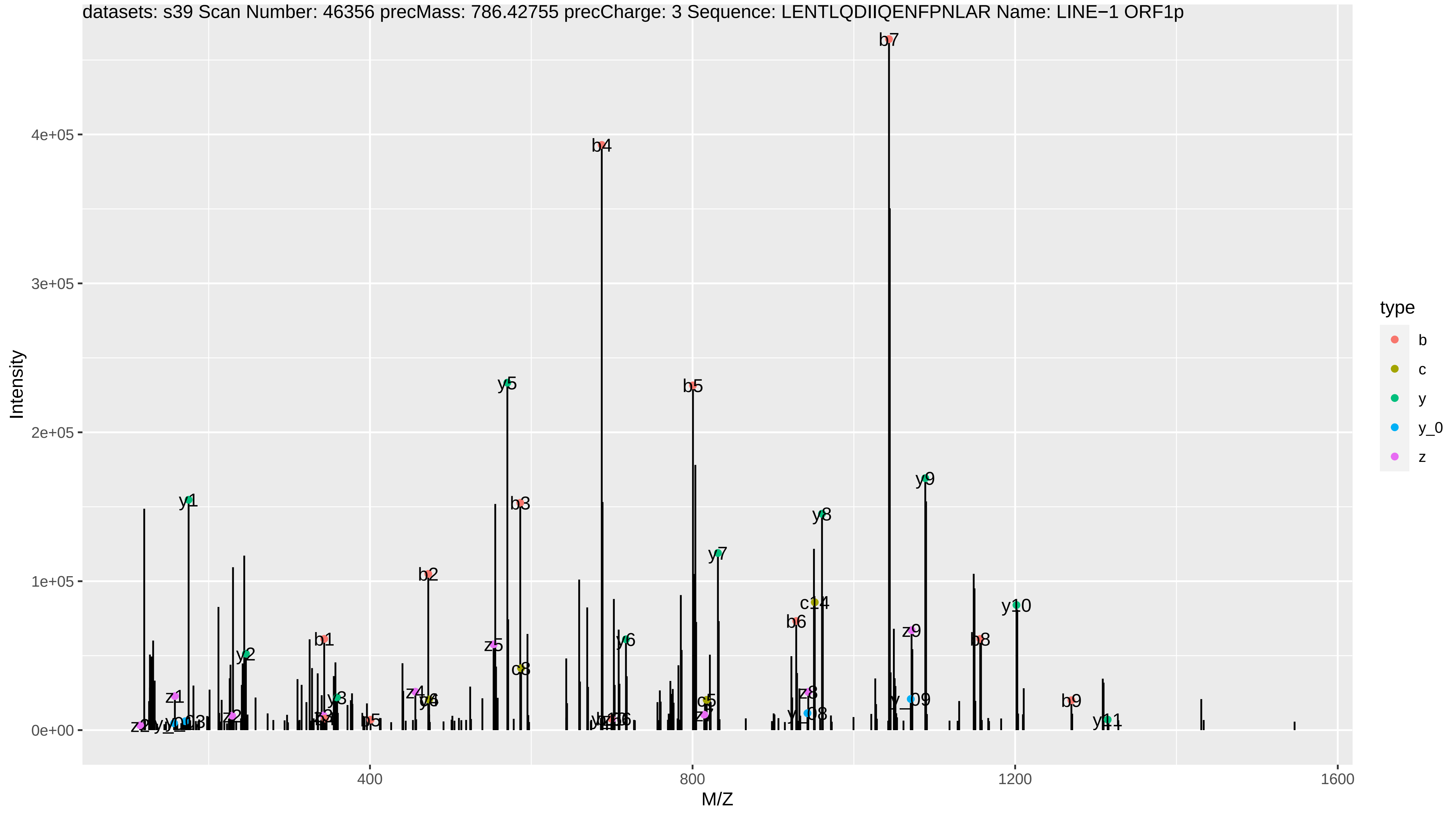

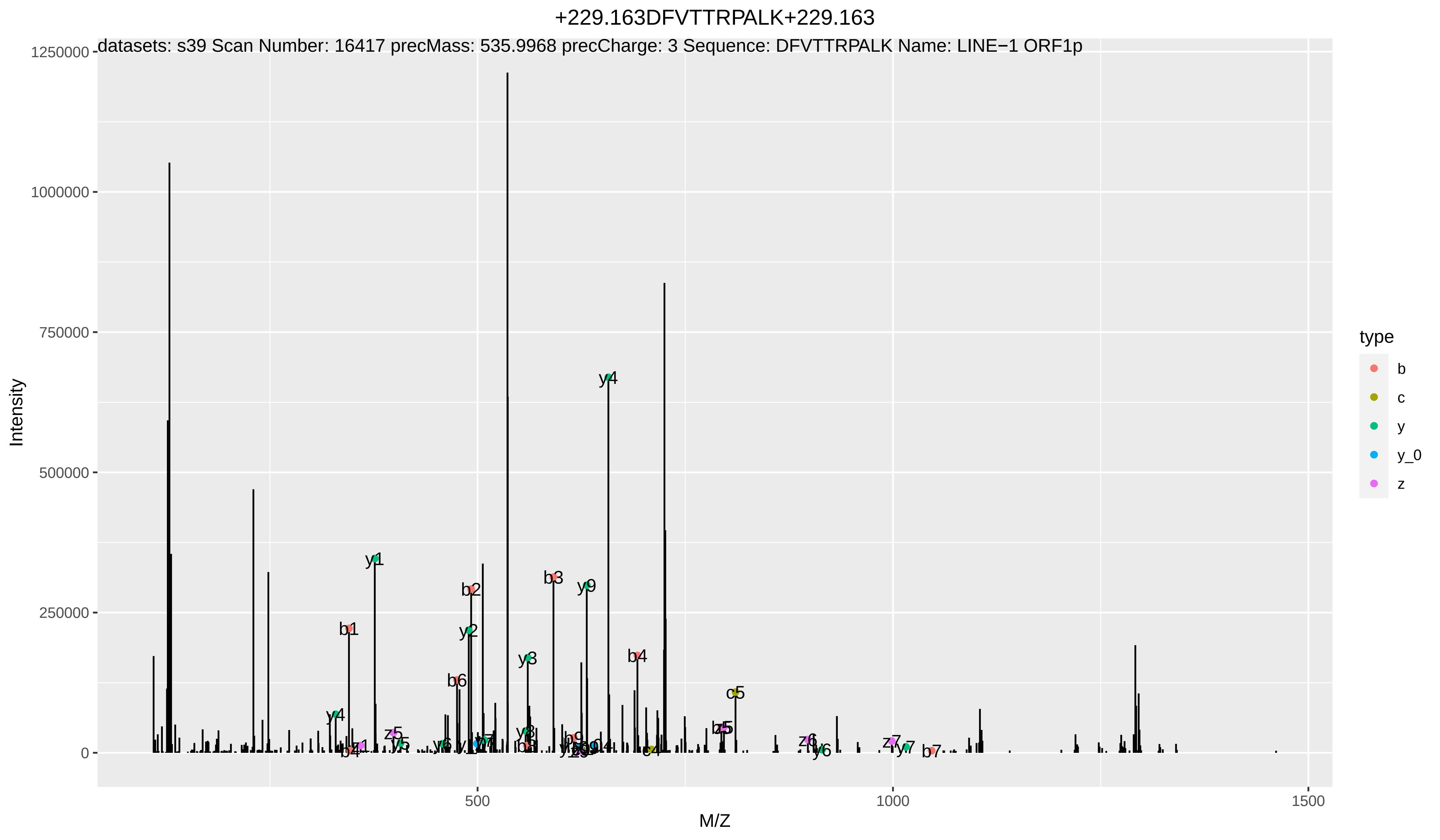
+229.163DFVTTRPALK+229.163
datasets: s39 Scan Number: 16417 precMass: 535.9968 precCharge: 3 Sequence: DFVTTRPALK Name: LINE−1 ORF1p
Intensity
1250000
1000000
750000
500000
250000
0
M/Z
500
1000
1500
type
b
c
y
y_0
z
y1
b1
b2
y2
b3
y9
y3
y4
b4
b6
c5
z6
y6
z7
y7
b7

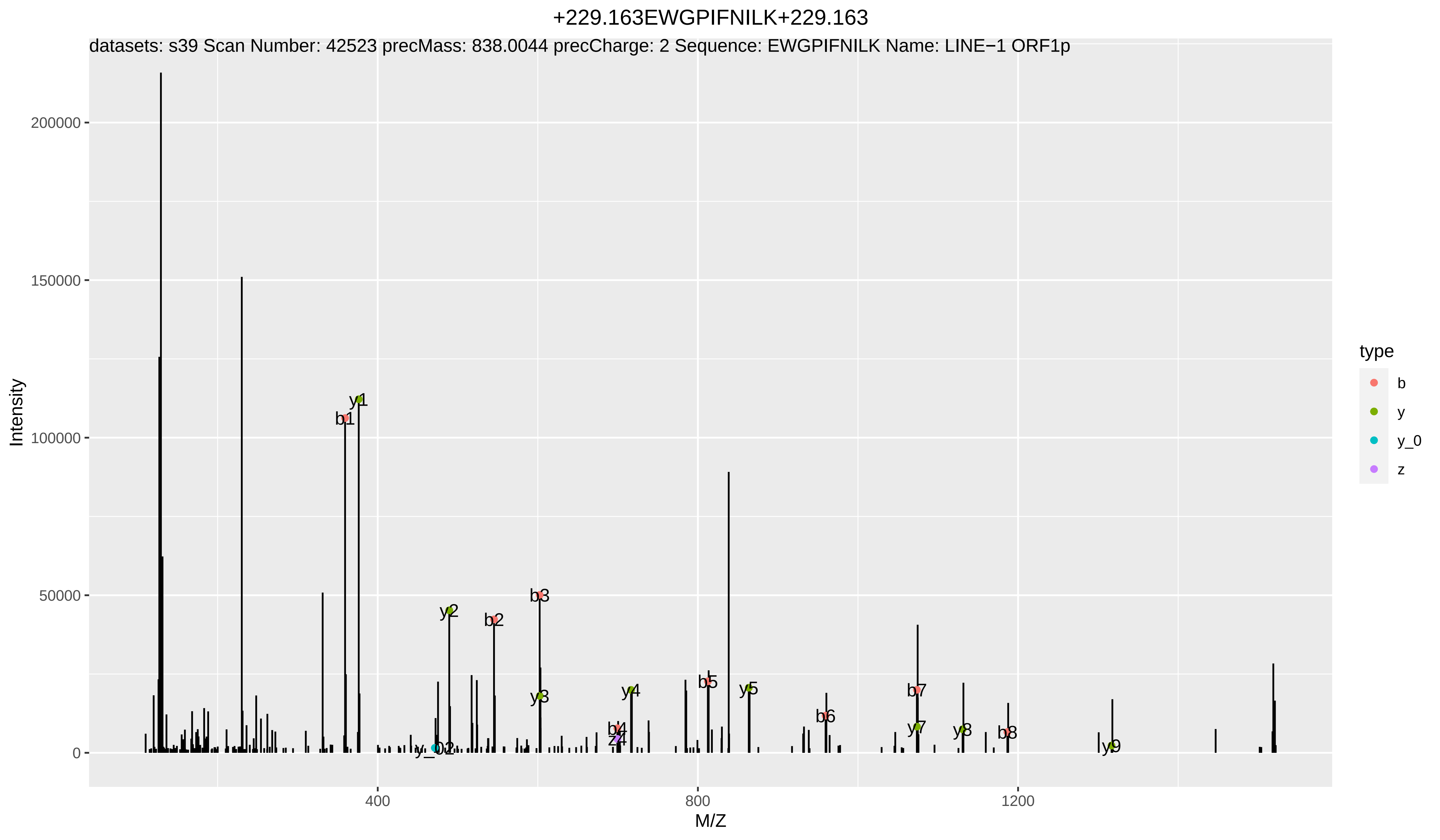
+229.163EWGPIFNILK+229.163
datasets: s39 Scan Number: 42523 precMass: 838.0044 precCharge: 2 Sequence: EWGPIFNILK Name: LINE−1 ORF1p
Intensity
200000
150000
100000
50000
0
M/Z
400
800
1200
y1
b1
y2
b2
b3
y3
y_02
b4
z4
y4
b5
y5
b6
b7
y7
y8
b8
y9
type
b
y
y_0
z

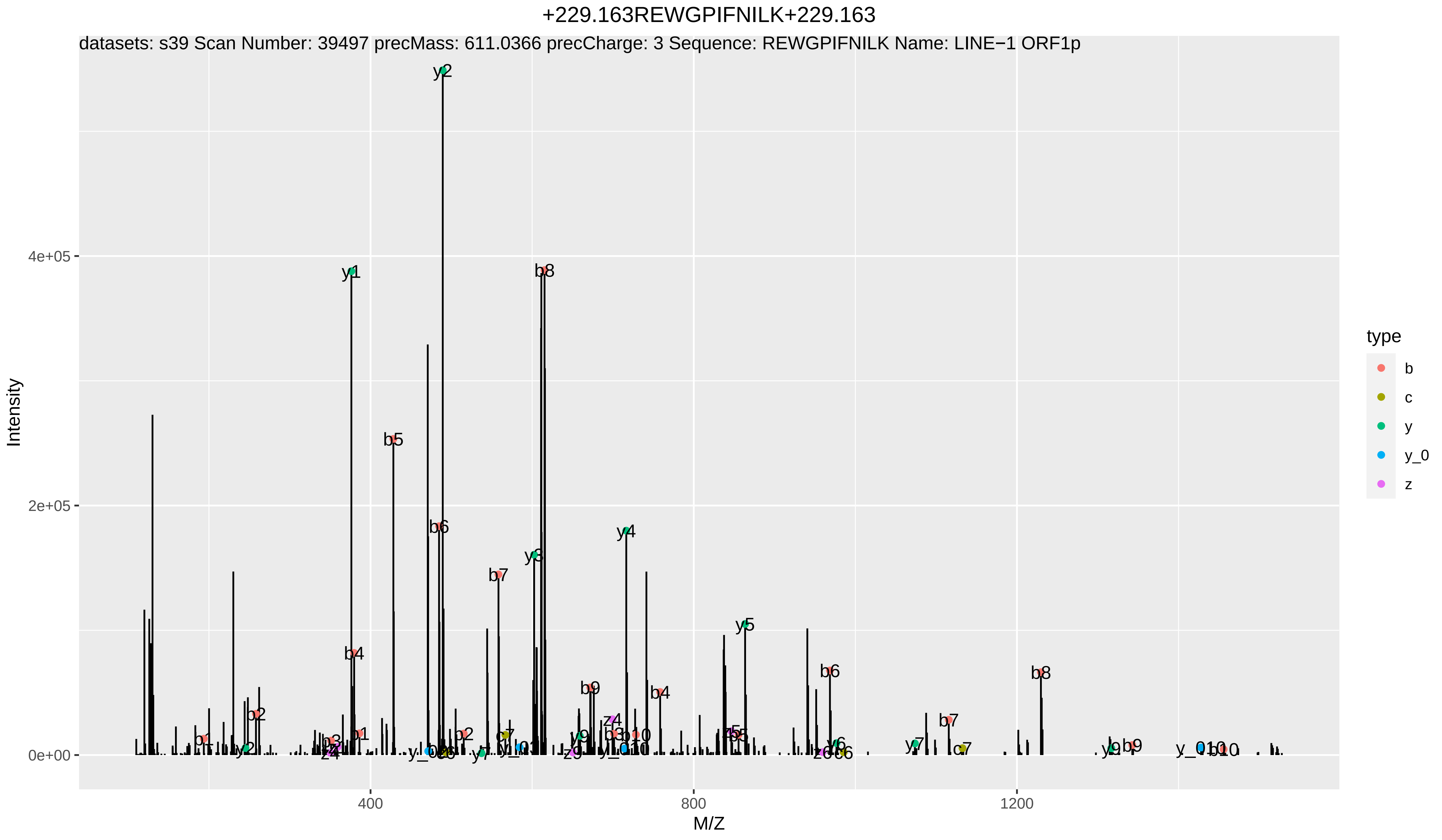

+229.163REWGPIFNILK+229.163
datasets: s39 Scan Number: 39497 precMass: 611.0366 precCharge: 3 Sequence: REWGPIFNILK Name: LINE−1 ORF1p
Intensity
4e+05
2e+05
0e+00
400
800
1200
M/Z
type
b
c
y
y_0
z
y2
y1
b8
b5
b6
y4
y3
b7
y5
b4
b6
b8
b9
b7
b2

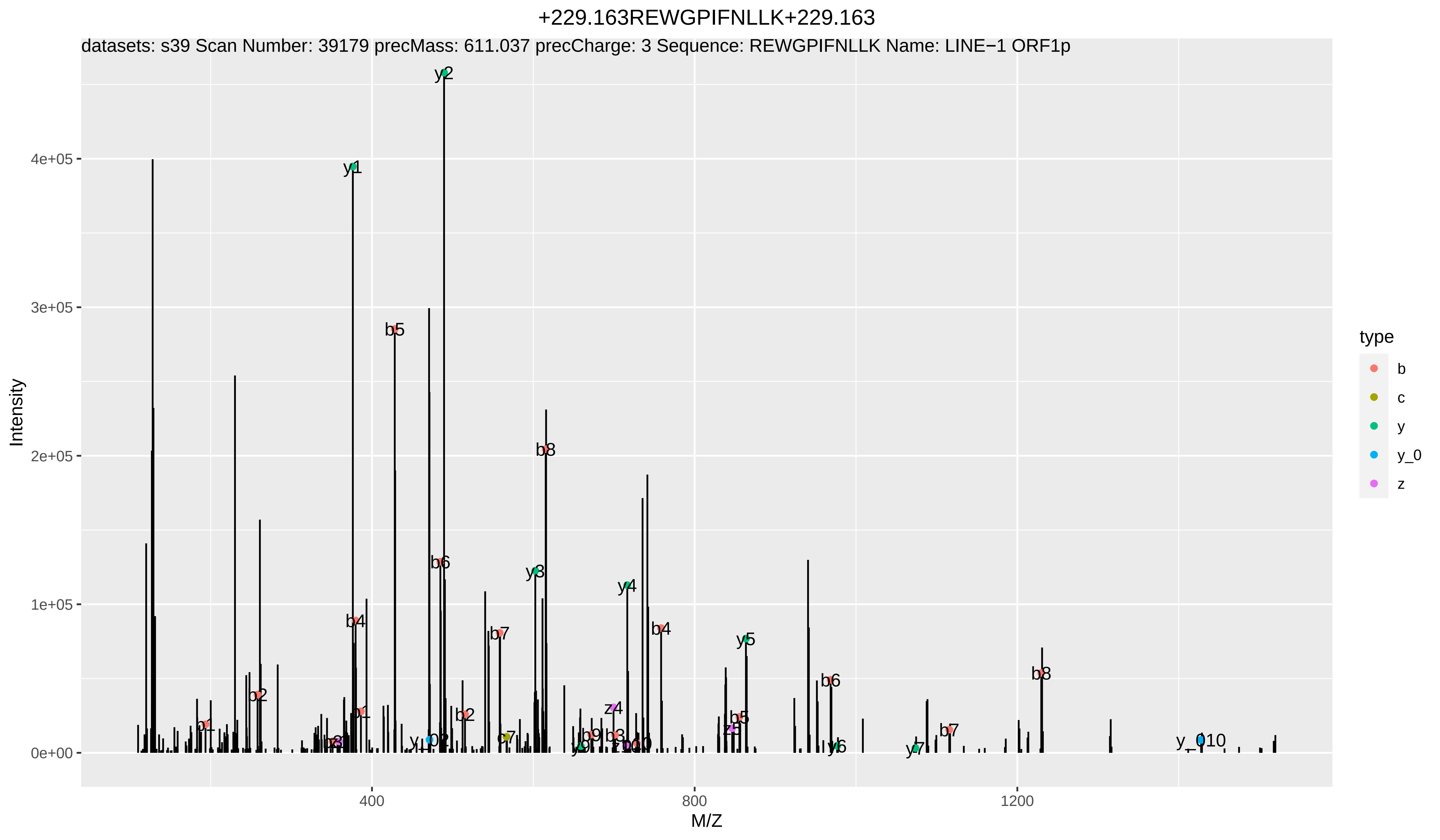
+229.163REWGPIFNLLK+229.163
datasets: s39 Scan Number: 39179 precMass: 611.037 precCharge: 3 Sequence: REWGPIFNLLK Name: LINE−1 ORF1p
Intensity
0e+00
1e+05
2e+05
3e+05
4e+05
M/Z
400
800
1200
type
b
c
y
y_0
z
y2
y1
b5
b8
b6
y3
y4
b4
b7
b4
y5
b6
b8
b2
b1
b2
z4
b5
c7
y7
b7
y6
y_010

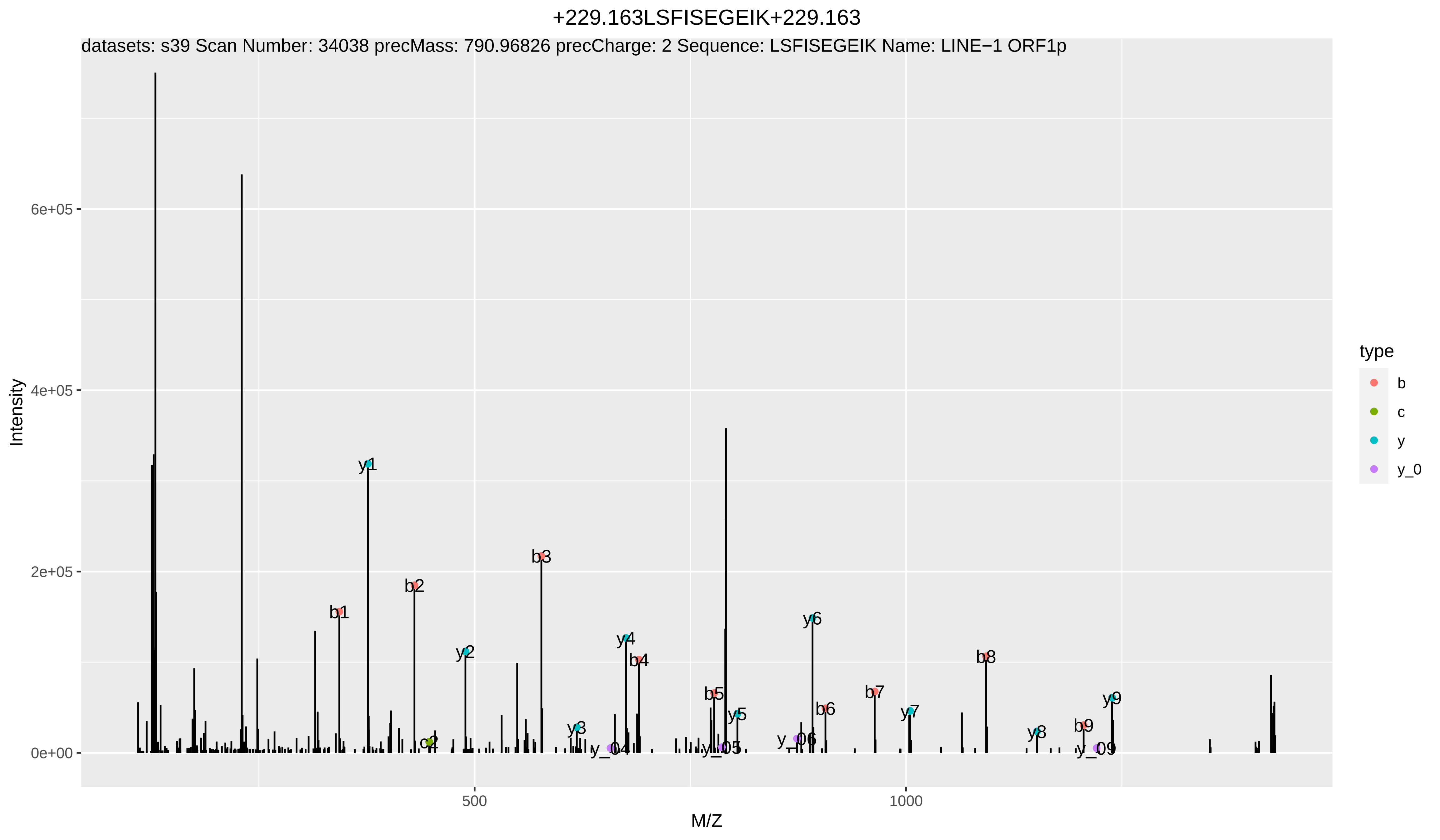
+229.163LSFISEGEIK+229.163
datasets: s39 Scan Number: 34038 precMass: 790.96826 precCharge: 2 Sequence: LSFISEGEIK Name: LINE−1 ORF1p
Intensity
6e+05
4e+05
2e+05
0e+00
M/Z
500
1000
y1
b1
b2
b3
y2
c2
y3
y_04
y4
b4
b5
y5
y_05
y_06
y6
b6
b7
y7
b8
y8
b9
y_09
y9
type
b
c
y
y_0

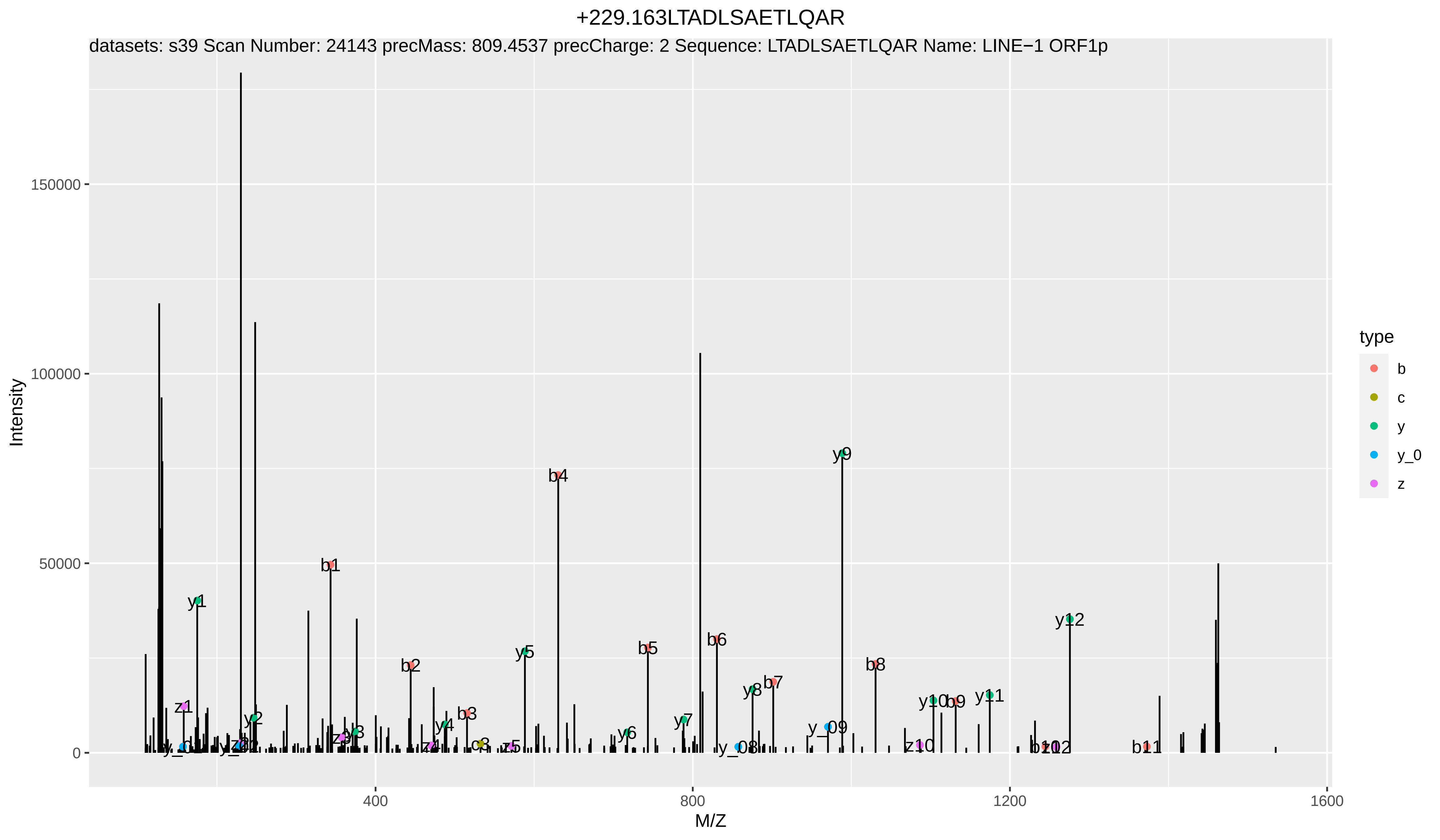
+229.163LTADLSAETLQAR
datasets: s39 Scan Number: 24143 precMass: 809.4537 precCharge: 2 Sequence: LTADLSAETLQAR Name: LINE−1 ORF1p
Intensity
M/Z
0
50000
100000
150000
400
800
1200
1600
type
b
c
y
y_0
z
y1
z1
b1
y2
y3
b2
y4
b3
y5
b4
y6
b5
y7
b6
y8
b7
y_08
y_09
y9
b8
z10
y10
b9
y11
y12
b11

+229.163LTVDLSAETLQAR

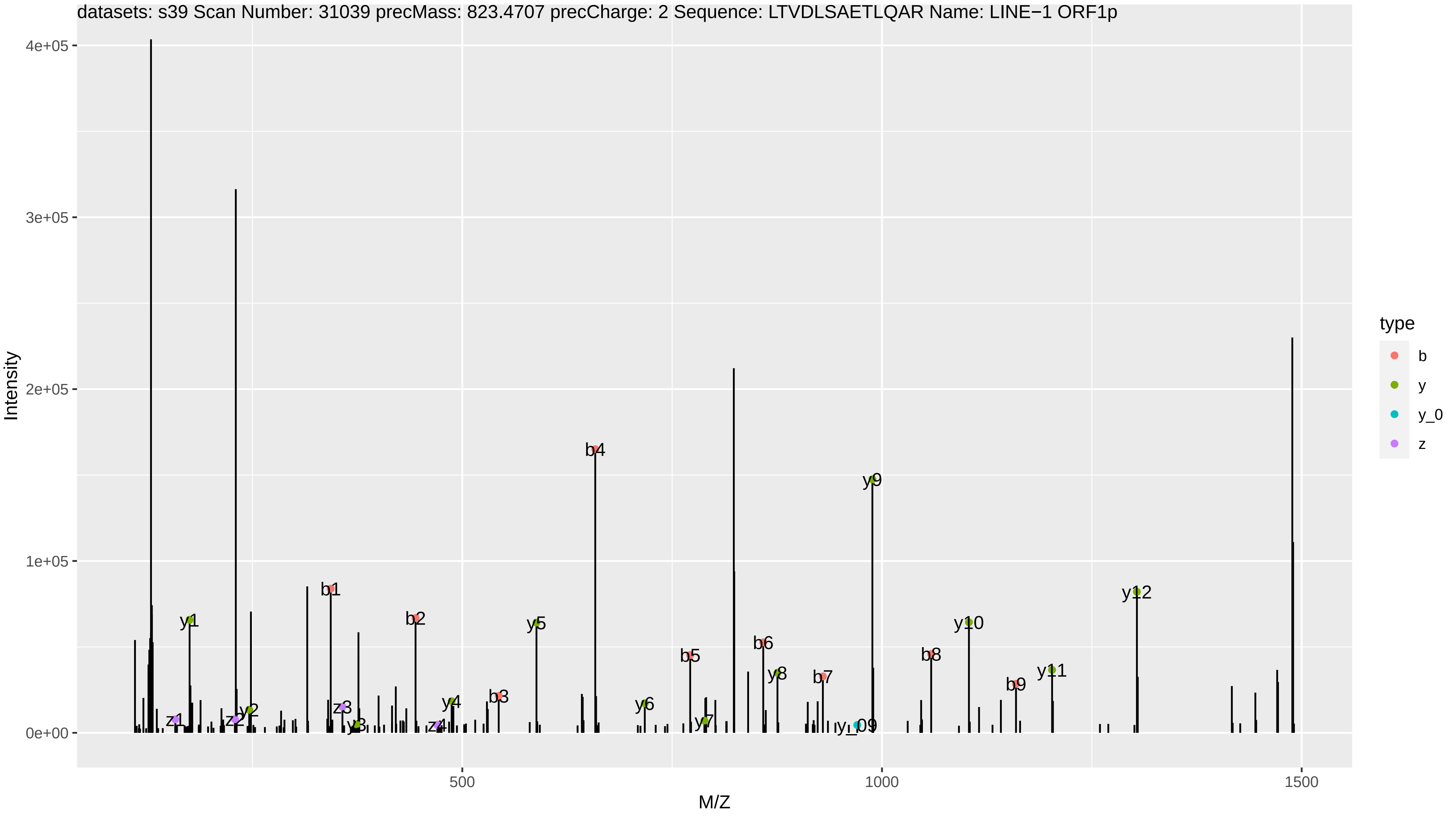

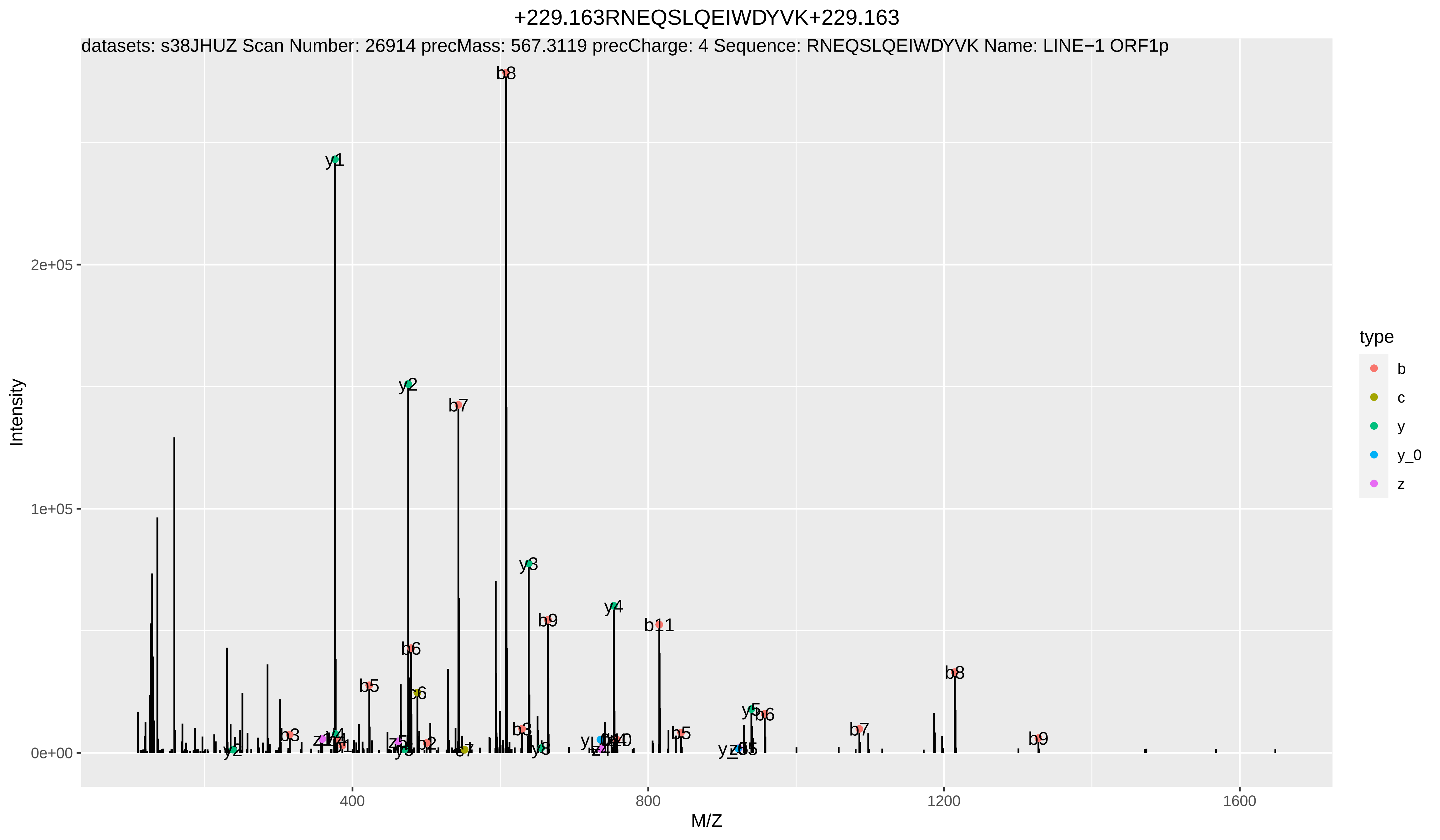
+229.163RNEQSLQEIWDYVK+229.163
datasets: s38JHUZ Scan Number: 26914 precMass: 567.3119 precCharge: 4 Sequence: RNEQSLQEIWDYVK Name: LINE−1 ORF1p
Intensity
0e+00
1e+05
2e+05
M/Z
400
800
1200
1600
b8
y1
y2
b7
y3
y4
b9
b11
b6
b5
c6
b8
y5
b6
b7
b3
b5
b9
b3
b2
y2
y3
c7
y8
z4
type
b
c
y
y_0
z

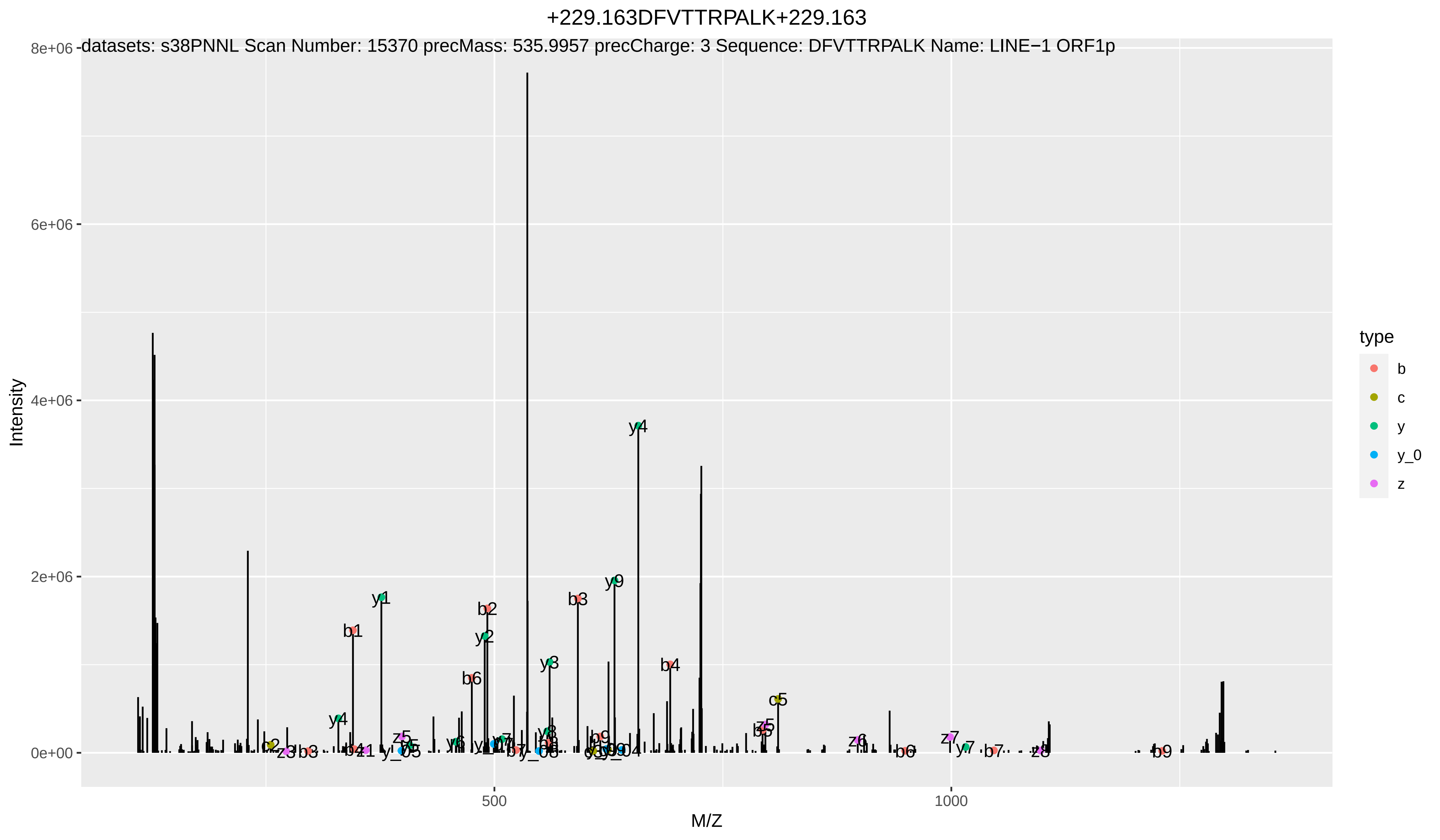
+229.163DFVTTRPALK+229.163
datasets: s38PNNL Scan Number: 15370 precMass: 535.9957 precCharge: 3 Sequence: DFVTTRPALK Name: LINE−1 ORF1p
Intensity
8e+06
6e+06
4e+06
2e+06
0e+00
M/Z
500
1000
type
b
c
y
y_0
z

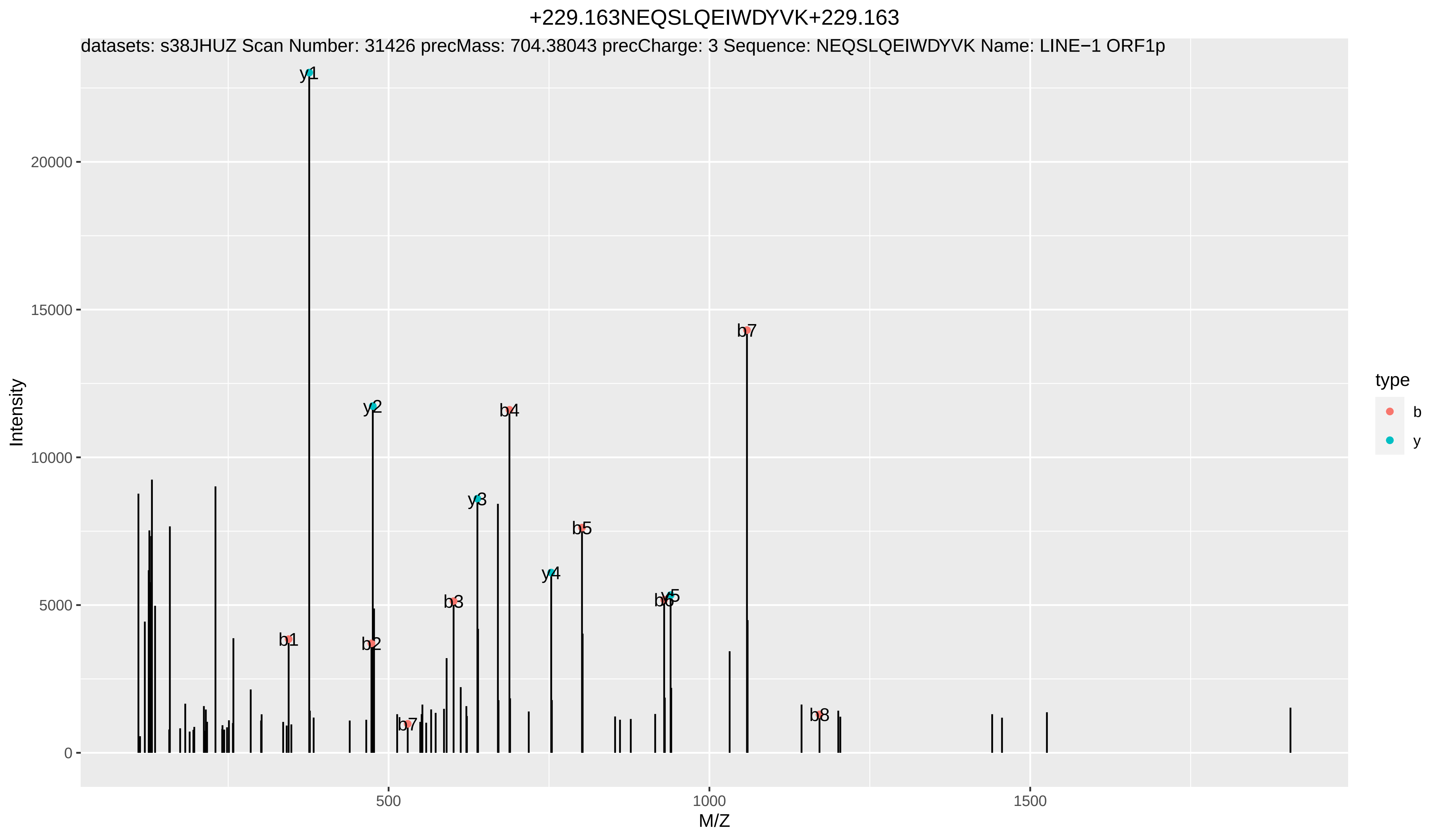

+229.163NEQSLQEIWDYVK+229.163
datasets: s38JHUZ Scan Number: 31426 precMass: 704.38043 precCharge: 3 Sequence: NEQSLQEIWDYVK Name: LINE−1 ORF1p
Intensity
M/Z
0
5000
10000
15000
20000
500
1000
1500
y1
y2
y3
y4
y5
b1
b2
b3
b4
b5
b6
b7
b7
b8
type
b
y

+229.163RNEQSLQEIWDYVK+229.163

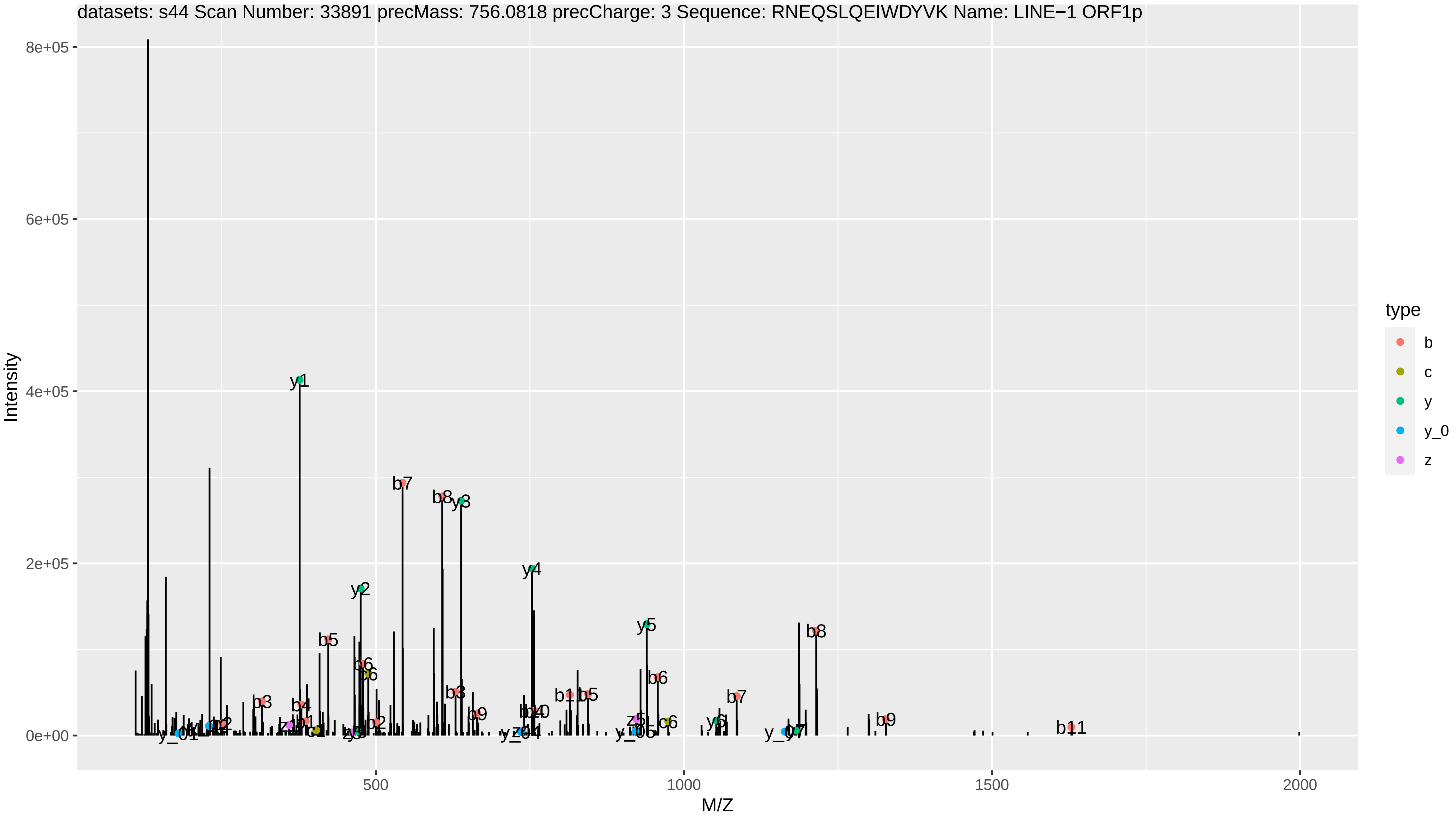

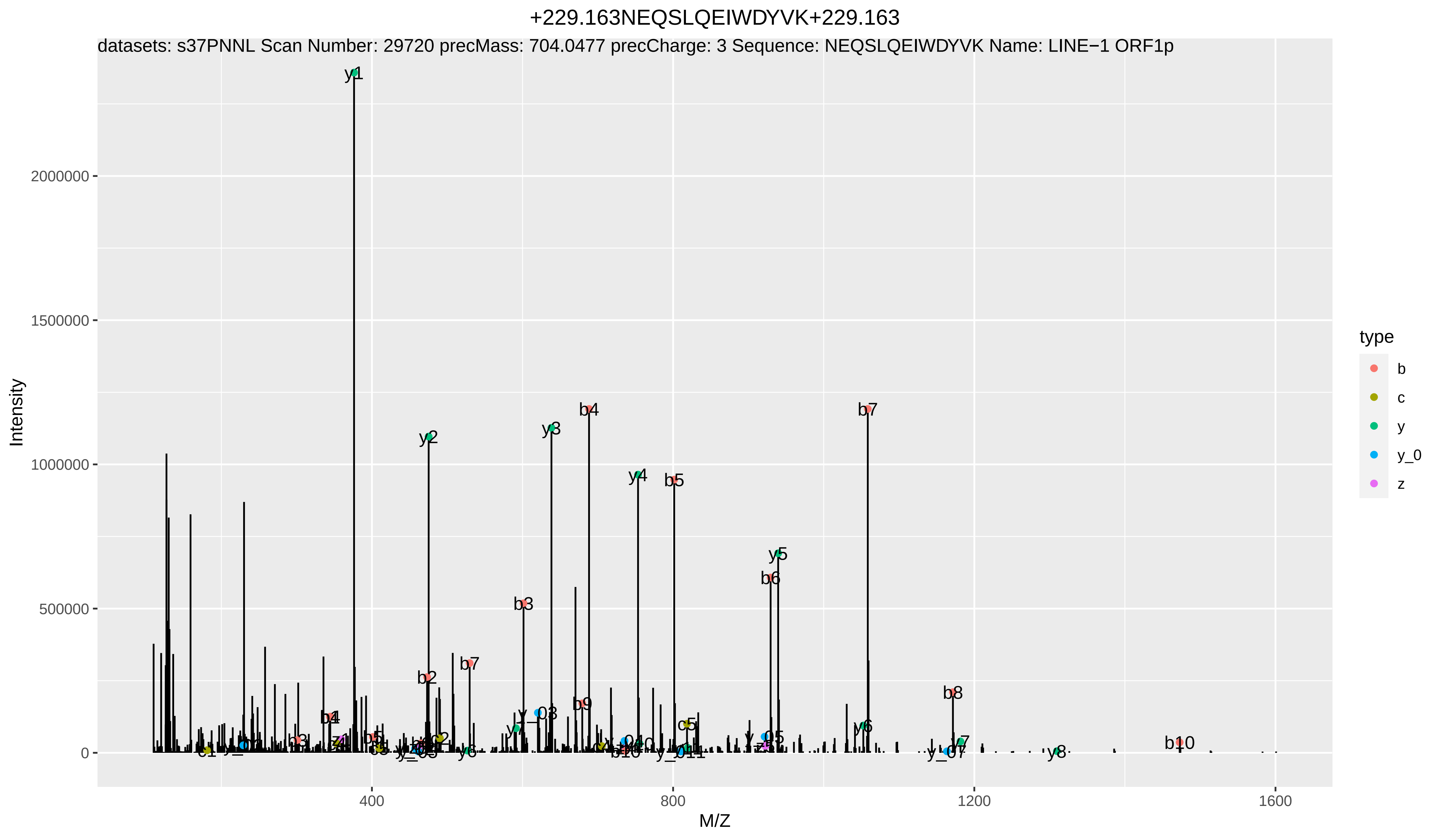

+229.163NEQSLQEIWDYVK+229.163
datasets: s37PNNL Scan Number: 29720 precMass: 704.0477 precCharge: 3 Sequence: NEQSLQEIWDYVK Name: LINE−1 ORF1p
Intensity
M/Z
type
b
c
y
y_0
z
y1
y2
y3
b4
y4
b5
y5
b6
b7
b3
b2
b7
b8
b9
b10
y6
y7
y8

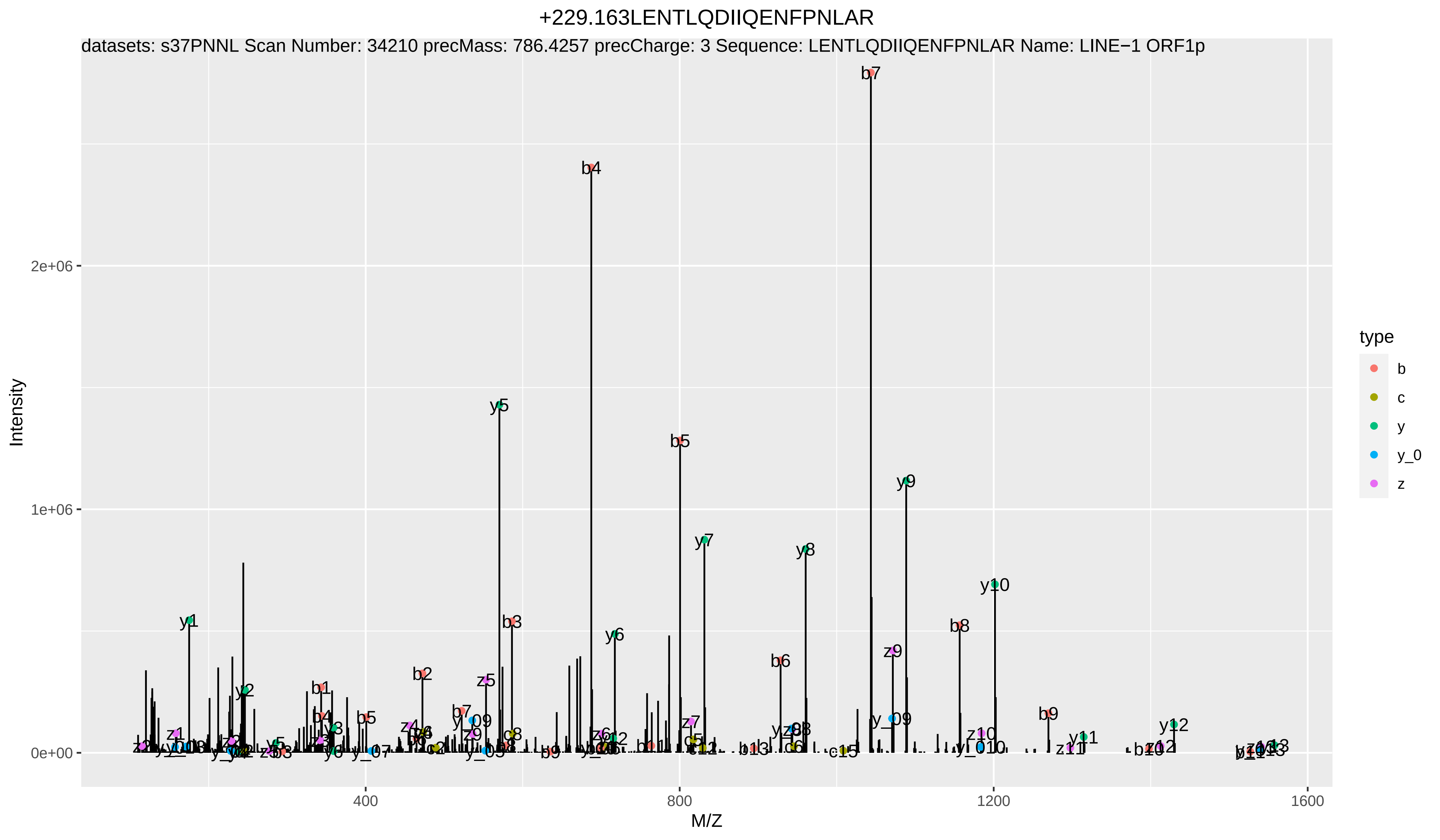
+229.163LENTLQDIIQENFPNLAR
datasets: s37PNNL Scan Number: 34210 precMass: 786.4257 precCharge: 3 Sequence: LENTLQDIIQENFPNLAR Name: LINE−1 ORF1p
Intensity
M/Z
0e+00
1e+06
2e+06
400
800
1200
1600
type
b
c
y
y_0
z
b7
b4
y5
b5
y9
y7
y8
y10
b8
b3
y1
y6
z9
b6
b2
z5
y2
b1
b9
y12
y11
z10
z7
y_09
z11
b10
c15
b13

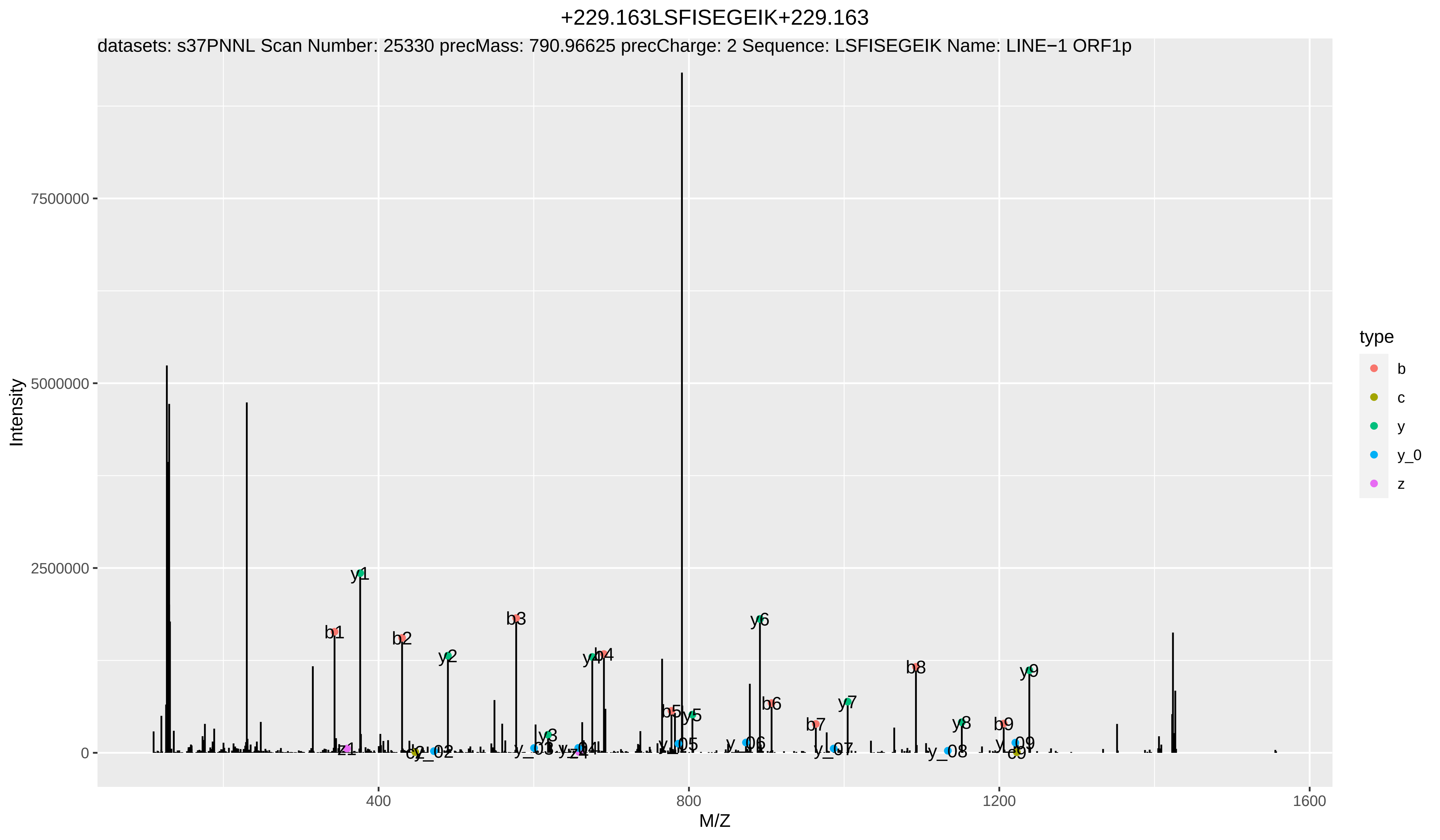
+229.163LSFISEGEIK+229.163
datasets: s37PNNL Scan Number: 25330 precMass: 790.96625 precCharge: 2 Sequence: LSFISEGEIK Name: LINE−1 ORF1p
Intensity
7500000
5000000
2500000
0
M/Z
400
800
1200
1600
type
b
c
y
y_0
z
y1
b1
b2
y2
b3
y3
b4
b5
y5
y6
b6
b7
y7
b8
y8
b9
y9
z1
y_02
y_03
y_05
y_06
y_07
y_08
c9

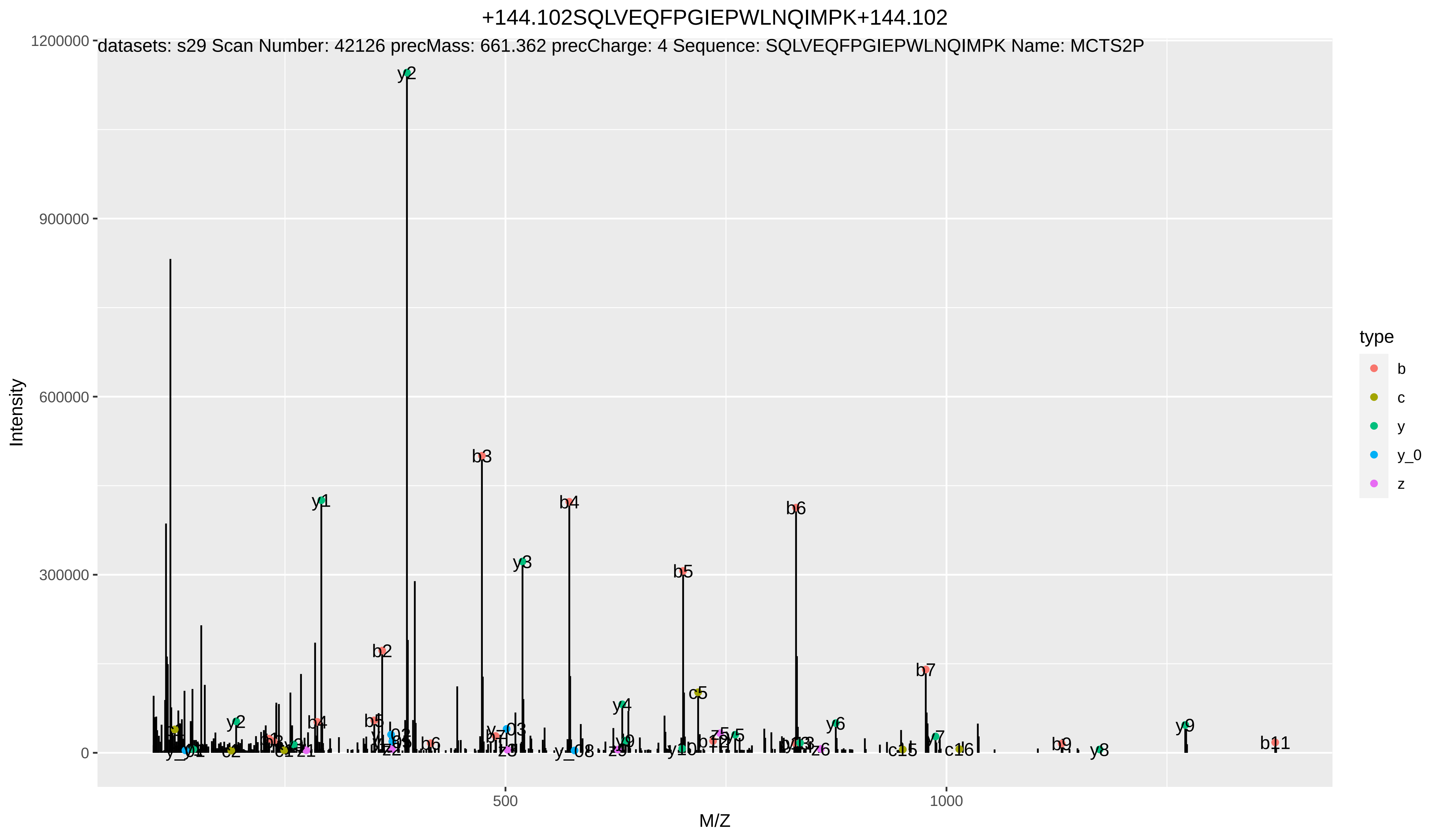
+144.102SQLVEQFPGIEPWLNQIMPK+144.102
datasets: s29 Scan Number: 42126 precMass: 661.362 precCharge: 4 Sequence: SQLVEQFPGIEPWLNQIMPK Name: MCTS2P
Intensity
1200000
900000
600000
300000
0
M/Z
500
1000
type
b
c
y
y_0
z
y2
b3
y1
b4
y3
b6
b5
b2
b7
c5
y4
y6
y9
b11
b9
y8
c15
c16
b6

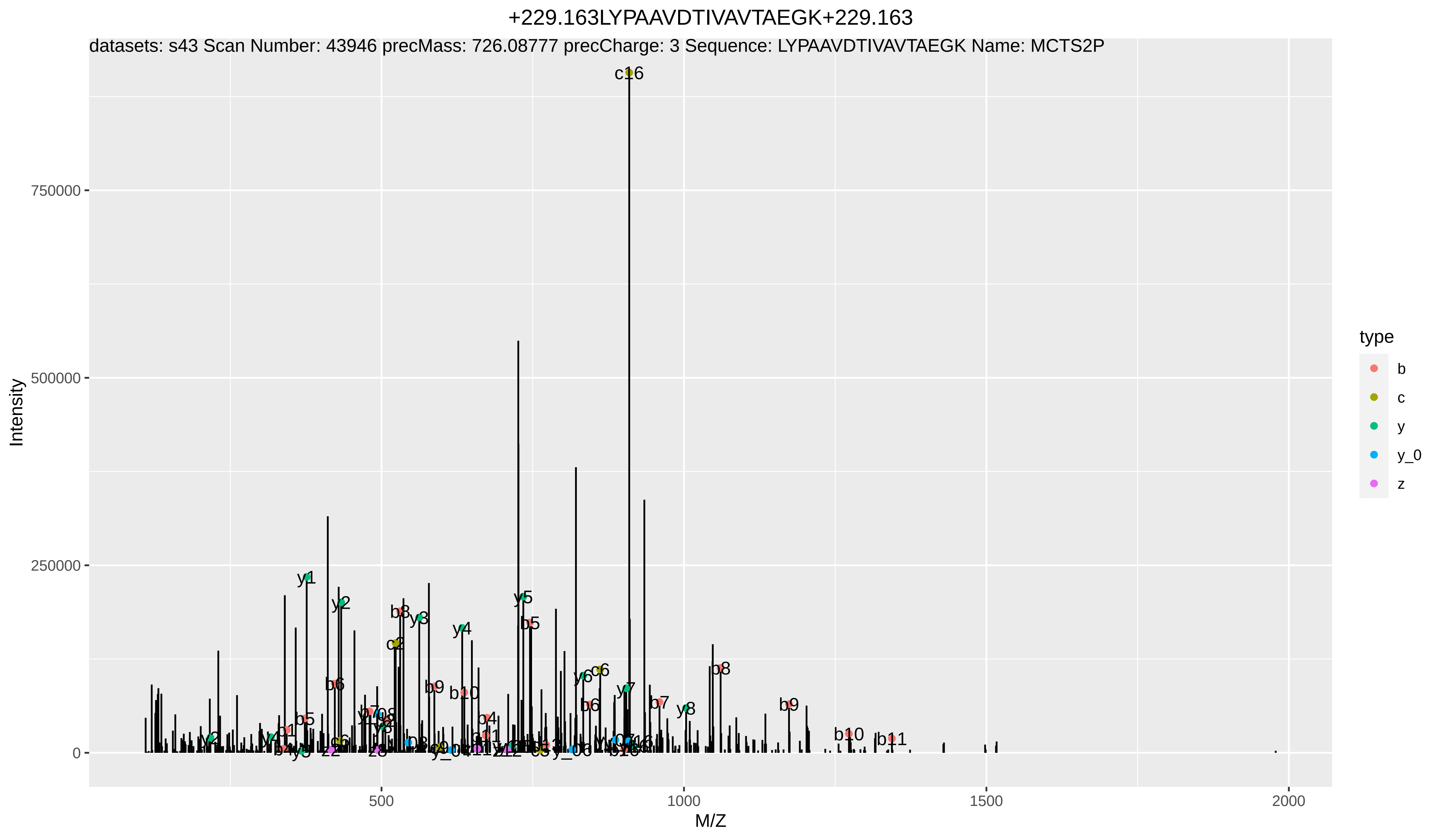

+229.163LYPAAVDTIVAVTAEGK+229.163
datasets: s43 Scan Number: 43946 precMass: 726.08777 precCharge: 3 Sequence: LYPAAVDTIVAVTAEGK Name: MCTS2P
Intensity
M/Z
type
b
c
y
y_0
z

+229.163ESVSNC+57.021IQLK+229.163

datasets: s43 Scan Number: 24395 precMass: 818.4618 precCharge: 2 Sequence: ESVSNCIQLK Name: MCTS2P

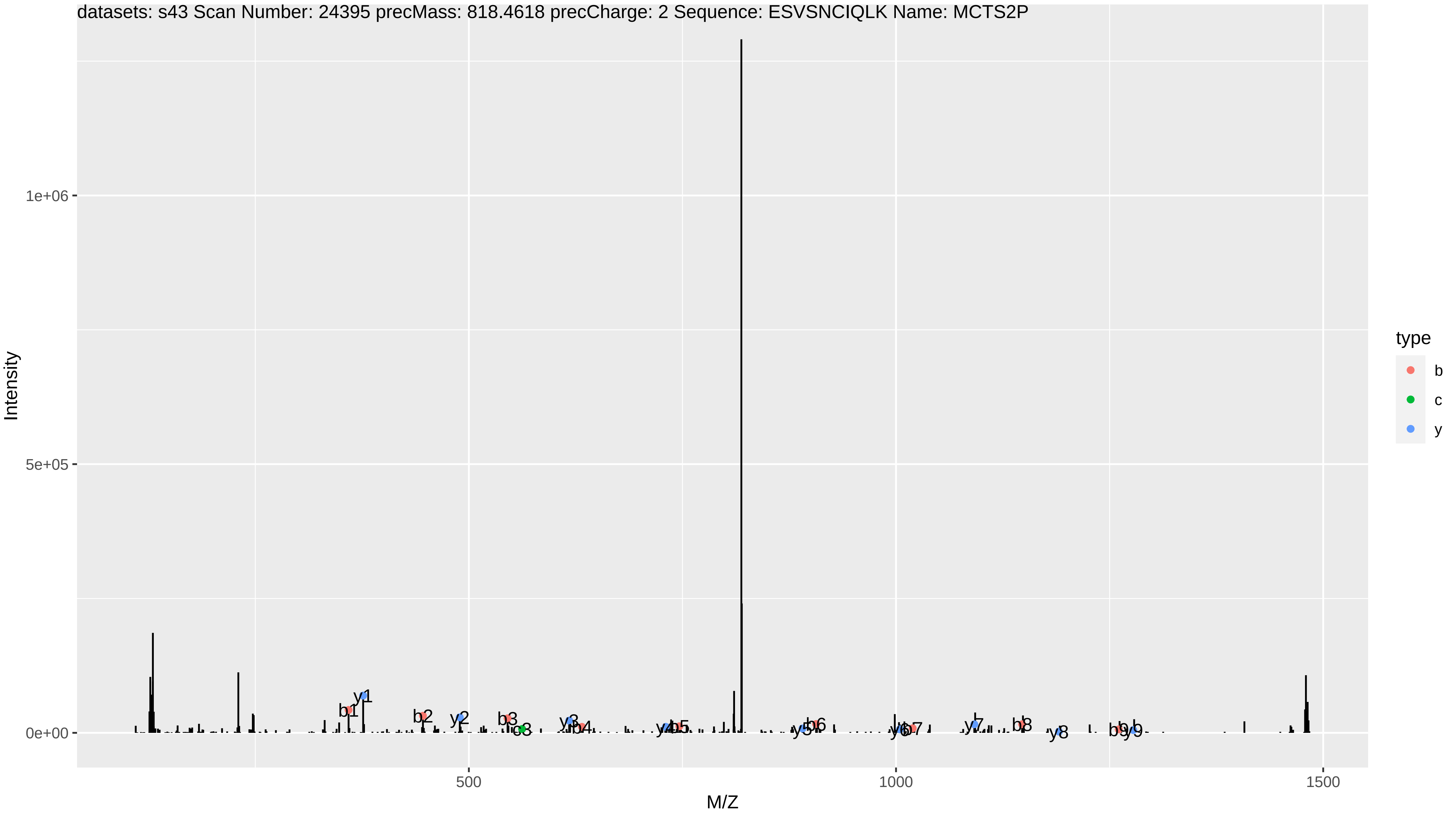

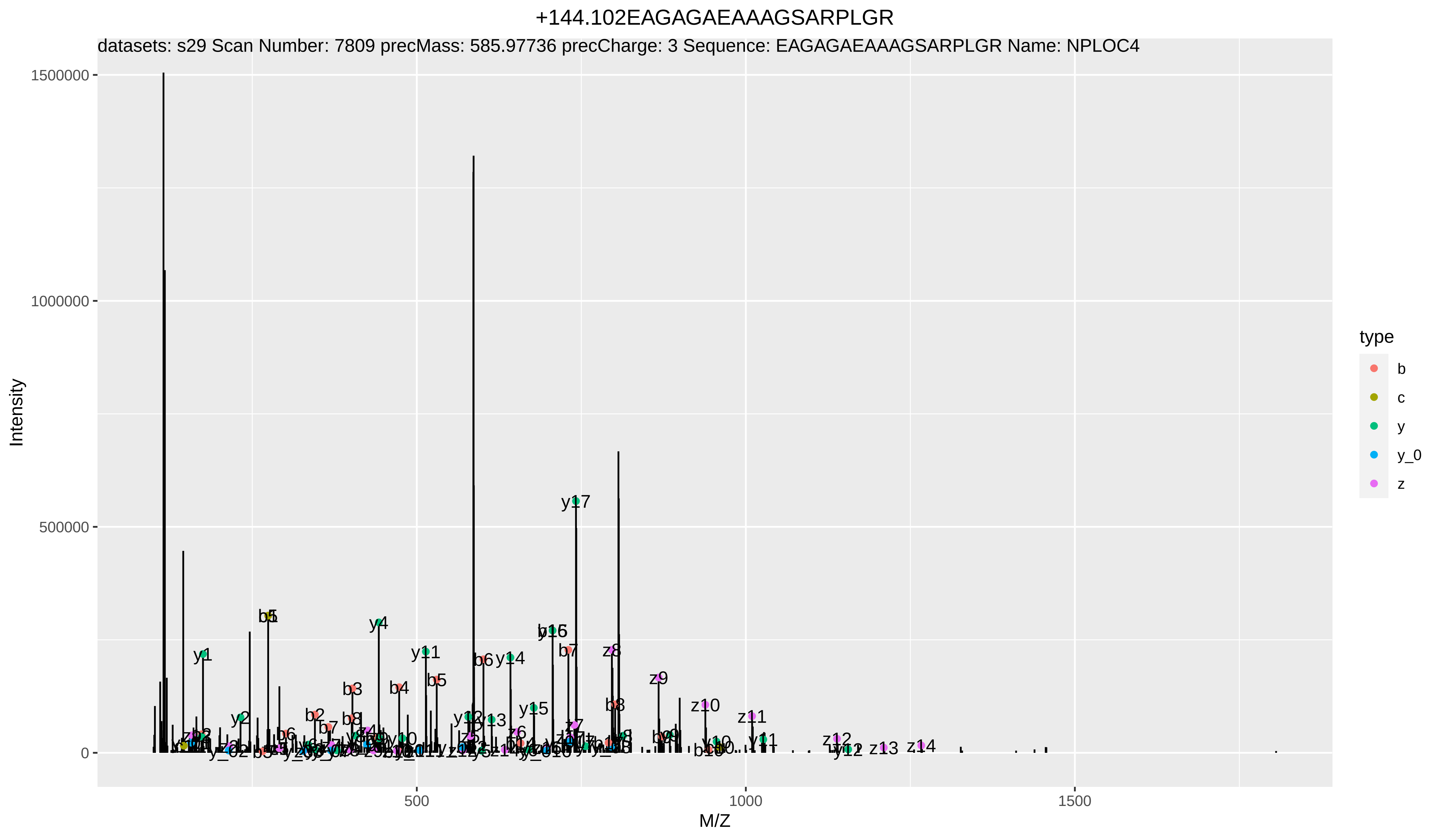

+144.102EAGAGAEAAAGSARPLGR
datasets: s29 Scan Number: 7809 precMass: 585.97736 precCharge: 3 Sequence: EAGAGAEAAAGSARPLGR Name: NPLOC4
Intensity
1500000
1000000
500000
0
500
1000
1500
M/Z
type
b
c
y
y_0
z

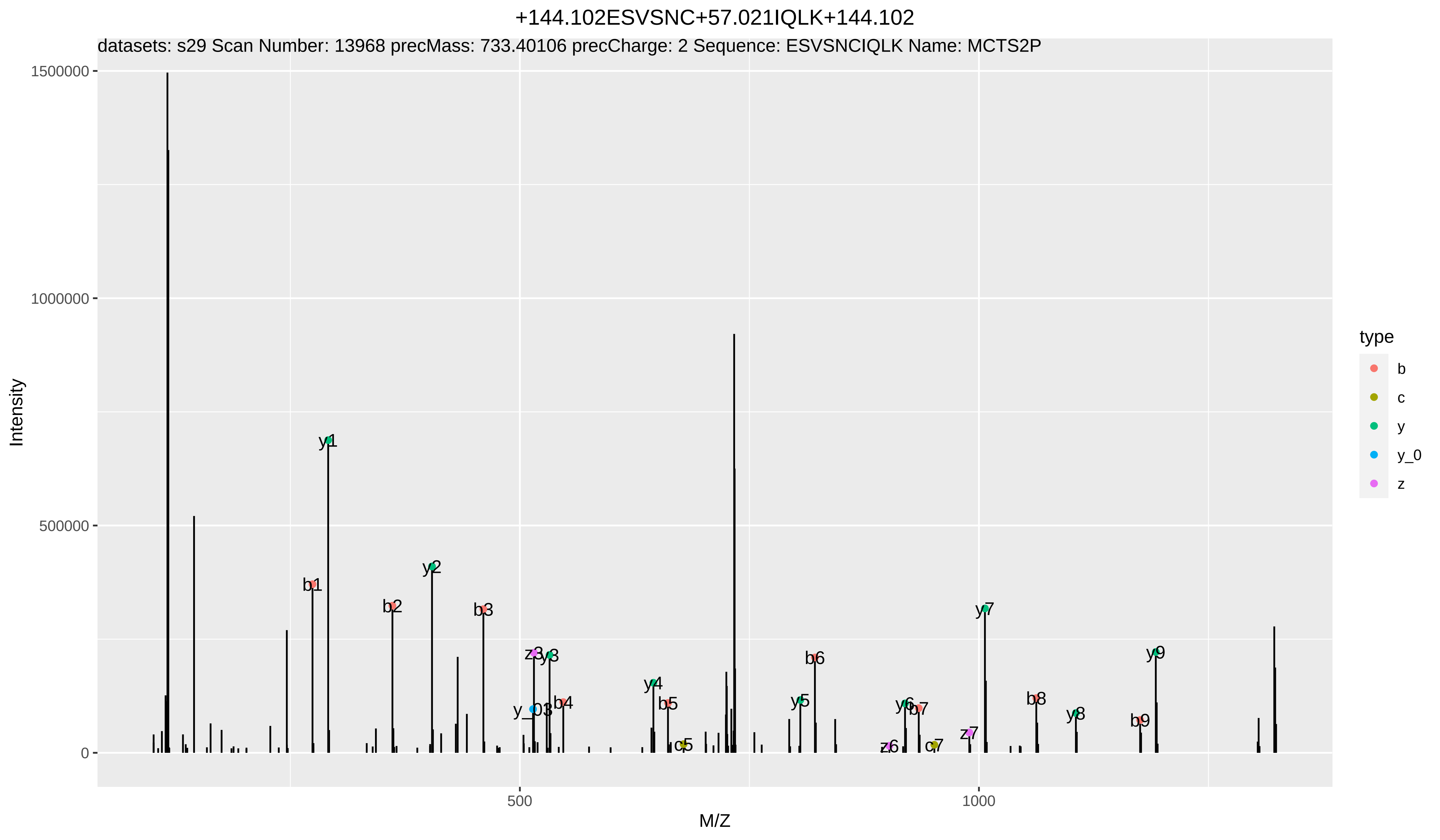

+144.102ESVSNC+57.021IQLK+144.102
datasets: s29 Scan Number: 13968 precMass: 733.40106 precCharge: 2 Sequence: ESVSNCIQLK Name: MCTS2P
Intensity
M/Z
1500000
1000000
500000
0
500
1000
y1
y2
b1
b2
b3
z3
y3
y_03
b4
y4
b5
c5
y5
b6
z6
y6
b7
c7
z7
y7
b8
y8
b9
y9
type
b
c
y
y_0
z

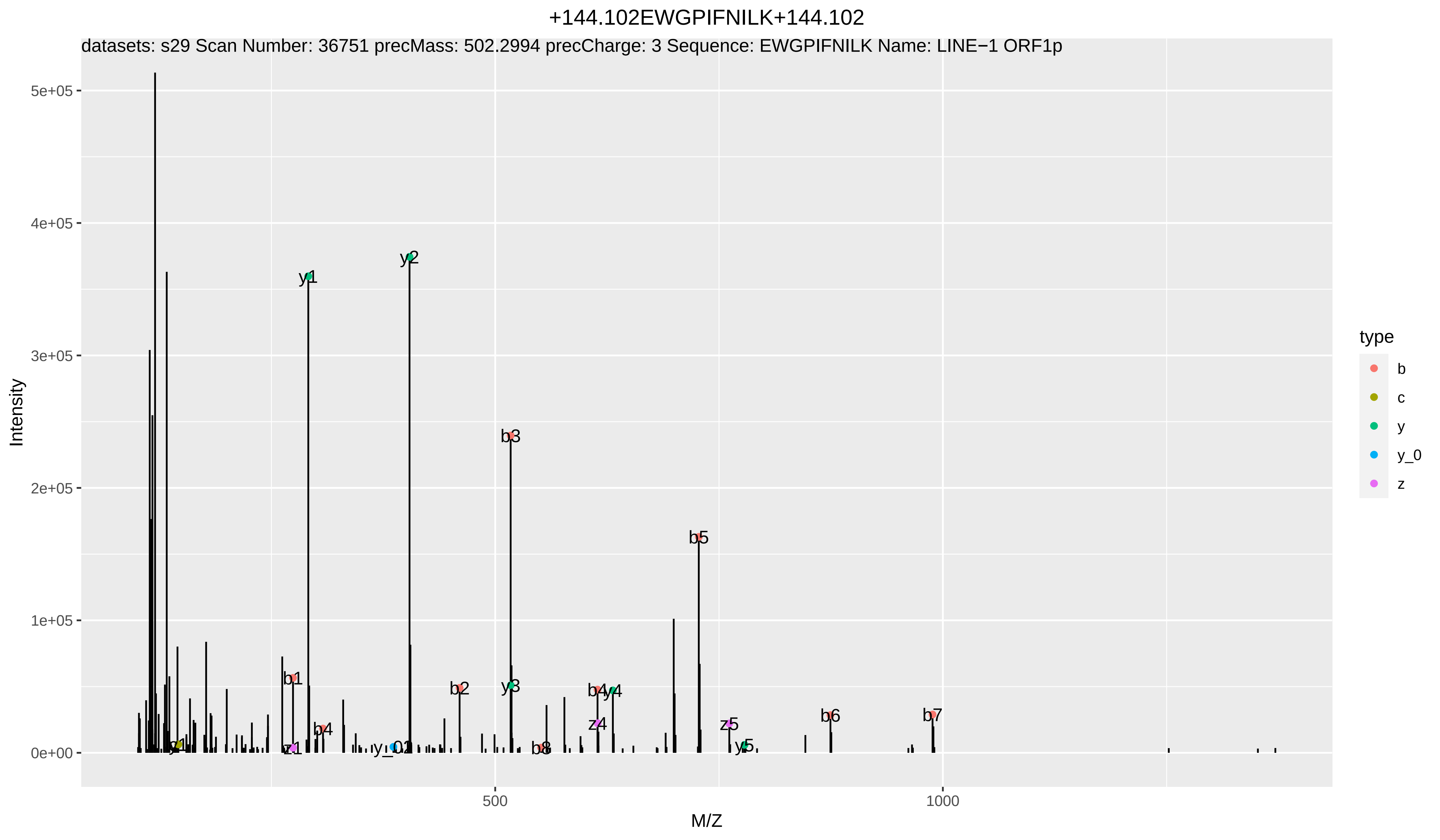
+144.102EWGPIFNILK+144.102
datasets: s29 Scan Number: 36751 precMass: 502.2994 precCharge: 3 Sequence: EWGPIFNILK Name: LINE−1 ORF1p
Intensity
M/Z
type
b
c
y
y_0
z
y1
y2
b3
b5
b1
b2
y3
b4
y4
z4
z5
b6
b7
b4
z1
y_01
b8
y5
5e+05
4e+05
3e+05
2e+05
1e+05
0e+00
500
1000

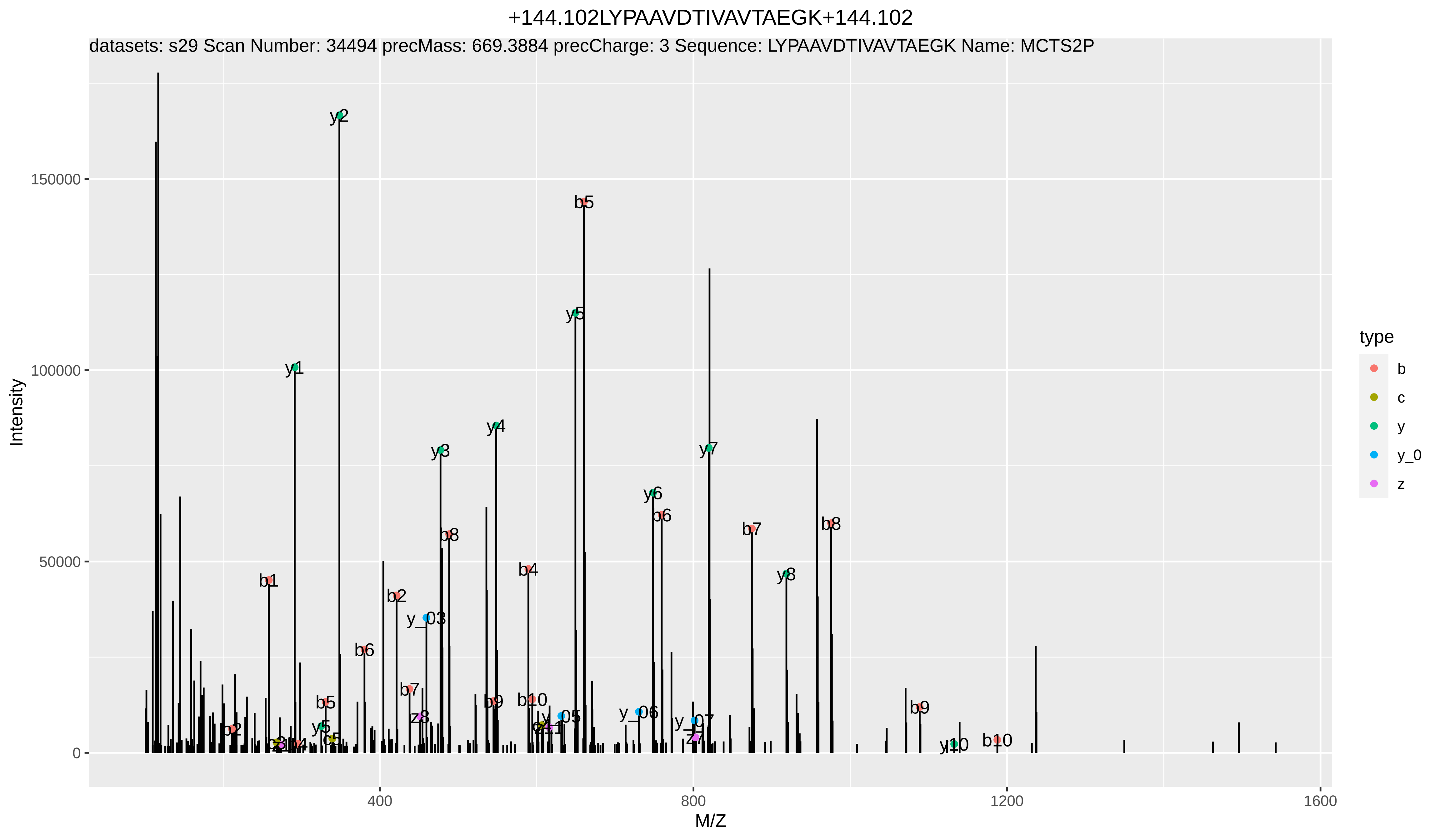

+144.102LYPAAVDTIVAVTAEGK+144.102
datasets: s29 Scan Number: 34494 precMass: 669.3884 precCharge: 3 Sequence: LYPAAVDTIVAVTAEGK Name: MCTS2P
Intensity
M/Z
type
b
c
y
y_0
z

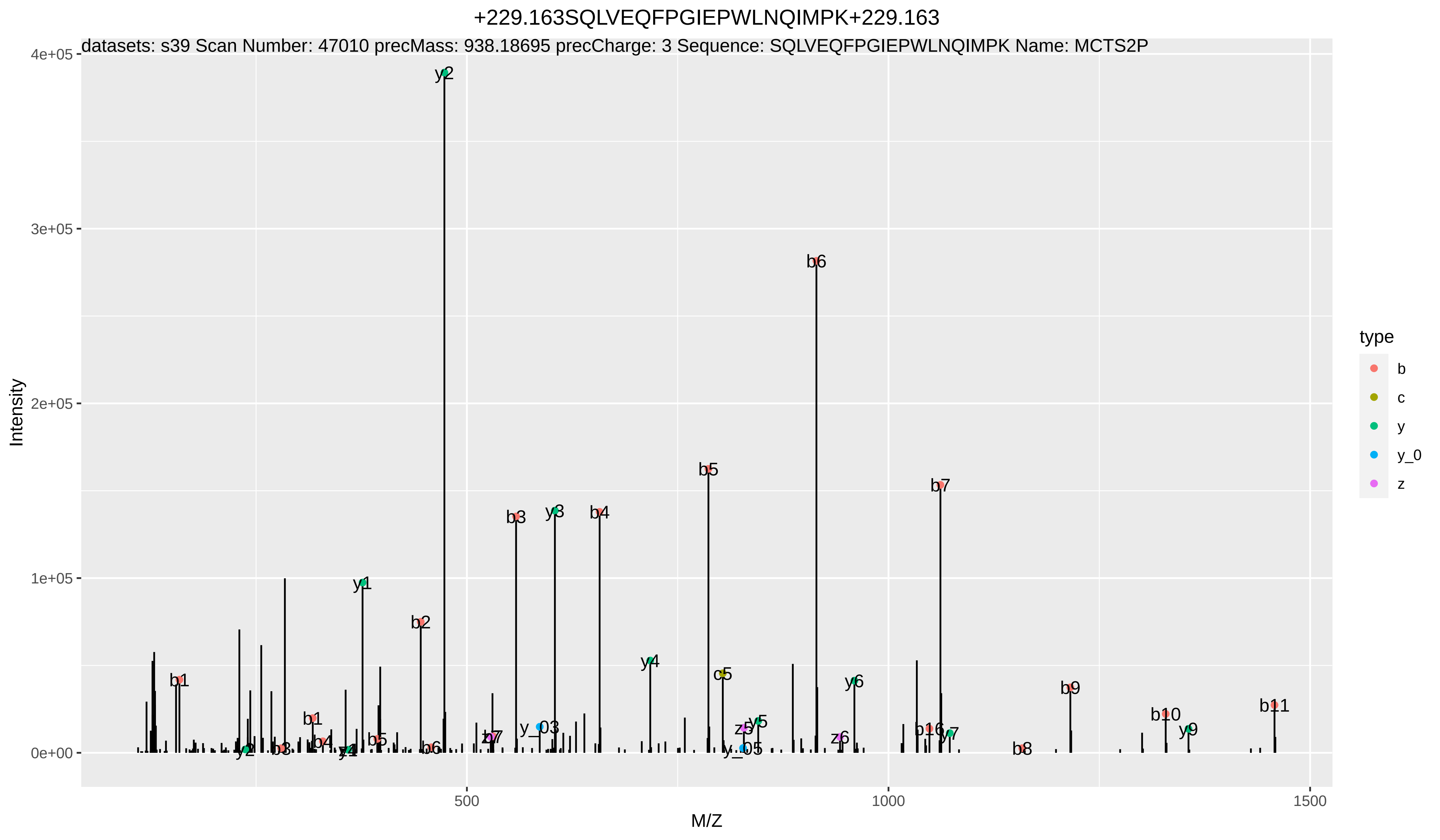

+229.163SQLVEQFPGIEPWLNQIMPK+229.163
datasets: s39 Scan Number: 47010 precMass: 938.18695 precCharge: 3 Sequence: SQLVEQFPGIEPWLNQIMPK Name: MCTS2P
Intensity
M/Z
type
b
c
y
y_0
z

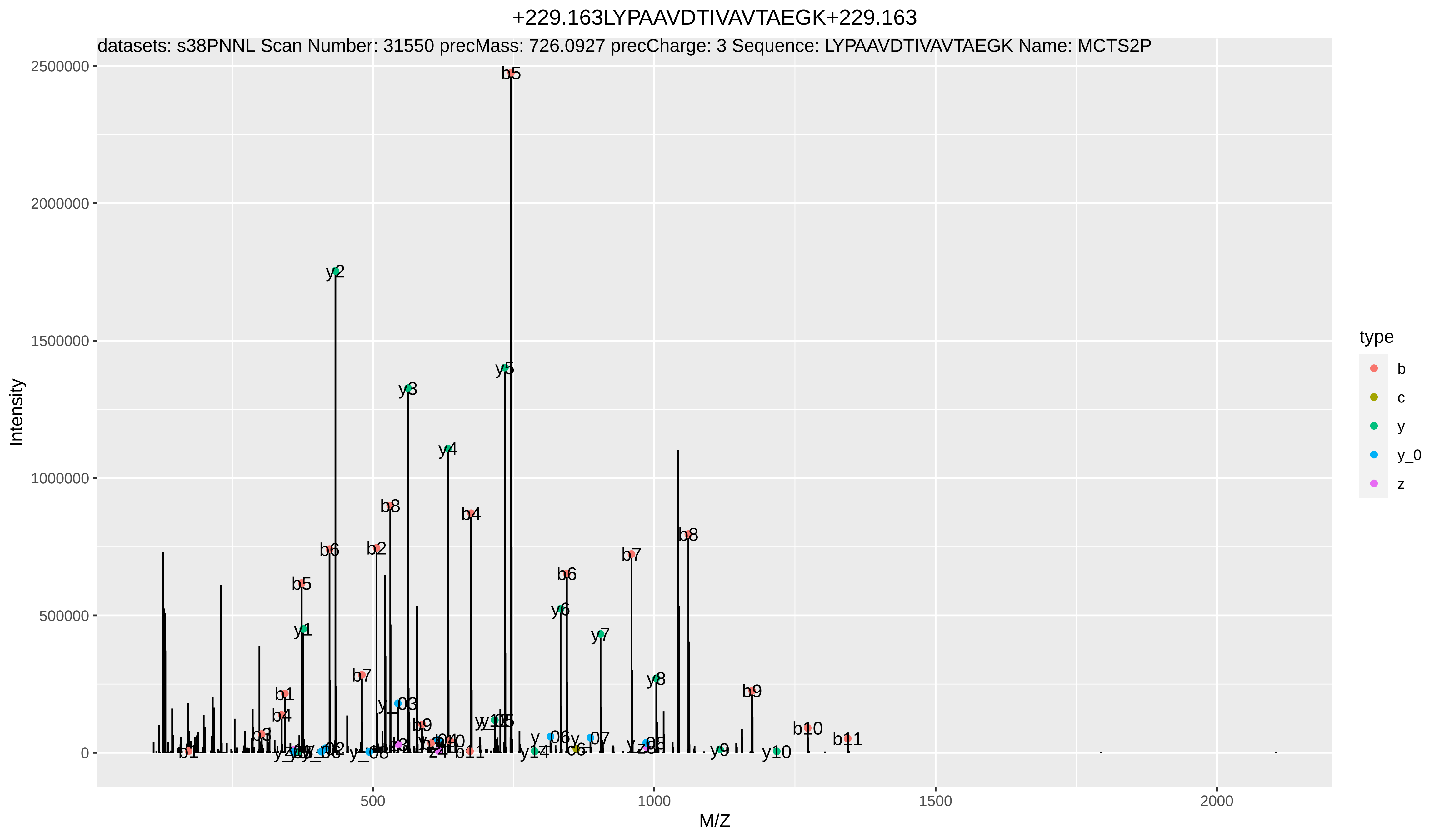
+229.163LYPAAVDTIVAVTAEGK+229.163
datasets: s38PNNL Scan Number: 31550 precMass: 726.0927 precCharge: 3 Sequence: LYPAAVDTIVAVTAEGK Name: MCTS2P
Intensity
M/Z
type
b
c
y
y_0
z

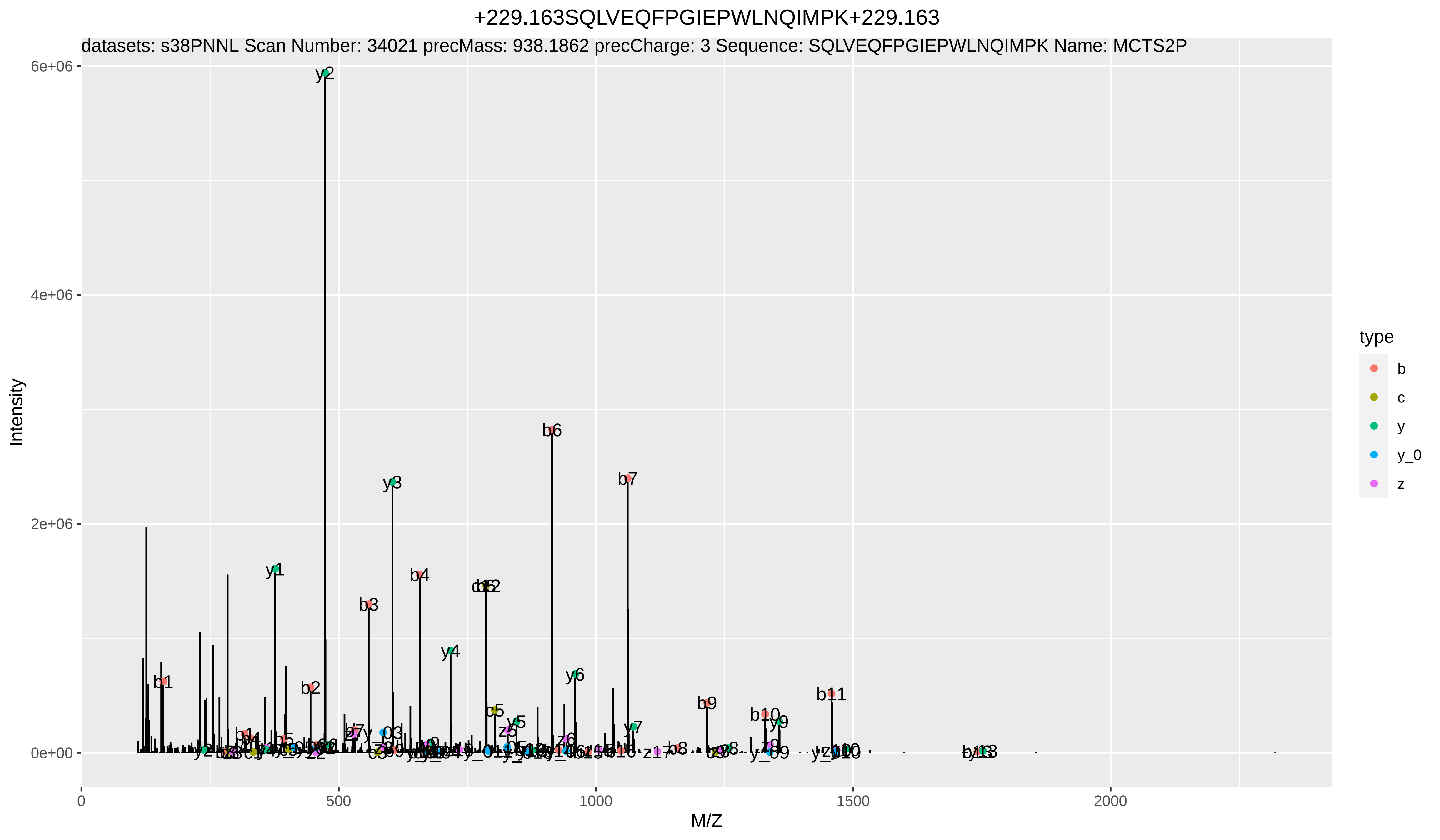

+229.163SQLVEQFPGIEPWLNQIMPK+229.163
datasets: s38PNNL Scan Number: 34021 precMass: 938.1862 precCharge: 3 Sequence: SQLVEQFPGIEPWLNQIMPK Name: MCTS2P
Intensity
M/Z
type
b
c
y
y_0
z

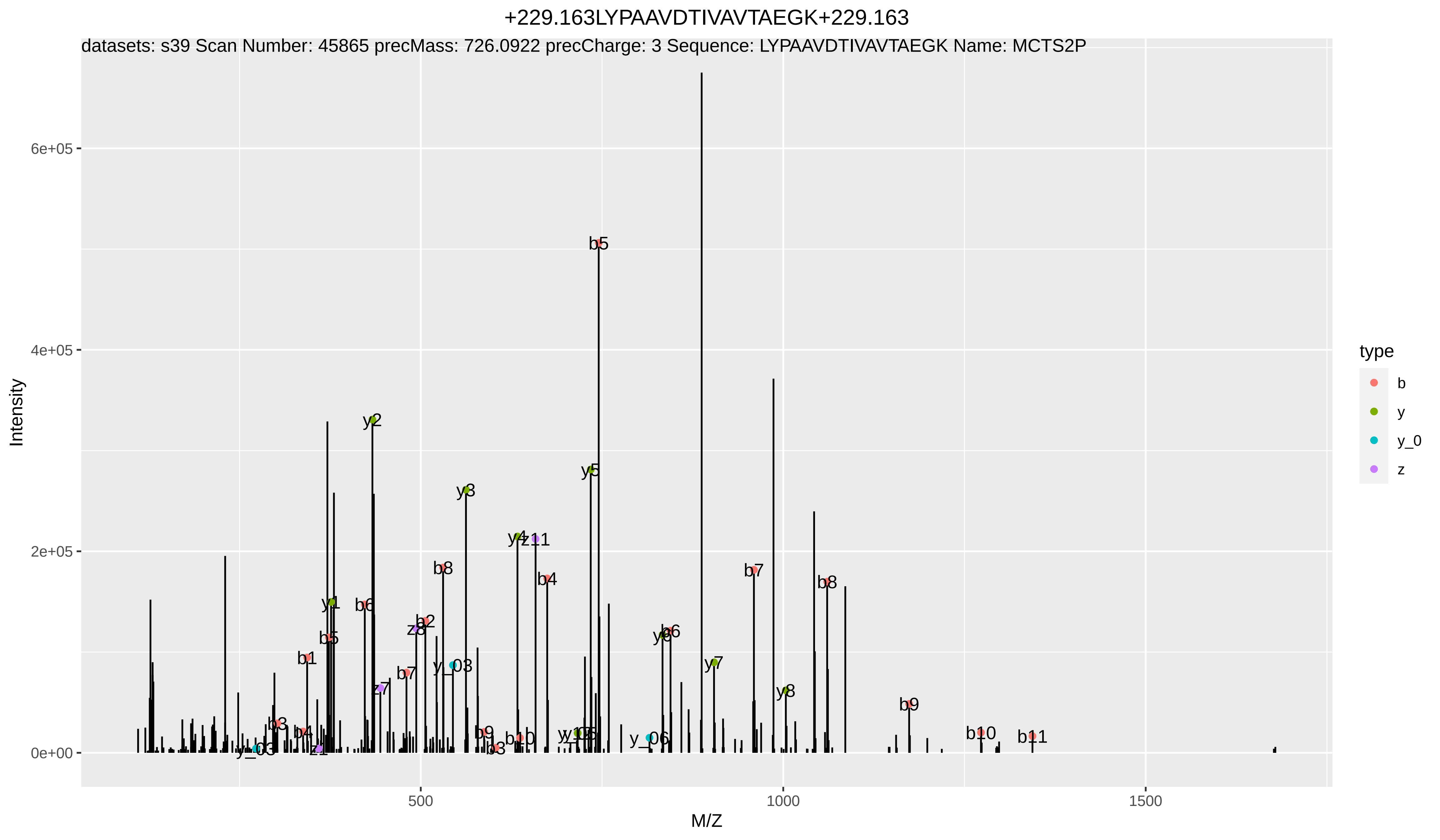

+229.163LYPAAVDTIVAVTAEGK+229.163
datasets: s39 Scan Number: 45865 precMass: 726.0922 precCharge: 3 Sequence: LYPAAVDTIVAVTAEGK Name: MCTS2P
Intensity
M/Z
6e+05
4e+05
2e+05
0e+00
500
1000
1500
type
b
y
y_0
z
b5
y2
y5
y3
y4
z11
b8
b4
b7
b8
y1
b6
b2
z8
b5
b1
y_03
b7
z7
y6
b6
y7
y8
b9
b3
b4
b9
b10
b3
y_03
z1
y_06
b10
b11

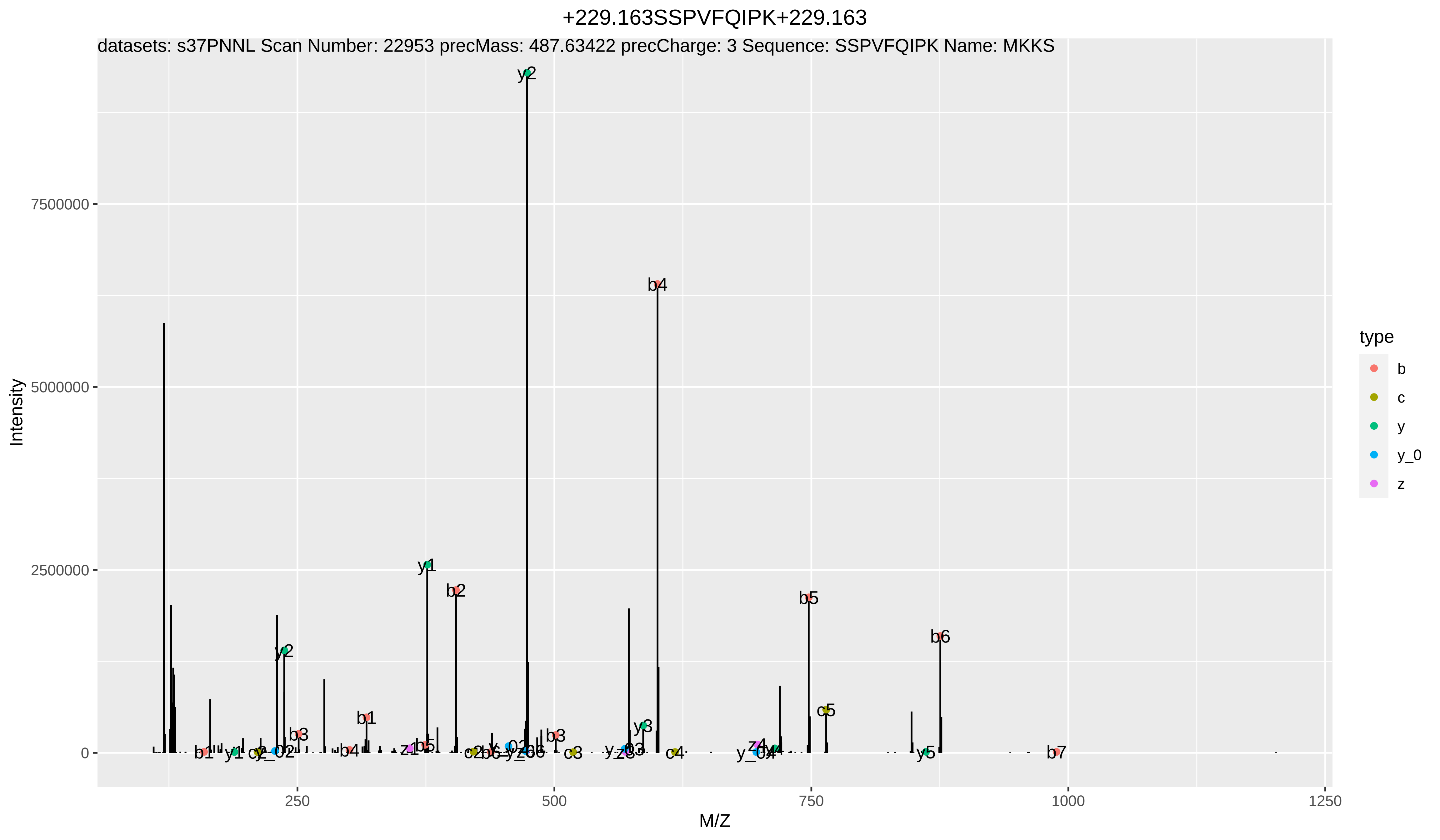
+229.163SSPVFQIPK+229.163
datasets: s37PNNL Scan Number: 22953 precMass: 487.63422 precCharge: 3 Sequence: SSPVFQIPK Name: MKKS
Intensity
7500000
5000000
2500000
0
M/Z
250
500
750
1000
1250
type
b
c
y
y_0
z
y2
b4
y1
b2
b5
b6
c5
b1
y3
b7

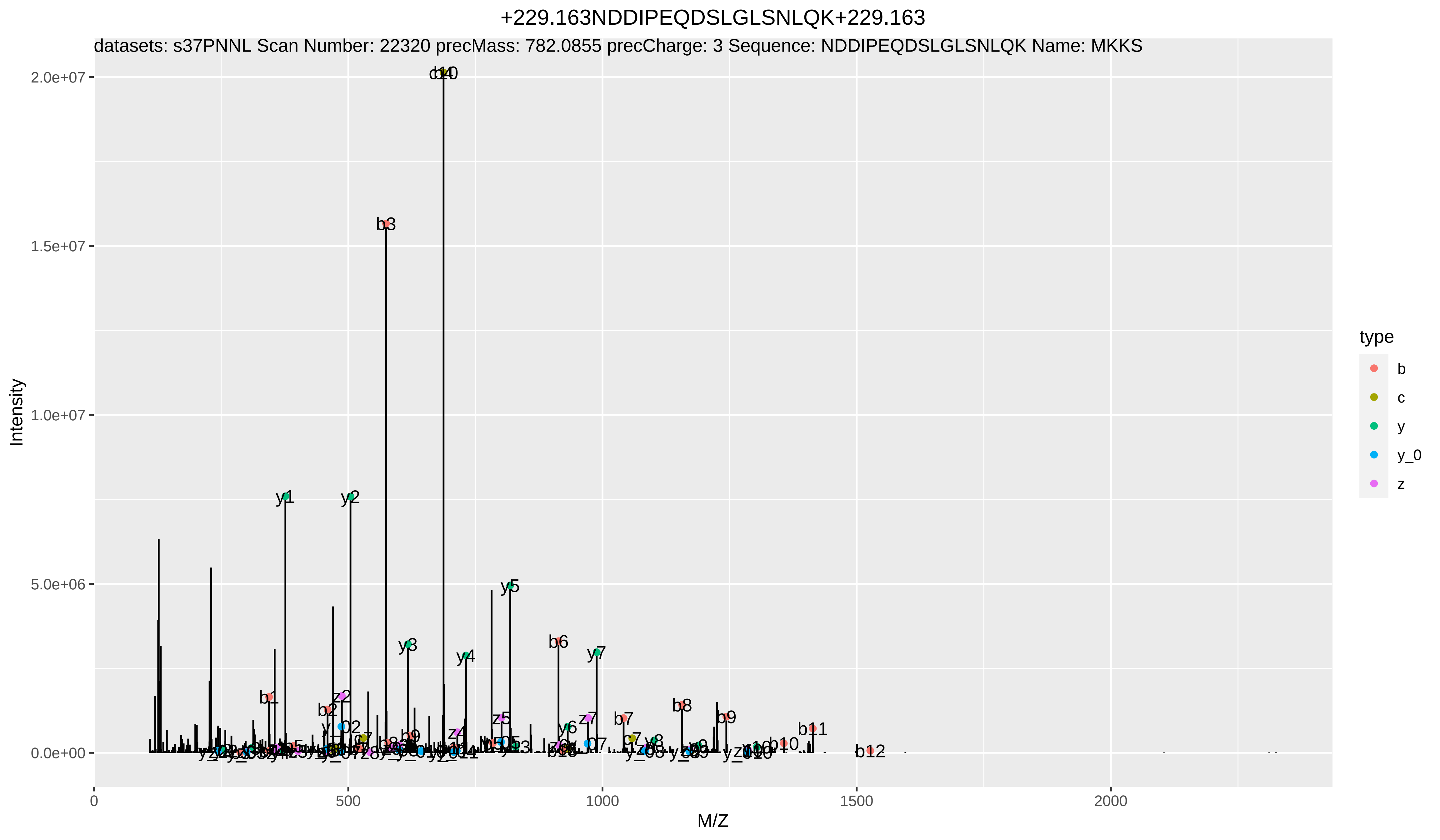
+229.163NDDIPEQDSLGLSNLQK+229.163
datasets: s37PNNL Scan Number: 22320 precMass: 782.0855 precCharge: 3 Sequence: NDDIPEQDSLGLSNLQK Name: MKKS
Intensity
2.0e+07
1.5e+07
1.0e+07
5.0e+06
0.0e+00
M/Z
0
500
1000
1500
2000
type
b
c
y
y_0
z
b3
y1
y2
y3
y4
y5
y7
b6
b8
b9
b11
b12
b7
b1
b2
z2
z5
z7
